# Iodine-Promoted C–H Bond Amination Reaction for the Synthesis of Fused Tricyclic Heteroarenes

**DOI:** 10.1021/acs.joc.4c02282

**Published:** 2024-12-13

**Authors:** Rachel E. Crittell, Rehema Nakiwala, Margaux J. Lavenue, Scott M. Hutchinson, Jeanne L. Bolliger

**Affiliations:** Department of Chemistry, 107 Physical Sciences, Oklahoma State University, Stillwater, Oklahoma 74078, United States

## Abstract

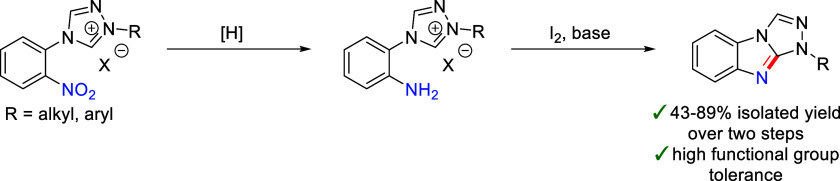

Fused heterocyclic scaffolds, such as benzimidazoles or larger ring systems containing a benzimidazole fragment, are frequently encountered in pharmaceutical compounds and other biologically active molecules. While there are many examples of N9- and/or C3-substituted 9*H*-benzo[4,5]imidazo[2,1-*c*][1,2,4]triazoles, current examples of the regioselective preparation of N1-substituted 1*H*-benzo[4,5]imidazo[2,1-*c*][1,2,4]triazoles are limited to N1-aryl substituted compounds, which also contain a C3-substituent. Here, we report an iodine-promoted C–H bond amination reaction that allows the selective preparation of 1*H*-benzo[4,5]imidazo[2,1-*c*][1,2,4]triazoles with a variety of aryl and alkyl N1-substituents. Not only do these cyclization reactions allow access to a new substitution pattern on the benzo[4,5]imidazo[2,1-*c*][1,2,4]triazole scaffold, but they are also tolerant toward a wide range of functional groups, including esters, amides, alcohols, alkynes, and alkenes. Our findings expand the synthetic toolbox for the preparation of nitrogen containing fused heteroarenes.

## Introduction

**Figure 1:**
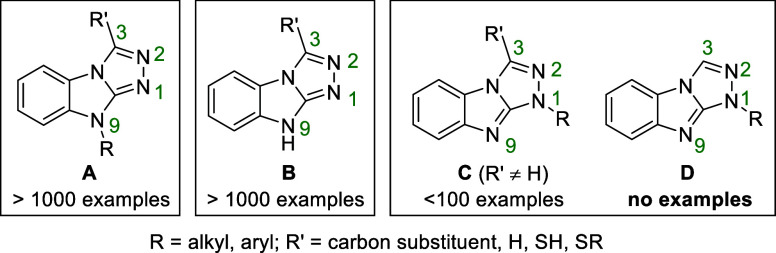
Substitution patterns of reported benzo[4,5]imidazo[2,1-*c*][1,2,4]triazoles (number of examples from SciFinder). The benzene ring may contain additional substituents, which have been omitted for clarity. The green numbers represent the locants, according to IUPAC rules.

Benzimidazoles are privileged heterocyclic scaffolds frequently encountered in pharmaceutical compounds.^1^ The synthesis of ring-fused benzimidazoles has more recently gained attention due to their promising biological activity.^2,3^ Examples of aromatic ring-fused benzimidazoles include antimalarial benzo[4,5]imidazo[1,2-*a*]pyridines,^4,5^ anticancer benzo[4,5]imidazo[1,2-*a*]pyrimidines,^6,7^ benzo[*d*]imidazo[1,2-*a*]imidazoles as COX-2 inhibitors and antioxidant agents,^8^ or benzo[4,5]imidazo[2,1-*c*][1,2,4]triazoles with activity against both Gram-positive and Gram-negative bacteria.^9−12^ The latter benzo[4,5]imidazo[2,1-*c*][1,2,4]triazole scaffold is present in molecules with various distinct substitution patterns on the imidazotriazole ring system, some of which are significantly more common than others (Figure 1).

**Scheme 1:**
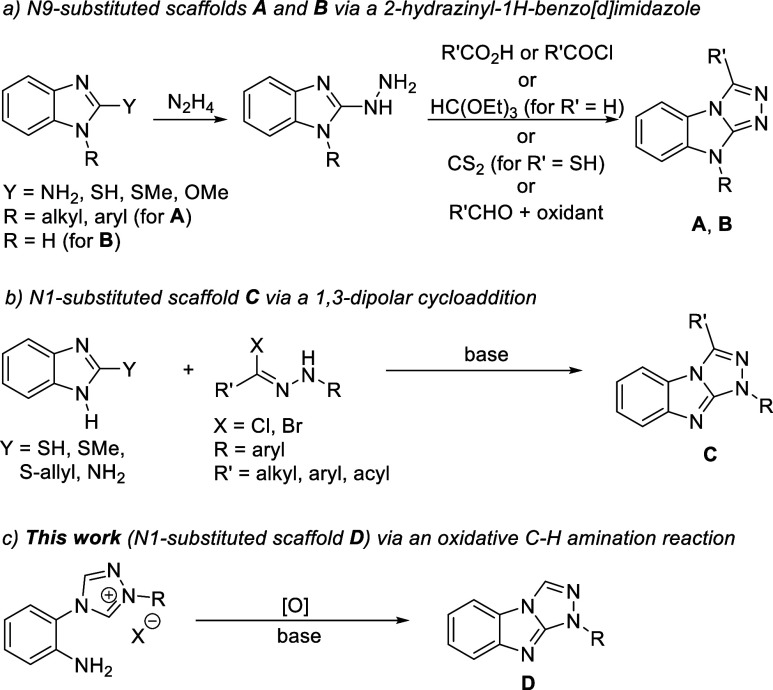
Synthetic Pathways Yielding the Benzo[4,5]imidazo[2,1-*c*][1,2,4]triazole Scaffold

Substituted scaffolds **A** and **B** are typically obtained by annulation of a 2-hydrazinyl-1-R-1*H*-benzo[*d*]imidazole derivative with the appropriate electrophile (Scheme 1a).^13−16^ Meanwhile, all examples of a selective synthesis leading to N1-substituted scaffold **C** have utilized a 1,3-dipolar cycloaddition and are limited to N-aryl substituted compounds (Scheme 1b).^9−11,17−20^ The few reported *N*-alkyl derivatives of scaffold **C** have been made by an unselective N-alkylation of 3-methyl-9*H*-benzo[4,5]imidazo[2,1-*c*][1,2,4]triazole, yielding a mixture of N1 and N9 substitution products.^21^ In this work, we report a fundamentally different synthetic approach, which allows the construction of previously unreported scaffold **D** containing *N*-alkyl or *N*-aryl substituents. Unlike in the aforementioned procedures, where the triazole ring is prepared last, we form the central imidazole ring by the construction of a new C–N bond in the final step (Scheme 1c). We discovered that this C–N bond formation can be accomplished under oxidative conditions by an iodine promoted C–H bond amination reaction from the 4-(2-aminophenyl)-4*H*-1,2,4-triazol-1-ium species.

To the best of our knowledge, the only literature example bearing similarity to our oxidative cyclization approach has been reported recently by Roy and coworkers.^22^ They describe an intermolecular C–H amination of azoles using iodine in a methanol/water mixture, which they were able to expand to the preparation of a different class of fused heterocycles (i.e., benzimidazoquinazolinones). Additionally, the synthesis of the related tricyclic imidazobenzimidazole scaffold has been achieved by a transition-metal catalyzed intramolecular C–H amination of azoles using secondary amines or azides as nitrogen sources.^23,24^

## Results and Discussion

**Scheme 2:**
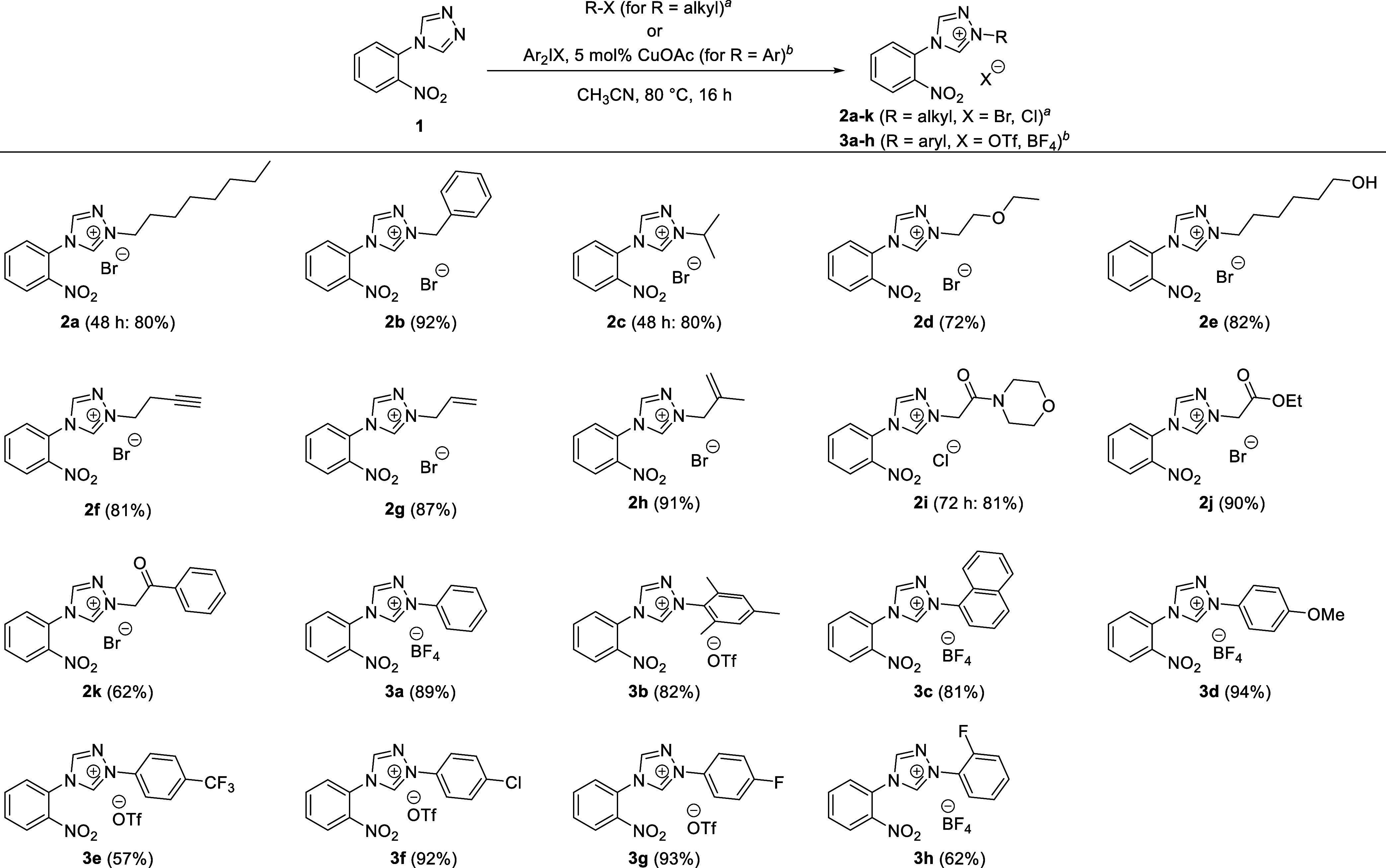
N-Alkylation and Catalytic N-Arylation of 4-(2-Nitrophenyl)-4*H*-1,2,4-triazole (**1**) Procedure for the N-alkylation of triazole **1**: triazole **1** (10.0 mmol, 1.0 equiv), alkyl halide (11.0 mmol, 1.1 equiv), 15-20 mL MeCN, 80 °C, 16 h. Procedure for the copper-catalyzed N-arylation of triazole **1**: triazole **1** (1.0 equiv), diaryliodonium salt (1.1 equiv), CuOAc (0.05 equiv), 10-25 mL MeCN, 80 °C, 16 h.

As shown in Scheme 2, N-alkylation or N-arylation of electron-deficient 4-(2-nitrophenyl)-4*H*-1,2,4-triazole (**1**) afforded the required starting materials. The 1-alkyl-4-(2-nitrophenyl)-4*H*-1,2,4-triazol-1-ium salts (**2a**–**k**) containing a wide range of functional groups on their N-alkyl substituent were obtained cleanly and in excellent yields upon heating **1** in the presence of the appropriate alkyl halide. The copper-catalyzed N-arylation of triazole **1** yielding 1-aryl-4-(2-nitrophenyl)-4*H*-1,2,4-triazol-1-ium salts (**3a**–**d**) was carried out with diaryliodonium salts using a procedure previously developed by our group.^25^ Good to excellent yields were observed with sterically unhindered and hindered electron rich aryl groups (**3a**–**d**) as well as *para*-substituted aryl halides (**3f**–**g**), while strongly electron withdrawing groups (**3e**) and *ortho*-substituted aryl halides resulted in lower yields.

**Scheme 3:**
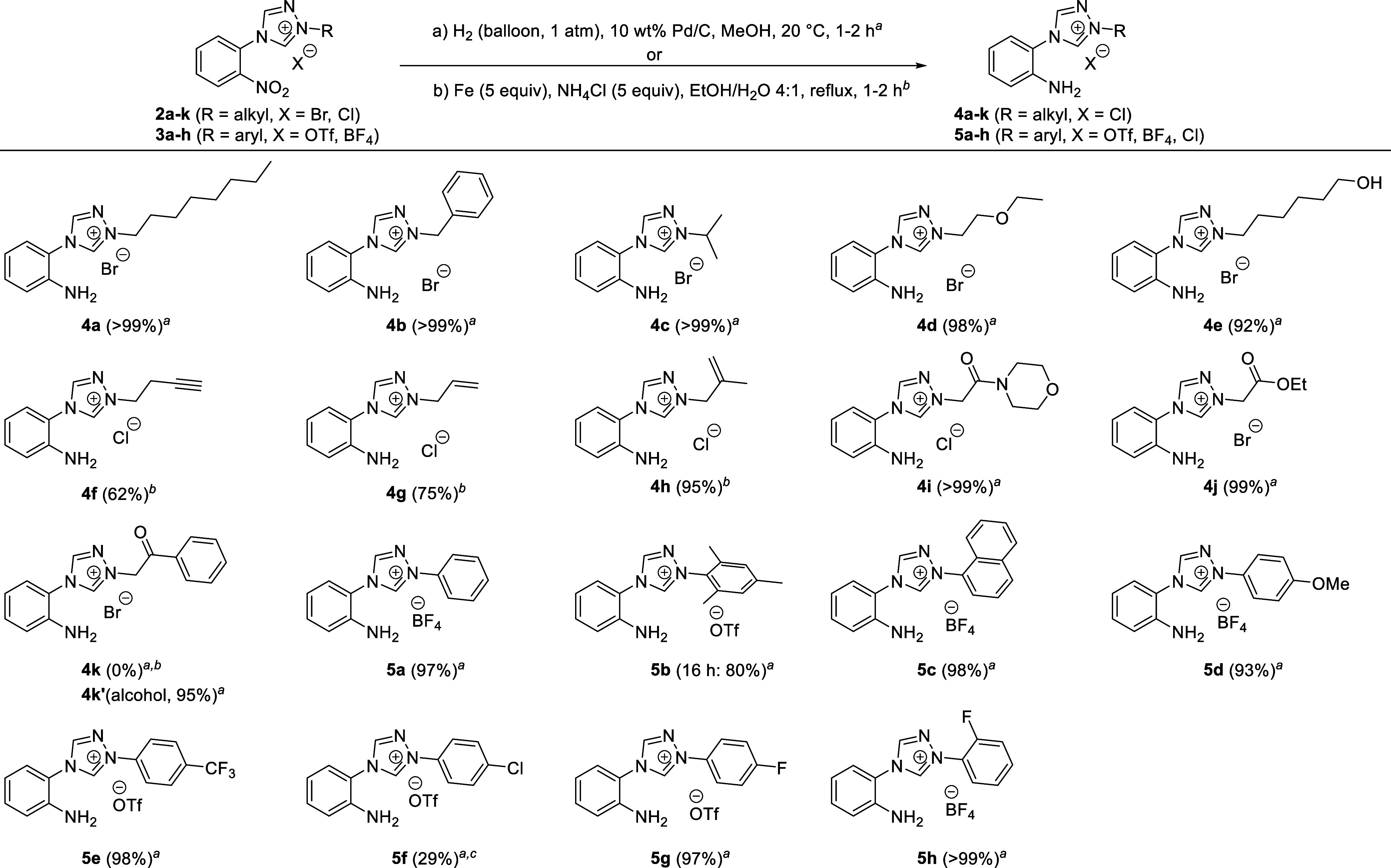
Reduction of 4-(2-Nitrophenyl)triazolium Salts to 4-(2-Aminophenyl)triazolium Salts **4a**–**k** and **5a**–**h** Procedure for the catalytic hydrogenation: 4-(2-nitrophenyl)triazolium salt **2** or **3** (1.0 equiv), 10 wt % Pd/C (10% Pd content) 6–10 mL of MeOH, H_2_ (balloon, 1 atm), 20 °C, 1–2 h. Procedure for the reduction using iron and ammonium chloride: 4-(2-nitrophenyl)triazolium salt **2** (1.0 equiv), Fe (5.0 equiv), NH_4_Cl (5.0 equiv), 10 mL of EtOH/H_2_O 4:1, reflux, 2 h. Hydrodechlorination is observed yielding the phenyl analogue as a byproduct.

Although full reduction of nitro compounds **2a**–**k** and **3a**–**h** to anilines **4a**–**k** and **5a**–**h** was easily accomplished using an excess of iron powder in the presence of ammonium chloride in a mixture of ethanol and water, the necessary aqueous extraction of the desired products proved to be challenging. Under basic conditions, a nucleophilic attack of hydroxide on C3 of the triazole followed by an oxidation (presumably by atmospheric oxygen) resulted in the rapid formation of the corresponding 2-substituted 4-(2-aminophenyl)-2,4-dihydro-3*H*-1,2,4-triazol-3-ones.^22^ Therefore, with the exception of the alkyne- and alkene-containing compounds **4f**–**h**, catalytic hydrogenation was used to obtain the pure anilines in excellent yields as illustrated in Scheme 3. A notable exception to this was **2k**, in which the activated ketone underwent reduction in parallel to the nitro group and yielded the alcohol **4k’** instead of the ketone **4k**. Furthermore, reduction of the *p*-chlorophenyl substituted species **3f** was accompanied by the partial loss of chlorine via a palladium-catalyzed hydrodechlorination.^26^ While the crude product contained about 85% of **5f** (as determined by NMR), separation from the phenyl byproduct significantly reduced the isolated yield.

With both the nitro precursors **2a**–**k** and **3a**–**h** and the fully reduced anilines **4a**–**k** and **4a**–**h** in hand, we went about exploring conditions for the oxidative cyclization reaction (Table 1) using 4-(2-aminophenyl)-1-octyl-4*H*-1,2,4-triazol-1-ium bromide (**4a**) as our test compound. As seen in entries 1 and 2, *m*-CPBA proved to be too strong of an oxidant and led to decomposition, while hydrogen peroxide led to an overoxidation of the aniline **4a** to yield the nitrobenzene **2a**. Meanwhile, DMSO proved to be too mild of an oxidant, and no reaction was observed (entry 3). However, upon the addition of an excess of iodine as an oxidant and potassium carbonate as a base to the reaction in DMSO, 81% conversion to the desired cyclization product **6a** was observed within 4 h (entry 4). Switching to potassium phosphate as a base decreased the observed conversion to 64% of **6a** (entry 5). Tertiary amines such as triethylamine (entry 6) and diethylisopropylamine (entry 7) proved to be inefficient and yielded only small amounts of the tricyclic product **6a**, whereas the amidine base DBU gave a clean transformation, resulting in over 99% conversion to the heteroarene **6a** within 4 h (entry 8). On the other hand, a very messy reaction with less than 5% conversion to the desired product was observed with potassium *tert*-butoxide as the base (entry 9). Having identified iodine as the best oxidant and DBU as the best base, a solvent screen (entries 10–13) revealed that dichloromethane could serve as an alternative to DMSO as it gave comparable results within the 4 h reaction time (entry 10), while ethanol, acetonitrile, and ethyl acetate led to the formation of other unwanted byproducts. We decided to use DMSO as the reaction solvent and last investigated the amount of iodine required. As seen in entry 14, no product was observed in the absence of iodine under conditions otherwise identical to those of entry 8. With one equivalent of iodine, 95% conversion was observed after 4 h (entry 15), and with 1.5 equiv of iodine (entry 16), the reaction was indistinguishable from the reaction with 2 equiv. A final comparison of the two best reaction solvents revealed that while the reaction in DMSO went to completion within 1 h, the reactions carried out in dichloromethane required at least 2 h for complete conversion. Therefore, we decided that the ideal conditions for the C–H amination reaction were to use DMSO as the solvent with 1.5 equiv of iodine as the oxidant and DBU as the base.

**Table 1:**
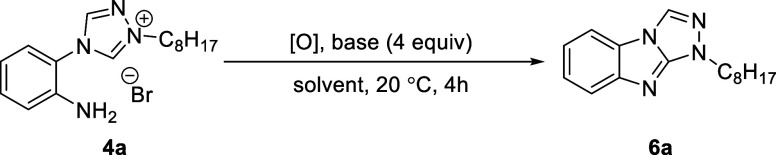
Optimization of the Oxidative Cyclization^a^

Entry	Solvent	[O]	Base	Conv.^b^
1^c^	CH_2_Cl_2_	*m*-CPBA	K_2_CO_3_	0%
2^d^	EtOH/H_2_O 1:1	H_2_O_2_ (5 equiv)	none	0%
3	DMSO	DMSO	none	0%
4	DMSO	I_2_ (2 equiv)	K_2_CO_3_	81%
5	DMSO	I_2_ (2 equiv)	K_3_PO_4_	64%
6	DMSO	I_2_ (2 equiv)	NEt_3_	<5%
7	DMSO	I_2_ (2 equiv)	NEt^i^Pr_2_	<5%
8	DMSO	I_2_ (2 equiv)	DBU	>99%
9^e^	DMSO	I_2_ (2 equiv)	KO^*t*^Bu	<5%
10	CH_2_Cl_2_	I_2_ (2 equiv)	DBU	>99%
11^e^	EtOH	I_2_ (2 equiv)	DBU	<10%
12^e^	MeCN	I_2_ (2 equiv)	DBU	<50%
13^e^	EtOAc	I_2_ (2 equiv)	DBU	<5%
14	DMSO	none	DBU	0%
15	DMSO	I_2_ (1 equiv)	DBU	>95%
16	DMSO	I_2_ (1.5 equiv)	DBU	>99%

a All reactions were performed on a 0.1 mmol scale in 3 mL of solvent under an argon atmosphere. Unless otherwise noted, the reactions proceeded cleanly.

b Conversion as determined by LCMS relative to **4a**.

c Decomposition of **4a**.

d Overoxidation to the nitro compound **2a** was observed.

e Several other byproducts were formed.

**Scheme 4:**
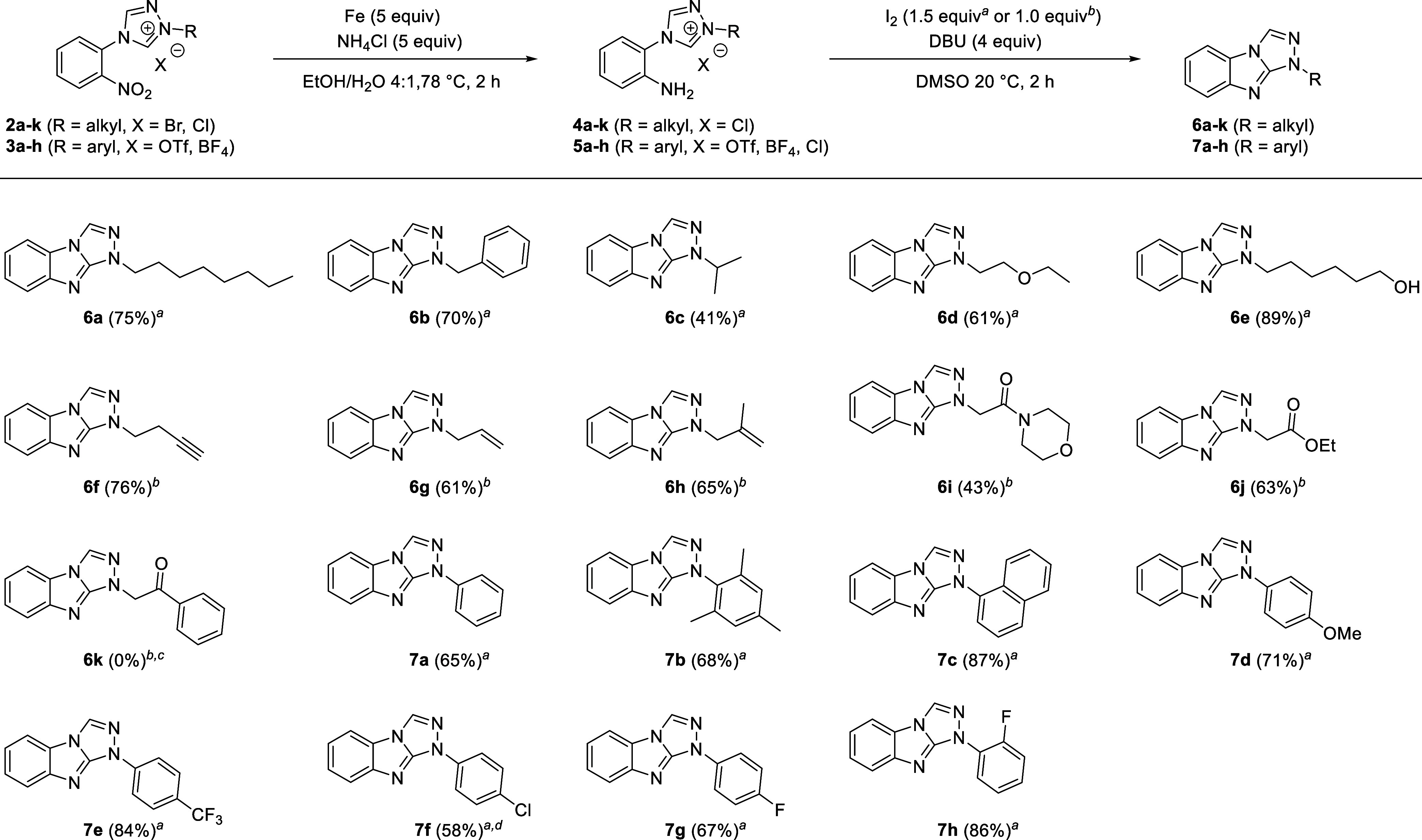
Sequential Reduction Followed by an Oxidative Cyclization Yielding Tricyclic Heteroarenes Experimental procedure using 1.5 equiv I_2_ for the oxidation step. Experimental procedure using 1.0 equiv I_2_ for the oxidation step. No cyclization product was observed but all starting material was consumed. Hydrodechlorination was observed (resulting in the formation of **7a** as the byproduct).

Rather than using the isolated anilines (Scheme 3) for carrying out the oxidative cyclization reaction, we decided to use the two-step approach shown in Scheme 4 to prepare the tricyclic compounds **6a**–**k** and **7a**–**h** directly from the nitro precursors **2a**–**k** and **3a**–**h** (see Experimental Section). By using the crude 4-(2-aminophenyl)triazolium salts **4a**–**k** and **5a**–**h** for the subsequent C–H amination step, we avoided having to make a possibly incorrect assumption about the counterion present after the reduction step. We found that the iodine-promoted C–H amination reaction is tolerant of N1-alkyl substituents containing a wide range of functional groups as well as N1-aryl substituents containing both electron-donating and electron-withdrawing groups. Isolated yields over two steps are typically greater than 65% (Scheme 4). The tricyclic heteroarenes with primary alkyl substituents on N1 are generally obtained in excellent yields (**6a**: 70%, **6b**: 75%) as are compounds carrying a primary alcohol substituent (**6e**: 89%). Lower isolated yields are observed for compounds bearing a very small alkyl substituent and are expected to give a very water-soluble 4-(2-aminophenyl)triazolium salt (e.g., **6c**: 41%, **6d**: 61%, **6g**: 61%), which leads to the loss of some of the intermediate (e.g., **4c**, **4d**, **6g**) during the necessary aqueous extraction following reduction. By lowering the amount of iodine to one equivalent, we were able to selectively conduct the iodine-promoted C–H amination reaction even in the presence of functional groups expected to react with iodine such as alkynes (**6f**) and alkenes (**6g**, **6h**). Interestingly, no iodine addition to the π bonds was observed in any of these reactions. Similarly, N1-substituents with α-hydrogen-containing amides and esters are tolerated, yielding the desired cyclization product cleanly but are isolated in lower yields (**6i**: 43%, **6j**: 63%) due to the difficulty in separating the base DBU from the desired product. The only substrate for which no cyclization product was detected even by LCMS was activated ketone **6k**: during the reduction of **2k** in the presence of iron and ammonium chloride both the nitro and carbonyl groups are reduced, as was previously observed with the palladium-catalyzed hydrogenation (Scheme 3). The subsequent oxidation with iodine gave neither **6k** nor its corresponding alcohol but rather a different compound, which we were not able to isolate and characterize. The oxidative C–H amination leading to the N1-aryl substituent cyclization products (**7a**–**7h**) generally gave good to excellent yields. One important point to note is that while the N1-alkyl substituted heteroarenes **6a**–**6j** appear to be stable for a longer period in the presence of DBU, prolonged reaction times or storage of the crude product containing DBU leads to a significant decrease in isolated yields of the N1-aryl substituted compounds **7a**–**7h**.

**Scheme 5:**
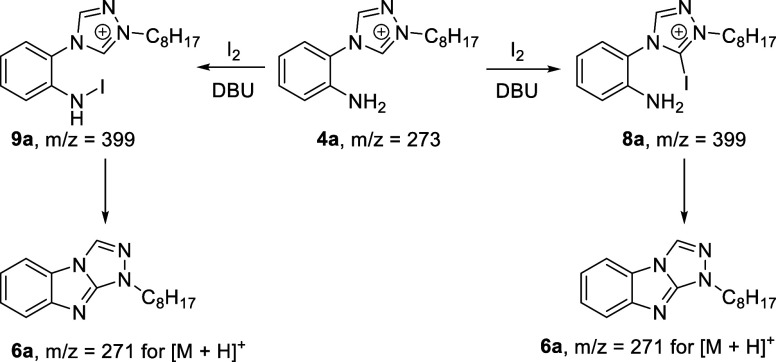
Possible Mechanisms for the Iodine-Promoted Oxidative C–H Amination

Interestingly, if the iodine is added to a solution of **4a** in DMSO before DBU, we generally observe a very clean transformation to **6a** within less than 1 min of reaction time (see Figure S126). However, if DBU is added first to **4a** in DMSO and iodine is added last, a range of other byproducts are formed including a species with *m*/*z* = 399 (see Figure S127). As shown in Scheme 5, the iodine-promoted oxidative cyclization reaction of **4a** is thought to proceed either via the 5-iodotriazolium intermediate **8a** in analogy to the mechanism proposed by Roy et al. for the amination of azoles^22^ or, alternatively, via the iodoamine intermediate **9a**. The latter case would involve an intramolecular nucleophilic attack of the carbene formed upon deprotonation of the triazolium salt on the iodoamine substituent of **9a**, thus leading to desired cyclization product **6a**. Unfortunately, both possible intermediates **8a** and **9a** have the same mass, and while we observed at least three different species by LCMS with this mass but different retention times, these were only observed when we deviated from our standard reaction protocol of adding first the iodine followed by DBU within less than 1 min to a solution of **4a** in DMSO.

## Conclusion

In conclusion, we have developed a novel procedure to access the tricyclic benzo[4,5]imidazo[2,1-*c*][1,2,4]triazole scaffold via an iodine-promoted oxidative C–H amination reaction. This new approach allows the selective synthesis of the previously unavailable N1-alkyl-substituted benzo[4,5]imidazo[2,1-*c*][1,2,4]triazoles as well as the N1-aryl analogues. Our cyclization method can be carried out in the presence of a wide range of functional groups, including alcohols, alkenes, alkynes, esters, and amides.

## Experimental Section

### Two-Step Synthesis of Heteroarenes **6a**–**6k** and **7a**–**7h** from **2a**–**2k** and **3a**–**3h**

The reagents and solvents of the 1 mmol procedure described here were scaled linearly for reactions performed on a larger scale. Reduction: a 25 mL round-bottomed flask was charged with the appropriate nitrophenyl triazolium salt (1.00 mmol, 1 equiv), Fe (279 mg, 5 mmol, 5 equiv), NH_4_Cl (267 mg, 5 mmol, 5 equiv), and 6 mL of EtOH/water 4:1. The reaction mixture was stirred in an oil bath set to 78 °C for 2 h under an argon atmosphere. After cooling to room temperature, the reaction mixture was filtered through a small plug of Celite, which was rinsed with an additional 50 mL of EtOH. After evaporation on a rotary evaporator, 30 mL of dichloromethane and 10 mL of brine were added; the organic phase was separated off, and the aqueous phase was extracted an additional three times with 30 mL of dichloromethane. All organic phases were combined, dried over anhydrous magnesium sulfate, filtered, and evaporated to dryness to yield the aminophenyl triazolium salt. Cyclization: the crude aminophenyl triazolium salt was dissolved in DMSO (2 mL). I_2_ (381 mg, 1.5 mmol, 1.5 equiv) was added followed by DBU (609 mg, 4 mmol, 4 equiv), and the reaction mixture was stirred for 2 h at room temperature. The resulting dark solution was diluted with 20 mL of 0.1 M NaOH, extracted three times with 30 mL of dichloromethane, dried over anhydrous magnesium sulfate, filtered, and concentrated. The pure cyclized product was obtained either by recrystallization or column chromatography, as described in the Supporting Information.

## Data Availability

The data underlying this study are available in the published article and its Supporting Information.

## Supporting Information Available

Complete experimental procedures including full characterization of all compounds and NMR spectra (PDF)
