# Supplementary material for: Iodine-Promoted C–H Bond Amination Reaction for the Synthesis of Fused Tricyclic Heteroarenes
Source: J Org Chem. 2024 Dec 13;90(1):344–9. doi: 10.1021/acs.joc.4c02282 (PMC11731302; doi:10.1021/acs.joc.4c02282)
Supplement: Supplementary file 1 — jo4c02282_si_001.pdf [file jo4c02282_si_001.pdf]

Supporting Information

Iodine-Promoted C–H Bond Amination Reaction for the Synthesis of Fused Tricyclic Heteroarenes

Rachel E. Crittell, Rehema Nakiwala, Margaux J. Lavenue, Scott M. Hutchinson, and Jeanne L. Bolliger*

Department of Chemistry, 107 Physical Sciences, Oklahoma State University, Stillwater, OK, 74078, United States

Email: jeanne.bolliger@okstate.edu

Contents

1. Experimental Procedures S3

1.1 General Considerations S3

1.2. Synthesis of 4-(2-Nitrophenyl)-4H-1,2,4-triazole S3

1.3. N-Alkylation of 4-(2-Nitrophenyl)-4H-1,2,4-triazole S4

1.4. N-Arylation of 4-(2-Nitrophenyl)-4H-1,2,4-triazole S4

1.5. Reduction of 4-(2-Nitrophenyl)-1-alkyl-4H-1,2,4-triazol-1-ium and 4-(2-Nitrophenyl)-1-aryl-4H-1,2,4-triazol-1-ium salts S4

1.6. Oxidative Cyclization of 4-(2-Aminophenyl)-1-alkyl-4H-1,2,4-triazol-1-ium and 4-(2-Aminophenyl)-1-aryl-4H-1,2,4-triazol-1-ium Salts S5

1.7. Two-Step Synthesis of Heteroarenes 6a-6k and 7a-7h from 2a-2k and 3a-3h S5

1.8. References S6

2. Purification and Characterization of Triazoles and Triazolium Salts S7

2.1. 4-(2-Nitrophenyl)-4H-1,2,4-triazole (1) S7

2.2. 4-(2-Nitrophenyl)-1-alkyl-4H-1,2,4-triazol-1-ium bromides and chlorides (2a-2k) S7

2.3. 4-(2-Nitrophenyl)-1-aryl-4H-1,2,4-triazol-1-ium tetrafluoroborates and trifluoromethanesulfonates (3a-3h) S11

2.4 4-(2-Aminophenyl)-1-alkyl-4H-1,2,4-triazol-1-ium bromides and chlorides (4a-4k) S13

2.5. 4-(2-Aminophenyl)-1-aryl-4H-1,2,4-triazol-1-ium tetrafluoroborates and trifluoromethanesulfonates (5a-5h) S17

2.6 1-Alkyl-1H-benzo[4,5]imidazo[2,1-c][1,2,4]triazoles(6a-6k) S20

2.7. 1-Aryl-1H-benzo[4,5]imidazo[2,1-c][1,2,4]triazoles (7a-7h) S24

3. NMR Spectra of Isolated Compounds S27

4. LCMS data S90

1. Experimental Procedures

1.1 General Considerations

General procedures. Most reagents and solvents were purchased from Fisher Scientific (Waltham, MA, United States), Oakwood Chemical (Estill, SC, United States), TCI America (Portland, OR, United States), and Avantor (Radnor, PA, United States) and were used as supplied unless otherwise noted. Thermo Scientific™ silica gel gel (for column chromatography, 0.035–0.070 mm, 60Å) from Fisher Scientific (Waltham, MA, United States) was used for chromatographic separations. Acetonitrile for (general procedure 2) was dried under inert atmosphere over CaH_2 and distilled prior to use. DMSO-d_6 was dried over molecular sieves; $CDCl_3$ was used as purchased.

Analyses. ^{1}H NMR, ^{13}C{^{1}H} NMR spectra, and ^{19}F{^{1}H} NMR spectra were all recorded on a 400 MHz Bruker Avance III spectrometer with a 5 mm liquid-state Smart Probe. Chemical shifts (δ_H, δ_C) are expressed in parts per million (ppm) and reported relative to the resonance of the residual protons of the DMSO-*d6* (δ_H = 2.50 ppm) or $CDCl_3$ (δ_H = 7.26 ppm) or in ^{13}C{^{1}H} NMR spectra relative to the resonance of the deuterated solvent DMSO-d_6 (δ_C = 39.52 ppm) or $CDCl_3$ (δ_C = 77.16 ppm). Chemical shifts in ^{19}F{^{1}H} NMR spectra are reported relative to the internal standard fluorobenzene (δ_F= -113.15). Coupling constants (J) are given in Hz. All measurements were carried out at 298 K. Abbreviations used in the description of NMR data are as follows: s, singlet; d, duplet; t, triplet; q, quart, sept, septet, m, multiplet. High Resolution Mass Spectrometry (HRMS) Data were obtained on a LTQ Orbitrap Fusion in FT Orbitrap Mode at a resolution of 240000.

1.2. Synthesis of 4-(2-Nitrophenyl)-4H-1,2,4-triazole (1)

Method 1: A 250 mL round bottomed flask was charged with formylhydrazine (6.607 g, 110 mmol, 1.1 equiv), 2-nitroaniline (13.81 g, 100 mmol, 1 equiv), 1-butanol (125 mL), and triethylorthoformate (18.3 mL, 110 mmol, 1.1 equiv), followed by methanesulfonic acid (10.57 g, 110 mmol, 1.1 equiv). After stirring for 5 minutes at room temperature, a reflux condenser was placed on the reaction flask and the reaction mixture heated to 120 ˚C under inert atmosphere in an oil bath for 2 h. The reaction mixture was cooled to room temperature and the volatiles evaporated. 200 mL of 1 M NaOH was added to the mixture which was extracted three times with 200 mL of dichloromethane. The combined extracts were dried over anhydrous magnesium sulfate, filtered, and evaporated. The crude mixture purified by column chromatography (silica gel, 1. dichloromethane (which eluted 8.570 g, 62.0 mmol of pure 2-nitroaniline), 2. dichloromethane/acetone 3:2 which eluted the desired product, R_f = 0.35). The title compound was obtained in form of a pale yellow powder in 38% (7.142 g, 37.6 mmol) yield, m.p. 127 °C (Lit.[1] 126 °C).

Method 2: According to the literature method[1] but with a reduced reaction time of 2 h, 37% yield was obtained on a 100 mmol scale. Significant decomposition resulting in lower yields is observed with longer reaction times (e.g., 5 h: 28%, 16 h: 5-14%).

1.3. N-Alkylation of 4-(2-Nitrophenyl)-4H-1,2,4-triazole

Unless mentioned otherwise, the synthesis of triazolium salts **2a-2k** was carried out on a 10 mmol scale according to the following procedure.

General Procedure 1 for the Preparation of Triazolium Salts 2a-2k

A 50 mL round bottom flask equipped with a stir bar was loaded with 1.902 g of 4-(2-nitrophenyl)-4*H*-1,2,4-triazole (**1**, 10.0 mmol, 1.0 equiv), 15-20 mL of acetonitrile, and the alkyl halide (11 mmol, 1.1 equiv). The reaction flask was fitted with a septum, placed into an oil bath set to 80 °C, and stirred for 16 h. After cooling to room temperature, the product was filtered off if it precipitated from acetonitrile; otherwise, the solvent was evaporated. The crude product was purified by washing with hexanes or diethyl ether or recrystallized from acetone as described below.

1.4. N-Arylation of 4-(2-Nitrophenyl)-4H-1,2,4-triazole

Diaryliodonium salts used in General Procedure 2 were synthesized according to existing literature procedures.[2,3]

General Procedure 2 for the Preparation of Triazolium Salts 3a-3h

A 50 mL round bottomed flask equipped with a stir bar was charged in a glovebox with 4-(2-nitrophenyl)-4*H*-1,2,4-triazole (**1**, 1.0 equiv), the diaryliodonium salt (1.1 equiv), 5 mol% of copper(I) acetate (relative to **1**), and between 10 and 25 mL of acetonitrile (depending on the reaction scale). The reaction flask was sealed with a septum, removed from the glovebox, and stirred in an oil bath at 80 ˚C overnight. After cooling to room temperature, the solvent was evaporated, and the crude product was purified by column chromatography.

1.5. Reduction of 4-(2-Nitrophenyl)-1-alkyl-4H-1,2,4-triazol-1-ium and 4-(2-Nitrophenyl)-1-aryl-4H-1,2,4-triazol-1-ium salts

General Procedure 3 for the Preparation of Anilines 4a-4e, 4i-4k, and 5a-5h from 2a-2e, 2i-2k, and 3a-3h

A 25 mL round bottomed flask was charged with the appropriate nitrophenyl triazolium salt, 10 wt% Pd/C, and 6-10 mL of MeOH. After sealing the flask with a septum, the reaction was set under hydrogen atmosphere using a new balloon filled with hydrogen gas and stirred at room temperature until TLC indicated complete consumption of the nitro starting material (typically 1-2 h). Filtration of the reaction mixture through celite gave upon evaporation the pure product.

<u>General Procedure 4</u> for the Preparation of Anilines 4f-4h

A 25 mL round bottomed flask was charged with the appropriate nitrophenyl triazolium salt (1.00 mmol, 1 equiv), Fe (5 mmol, 5 equiv), NH_4Cl (5 mmol, 5 equiv), and 10 mL of ethanol/water 4:1. The reaction was heated under an Argon atmosphere to 80 °C for 1-2 h. After cooling to room temperature, the reaction mixture was filtered through celite which was rinsed with additional ethanol until the filtrate appeared colorless. The filtrate was concentrated, 50 mL of degassed saturated sodium bicarbonate was added to the crude product, and the aqueous phase extracted four times with 50 mL of dichloromethane. The combined extracts were dried over anhydrous magnesium sulfate, filtered, and evaporated to dryness.

1.6. Oxidative Cyclization of 4-(2-Aminophenyl)-1-alkyl-4H-1,2,4-triazol-1-ium and 4-(2-Aminophenyl)-1-aryl-4H-1,2,4-triazol-1-ium Salts

<u>General Procedure 5</u> for the Oxidative Cyclization Yielding Heteroarenes 6a-6k and 7a-7h from Anilines 4a-4k and 5a-5h

The reagents and solvents of the 1 mmol procedure described here were scaled linearly for reactions performed on a larger scale.

Cyclization. The aminophenyl triazolium salt (1 mmol, 1 equiv) was dissolved in DMSO (2 mL). I_2 (381 mg, 1.5 mmol, 1.5 equiv) was added followed by DBU (609 mg, 4 mmol, 4 equiv) and the reaction mixture was stirred for 1 h at room temperature. The resulting dark solution was diluted with 20 mL of 0.1 M NaOH, extracted three times with 30 mL of dichloromethane, dried over anhydrous magnesium sulfate, filtrated, and concentrated. The pure cyclized product was obtained either by recrystallization or column chromatography as described below.

1.7. Two-Step Synthesis of Heteroarenes 6a-6k and 7a-7h from 2a-2k and 3a-3h

<u>General Procedure 6</u> for the Synthesis of Heteroarenes 6a-6k and 7a-7h from the Nitro Compounds 2a-2k and 3a-3h

The reagents and solvents of the 1 mmol procedure described here were scaled linearly for reactions performed on a larger scale.

Reduction. A 25 ml round bottomed flask was charged with the appropriate nitrophenyl triazolium salt (1.00 mmol, 1 equiv), Fe (279 mg, 5 mmol, 5 equiv), NH_4Cl (267 mg, 5 mmol, 5 equiv), and 6 mL of EtOH/water 4:1. The reaction mixture was stirred in an oil bath set to 78 °C for 2 h under an argon atmosphere. After cooling to room temperature, the reaction mixture was filtered through a small plug of celite which was rinsed with an additional 50 mL of EtOH. After evaporation on a rotary evaporator, 30 ml of dichloromethane and 10 mL of brine were added, the organic phase was separated off and the aqueous phase was extracted an additional three times with 30 mL of dichloromethane. All organic

phases were combined, dried over anhydrous magnesium sulfate, filtered, and evaporated to dryness to yield the aminophenyl triazolium salt.

Cyclization. The crude aminophenyl triazolium salt was dissolved in DMSO (2 mL). I_2 (381 mg, 1.5 mmol, 1.5 equiv) was added followed by DBU (609 mg, 4 mmol, 4 equiv) and the reaction mixture was stirred for 2 h at room temperature. The resulting dark solution was diluted with 20 mL of 0.1 M NaOH, extracted three times with 30 mL of dichloromethane, dried over anhydrous magnesium sulfate, filtrated, and concentrated. The pure cyclized product was obtained either by recrystallization or column chromatography as described below.

1.8. References

[1] S. C. Holm, A. F. Siegle, C. Loos, F. Rominger, B. F. Straub, *Synthesis* **2010**, *2010*, 2278–2286.
[2] M. Bielawski, D. Aili, B. Olofsson, *J. Org. Chem.* **2008**, *73*, 4602–4607.
[3] M. Bielawski, M. Zhu, B. Olofsson, *Adv. Synth. Catal.* **2007**, *349*, 2610–2618.

2. Purification and Characterization of Triazoles and Triazolium Salts

2.1. 4-(2-Nitrophenyl)-4H-1,2,4-triazole (1)

4-(2-nitrophenyl)-4*H*-1,2,4-triazole (1)

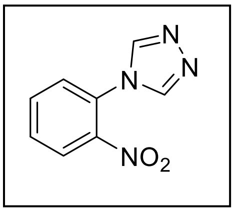

The title compound was prepared on a 100 mmol scale as described above. Purification by column chromatography (silica gel, 1. dichloromethane (which eluted 8.570 g, 62.0 mmol of pure 2-nitroaniline), 2. dichloromethane/acetone 3:2 which eluted the desired product, R_f = 0.35). The title compound was obtained in form of a pale yellow powder in 38% (7.142 g, 37.6 mmol) yield, m.p. 127 °C (Lit.[1] 126 °C).

^{1}H NMR (400 MHz, DMSO-d_6, 298 K): δ = 8.87 (s, 2H), 8.31 (d, ^3J = 8.3 Hz, 1H), 7.99-7.92 (m, 1H), 7.87-7.80 (m, 1H), 7.78 (d, ^3J = 7.8, 1H); ^{13}C{^{1}H} NMR (100 MHz, DMSO-d_6, 298 K): δ = 144.0, 143.2, 135.02, 131.0, 129.7, 127.4, 125.9. The NMR data of **1** in $CDCl_3$ is in agreement with its literature values.[1] HRMS (ESI) *m/z*: [M + H]$^+$ Calcd for $C_8H_7N_4O_2$ 191.0564; Found 191.0559.

2.2. 4-(2-Nitrophenyl)-1-alkyl-4H-1,2,4-triazol-1-ium bromides and chlorides (2a-2k)

4-(2-Nitrophenyl)-1-octyl-4*H*-1,2,4-triazol-1-ium bromide (2a)

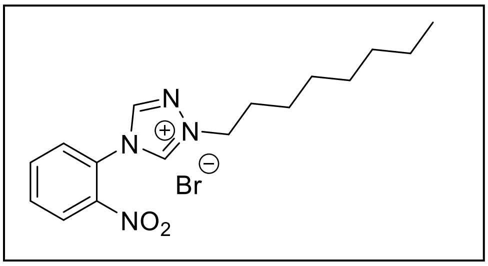

The title compound was prepared according to general procedure 1 on a 10.0 mmol scale with an extended reaction time of 48 h. The crude solid was washed with diethyl ether which yielded the product as a white powder in 80% yield (3.07 g, 8.01 mmol); m.p. 186-187 °C.

^{1}H NMR (400 MHz, DMSO-d_6, 298 K): δ = 10.62 (s, 1H), 9.61 (s, 1H), 8.49 (d, J = 8.1 Hz, 1H), 8.12-8.00 (m, 3H), 4.53 (t, J = 7.0 Hz, 2H), 2.01-1.87 (m, 2H), 1.45-1.11 (m, 10H), 0.86 (t, J = 6.4 Hz, 3H); ^{13}C{^{1}H} NMR (100 MHz, DMSO-d_6, 298 K): δ = 145.3, 143.6, 143.2, 135.8, 133.1, 130.7, 126.5, 125.2, 52.0, 31.1, 28.5, 28.3, 27.9, 25.3, 22.1, 13.9. HRMS (ESI) *m/z*: [M - Br]$^+$ Calcd for $C_{16}H_{23}N_4O_2$ 303.1816; Found 303.1820.

1-Benzyl-4-(2-nitrophenyl)-4*H*-1,2,4-triazol-1-ium bromide (2b)

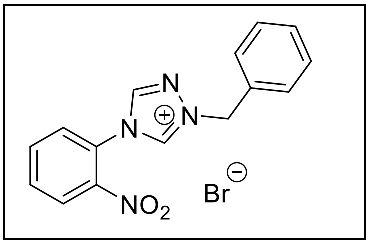

The title compound was prepared according to general procedure 1 on a 10 mmol scale. After cooling to room temperature, the solid was filtered off and recrystallized from acetone which afforded the desired product as a pale yellow powder in 92% yield (3.31 g, 9.16 mmol); m.p. 170-172 °C.

^{1}H NMR (400 MHz, DMSO-d_6, 298 K): δ = 10.76 (s, 1H), 9.63 (s, 1H), 8.49 (d, J = 8.2 Hz, 1H), 8.19-7.93 (m, 3H), 7.63-7.35 (m, 5H), 5.83 (s, 2H); ^{13}C{^{1}H} NMR (100 MHz, DMSO-d_6, 298 K): δ = 145.8, 144.0, 143.2, 135.7, 133.1, 132.9, 130.4, 129.0, 128.9, 126.5, 125.3, 55.0. HRMS (ESI) *m/z*: [M - Br]$^+$ Calcd for $C_{15}H_{13}N_4O_2$ 281.1033; Found 281.1035.

1-Isopropyl-4-(2-nitrophenyl)-4*H*-1,2,4-triazol-1-ium bromide (2c)

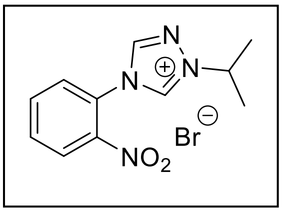

The title compound was prepared according to general procedure 1 on a 10 mmol scale with an excess of 2-bromopropane (2.82 mL, 30 mmol, 3 equiv) and an extended reaction time of 48 h. The crude product was washed with hexanes, followed by diethyl ether which yielded the product as a pale yellow powder in 80% yield (2.50 g, 7.98 mmol); m.p. 184-185 °C.

^{1}H NMR (400 MHz, DMSO-d_6, 298 K): δ = 10.63 (s, 1H), 9.47 (s, 1H), 7.37 – 7.31 (m, 2H), 6.99 (dd, *J* = 8.2, 1.3 Hz, 1H), 6.77 (td, *J* = 7.6, 1.4 Hz, 1H), 4.84 (sept, *J* = 6.6 Hz, 1H), 1.59 (d, *J* = 6.6 Hz, 6H); ^{13}C{^{1}H} NMR (101 MHz, DMSO-d_6, 298 K): δ = 145.1, 144.1, 142.6, 141.8, 132.1, 128.1, 118.4, 118.3, 55.6, 21.4. HRMS (ESI) *m/z*: [M - Br]$^+$ Calcd for $C_{11}H_{13}N_4O_2$ 233.1033; Found 233.1027.

1-(2-Ethoxyethyl)-4-(2-nitrophenyl)-4*H*-1,2,4-triazol-1-ium bromide (2d)

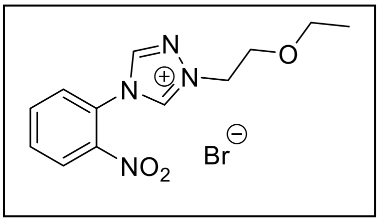

The title compound was prepared according to general procedure 1 on a 10.0 mmol scale with an excess of 1-bromo-2-ethoxyethane (4.6 g, 30 mmol, 3 equiv). The crude product was washed with hexanes followed by diethyl ether. Recrystallization from acetone yielded the product as a light yellow power in 72% yield (2.48 g, 7.23 mmol); m.p. 110-113 °C.

^{1}H NMR (400 MHz, DMSO-d_6, 298 K): δ = 10.62 (s, 1H), 9.63 (s, 1H), 8.50 (dd, *J* = 8.5, 1.5 Hz, 1H), 8.15–8.09 (m, 1H), 8.05–7.99 (m, 2H), 4.73 (t, *J* = 5.0 Hz, 2H), 3.88 (t, *J* = 5.0 Hz, 2H), 3.50 (q, *J* = 7.0 Hz, 2H), 1.09 (t, *J* = 7.0 Hz, 3H); ^{13}C{^{1}H} NMR (100 MHz, DMSO-d_6, 298 K): δ = 145.2, 144.1, 143.2, 135.8, 133.2, 130.4, 126.5, 125.2, 65.9, 65.6, 52.3, 14.8. HRMS (ESI) *m/z*: [M - Br]$^+$ Calcd for $C_{12}H_{15}N_4O_3$ 263.1139; Found 263.1143.

1-(6-Hydroxyhexyl)-4-(2-nitrophenyl)-4*H*-1,2,4-triazol-1-ium bromide (2e)

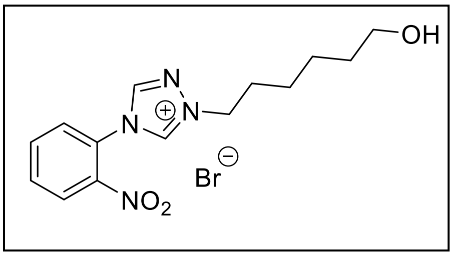

The title compound was prepared according to general procedure 1 on a 10 mmol scale. Recrystallization yielded the product as a pale yellow powder in 82% yield (3.06 g, 8.24 mmol); m.p. 104-108 °C.

^{1}H NMR (400 MHz, DMSO-d_6, 298 K): δ = 10.63 (s, 1H), 9.62 (s, 1H), 8.50 (dd, *J* = 8.2, 1.4 Hz, 1H), 8.17- 8.09 (m, 1H), 8.09-7.96 (m, 2H), 4.53 (t, *J* = 6.9 Hz, 2H), 3.39 (t, *J* = 6.3 Hz, 2H), 2.03-1.88 (m, 2H), 1.54-1.26 (m, 6H); ^{13}C{^{1}H} NMR (100 MHz, DMSO, 298 K): δ =145.4, 143.7, 143.2, 135.8, 133.2, 130.4, 126.6, 125.2, 60.5, 52.0, 32.2, 28.0, 25.2, 24.8. HRMS (ESI) *m/z*: [M - Br]$^+$ Calcd for $C_{14}H_{19}N_4O_3$ 291.1452; Found 291.1455.

1-(But-3-yn-1-yl)-4-(2-nitrophenyl)-4*H*-1,2,4-triazol-1-ium bromide (2f)

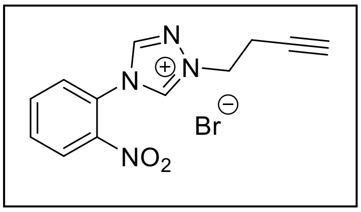

The title compound was prepared according to general procedure 1 on a 10 mmol scale with an excess of 4-bromobut-1-yne (2.00g, 15 mmol, 1.5 equiv). The crude product was washed with hexanes, followed by diethyl ether, and recrystallizedd from acetone which yielded the product as a yellow powder in 81% yield (2.62 g, 8.11 mmol); m.p. 101-108 °C.

^{1}H NMR (400 MHz, DMSO-d_6, 298 K): δ = 10.70 (s, 1H), 9.66 (s, 1H), 8.50 (dd, *J* = 8.2, 1.4 Hz, 1H), 8.12 (td, *J* = 7.7, 1.5 Hz, 1H), 8.06–7.98 (m, 2H), 4.69 (t, *J* = 6.5 Hz, 2H), 3.06 (t, *J* = 2.6 Hz, 1H), 2.93 (td, *J* = 6.5, 2.6 Hz, 2H).; ^{13}C{^{1}H} NMR (100 MHz, DMSO-d_6, 298 K): δ = 145.5, 144.1, 143.2, 135.8, 133.2, 130.3, 126.6, 125.2, 79.1, 74.4, 50.6, 18.4. HRMS (ESI) *m/z*: [M - Br]$^+$ Calcd for $C_{12}H_{11}N_4O_2$ 243.0877; Found 243.0881.

1-Allyl-4-(2-nitrophenyl)-4*H*-1,2,4-triazol-1-ium bromide (2g)

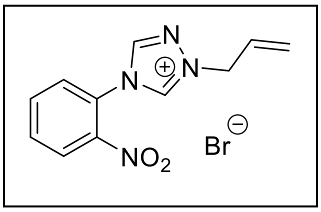

The title compound was prepared according to general procedure 1 on a 10.0 mmol scale. The crude product was recrystallized from acetone yielded the desired compound as a yellow powder in 87% yield (2.71 g, 871 mmol); m.p. 158-160 °C.

^{1}H NMR (400 MHz, DMSO-d_6, 298 K): δ = 10.69 (s, 1H), 9.66 (s, 1H), 8.50 (d, *J* = 8.2 Hz, 1H), 8.18-8.95 (m, 3H), 6.14 (ddt, *J* = 16.8, 11.4, 6.2 Hz, 1H), 5.58–5.38 (m, 2H), 5.24 (d, *J* = 6.2 Hz, 2H).; ^{13}C{^{1}H} NMR (100 MHz, DMSO-d_6, 298 K): δ = 145.5, 143.9, 143.2, 135.8, 133.2, 130.4, 130.0, 126.5, 125.2, 121.4, 54.0. HRMS (ESI) *m/z*: [M - Br]$^+$ Calcd for $C_{11}H_{11}N_4O_2$ 231.0877; Found 231.0878.

1-(2-Methylallyl)-4-(2-nitrophenyl)-4*H*-1,2,4-triazol-1-ium bromide (2h)

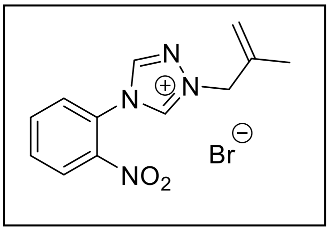

The title compound was prepared according to general procedure 1 on a 10.0 mmol scale. After filtering off the solid, recrystallization from acetone yielded the product as a yellow powder in 91% yield (2.94 g, 9.05 mmol); m.p. 185-186 °C.

^{1}H NMR (400 MHz, DMSO-d_6, 298 K): δ = 10.71 (s, 1H), 9.66 (s, 1H), 8.51 (d, *J* = 8.2 Hz, 1H), 8.18-7.98 (m, 3H), 5.20 (s, 2H), 5.17 (s, 1H), 5.05 (s, 1H), 1.78 (s, 3H); ^{13}C{^{1}H} NMR (100 MHz, DMSO-d_6, 298 K): δ = 145.6, 144.3, 143.2, 137.7, 135.8, 133.2, 130.4, 126.5, 125.2, 116.4, 57.2, 19.5. HRMS (ESI) *m/z*: [M - Br]$^+$ Calcd for $C_{12}H_{13}N_4O_2$ 245.1033; Found 245.1032.

1-(2-Morpholino-2-oxoethyl)-4-(2-nitrophenyl)-4*H*-1,2,4-triazol-1-ium chloride (2i)

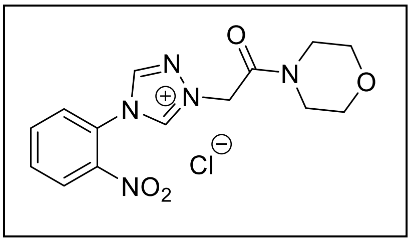

The title compound was prepared according to general procedure 1 on a 10 mmol scale with an extended reaction time of 72 h. After filtering off the solid, recrystallization from actetone yielded the product as an off-white yellow powder in 81% yield (2.85 g, 8.06 mmol); m.p. 184-186 °C.

^{1}H NMR (400 MHz, DMSO-d_6, 298 K): δ = 10.71 (s, 1H), 9.68 (s, 1H), 8.50 (d, *J* = 8.1 Hz, 1H), 8.11 (t, *J* = 7.6 Hz, 1H), 8.03 (d, *J* = 8.1 Hz, 2H), 5.84 (s, 2H), 3.83–3.44 (m, 8H); ^{13}C{^{1}H} NMR (100 MHz, DMSO-d_6, 298 K): δ = 162.6, 145.4, 145.0, 143.2, 135.8, 133.2, 130.4, 126.6, 125.3, 65.9, 53.5, 44.8, 42.2. HRMS (ESI) *m/z*: [M - Cl]$^+$ Calcd for $C_{14}H_{16}N_5O_4$ 318.1197; Found 318.1200.

1-(2-Ethoxy-2-oxoethyl)-4-(2-nitrophenyl)-4*H*-1,2,4-triazol-1-ium bromide (2j)

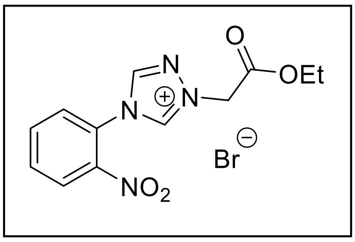

The title compound was prepared according to general procedure 1 on a 10 mmol scale. After filtering off the solid, the crude product was washed with hexanes and diethyl which yielded the desired compound as light-yellow powder in 90% yield (3.18 g, 8.89 mmol); m.p. 164-165 °C.

^{1}H NMR (400 MHz, DMSO-d_6, 298 K): δ = 10.64 (s, 1H), 9.68 (s, 1H), 8.51 (dd, *J* = 8.5, 1.5 Hz, 1H), 8.14-8.09 (m, 1H), 8.05-8.0 (m, 2H), 5.72 (s, 2H), 4.27 (q, *J* = 7.1 Hz, 2H), 1.27 (t, *J* = 7.1 Hz, 3H); ^{13}C{^{1}H} NMR (100 MHz, DMSO-d_6, 298 K): δ = 165.4, 145.4, 145.4, 143.2, 135.8, 133.3, 130.4, 126.5, 125.2, 62.3, 52.8, 13.9. HRMS (ESI) *m/z*: [M - Br]$^+$ Calcd for $C_{12}H_{13}N_4O_4$ 277.0931; Found 277.0934.

4-(2-Nitrophenyl)-1-(2-oxo-2-phenylethyl)-4*H*-1,2,4-triazol-1-ium bromide (2k)

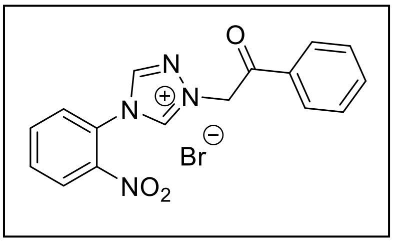

The title compound was prepared according to general procedure 1 on a 10 mmol scale. The crude product was washed with diethyl ether and recrystallized from acetone which yielded the product as a pale-yellow powder in 62% yield (2.43 g, 6.25 mmol); m.p. 198-200 °C.

^{1}H NMR (400 MHz, DMSO-d_6, 298 K): δ = 10.63 (s, 1H), 9.73 (s, 1H), 8.52 (dd, *J* = 8.2, 1.4 Hz, 1H), 8.19- 7.98 (m, 5H), 7.84-7.75 (m, 1H), 7.67 (t, *J* = 7.8 Hz, 2H), 6.54 (s, 2H); ^{13}C{^{1}H} NMR (100 MHz, DMSO-d_6, 298 K): δ = 190.0, 145.4, 145.3, 143.3, 135.8, 134.8, 133.4, 133.2, 130.4, 129.1, 128.5, 126.5, 125.3, 58.7. HRMS (ESI) *m/z*: [M - Br]$^+$ Calcd for $C_{16}H_{13}N_4O_3$ 309.0982; Found 309.0988.

2.3. 4-(2-Nitrophenyl)-1-aryl-4H-1,2,4-triazol-1-ium tetrafluoroborates and trifluoromethane-sulfonates (3a-3h)

4-(2-Nitrophenyl)-1-phenyl-4*H*-1,2,4-triazol-1-ium tetrafluoroborate (3a)

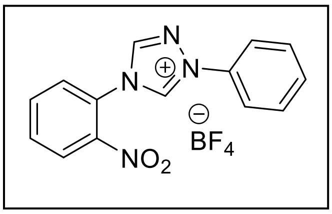

The title compound was prepared according to general procedure 2 on a 10.0 mmol scale. Purification by column chromatography (silica gel, 1. dichloromethane, 2. dichloromethane/methanol 4:1, R_f = 0.50) yielded the product as a white powder in 89% yield (3.149 g, 8.894 mmol); m.p. 201-202 °C.

^{1}H NMR (400 MHz, DMSO-d_6, 298 K): δ = 11.39 (s, 1H), 9.90 (s, 1H), 8.56 (dd, *J* = 8.3, 1.4 Hz, 1H), 8.20 (td, *J* = 7.6, 1.4 Hz, 1H), 8.16-7.97 (m, 4H), 7.82-7.75 (m, 2H), 7.74-7.67 (m, 1H).; ^{13}C{^{1}H} NMR (100 MHz, DMSO-d_6, 298 K): δ = 146.0, 143.3, 142.8, 135.9, 134.6, 133.5, 131.1, 130.5, 130.4, 126.8, 125.0, 120.8. HRMS (ESI) *m/z*: [M - BF_4]$^+$ Calcd for $C_{14}H_{11}N_4O_2$ 267.0877; Found 267.0882.

1-Mesityl-4-(2-nitrophenyl)-4*H*-1,2,4-triazol-1-ium trifluoromethanesulfonate (3b)

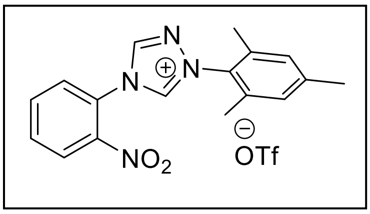

The title compound was prepared according to general procedure 2 on a 10.0 mmol scale. Purification by column chromatography (silica gel, 1. dichloromethane, 2. dichloromethane/acetone 7:3, R_f = 0.20) yielded the product as a white powder in 82% yield (3.78 g, 8.25 mmol); m.p. 120-121 °C.

^{1}H NMR (400 MHz, DMSO-d_6, 298 K): δ = 10.96 (s, 1H), 9.92 (s, 1H), 8.64 – 8.51 (d, *J* = 8.2 Hz, 1H), 8.27-8.16 (m, 2H), 8.08 (td, *J* = 7.8, 1.6 Hz, 1H), 7.23 (s, 2H), 2.38 (s, 3H), 2.18 (s, 6H); ^{13}C{^{1}H} NMR (100 MHz, DMSO-d_6, 298 K): δ = 146.3, 145.9, 143.2, 141.8, 135.9, 134.9, 133.5, 130.8, 130.7, 129.6, 126.7, 125.1, 20.7, 16.9. HRMS (ESI) *m/z*: [M - CF_3SO_3]$^+$ Calcd for $C_{17}H_{17}N_4O_2$ 309.1346; Found 309.1348.

1-(Naphthalen-1-yl)-4-(2-nitrophenyl)-4*H*-1,2,4-triazol-1-ium tetrafluoroborate (3c)

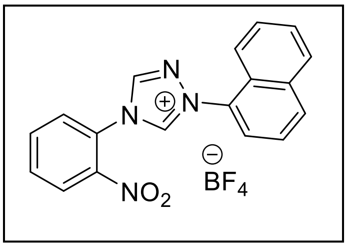

The title compound was prepared according to general procedure 2 on a 2.60 mmol scale. Purification by column chromatography (silica gel, 1. dichloromethane/methanol 20:1, 2. dichloromethane/methanol 9:1, R_f = 0.27) yielded the product as a light-yellow powder in 81% yield (844 mg, 2.09 mmol); m.p. 198-201 °C.

^{1}H NMR (400 MHz, DMSO-d_6, 298 K): δ = 11.24 (s, 1H), 10.00 (s, 1H), 8.58 (dd, *J* = 8.2, 1.3 Hz, 1H), 8.39 (dd, *J* = 8.3, 1.2 Hz, 1H), 8.27 – 8.16 (m, 3H), 8.08 (ddd, *J* = 8.3, 6.8, 2.2 Hz, 1H), 8.02 (dd, *J* = 7.4, 1.2 Hz, 1H), 7.90 – 7.75 (m, 4H); ^{13}C{^{1}H} NMR (100 MHz, DMSO-d_6, 298 K): δ = 146.2, 146.2, 143.3, 136.0, 133.8, 133.5, 132.5, 130.6, 130.5, 128.8, 128.8, 127.9, 127.0, 126.8, 125.5, 125.2, 125.2, 121.5 HRMS (ESI) *m/z*: [M - BF_4]$^+$ Calcd for $C_{18}H_{13}N_4O_2$ 317.1033; Found 317.1036.

1-(4-Methoxyphenyl)-4-(2-nitrophenyl)-4*H*-1,2,4-triazol-1-ium tetrafluoroborate (3d)

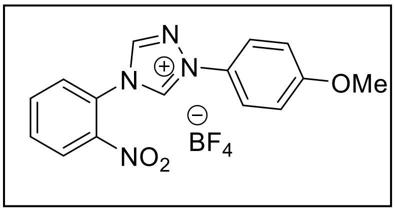

The title compound was prepared according to general procedure 2 on a 10.0 mmol scale. Purification by column chromatography (silica gel, dichloromethane/acetone 7:3, R_f = 0.17) yielded the product as a light brown powder in 94% yield (3.62 g, 9.42 mmol); m.p. 200-201 °C.

^{1}H NMR (400 MHz, DMSO-d_6, 298 K): δ = 11.24 (s, 1H), 9.84 (s, 1H), 8.53 (dd, *J* = 8.2, 1.4 Hz, 1H), 8.17 (td, *J* = 7.7, 1.5 Hz, 1H), 8.13-8.01 (m, 2H), 7.94 (d, *J* = 9.1 Hz, 2H), 7.30 (d, *J* = 9.1 Hz, 2H), 3.88 (s, 3H); ^{13}C{^{1}H} NMR (100 MHz, DMSO-d_6, 298 K): δ = 160.9, 145.8, 143.2, 142.0, 135.9, 133.4, 130.4, 127.7, 126.7, 125.1, 122.6, 115.4, 55.9. HRMS (ESI) *m/z*: [M - BF_4]$^+$ Calcd for $C_{15}H_{13}N_4O_3$ 297.0982; Found 297.0987.

4-(2-Nitrophenyl)-1-(4-(trifluoromethyl)phenyl)-4*H*-1,2,4-triazol-1-ium trifluoromethanesulfonate (3e)

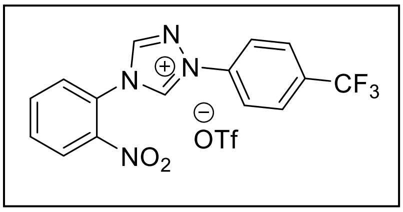

The title compound was prepared according to general procedure 2 on an 8.00 mmol scale. Purification by column chromatography (silica gel, 1. dichloromethane, 2. dichloromethane/methanol 9:1, R_f = 0.45) yielded the product as a white powder in 57% yield (1.91 g, 4.54 mmol); m.p. 155-157 °C.

^{1}H NMR (400 MHz, DMSO-d_6, 298 K): δ = 11.53 (s, 1H), 9.96 (s, 1H), 8.55 (dd, *J* = 8.2, 1.4 Hz, 1H), 8.27 (d, *J* = 8.6 Hz, 2H), 8.22-8.16 (m, 3H), 8.11-8.04 (m, 2H); ^{13}C{^{1}H} NMR (100 MHz, DMSO-d_6, 298 K): δ 146.2, 143.9, 143.2, 137.5, 135.9, 133.5, 131.0 (q, *J* = 32.8 Hz), 130.2, 127.8 (q, *J* = 3.9 Hz), 126.8, 124.9, 123.5 (q, *J* = 272.2 Hz), 121.6, 120.6 (q, *J* = 322.1 Hz, triflate); ^{19}F{^{1}H} NMR (376 MHz, DMSO-d_6, 298 K, referenced to C_6H_5F): δ = -61.36, -77.85. HRMS (ESI) *m/z*: [M - CF_3SO_3]$^+$ Calcd for $C_{15}H_{10}$ $F_3N_4O_2$ 335.0750; Found 335.0756.

1-(4-Chlorophenyl)-4-(2-nitrophenyl)-4*H*-1,2,4-triazol-1-ium trifluoromethanesulfonate (3f)

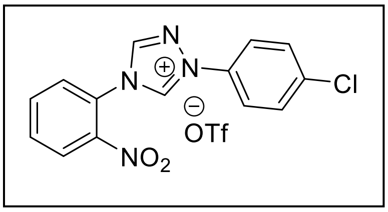

The title compound was prepared according to general procedure 2 on a 5.00 mmol scale. Purification by column chromatography (silica gel, dichloromethane/acetone 7:3, R_f = 0.11) yielded the product as an off-white powder in 92% yield (2.07 g, 4.59 mmol); m.p. 188-189 °C.

^{1}H NMR (400 MHz, DMSO-d_6, 298 K): δ = 11.41 (s, 1H), 9.91 (s, 1H), 8.54 (dd, *J* = 8.2, 1.4 Hz, 1H), 8.20-8.15 (m, 1H), 8.10-8.03 (m, 4H), 7.87 (d, *J* = 9.0 Hz, 2H); ^{13}C{^{1}H} NMR (100 MHz, DMSO-d_6, 298 K): δ = 146.0, 143.2, 143.2, 135.9, 135.5, 133.5, 133.5, 130.5, 130.3, 126.8, 125.0, 122.6. HRMS (ESI) *m/z*: [M - CF_3SO_3]$^+$ Calcd for $C_{14}H_{10}ClN_4O_2$ 301.0487; Found 301.0481.

1-(4-Fluorophenyl)-4-(2-nitrophenyl)-4*H*-1,2,4-triazol-1-ium trifluoromethanesulfonate (3g)

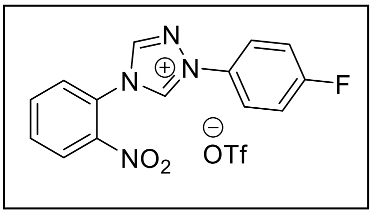

The title compound was prepared according to general procedure 2 on a 5.00 mmol scale. Purification by column chromatography (silica gel, dichloromethane/acetone 7:3, R_f = 0.11) yielded the product as an off-white powder in 93% yield (2.02 g, 4.65 mmol); m.p. 198-199 °C.

^{1}H NMR (400 MHz, DMSO-d_6, 298 K): δ = ^{1}H NMR (400 MHz, DMSO) δ 11.35 (s, 1H), 9.90 (s, 1H), 8.54 (dd, J = 8.2, 1.4 Hz, 1H), 8.18 (td, J = 7.6, 1.5 Hz, 1H), 8.13-8.01 (m, 4H), 7.65 (t, J = 8.8 Hz, 2H); ^{13}C{^{1}H} NMR (100 MHz, DMSO-d_6, 298 K): δ 162.9 (d, J = 249.1 Hz), 146.0, 143.2, 143.0, 135.9, 133.5, 131.1 (d, J = 2.7 Hz), 130.3, 126.8, 125.0, 123.5 (d, J = 9.4 Hz), 120.7 (q, J = 322.1 Hz), 117.5 (d, J = 23.9 Hz); ^{19}F{^{1}H} NMR (376 MHz, DMSO-d_6, 298 K, referenced to C_6H_5F): δ = -77.86, -109.50. HRMS (ESI) *m/z*: $[M - CF_3SO_3]^+$ Calcd for $C_{14}H_{10}FN_4O_2$ 285.0782; Found 285.0786.

1-(2-Fluorophenyl)-4-(2-nitrophenyl)-4*H*-1,2,4-triazol-1-ium tetrafluoroborate (3h)

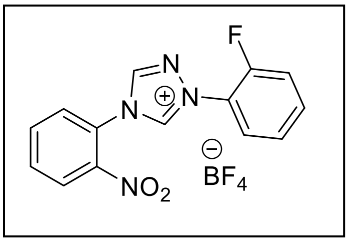

The title compound was prepared according to general procedure 2 on an 8.00 mmol scale. Purification by column chromatography (silica gel, 1. dichloromethane, 2. dichloromethane/methanol 9:1, R_f = 0.19) yielded the product as an off-white powder in 62% yield (1.83 g, 4.93 mmol); m.p. 210-213 °C.

^{1}H NMR (400 MHz, DMSO-d_6, 298 K): δ = ^{1}H NMR (400 MHz, DMSO) δ 11.23 (s, 1H), 9.94 (s, 1H), 8.55 (dd, J = 8.2, 1.4 Hz, 1H), 8.17 (td, J = 7.6, 1.4 Hz, 1H), 8.11-7.98 (m, 3H), 7.85-7.69 (m, 2H), 7.61 (td, J = 7.7, 1.5 Hz, 1H); ^{13}C{^{1}H} NMR (100 MHz, DMSO-d_6, 298 K): δ 154.0 (d, J = 253.8 Hz), 146.0, 145.6 (d, J = 6.7 Hz), 143.3, 135.9, 133.6 (d, J = 8.1 Hz), 133.5, 130.5, 126.7, 126.2 (d, J = 3.9 Hz), 125.9, 125.0, 122.7 (d, J = 10.4 Hz), 117.7 (d, J = 18.7 Hz); ^{19}F{^{1}H} NMR (376 MHz, DMSO-d_6, 298 K, referenced to C_6H_5F): δ = -122.8, -148.42. HRMS (ESI) *m/z*: $[M - BF_4]^+$ Calcd for $C_{14}H_{10}FN_4O_2$ 285.0782; Found 285.0785.

2.4 4-(2-Aminophenyl)-1-alkyl-4H-1,2,4-triazol-1-ium bromides and chlorides (4a-4k)

4-(2-Aminophenyl)-1-octyl-4*H*-1,2,4-triazol-1-ium bromide (4a)

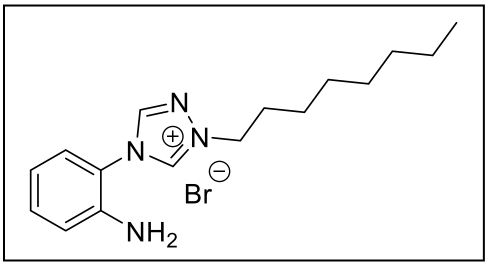

The title compound was prepared according to general procedure 3 on a 1.00 mmol scale. The product was obtained quantitatively without further purification in form of a white powder (353 mg, 1.00 mmol); m.p. 139-140 °C.

^{1}H NMR (400 MHz, $CDCl_3$, 298 K): δ = 11.08 (s, 1H), 8.48 (s, 1H), 7.21 (ddd, J = 8.4, 7.3, 1.5 Hz, 1H), 7.13 (dd, J = 7.9, 1.5 Hz, 1H), 6.89 (dd, J = 8.2, 1.3 Hz, 1H), 6.74 (td, J = 7.7, 1.3 Hz, 1H), 5.04 (bs, 2H), 4.57 (t, J

= 7.5 Hz, 2H), 2.05 (p, *J* = 7.5 Hz, 2H), 1.41 – 1.20 (m, 10H), 0.90 – 0.87 (t, *J* = 6.6 Hz, 3H); $^{13}C\{^1H\}$ NMR (100 MHz, $CDCl_3$, 298 K): δ = 143.3, 143.2, 142.9, 132.3, 126.4, 118.8, 118.5, 117.5, 53.4, 31.8, 29.2, 29.1, 28.4, 26.5, 22.7, 14.2. HRMS (ESI) *m/z*: $[M - Br]^+$ Calcd for $C_{16}H_{25}N_4$ 273.2074; Found 273.2078.

4-(2-Aminophenyl)-1-benzyl-4*H*-1,2,4-triazol-1-ium bromide (4b)

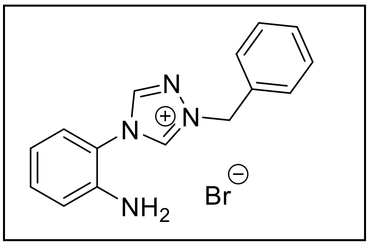

The title compound was prepared according to general procedure 3 on a 1.00 mmol scale. The pure product was obtained quantitatively without further purification in form of an off-white powder (331 mg, 1.00 mmol); m.p. 155-156 °C.

^1H NMR (400 MHz, DMSO-d_6, 298 K): δ = 10.68 (s, 1H), 9.46 (s, 1H), 7.61 – 7.54 (m, 2H), 7.48-7.40 (m, 3H), 7.36 (dd, *J* = 8.0, 1.5 Hz, 1H), 7.33 – 7.27 (m, 1H), 6.97 – 6.91 (m, 1H), 6.76 – 6.67 (m, 1H), 5.75 (s, 2H), 5.67 (s, 2H); $^{13}C\{^1H\}$ NMR (100 MHz, DMSO-d_6, 298 K): δ = 145.4, 144.0, 143.6, 132.8, 131.8, 129.3, 128.9, 128.7, 127.5, 116.7, 116.6, 116.1, 54.9. HRMS (ESI) *m/z*: $[M - Br]^+$ Calcd for $C_{15}H_{15}N_4$ 251.1291; Found 251.1295.

4-(2-Aminophenyl)-1-isopropyl-4*H*-1,2,4-triazol-1-ium bromide (4c)

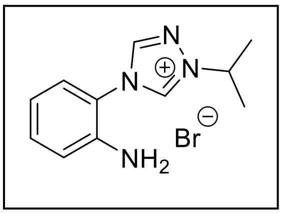

The title compound was prepared according to general procedure 3 on a 2.00 mmol scale. The pure product was obtained quantitatively without further purification as an off-white powder (568 mg, 2.00 mmol); m.p. 233-234 °C.

^1H NMR (400 MHz, DMSO-d_6, 298 K): δ = 10.51 (s, 1H), 9.44 (s, 1H), 7.36-7.29 (m, 2H), 6.93 (d, *J* = 8.2 Hz, 1H), 6.72 (t, *J* = 7.6 Hz, 1H), 5.67 (bs, 2H), 4.82 (sept, *J* = 6.6 Hz, 1H), 1.59 (d, *J* = 6.6 Hz, 6H); $^{13}C\{^1H\}$ NMR (100 MHz, DMSO-d_6, 298 K): δ = 145.0, 144.1, 142.2, 131.8, 127.6, 116.6, 116.6, 116.0, 55.4, 21.1. HRMS (ESI) *m/z*: $[M - Br]^+$ Calcd for $C_{11}H_{15}N_4$ 203.1291; Found 203.1288.

4-(2-Aminophenyl)-1-(2-ethoxyethyl)-4*H*-1,2,4-triazol-1-ium bromide (4d)

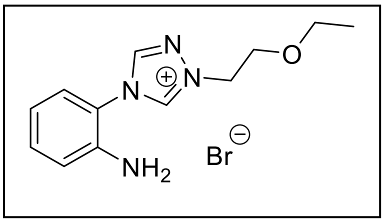

The title compound was prepared according to general procedure 3 on a 0.25 mmol scale. The product was obtained without further purification as a light yellow oil in 98% yield (77.2 mg, 0.247 mmol).

^1H NMR (400 MHz, DMSO-d_6, 298 K): δ = 10.53 (s, 1H), 9.48 (s, 1H), 7.33-7.29 m, 2H), 6.94 (dd, *J* = 8.7, 1.4 Hz, 1H), 6.74-6.70 (m, 1H), 5.73 (s, 2H), 4.60 (t, *J* = 5.2 Hz, 2H), 3.90 (t, *J* = 5.2 Hz, 2H), 3.52 (q, *J* = 7.0 Hz, 2H), 1.11 (t, *J* = 7.0 Hz, 3H).; $^{13}C\{^1H\}$ NMR (100 MHz, DMSO-d_6, 298 K): δ = 145.2, 144.0, 143.7, 131.9, 127.5, 116.7, 116.5, 116.1, 66.0, 65.6, 52.0, 14.9. HRMS (ESI) *m/z*: $[M - Br]^+$ Calcd for $C_{12}H_{17}N_4O$ 233.1397; Found 233.1398.

4-(2-Aminophenyl)-1-(6-hydroxyhexyl)-4*H*-1,2,4-triazol-1-ium bromide (4e)

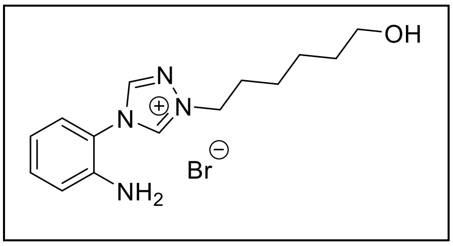

The title compound was prepared according to general procedure 3 on a 1.00 mmol scale. The product was obtained without further purification as a white powder 92% yield (315 mg, 0.922 mmol); m.p. 127-128 °C.

^{1}H NMR (400 MHz, DMSO-d_6, 298 K): δ = 10.53 (s, 1H), 9.45 (s, 1H), 7.34-7.28 (m, 2H), 6.93 (d, *J* = 8.2 Hz, 1H), 6.71 (t, *J* = 7.6 Hz, 1H), 5.73 (s, 2H), 4.43-4.34 (m, 3H), 3.40 (q, *J* = 5.9 Hz, 2H), 1.95 (p, *J* = 7.2 Hz, 2H), 1.48-1.29 (m, 6H); ^{13}C{^{1}H} NMR (100 MHz, DMSO, 298 K): δ = 145.0, 144.0, 143.3, 131.8, 127.5, 116.7, 116.5, 116.0, 60.5, 51.8, 32.2, 27.9, 25.5, 25.0. HRMS (ESI) *m/z*: [M - Br]$^+$ Calcd for $C_{14}H_{21}N_4O$ 261.1710; Found 261.1712.

4-(2-Aminophenyl)-1-(but-3-yn-1-yl)-4*H*-1,2,4-triazol-1-ium chloride (4f)

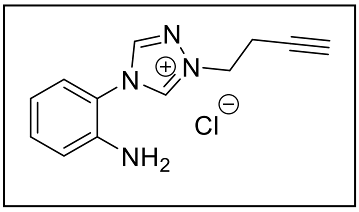

The title compound was prepared according to general procedure 4 on a 1.00 mmol scale. After washing the residue with hexanes and diethyl ether, the crude product was obtained as off-white powder in 62% yield (154 mg, 0.620 mmol); m.p. 158-161 °C.

^{1}H NMR (400 MHz, DMSO-d_6, 298 K): *δ* = 10.58 (s, 1H), 9.50 (s, 1H), 7.35-7.25 (m, 2H), 6.93 (d, *J* = 8.2 Hz, 1H), 6.72 (t, *J* = 7.6 Hz, 1H), 5.72 (s, 2H), 4.58 (t, *J* = 7.0 Hz, 2H), 3.10 (t, *J* = 2.6 Hz, 1H), 2.91 (td, *J* = 7.0, 2.6 Hz, 2H); ^{13}C{^{1}H} NMR (100 MHz, DMSO-d_6, 298 K): *δ* = 145.2, 144.1, 143.9, 131.8, 127.3, 116.8, 116.4, 116.0, 79.6, 74.1, 50.2, 18.2. HRMS (ESI) *m/z*: [M - Cl]$^+$ Calcd for $C_{12}H_{13}N_4$ 213.1135; Found 213.1138.

1-Allyl-4-(2-aminophenyl)-4*H*-1,2,4-triazol-1-ium chloride (4g)

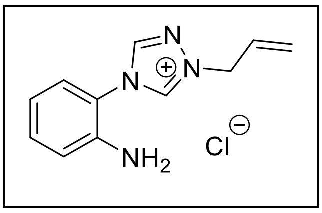

The title compound was prepared according to general procedure 4 on a 1.00 mmol scale. The compound was obtained as a pale orange oil in 75% yield (177 mg, 0.748 mmol).

^{1}H NMR (400 MHz, DMSO-d_6, 298 K): *δ* =10.54 (s, 1H), 9.46 (s, 1H), 7.35-7.26 (m, 2H), 6.92 (dt, *J* = 8.8, 1.4 Hz, 1H), 6.71 (td, *J* = 7.6, 1.4 Hz, 1H), 6.17-6.05 (m, 1H), 5.73 (s, 2H), 5.56 (dd, *J* = 17.2, 1.4 Hz, 1H), 5.47 (dt, *J* = 10.3, 1.4 Hz, 1H), 5.13- 5.05 (m, 2H); ^{13}C{^{1}H} NMR (100 MHz, DMSO-d_6, 298 K): *δ* = 145.2, 144.2, 143.7, 131.8, 130.1, 127.5, 121.5, 116.7, 116.5, 115.9, 53.9. HRMS (ESI) *m/z*: [M - Cl]$^+$ Calcd for $C_{11}H_{13}N_4$ 201.1135; Found 201.1137.

4-(2-Aminophenyl)-1-(2-methylallyl)-4*H*-1,2,4-triazol-1-ium chloride (4h)

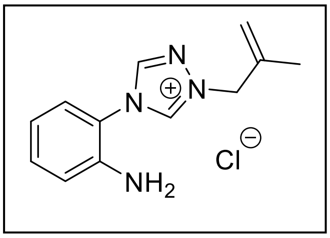

The title compound was prepared according to general procedure 4 on a 1.00 mmol scale. The product was obtained as a pale brown oil in 95% yield (238 mg, 0.949 mmol).

^{1}H NMR (400 MHz, DMSO-d_6, 298 K): δ = 10.56 (s, 1H), 9.49 (s, 1H), 7.38-7.29 (m, 2H), 6.93 (dd, J = 8.3, 1.3 Hz, 1H), 6.72 (ddd, J = 8.3, 7.3, 1.3 Hz, 1H), 5.72 (s, 2H), 5.17-5.13 (m, 2H), 5.01 (s, 2H), 1.81 (s, 3H); ^{13}C{^{1}H} NMR (100 MHz, DMSO-d_6, 298 K): δ = 145.5, 144.0, 143.9, 137.6, 131.9, 127.6, 116.9, 116.7, 116.6, 116.1, 57.2, 19.8. HRMS (ESI) *m/z*: [M - Cl]$^+$ Calcd for $C_{12}H_{15}N_4$ 215.1291; Found 215.1293.

4-(2-Aminophenyl)-1-(2-morpholino-2-oxoethyl)-4*H*-1,2,4-triazol-1-ium chloride (4i)

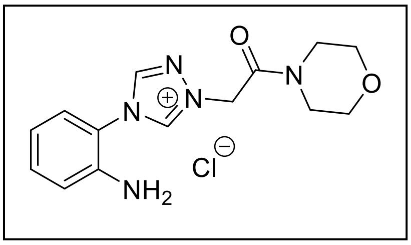

The title compound was prepared according to general procedure 3 on a 0.50 mmol scale. The product was obtained without further purification as an off-white powder in >99% yield (161 mg, 0.498 mmol); m.p. 194-195 °C.

^{1}H NMR (400 MHz, DMSO-d_6, 298 K): δ = 10.54 (s, 1H), 9.52 (s, 1H), 7.39-7.25 (m, 2H), 6.94 (d, J = 8.2 Hz, 1H), 6.74 (t, J = 7.6 Hz, 1H), 5.69 (s, 4H), 3.73-3.47 (m, 8H); ^{13}C{^{1}H} NMR (100 MHz, DMSO-d_6, 298 K): δ = 162.8, 145.2, 144.8, 144.0, 131.9, 127.2, 117.0, 116.5, 116.2, 65.9, 53.1, 44.9, 42.2. HRMS (ESI) *m/z*: [M - Cl]$^+$ Calcd for $C_{14}H_{18}N_5O_2$ 288.1455; Found 288.1458.

4-(2-Aminophenyl)-1-(2-ethoxy-2-oxoethyl)-4*H*-1,2,4-triazol-1-ium bromide (4j)

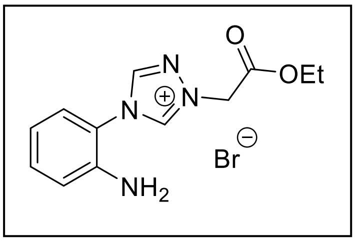

The title compound was prepared according to general procedure 3 on a 1.00 mmol scale. The product was obtained without further purification as a off-white powder in >99% yield (326 mg, 0.997 mmol); m.p. 75-76 °C.

^{1}H NMR (400 MHz, DMSO-d_6, 298 K): δ = 10.60 (s, 1H), 9.56 (s, 1H), 7.40-7.27 (m, 2H), 6.95 (d, J = 8.2 Hz, 1H), 6.73 (t, J = 7.5 Hz, 1H), 5.72 (s, 2H), 5.58 (s, 2H), 4.27 (q, J = 7.2 Hz, 2H), 1.27 (t, J = 7.2 Hz, 3H); ^{13}C{^{1}H} NMR (100 MHz, DMSO-d_6, 298 K): δ = 165.4, 145.3, 145.1, 143.9, 131.9, 127.3, 116.9, 116.4, 116.3, 62.2, 52.6, 14.0. HRMS (ESI) *m/z*: [M - Br]$^+$ Calcd for $C_{12}H_{15}N_4O_2$ 247.1190; Found 247.1190.

4-(2-Aminophenyl)-1-(2-oxo-2-phenylethyl)-4*H*-1,2,4-triazol-1-ium bromide (4k)

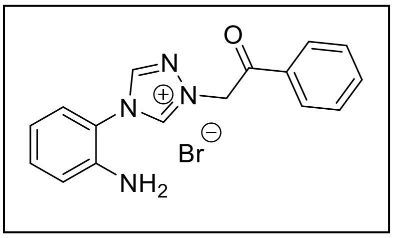

The title compound could not be isolated in pure form. General procedure 3 resulted in **4k'** instead. With general procedure 4, the presence of the title compound (among other compounds) was confirmed by HRMS.

HRMS (ESI) *m/z*: $[M - Br]^+$ Calcd for $C_{16}H_{15}N_4O$ 279.1240; Found 279.1243.

4-(2-Aminophenyl)-1-(2-hydroxy-2-phenylethyl)-4*H*-1,2,4-triazol-1-ium bromide (4k')

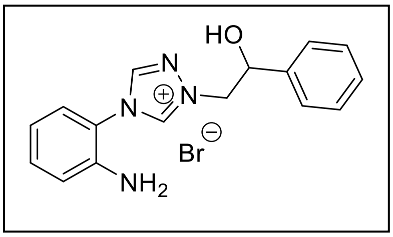

The title compound was prepared according to general procedure 3 on a 1.00 mmol scale. The product was obtained without further purification as an off-white powder in 95% yield (341 mg, 0.946 mmol); m.p. 188-189 °C.

^1H NMR (400 MHz, DMSO-d_6, 298 K): δ = 10.60 (s, 1H), 9.53 (s, 1H), 7.57 – 7.48 (m, 2H), 7.43 (dd, *J* = 8.2, 6.6 Hz, 2H), 7.39 – 7.27 (m, 3H), 6.95 (dd, *J* = 8.2, 1.3 Hz, 1H), 6.74 (ddd, *J* = 8.2, 7.3, 1.3 Hz, 1H), 6.01 (bs, 1H), 5.71 (bs, 2H), 5.14 (dd, *J* = 9.5, 3.0 Hz, 1H), 4.64 (dd, *J* = 13.6, 3.0 Hz, 1H), 4.48 (dd, *J* = 13.6, 9.5 Hz, 1H); $^{13}C\{^1H\}$ NMR (100 MHz, DMSO-d_6, 298 K): δ = 145.3, 144.0, 144.0, 140.5, 131.9, 128.5, 128.0, 127.4, 126.2, 116.8, 116.5, 116.2, 69.7, 59.0. HRMS (ESI) *m/z*: $[M - Br]^+$ Calcd for $C_{16}H_{17}N_4O$ 281.1397; Found 281.1391.

2.5. 4-(2-Aminophenyl)-1-aryl-4H-1,2,4-triazol-1-ium tetrafluoroborates and trifluoromethane-sulfonates (5a-5h)

4-(2-Aminophenyl)-1-phenyl-4*H*-1,2,4-triazol-1-ium tetrafluoroborate (5a)

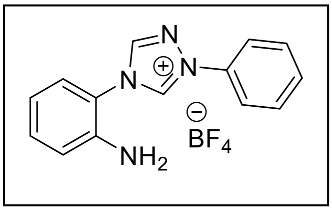

The title compound was prepared according to general procedure 3 on a 0.50 mmol scale. The product was obtained without further purification as an off-white powder in 97% yield (157 mg, 0.486 mmol); m.p. 195-196 °C.

^1H NMR (400 MHz, DMSO-d_6, 298 K): δ = 11.28 (s, 1H), 9.71 (s, 1H), 8.04-7.99 (m, 2H), 7.92 – 7.78 (m, 2H), 7.43 – 7.29 (m, 2H), 6.99 – 6.90 (m, 1H), 6.76 (t, *J* = 7.6 Hz, 1H), 5.81 (s, 2H); $^{13}C\{^1H\}$ NMR (100 MHz, DMSO-d_6, 298 K): δ = 145.8, 144.2, 142.9, 135.1, 133.9, 132.3, 130.4, 127.6, 122.5, 116.6, 116.2, 116.1; HRMS (ESI) *m/z*: $[M - BF_4]^+$ Calcd for $C_{14}H_{13}N_4$ 237.1135; Found 237.1138.

4-(2-Aminophenyl)-1-mesityl-4*H*-1,2,4-triazol-1-ium trifluoromethanesulfonate (5b)

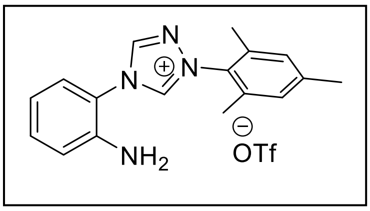

The title compound was prepared according to general procedure 3 on a 0.10 mmol scale with an extended reaction time of 16 h. The product was obtained without further purification as an off-white powder in 80% yield (34.3 mg, 0.0801 mmol); m.p. 140-141 °C. ^{1}H NMR (400 MHz, DMSO-d_6, 298 K): δ = 10.89 (s, 1H), 9.82 (s, 1H), 7.49 (dd, *J* = 8.0, 1.6 Hz, 1H), 7.36 (ddd, *J* = 8.5, 7.3, 1.6 Hz, 1H), 7.19 (s, 2H), 7.00 (dd, *J* = 8.3, 1.3 Hz, 1H), 6.80 (ddd, *J* = 8.3, 7.3, 1.3 Hz, 1H), 5.76 (s, 2H), 2.36 (s, 3H), 2.21 (s, 6H); ^{13}C{^{1}H} NMR (100 MHz, DMSO-d_6, 298 K): δ = 146.1, 145.2, 143.9, 141.3, 134.7, 132.1, 131.0, 129.6, 127.9, 117.2, 116.8, 116.5, 20.7, 17.3. HRMS (ESI) *m/z*: [M - CF_3SO_3]$^+$ Calcd for $C_{17}H_{19}N_4$ 279.1604; Found 279.1605.

4-(2-Aminophenyl)-1-(naphthalen-1-yl)-4*H*-1,2,4-triazol-1-ium tetrafluoroborate (5c)

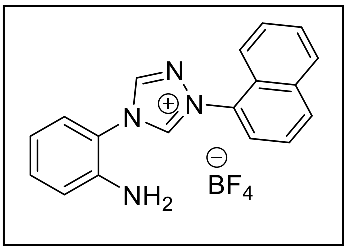

The title compound was prepared according to general procedure 3 on a 0.10 mmol scale. The product was obtained without further purification as a pale brown oil in 98% yield (36.7 mg, 0.0981 mmol). ^{1}H NMR (400 MHz, DMSO-d_6, 298 K): δ = 11.15 (s, 1H), 9.85 (s, 1H), 8.35 (d, *J* = 8.3 Hz, 1H), 8.25 – 8.18 (m, 1H), 8.18 – 8.11 (m, 1H), 8.03 (dd, *J* = 7.4, 1.2 Hz, 1H), 7.83 (dd, *J* = 8.3, 7.4 Hz, 1H), 7.80 – 7.72 (m, 2H), 7.55 (dd, *J* = 8.0, 1.6 Hz, 1H), 7.38 (ddd, *J* = 8.6, 7.3, 1.6 Hz, 1H), 7.00 (dd, *J* = 8.3, 1.4 Hz, 1H), 6.81 (ddd, *J* = 8.3, 7.3, 1.3 Hz, 1H), 5.93 (s, 2H); ^{13}C{^{1}H} NMR (100 MHz, DMSO-d_6, 298 K): δ = 145.9, 145.4, 144.1, 133.8, 132.1, 132.1, 128.5, 128.3, 127.9, 127.7, 127.0, 125.3, 125.2, 122.6, 116.8, 116.5, 116.5, 116.2. HRMS (ESI) *m/z*: [M - BF_4]$^+$ Calcd for $C_{18}H_{15}N_4$ 287.1291; Found 287.1294.

4-(2-Aminophenyl)-1-(4-methoxyphenyl)-4*H*-1,2,4-triazol-1-ium tetrafluoroborate (5d)

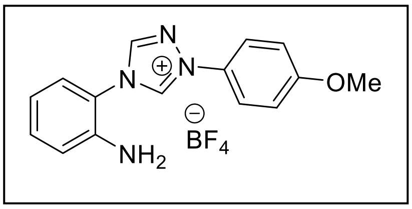

The title compound was prepared according to general procedure 3 on a 1.00 mmol scale. The product was obtained without further purification as a pale brown powder in 93% yield (330 mg, 0.931 mmol); m.p. 187-188 °C. ^{1}H NMR (400 MHz, DMSO-d_6, 298 K): δ = 11.12 (s, 1H), 9.64 (s, 1H), 7.92 (d, *J* = 8.7 Hz, 2H), 7.44 – 7.38 (m, 1H), 7.35 (t, *J* = 7.5 Hz, 1H), 7.28 (d, *J* = 8.7 Hz, 2H), 6.95 (d, *J* = 8.2 Hz, 1H), 6.76 (t, *J* = 7.6 Hz, 1H), 5.80 (s, 2H), 3.88 (s, 3H); ^{13}C{^{1}H} NMR (100 MHz, DMSO-d_6, 298 K): δ = 160.7, 145.5, 144.2, 141.8, 132.2, 128.2, 127.7, 122.5, 116.6, 116.4, 116.1, 115.3, 55.8. HRMS (ESI) *m/z*: [M - BF_4]$^+$ Calcd for $C_{15}H_{15}N_4O$ 267.1240; Found 267.1244.

4-(2-Aminophenyl)-1-(4-(trifluoromethyl)phenyl)-4*H*-1,2,4-triazol-1-ium trifluoromethanesulfonate (5e)

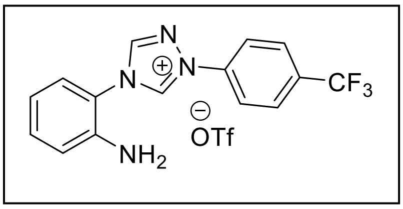

The title compound was prepared according to general procedure 3 on a 1.00 mmol scale. The product was obtained without further purification as a white powder in 98% yield (447 mg, 0.985 mmol); m.p. 152-154 °C.

^{1}H NMR (400 MHz, DMSO-d_6, 298 K): δ = 11.42 (s, 1H), 9.77 (s, 1H), 8.24 (d, J = 8.6 Hz, 2H), 8.18 (d, J = 8.6 Hz, 2H), 7.44 – 7.33 (m, 2H), 6.96 (dd, J = 8.2, 1.3 Hz, 1H), 6.77 (td, J = 7.6, 1.3 Hz, 1H), 5.82 (s, 2H); ^{13}C{^{1}H} NMR (100 MHz, DMSO-d_6, 298 K): δ 146.4, 144.6, 144.1, 138.4, 132.8, 131.0 (q, J = 32.6 Hz), 128.2 (q, J = 3.8 Hz), 128.0, 124.1 (q, J = 265.2 Hz), 121.9, 121.1 (q, J = 315.1 Hz, triflate), 117.1, 116.6, 116.5; ^{19}F{^{1}H} NMR (376 MHz, DMSO-d_6, 298 K, referenced to C_6H_5F): δ = -61.32, -77.86. HRMS (ESI) *m/z*: $[M - CF_3SO_3]^+$ Calcd for $C_{15}H_{12}F_3N_4$ 305.1009; Found 305.1005.

4-(2-Aminophenyl)-1-(4-chlorophenyl)-4*H*-1,2,4-triazol-1-ium trifluoromethanesulfonate (5f)

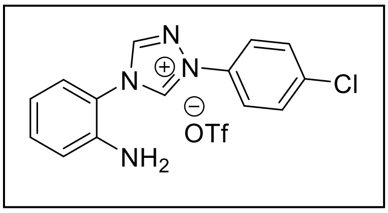

The title compound was prepared according to general procedure 3 on a 1.00 mmol scale. Purification by column chromatography (silica gel, 1. dichloromethane, 2. dichloromethane/methanol 17:3, R_f = 0.35) yielded the product as off-white powder in 29% yield (120 mg, 0.286 mmol); m.p. 184-185 °C.

^{1}H NMR (400 MHz, DMSO-d_6, 298 K): δ = 11.28 (s, 1H), 9.71 (s, 1H), 8.03 (d, J = 8.9 Hz, 2H), 7.85 (d, J = 8.9 Hz, 2H), 7.45 – 7.29 (m, 2H), 7.01 – 6.89 (m, 1H), 6.76 (t, J = 7.6 Hz, 1H), 5.80 (s, 2H); ^{13}C{^{1}H} NMR (100 MHz, DMSO-d_6, 298 K): δ = 145.8, 144.1, 142.9, 135.1, 133.8, 132.2, 130.4, 127.6, 122.5, 116.6, 116.2, 116.0. HRMS (ESI) *m/z*: $[M - CF_3SO_3]^+$ Calcd for $C_{14}H_{12}ClN_4$ 271.0745; Found 271.0741.

4-(2-Aminophenyl)-1-(4-fluorophenyl)-4*H*-1,2,4-triazol-1-ium trifluoromethanesulfonate (5g)

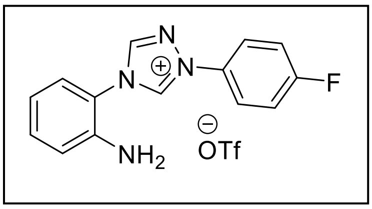

The title compound was prepared according to general procedure 3 on a 1.00 mmol scale. The product was obtained without further purification as an off-white powder in 97% yield (391 mg, 0.966 mmol); m.p. 184-185 °C.

^{1}H NMR (400 MHz, DMSO-d_6, 298 K): δ = ^{1}H NMR (400 MHz, DMSO) δ 11.22 (s, 1H), 9.70 (s, 1H), 8.12 – 7.98 (m, 2H), 7.63 (t, J = 8.8 Hz, 2H), 7.43 – 7.29 (m, 2H), 6.95 (dd, J = 8.2, 1.3 Hz, 1H), 6.81 – 6.70 (m, 1H), 5.81 (s, 2H); ^{13}C{^{1}H} NMR (100 MHz, DMSO-d_6, 298 K): δ 163.1 (d, J = 248.6 Hz), 146.1, 144.6, 143.2, 132.7, 132.0 (d, J = 2.9 Hz), 128.1, 123.8 (d, J = 9.2 Hz), 121.1 (q, J = 322.2 Hz), 117.8 (d, J = 23.8 Hz), 117.1, 116.7, 116.5; ^{19}F{^{1}H} NMR (376

MHz, DMSO-d_6, 298 K, referenced to C_6H_5F): δ = -77.85, -110.05. HRMS (ESI) *m/z*: $[M - CF_3SO_3]^+$ Calcd for $C_{14}H_{12}FN_4$ 255.1041; Found 255.1044.

4-(2-Aminophenyl)-1-(2-fluorophenyl)-4*H*-1,2,4-triazol-1-ium tetrafluoroborate (5h)

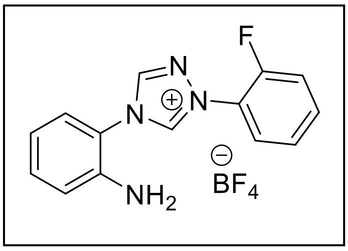

The title compound was prepared according to general procedure 3 on a 0.40 mmol scale. The product was obtained quantitatively without further purification as an off-white powder (137 mg, 0.40 mmol); m.p. 157-158 °C.

^{1}H NMR (400 MHz, DMSO-d_6, 298 K): δ = 11.12 (s, 1H), 9.77 (s, 1H), 7.99 (t, *J* = 7.6 Hz, 1H), 7.84 – 7.68 (m, 2H), 7.59 (t, *J* = 7.7 Hz, 1H), 7.43 (d, *J* = 8.0 Hz, 1H), 7.36 (t, *J* = 7.8 Hz, 1H), 6.95 (d, *J* = 8.2 Hz, 1H), 6.76 (t, *J* = 7.6 Hz, 1H), 5.81 (s, 2H); ^{13}C{^{1}H} NMR (100 MHz, DMSO-d_6, 298 K): δ = 153.8 (d, *J* = 254.3 Hz), 145.8, 145.1 (d, *J* = 6.2 Hz), 144.2, 133.2 (d, *J* = 7.9 Hz), 132.2, 127.7, 126.1 (d, *J* = 3.8 Hz), 125.8, 123.0 (d, *J* = 10.2 Hz), 117.7 (d, *J* = 18.8 Hz), 116.7, 116.3, 116.1; ^{19}F{^{1}H} NMR (376 MHz, DMSO-d_6, 298 K, referenced to C_6H_5F): δ = -122.18, -148.43. HRMS (ESI) *m/z*: $[M - BF_4]^+$ Calcd for $C_{14}H_{12}FN_4$ 255.1041; Found 255.1043.

2.6 1-Alkyl-1H-benzo[4,5]imidazo[2,1-c][1,2,4]triazoles(6a-6k)

1-Octyl-1*H*-benzo[4,5]imidazo[2,1-*c*][1,2,4]triazole (6a)

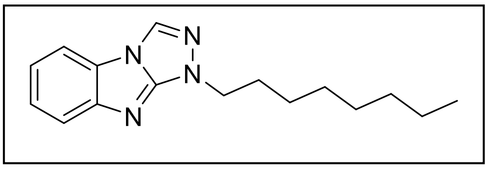

The title compound was prepared in two steps according to general procedure 6 from 4-(2-nitrophenyl)-1-octyl-4*H*-1,2,4-triazol-1-ium bromide (**2a**) on a 383 mg (1.00 mmol) scale. Purification by column chromatography (silica gel, hexanes/acetone 4:1, R_f = 0.16) yielded the product as an off-white powder in 75% yield (202 mg, 0.748 mmol); m.p. 93-94 °C.

^{1}H NMR (400 MHz, $CDCl_3$, 298 K): δ = 8.18 (s, 1H), 7.63 (d, *J* = 8.2 Hz, 1H), 7.54 (d, *J* = 8.0 Hz, 1H), 7.36 – 7.27 (m, 1H), 7.12 – 7.03 (m, 1H), 4.18 (t, *J* = 7.3 Hz, 2H), 1.91 (h, *J* = 7.1 Hz, 2H), 1.37 – 1.13 (m, 10H), 0.81 (t, *J* = 6.4 Hz, 3H); ^{13}C{^{1}H} NMR (100 MHz, $CDCl_3$, 298 K): δ = 153.9, 150.2, 126.4, 125.1, 124.9, 119.0, 118.6, 111.0, 48.3, 31.7, 29.1, 29.0, 28.7, 26.5, 22.6, 14.0. HRMS (ESI) *m/z*: $[M + H]^+$ Calcd for $C_{16}H_{23}N_4$ 271.1917; Found 271.1920.

1-Benzyl-1*H*-benzo[4,5]imidazo[2,1-*c*][1,2,4]triazole (6b)

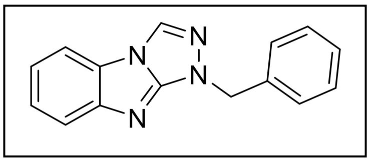

The title compound was prepared in two steps according to general procedure 6 from 1-benzyl-4-(2-nitrophenyl)-4*H*-1,2,4-triazol-1-ium bromide (**2b**) on a 1.445 g (4.00 mmol) scale. Purification by column chromatography (silica gel, hexanes/acetone 4:1, R_f = 0.11) yielded the product as an off-white powder in 70% yield (695 mg, 2.80 mmol); m.p. 168-169 °C.

^{1}H NMR (400 MHz, $CDCl_3$, 298 K) δ = 8.28 (s, 1H), 7.72 (d, *J* = 8.3 Hz, 1H), 7.64 (d, *J* = 8.0 Hz, 1H), 7.52 – 7.44 (m, 2H), 7.44 – 7.38 (m, 1H), 7.38 – 7.28 (m, 3H), 7.19 (ddd, *J* = 8.2, 7.4, 1.1 Hz, 1H), 5.45 (s, 2H); ^{13}C{^{1}H} NMR (100 MHz, $CDCl_3$, 298 K) δ = 153.6, 150.0, 135.1, 128.6, 128.1, 128.0, 126.7, 125.0, 124.8, 119.1, 118.6, 110.8, 51.6. HRMS (ESI) *m/z*: $[M + H]^+$ Calcd for $C_{15}H_{13}N_4$ 249.1135; Found 249.1139.

1-Isopropyl-1*H*-benzo[4,5]imidazo[2,1-*c*][1,2,4]triazole (6c)

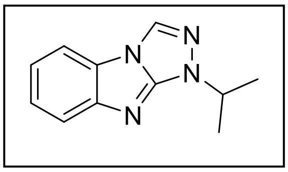

The title compound was prepared in two steps according to general procedure 6 from 1-isopropyl-4-(2-nitrophenyl)-4*H*-1,2,4-triazol-1-ium bromide (**2c**) on a 626 mg (2.00 mmol) scale. Purification by column chromatography (silica gel, hexanes/acetone 4:1, R_f = 0.14) yielded the product as an off-white powder in 41% yield (165 mg, 0.826 mmol); m.p. 82-83 °C.

^{1}H NMR (400 MHz, $CDCl_3$, 298 K) δ = 8.32 (s, 1H), δ 8.23 (s, 1H), 7.67 (dt, *J* = 8.3, 0.9 Hz, 1H), 7.60 (dt, *J* = 8.0, 1.0 Hz, 1H), 7.35 (ddd, *J* = 8.3, 7.3, 1.2 Hz, 1H), 7.13 (ddd, *J* = 8.2, 7.3, 1.1 Hz, 1H), 4.78 (sept, *J* = 6.7 Hz, 1H), 1.60 (d, *J* = 6.7 Hz, 6H); ^{13}C{^{1}H} NMR (100 MHz, $CDCl_3$, 298 K) δ = 153.4, 150.2, 126.4, 125.0, 124.8, 119.1, 118.7, 111.1, 51.2, 21.4. HRMS (ESI) *m/z*: $[M + H]^+$ Calcd for $C_{11}H_{13}N_4$ 201.1135; Found 201.1134.

1-(2-Ethoxyethyl)-1*H*-benzo[4,5]imidazo[2,1-*c*][1,2,4]triazole (6d)

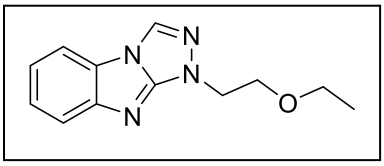

The title compound was prepared in two steps according to general procedure 6 from 1-(2-ethoxyethyl)-4-(2-nitrophenyl)-4*H*-1,2,4-triazol-1-ium bromide (**2d**) on a 343 mg (1.00 mmol) scale. Purification by column chromatography (silica gel, hexanes/acetone 4:1, R_f = 0.10) yielded the product as an off-white powder in 61% yield (140 mg, 0.610 mmol); m.p. 97-98 °C.

^{1}H NMR (400 MHz, $CDCl_3$, 298 K): *δ* = 8.23 (s, 1H), 7.64 (dt, *J* = 8.3, 0.9 Hz, 1H), 7.57 (dt, *J* = 8.0, 0.9 Hz, 1H), 7.33 (ddd, *J* = 8.4, 7.3, 1.2 Hz, 1H), 7.10 (ddd, *J* = 8.2, 7.4, 1.1 Hz, 1H), 4.39 (t, *J* = 5.7 Hz, 2H), 3.90 (t, *J* = 5.7 Hz, 2H), 3.49 (q, *J* = 7.0 Hz, 2H), 1.10 (t, *J* = 7.0 Hz, 3H); ^{13}C{^{1}H} NMR (100 MHz, $CDCl_3$, 298 K): *δ* = 154.0, 150.2, 126.8, 125.2, 124.9, 119.2, 118.7, 111.1, 67.2, 66.5, 48.0, 15.0. HRMS (ESI) *m/z*: $[M + H]^+$ Calcd for $C_{12}H_{15}N_4O$ 231.1240; Found 231.1243.

6-(1*H*-Benzo[4,5]imidazo[2,1-*c*][1,2,4]triazol-1-yl)hexan-1-ol (6e)

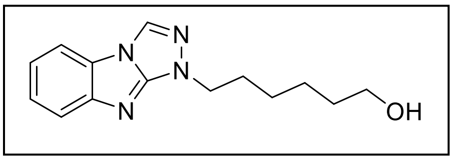

The title compound was prepared in two steps according to general procedure 6 from 1-(6-hydroxyhexyl)-4-(2-nitrophenyl)-4*H*-1,2,4-triazol-1-ium bromide (**2e**) on a 371 mg (1.00 mmol) scale. Purification by column chromatography

(silica gel, hexanes/acetone 3:2, R_f = 0.20) yielded the product as an off-white powder in 89% yield (202 mg, 0.748 mmol); m.p. 67-68 °C.

^{1}H NMR (400 MHz, $CDCl_3$, 298 K): δ = 8.26 (s, 1H), 7.67 (d, *J* = 8.3 Hz, 1H), 7.63 (d, *J* = 8.0, 1H), 7.37 (ddd, *J* = 8.3, 7.3, 1.2 Hz, 1H), 7.16 (ddd, *J* = 8.0, 7.3, 1.0 Hz, 1H), 4.27 (t, *J* = 6.9 Hz, 2H), 3.60 (t, *J* = 6.2 Hz, 2H), 2.43 (s, 1H), 1.98 (p, *J* = 7.0 Hz, 2H), 1.62 – 1.33 (m, 6H); ^{13}C{^{1}H} NMR (100 MHz, $CDCl_3$, 298 K): δ =154.0, 150.0, 126.7, 125.2, 125.1, 119.4, 118.8, 111.2, 62.1, 48.1, 32.4, 28.9, 25.8, 24.8. HRMS (ESI) *m/z*: $[M + H]^+$ Calcd for $C_{14}H_{19}N_4O$ 259.1553; Found 259.1556.

1-(But-3-yn-1-yl)-1*H*-benzo[4,5]imidazo[2,1-*c*][1,2,4]triazole (6f)

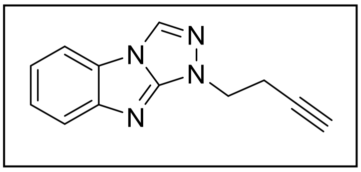

The title compound was prepared in two steps according to general procedure 6 from 1-(but-3-yn-1-yl)-4-(2-nitrophenyl)-4*H*-1,2,4-triazol-1-ium bromide (**2f**) on a 190 mg (0.588 mmol) scale. Purification by column chromatography (silica gel, hexanes/acetone 4:1, R_f = 0.13) yielded the product as an off-white powder in 76% yield (94.4 mg, 0.449 mmol); m.p. 132-133 °C.

^{1}H NMR (400 MHz, $CDCl_3$, 298 K) δ = 8.30 (s, 1H), 7.70 (d, *J* = 8.2 Hz, 1H), 7.66 (d, *J* = 8.0 Hz, 1H), 7.43-7.38 (m, 1H), 7.22-7.18 (m, 1H), 4.47 (t, *J* = 7.0 Hz, 2H), 2.89 (td, *J* = 7.0, 2.7 Hz, 2H), 1.99 (t, *J* = 2.7 Hz, 1H); ^{13}C{^{1}H} NMR (100 MHz, $CDCl_3$, 298 K) δ = 153.8, 150.3, 127.1, 125.3, 125.2, 119.5, 119.0, 111.2, 79.8, 70.9, 46.9, 19.0. HRMS (ESI) *m/z*: $[M + H]^+$ Calcd for $C_{12}H_{11}N_4$ 211.0978; Found 211.0982.

1-Allyl-1*H*-benzo[4,5]imidazo[2,1-*c*][1,2,4]triazole (6g)

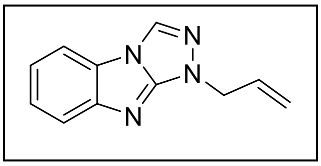

The title compound was prepared in two steps according to general procedure 6 from 1-allyl-4-(2-nitrophenyl)-4*H*-1,2,4-triazol-1-ium bromide (**2g**) on a 311 mg (1.00 mmol) scale. Purification by column chromatography (silica gel, hexanes/acetone 4:1, R_f = 0.15) yielded the product as an off-white powder in 61% yield (122 mg, 0.613 mmol); m.p. 112-113 °C.

^{1}H NMR (400 MHz, $CDCl_3$, 298 K): *δ* = 8.27 (s, 1H), 7.68 (dt, *J* = 8.2, 1.0 Hz, 1H), 7.63 (dt, *J* = 8.0, 1.0 Hz, 1H), 7.39-7.35 (m, 1H), 7.18-7.14 (m, 1H), 6.07 (ddt, *J* = 17.1, 10.2, 5.9 Hz, 1H), 5.40 – 5.30 (m, 2H), 4.86 (dt, *J* = 5.9, 1.5 Hz, 2H); ^{13}C{^{1}H} NMR (100 MHz, $CDCl_3$, 298 K): *δ* = 153.8, 150.2, 131.2, 127.0, 125.3, 125.2, 119.5, 119.5, 119.0, 111.2, 50.6. HRMS (ESI) *m/z*: $[M + H]^+$ Calcd for $C_{11}H_{11}N_4$ 199.0978; Found 199.0981.

1-(2-Methylallyl)-1*H*-benzo[4,5]imidazo[2,1-*c*][1,2,4]triazole (6h)

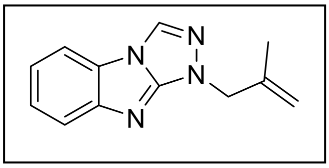

The title compound was prepared in two steps according to general procedure 6 from 1-(2-methylallyl)-4-(2-nitrophenyl)-4*H*-1,2,4-triazol-1-ium bromide (**2h**) on a 325 mg (1.00 mmol) scale. Purification by column chromatography (silica gel, hexanes/acetone 4:1, R_f = 0.18) yielded the product as an off-white powder in 65% yield (138 mg, 0.649 mmol); m.p. 135-136 °C.

^{1}H NMR (400 MHz, $CDCl_3$, 298 K) δ = 8.32 (s, 1H), 7.71 (d, *J* = 8.2 Hz, 1H), 7.66 (d, *J* = 8.0 Hz, 1H), 7.43-7.38 (m, 1H), 7.22-7.18 (m, 1H), 5.08-4.94 (m, 2H), 4.84 (s, 2H), 1.79 (s, 3H); ^{13}C{^{1}H} NMR (100 MHz, $CDCl_3$, 298 K): δ = 154.0, 150.3, 139.2, 126.9, 125.2, 125.0, 119.3, 118.9, 114.4, 111.1, 53.8, 20.0. HRMS (ESI) *m/z*: $[M + H]^+$ Calcd for $C_{12}H_{13}N_4$ 213.1135; Found 213.1139.

2-(1*H*-Benzo[4,5]imidazo[2,1-*c*][1,2,4]triazol-1-yl)-1-morpholinoethan-1-one (6i)

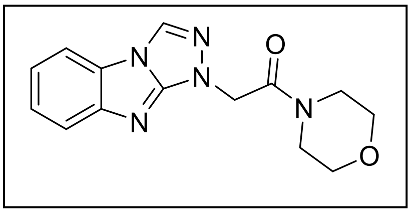

The title compound was prepared in two steps according to general procedure 6 from 1-(2-morpholino-2-oxoethyl)-4-(2-nitrophenyl)-4*H*-1,2,4-triazol-1-ium chloride (**2i**) on a 354 mg (1.00 mmol) scale. Purification by column chromatography (silica gel, hexanes/acetone 3:2, R_f = 0.08), followed by removal of DBU under high vacuum yielded the product as a white powder in 43% yield (121 mg, 0.425 mmol); m.p. 185-186 °C.

^{1}H NMR (400 MHz, $CDCl_3$, 298 K): δ = 8.36 (s, 1H), 7.67-7.64 (m, 2H), 7.39 (ddd, *J* = 8.0, 7.6, 1.2 Hz, 1H), 7.20 (td, *J* = 7.7, 1.0 Hz, 1H), 5.18 (s, 2H), 3.82 – 3.48 (m, 8H); ^{13}C{^{1}H} NMR (100 MHz, $CDCl_3$, 298 K): δ = 164.1, 154.5, 149.8, 128.0, 125.6, 125.2, 119.9, 119.0, 111.3, 66.8, 66.3, 49.2, 45.3, 42.5. HRMS (ESI) *m/z*: $[M + H]^+$ Calcd for $C_{14}H_{16}N_5O_2$ 286.1299; Found 286.1303.

Ethyl 2-(1*H*-benzo[4,5]imidazo[2,1-*c*][1,2,4]triazol-1-yl)acetate (6j)

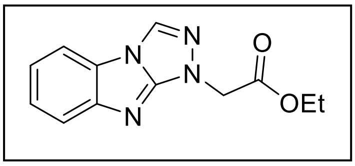

The title compound was prepared in two steps according to general procedure 6 from 1-(2-ethoxy-2-oxoethyl)-4-(2-nitrophenyl)-4*H*-1,2,4-triazol-1-ium bromide (**2j**) on a 357 mg (1.00 mmol) scale. Purification by column chromatography (silica gel, 1. Hexanes/acetone 4:1, 2. hexanes/acetone 3:2, R_f = 0.40) yielded the product as an off-white powder in 63% yield (155 mg, 0.7635 mmol); m.p. 167-168 °C.

^{1}H NMR (400 MHz, $CDCl_3$, 298 K): δ = ^{1}H NMR (400 MHz, $CDCl_3$) δ 8.32 (s, 1H), 7.67 (t, *J* = 8.2 Hz, 2H), 7.39 (ddd, *J* = 8.2, 7.4, 1.2 Hz, 1H), 7.20 (ddd, *J* = 8.2, 7.4, 1.0 Hz, 1H), 5.03 (s, 2H), 4.25 (q, *J* = 7.1 Hz, 2H), 1.28 (t, *J* = 7.1 Hz, 3H); ^{13}C{^{1}H} NMR (100 MHz, $CDCl_3$, 298 K): δ = 167.0, 154.2, 150.2, 127.8, 125.6, 125.1, 119.8, 119.1, 111.2, 62.2, 48.9, 14.2. HRMS (ESI) *m/z*: $[M + H]^+$ Calcd for $C_{12}H_{13}N_4O_2$ 245.1033; Found 245.1037.

2-(1*H*-Benzo[4,5]imidazo[2,1-*c*][1,2,4]triazol-1-yl)-1-phenylethan-1-one (6k)

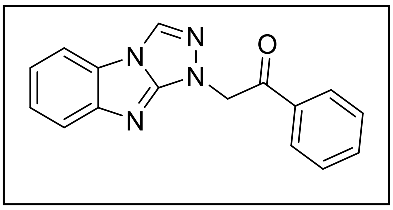

The title compound could not be prepared in two steps according to general procedure 6 from 4-(2-Nitrophenyl)-1-(2-oxo-2-phenylethyl)-4*H*-1,2,4-triazol-1-ium bromide (**2k**). HRMS indicated that it was present in trace amounts in a complex mixture. HRMS (ESI) *m/z*: $[M + H]^+$ Calcd for $C_{16}H_{13}N_4O$ 277.1084; Found, 277.1087.

2.7. 1-Aryl-1H-benzo[4,5]imidazo[2,1-c][1,2,4]triazoles (7a-7h)

1-Phenyl-1*H*-benzo[4,5]imidazo[2,1-*c*][1,2,4]triazole (7a)

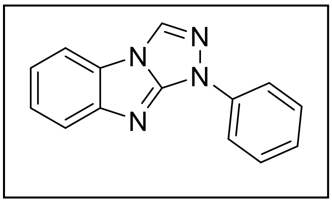

The title compound was prepared in two steps according to general procedure 6 from 4-(2-nitrophenyl)-1-phenyl-4*H*-1,2,4-triazol-1-ium tetrafluoroborate (**3a**) on a 354 mg (1.00 mmol) scale. Purification by column chromatography (silica gel, hexanes/acetone 4:1, R_f = 0.23) yielded the product as an off-white powder in 65% yield (152 mg, 0.648 mmol); m.p. 188-189 °C.

^{1}H NMR (400 MHz, $CDCl_3$, 298 K) δ = 8.45 (s, 1H), 8.33-8.25 (m, 2H), 7.85 (dt, *J* = 8.2, 0.9 Hz, 1H), 7.72 (dt, *J* = 8.0, 0.9 Hz, 1H), 7.62-7.54 (m, 2H), 7.47 (ddd, *J* = 8.3, 7.3, 1.2 Hz, 1H), 7.36-7.25 (m, 2H); ^{13}C{^{1}H} NMR (100 MHz, $CDCl_3$, 298 K) δ = 151.5, 150.0, 137.7, 129.6, 127.6, 125.7, 125.4, 124.6, 120.4, 119.6, 117.5, 111.1. HRMS (ESI) *m/z*: $[M + H]^+$ Calcd for $C_{14}H_{11}N_4$ 235.0978; Found 235.0981.

1-Mesityl-1*H*-benzo[4,5]imidazo[2,1-*c*][1,2,4]triazole (7b)

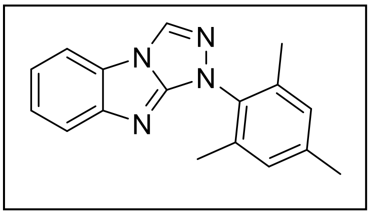

The title compound was prepared in two steps according to general procedure 6 from 1-mesityl-4-(2-nitrophenyl)-4*H*-1,2,4-triazol-1-ium trifluoromethanesulfonate (**3b**) on a 430 mg (0.94 mmol) scale. Purification by column chromatography (silica gel, hexanes/acetone 9:1, R_f = 0.22) yielded the product as an off-white powder in 68% yield (177 mg, 0.642 mmol); m.p. 161-162 °C.

^{1}H NMR (400 MHz, $CDCl_3$, 298 K) δ = 8.49 (s, 1H), 7.71 (m, 2H), 7.41 (t, *J* = 7.9 Hz, 1H), 7.23 (t, *J* = 7.6 Hz, 1H), 7.00 (s, 2H), 2.35 (s, 3H), 2.11 (s, 6H); ^{13}C{^{1}H} NMR (100 MHz, $CDCl_3$, 298 K) δ = 153.5, 150.6, 140.1, 136.8, 131.5, 129.5, 127.7, 125.4, 125.1, 119.5, 119.3, 111.2, 21.3, 17.9. HRMS (ESI) *m/z*: $[M + H]^+$ Calcd for $C_{17}H_{17}N_4$ 277.1448; Found 277.1452.

1-(Naphthalen-1-yl)-1*H*-benzo[4,5]imidazo[2,1-*c*][1,2,4]triazole (7c)

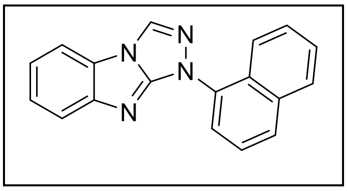

The title compound was prepared in two steps according to general procedure 6 from 1-(naphthalen-1-yl)-4-(2-nitrophenyl)-4*H*-1,2,4-triazol-1-ium tetrafluoroborate (**3c**) on a 67 mg (0.165 mmol) scale. Purification by column chromatography (silica gel, hexanes/acetone 9:1, R_f = 0.15) yielded the product as an off-white powder in 87% yield (40.8 mg, 0.143 mmol); m.p. 176-178 °C.

^{1}H NMR (800 MHz, $CDCl_3$, 298 K): δ = 8.63 (s, 1H), 8.09 (d, J = 7.8 Hz, 1H), 8.01-7.95 (m, 2H), 7.92 (d, J = 7.2 Hz, 1H), 7.77 (dd, J = 8.2, 4.0 Hz, 2H), 7.63 (t, J = 7.8 Hz, 1H), 7.60-7.55 (m, 2H), 7.45 (t, J = 7.7 Hz, 1H), 7.29 (t, J = 7.7 Hz, 1H); ^{13}C{^{1}H} NMR (200 MHz, $CDCl_3$, 298 K): δ = 152.9, 148.8, 134.8, 132.5, 129.9, 128.7, 128.4, 127.4, 127.0, 125.8, 125.6, 124.9, 123.5, 123.2, 120.7, 119.3, 111.4. HRMS (ESI) *m/z*: $[M + H]^+$ Calcd for $C_{18}H_{13}N_4$ 285.1135; Found 285.1134.

1-(4-Methoxyphenyl)-1*H*-benzo[4,5]imidazo[2,1-*c*][1,2,4]triazole (7d)

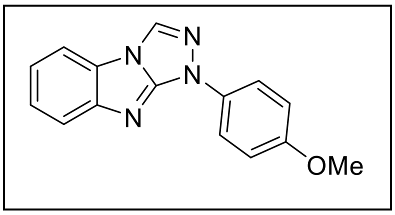

The title compound was prepared in two steps according to general procedure 6 from 1-(4-methoxyphenyl)-4-(2-nitrophenyl)-4*H*-1,2,4-triazol-1-ium tetrafluoroborate (**3d**) on a 384 mg (1.00 mmol) scale. Purification by column chromatography (silica gel, hexanes/acetone 4:1, R_f = 0.16) yielded the product as an off-white powder in 71% yield (188 mg, 0.710 mmol); m.p. 160-161 °C.

^{1}H NMR (400 MHz, $CDCl_3$, 298 K) δ = 8.38 (s, 1H), 8.11 (d, J = 9.1 Hz, 2H), 7.78 (dt, J = 8.2, 0.9 Hz, 1H), 7.67 (dt, J = 8.1, 0.9 Hz, 1H), 7.41 (ddd, J = 8.3, 7.4, 1.2 Hz, 1H), 7.21 (ddd, J = 8.3, 7.3, 1.1 Hz, 1H), 7.05 (d, J = 9.1 Hz, 2H), 3.85 (s, 3H); ^{13}C{^{1}H} NMR (100 MHz, $CDCl_3$, 298 K) δ = 157.5, 151.5, 150.1, 131.3, 127.2, 125.3, 124.7, 120.1, 119.4, 119.1, 114.7, 111.1, 55.7. HRMS (ESI) *m/z*: $[M + H]^+$ Calcd for $C_{15}H_{13}N_4O$ 265.1084; Found 265.1089.

1-(4-(Trifluoromethyl)phenyl)-1*H*-benzo[4,5]imidazo[2,1-*c*][1,2,4]triazole (7e)

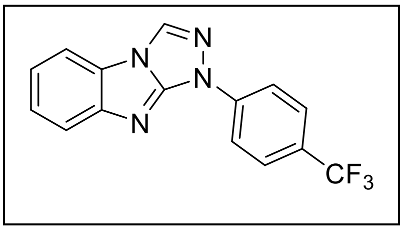

The title compound was prepared in two steps according to general procedure 6 from 4-(2-nitrophenyl)-1-(4-(trifluoromethyl)-phenyl)-4*H*-1,2,4-triazol-1-ium trifluoromethanesulfonate (**3e**) on a 484 mg (1.00 mmol) scale. Purification by column chromatography (silica gel, hexanes/acetone 4:1, R_f = 0.30) yielded the product as an off-white powder in 84% yield (253 mg, 0.837 mmol); m.p. 223-224 °C.

^{1}H NMR (400 MHz, $CDCl_3$, 298 K): δ = 11.53 (s, 1H), 9.96 (s, 1H), 8.55 (dd, J = 8.2, 1.4 Hz, 1H), 8.27 (d, J = 8.6 Hz, 2H), 8.22-8.16 (m, 3H), 8.11-8.04 (m, 2H); ^{13}C{^{1}H} NMR (100 MHz, $CDCl_3$, 298 K): δ 151.3, 149.8, 140.2, 128.4, 127.4 (q, J = 32.9 Hz), 126.9 (q, J = 3.8 Hz), 125.7, 124.7, 124.1 (q, J =

271.7 Hz), 121.0, 119.8, 117.3, 111.2.; $^{19}F\{^1H\}$ NMR (376 MHz, $CDCl_3$, 298 K, referenced to C_6H_5F): δ = -62.21. HRMS (ESI) *m/z*: $[M + H]^+$ Calcd for $C_{15}H_{10}F_3N_4$ 303.0852; Found 303.0850.

1-(4-Chlorophenyl)-1*H*-benzo[4,5]imidazo[2,1-*c*][1,2,4]triazole (7f)

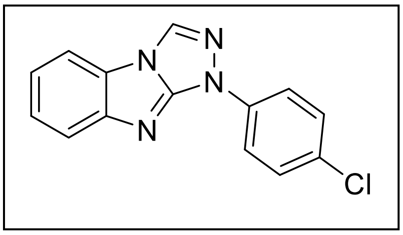

The title compound was prepared in two steps according to general procedure 6 from 1-(4-chlorophenyl)-4-(2-nitrophenyl)-4*H*-1,2,4-triazol-1-ium trifluoromethanesulfonate (**3f**) on a 451 mg (1.00 mmol) scale. Purification by column chromatography (silica gel, hexanes/acetone 17:3, R_f = 0.25) yielded the product as an off-white powder in 58% yield (156 mg, 0.584 mmol); m.p. 223-229 °C.

^1H NMR (400 MHz, $CDCl_3$, 298 K) δ = 8.43 (s, 1H), 8.20 (d, *J* = 8.7 Hz, 2H), 7.80 (d, *J* = 8.2 Hz, 1H), 7.69 (d, *J* = 7.9 Hz, 1H), 7.50 (d, *J* = 8.7 Hz, 2H), 7.44 (t, *J* = 7.8 Hz, 1H), 7.30-7.22 (m, 1H).$^{13}C\{^1H\}$ NMR (100 MHz, $CDCl_3$, 298 K) δ = 151.1, 149.7, 136.2, 131.0, 129.7, 127.9, 125.6, 124.6, 120.8, 119.6, 118.7, 111.2. HRMS (ESI) *m/z*: $[M + H]^+$ Calcd for $C_{14}H_{10}ClN_4$ 269.0589; Found 269.0590.

1-(4-Fluorophenyl)-1*H*-benzo[4,5]imidazo[2,1-*c*][1,2,4]triazole (7g)

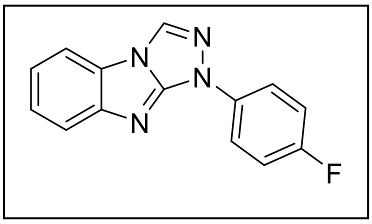

The title compound was prepared in two steps according to general procedure 6 from 1-(4-fluorophenyl)-4-(2-nitrophenyl)-4*H*-1,2,4-triazol-1-ium trifluoromethanesulfonate (**3g**) on a 434 mg (1.00 mmol) scale. Purification by column chromatography (silica gel, hexanes/acetone 9:1, R_f = 0.20) yielded the product as an off-white powder in 67% yield (170 mg, 0.674 mmol); m.p. 187-188 °C.

^1H NMR (400 MHz, $CDCl_3$, 298 K) δ = 8.43 (s, 1H), 8.26-8.17 (m, 2H), 7.80 (d, *J* = 8.2 Hz, 1H), 7.70 (d, *J* = 8.0 Hz, 1H), 7.47-7.41 (m, 1H), 7.28-7.19 (m, 3H); $^{13}C\{^1H\}$ NMR (100 MHz, $CDCl_3$, 298 K) δ = 160.5 (d, *J* = 245.2 Hz), 127.7, 119.3 (d, *J* = 8.2 Hz), 116.4 (d, *J* = 23.1 Hz), 111.2; $^{19}F\{^1H\}$ NMR (376 MHz, $CDCl_3$, 298 K, referenced to C_6H_5F): δ = -116.56. HRMS (ESI) *m/z*: $[M + H]^+$ Calcd for $C_{14}H_{10}FN_4$ 253.0884; Found 253.0884.

1-(2-Fluorophenyl)-1*H*-benzo[4,5]imidazo[2,1-*c*][1,2,4]triazole (7h)

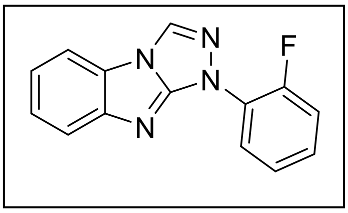

The title compound was prepared in two steps according to general procedure 6 from 1-(2-fluorophenyl)-4-(2-nitrophenyl)-4*H*-1,2,4-triazol-1-ium tetrafluoroborate (**3h**) on a 372 mg (1.00 mmol) scale. Purification by column chromatography (silica gel, hexanes/acetone 4:1, R_f = 0.15) yielded the product as an off-white powder in 86% yield (216 mg, 0.855 mmol); m.p. 151-152 °C.

^1H NMR (400 MHz, $CDCl_3$, 298 K): δ = ^1H NMR (400 MHz, DMSO) 8.48 (s, 1H), 7.92- 7.83 (m, 1H), 7.74 (d, *J* = 8.2 Hz, 1H), 7.69 (d, *J* = 8.0 Hz, 1H), 7.44-7.35 (m, 2H), 7.31 (t, *J* = 8.6 Hz, 2H), 7.23 (t, *J*

= 7.5 Hz, 1H)); $^{13}C\{^1H\}$ NMR (100 MHz, $CDCl_3$, 298 K): δ 155.8 (d, *J* = 254.1 Hz), 153.0, 150.4, 129.8 (d, *J* = 7.6 Hz), 128.9, 125.9, 125.7, 125.5, 125.3 (d, *J* = 3.9 Hz), 125.2 (d, *J* = 10.9 Hz), 120.6, 119.9, 117.8 (d, *J* = 19.1 Hz), 111.5; $^{19}F\{^1H\}$ NMR (376 MHz, $CDCl_3$, 298 K, referenced to C_6H_5F): δ = -120.7. HRMS (ESI) *m/z*: $[M + H]^+$ Calcd for $C_{14}H_{10}FN_4$ 253.0884; Found 253.0884.

3. NMR Spectra of Isolated Compounds

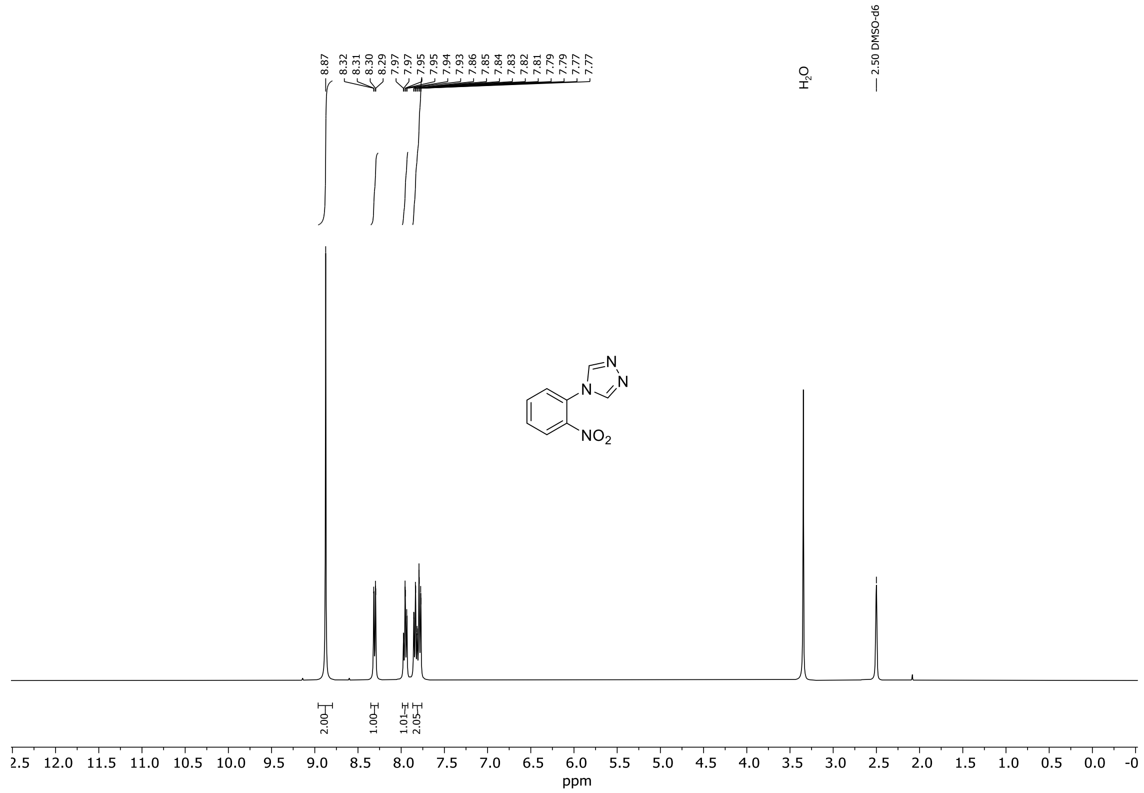

Figure S01: ^{1}H NMR spectrum of 4-(2-nitrophenyl)-4*H*-1,2,4-triazole (**1**) (400 MHz, DMSO-d_6, 298 K).

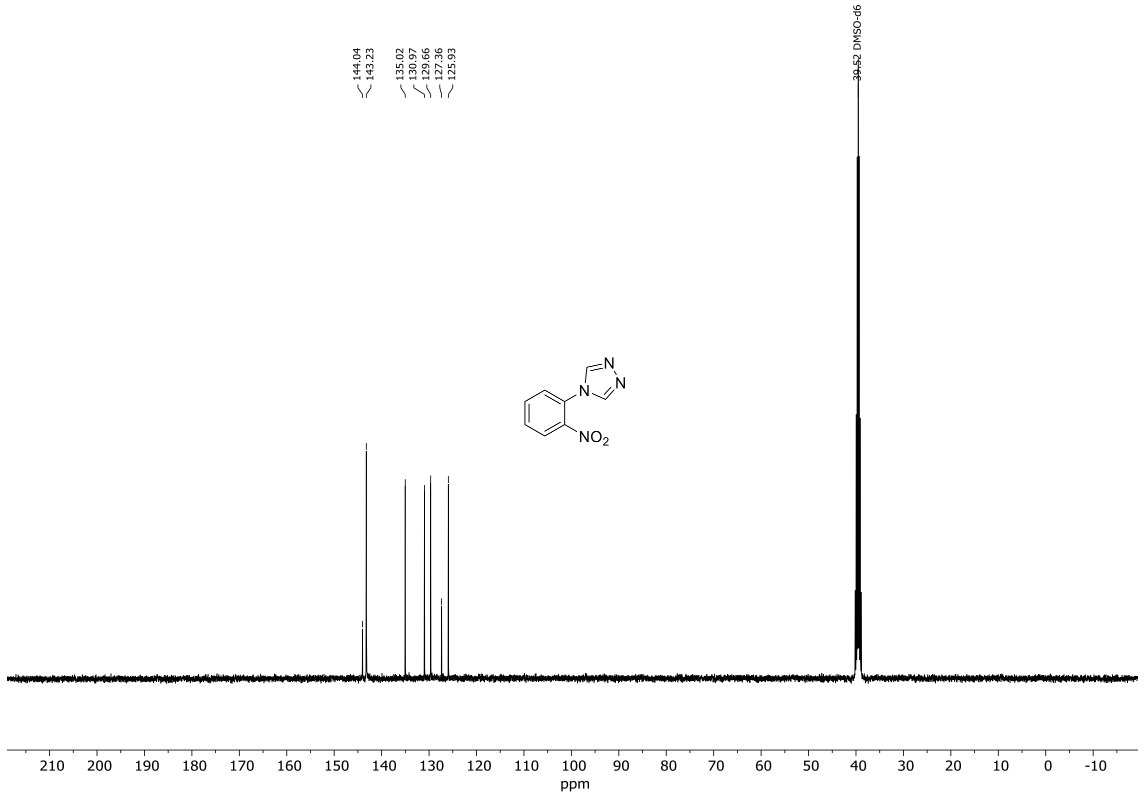

Figure S02: ^{13}C{^{1}H} NMR spectrum of 4-(2-nitrophenyl)-4*H*-1,2,4-triazole (**1**) (100 MHz, DMSO-d_6, 298 K).

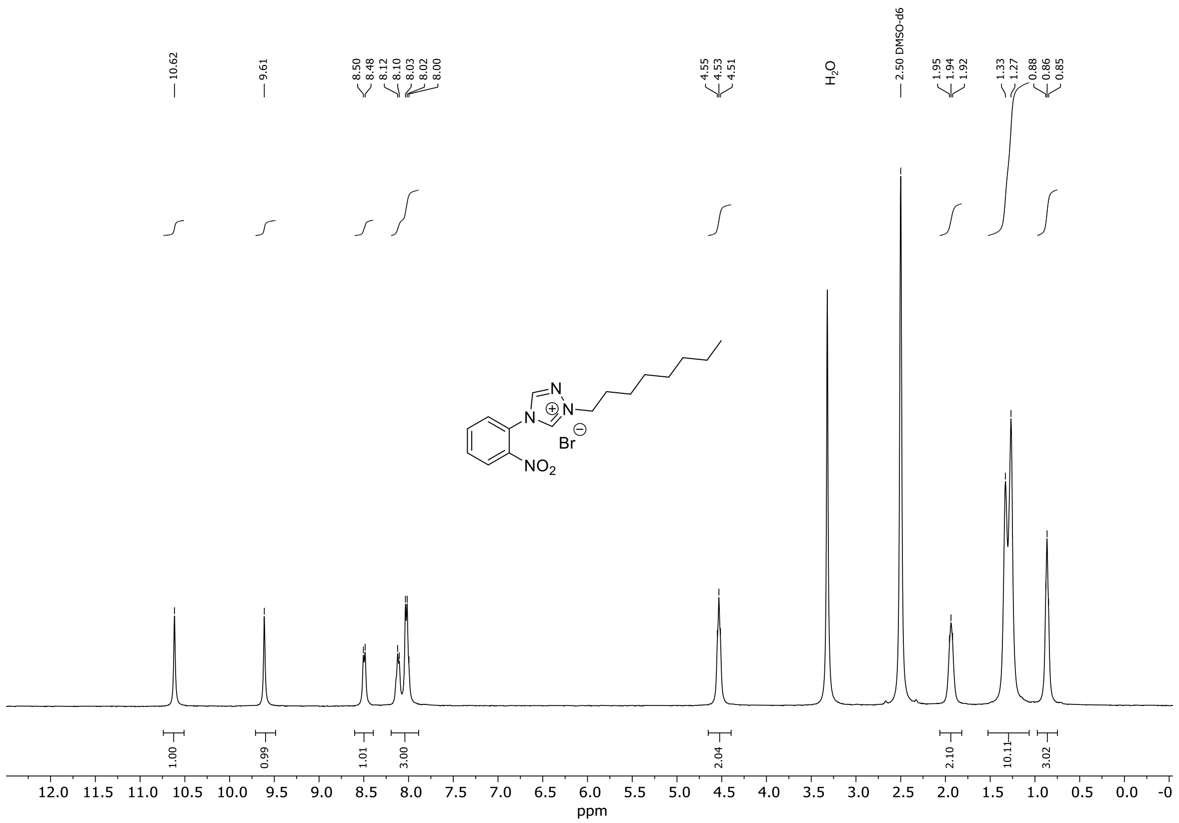

Figure S03: ^{1}H NMR spectrum of 4-(2-nitrophenyl)-1-octyl-4*H*-1,2,4-triazol-1-ium bromide (**2a**) (400 MHz, DMSO-d_6, 298 K).

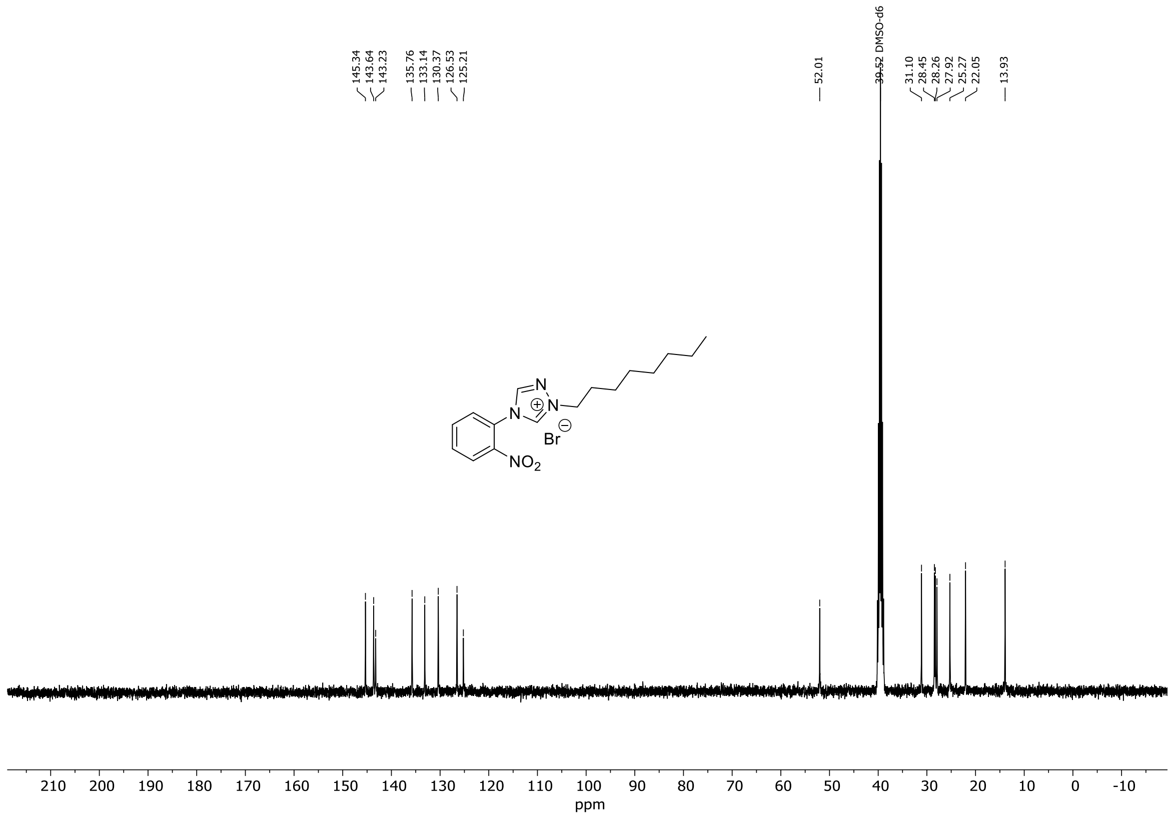

Figure S04: $^{13}C\{^{1}H\}$ NMR spectrum of 4-(2-nitrophenyl)-1-octyl-4*H*-1,2,4-triazol-1-ium bromide (**2a**) (100 MHz, DMSO-d_6, 298 K).

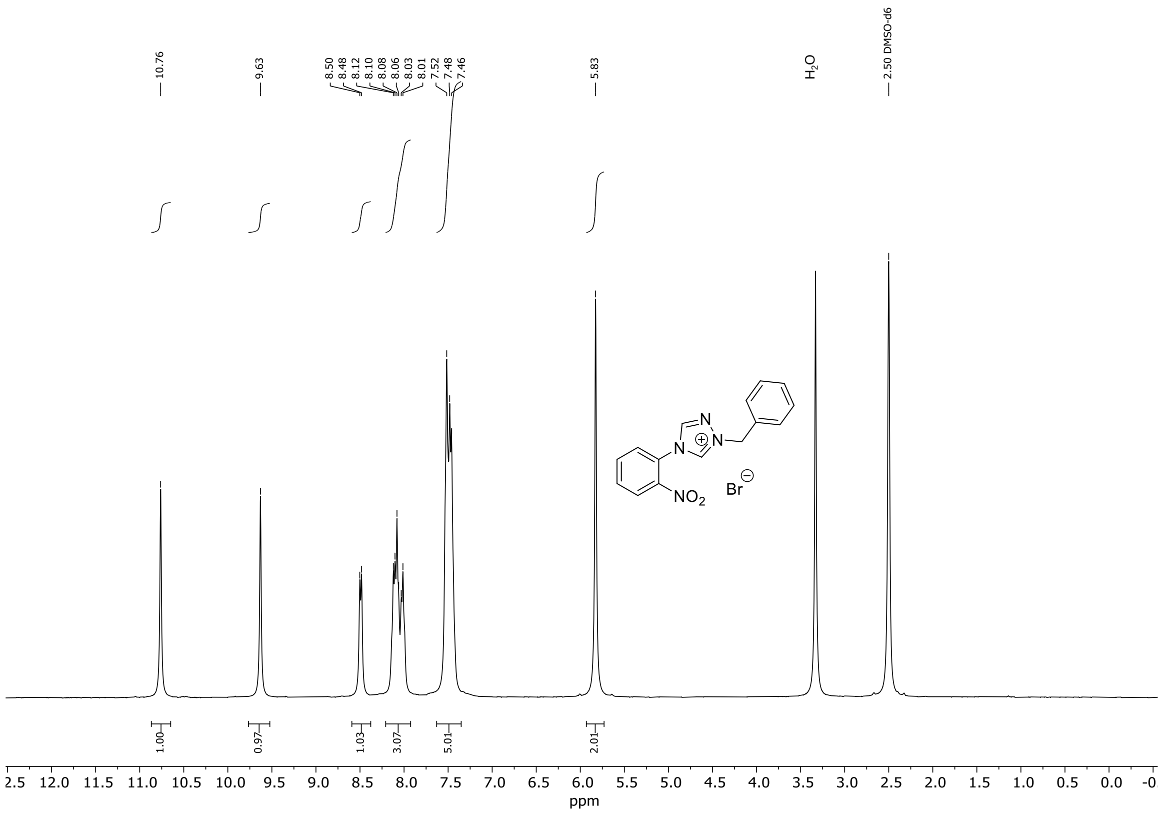

Figure S05: ^{1}H NMR spectrum of 1-benzyl-4-(2-nitrophenyl)-4*H*-1,2,4-triazol-1-ium bromide (**2b**) (400 MHz, DMSO-d_6, 298 K).

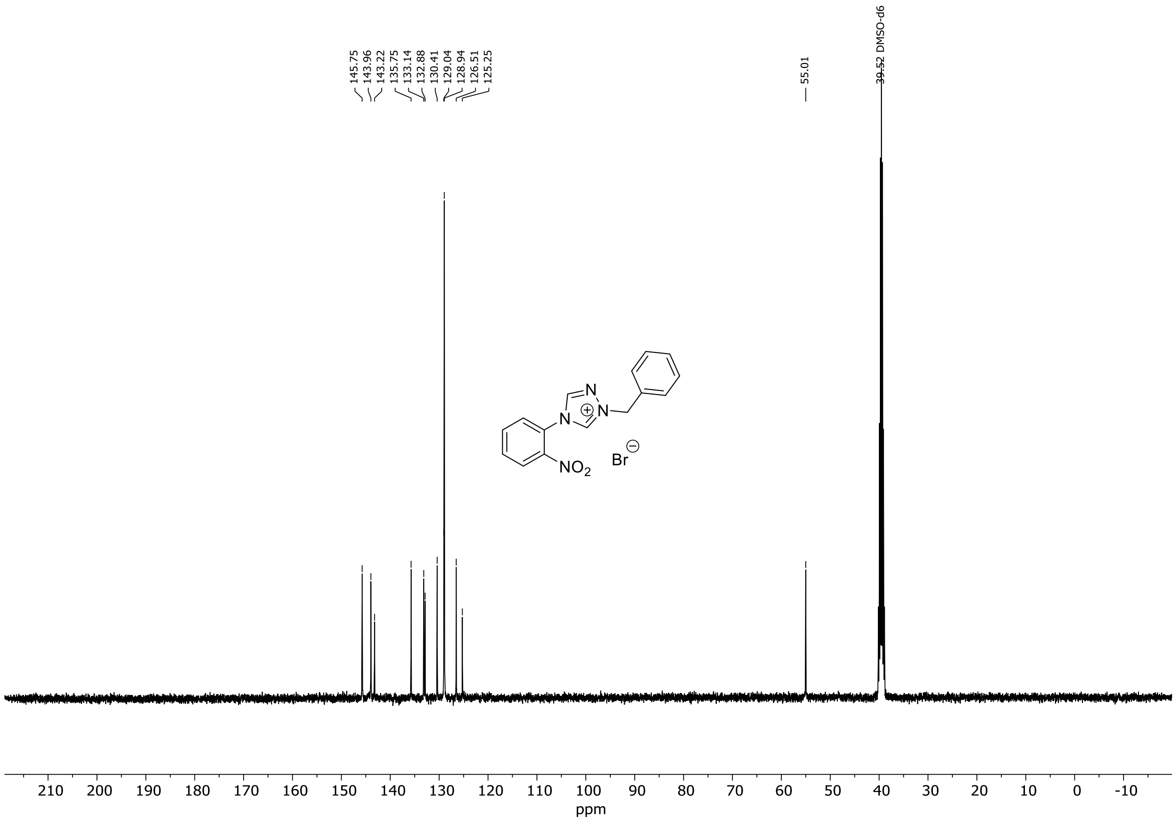

Figure S06: $^{13}C\{^{1}H\}$ NMR spectrum of 1-benzyl-4-(2-nitrophenyl)-4*H*-1,2,4-triazol-1-ium bromide (**2b**) (100 MHz, DMSO-d_6, 298 K).

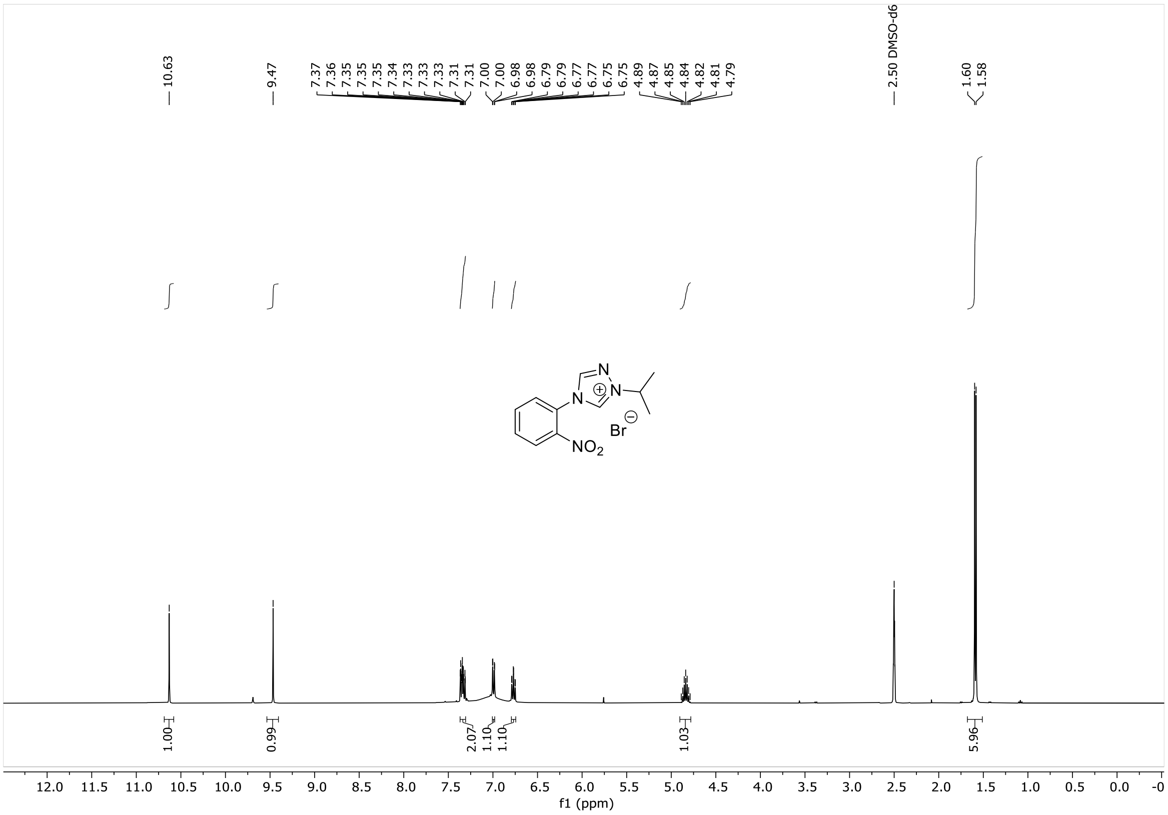

Figure S07: ^{1}H NMR spectrum of 1-isopropyl-4-(2-nitrophenyl)-4*H*-1,2,4-triazol-1-ium bromide (**2c**) (400 MHz, DMSO-d_6, 298 K).

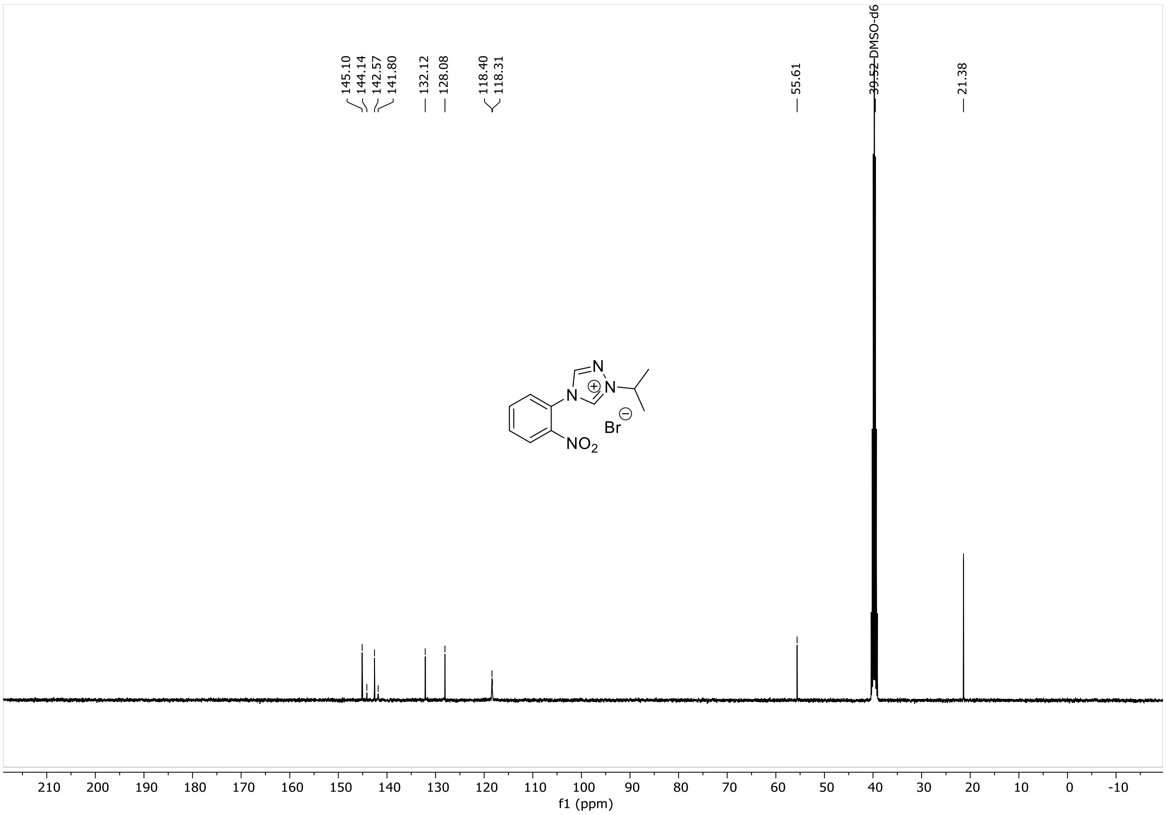

Figure S08: ^{13}C{^{1}H} NMR of 1-isopropyl-4-(2-nitrophenyl)-4*H*-1,2,4-triazol-1-ium bromide (**2c**) (100 MHz, DMSO-d_6, 298 K).

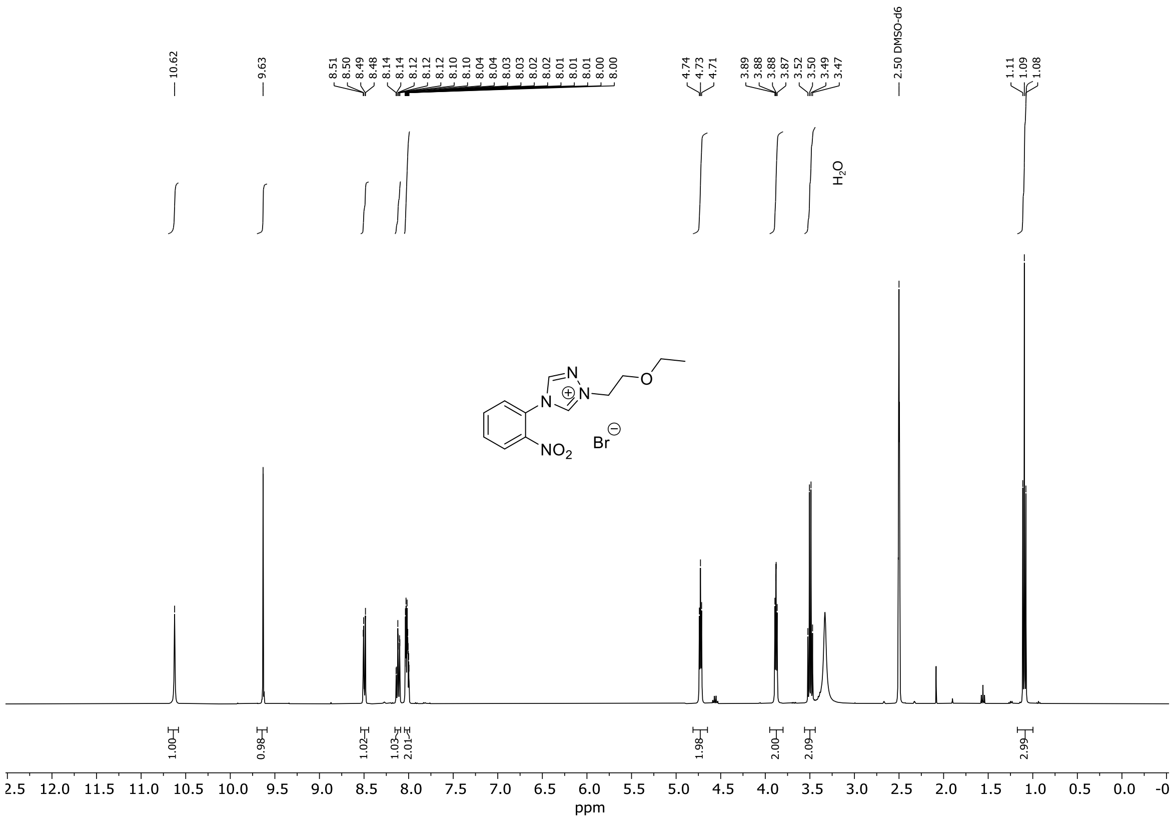

Figure S09: ^{1}H NMR spectrum of 1-(2-ethoxyethyl)-4-(2-nitrophenyl)-4*H*-1,2,4-triazol-1-ium bromide (**2d**) (400 MHz, DMSO-d_6, 298 K).

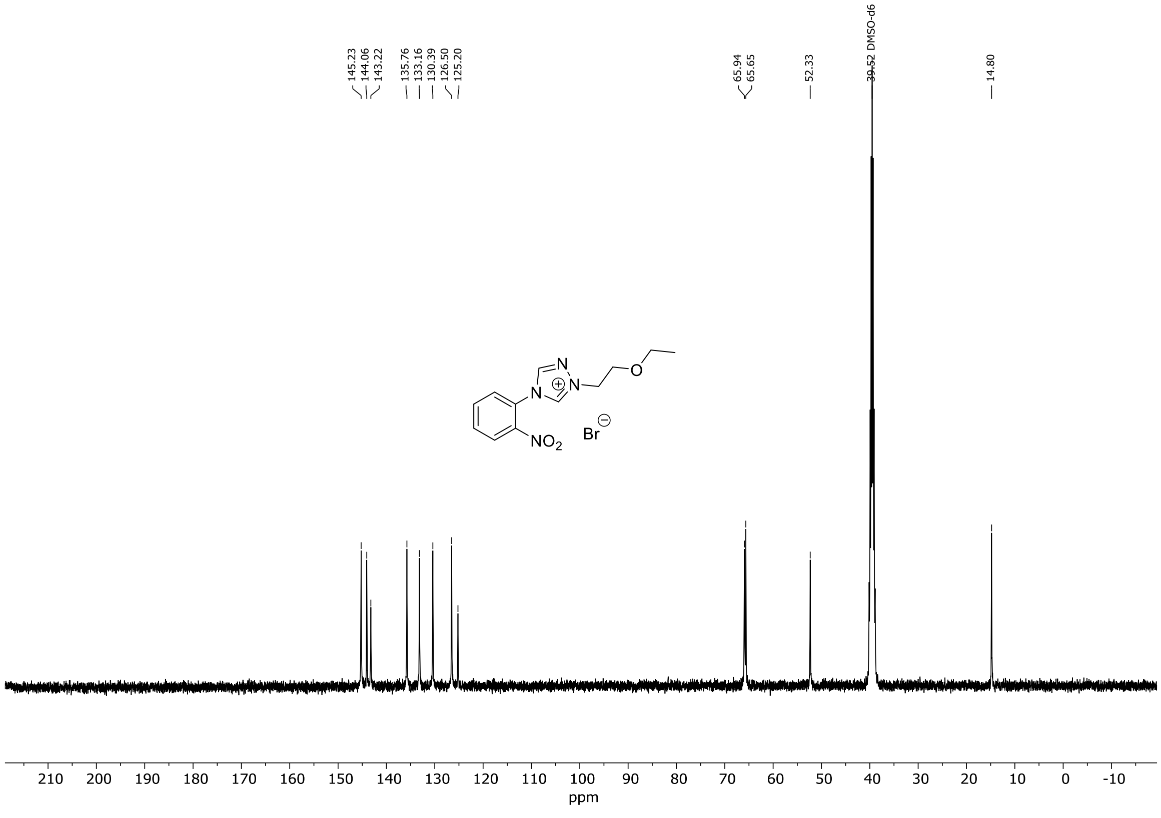

Figure S10: ^{13}C{^{1}H} NMR of 1-(2-ethoxyethyl)-4-(2-nitrophenyl)-4*H*-1,2,4-triazol-1-ium bromide (**2d**) (100 MHz, DMSO-d_6, 298 K).

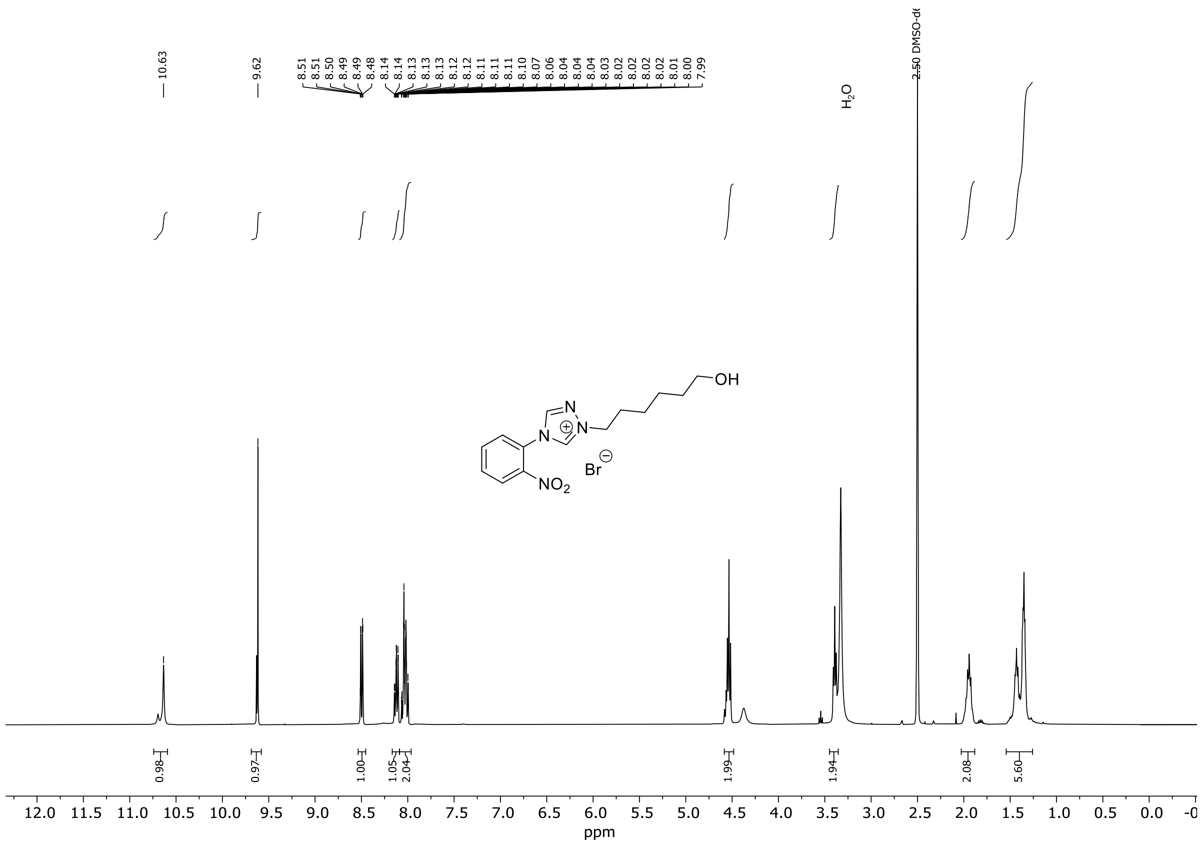

Figure S11: ^{1}H NMR spectrum of 1-(6-hydroxyhexyl)-4-(2-nitrophenyl)-4*H*-1,2,4-triazol-1-ium bromide (**2e**) (400 MHz, DMSO-d_6, 298 K).

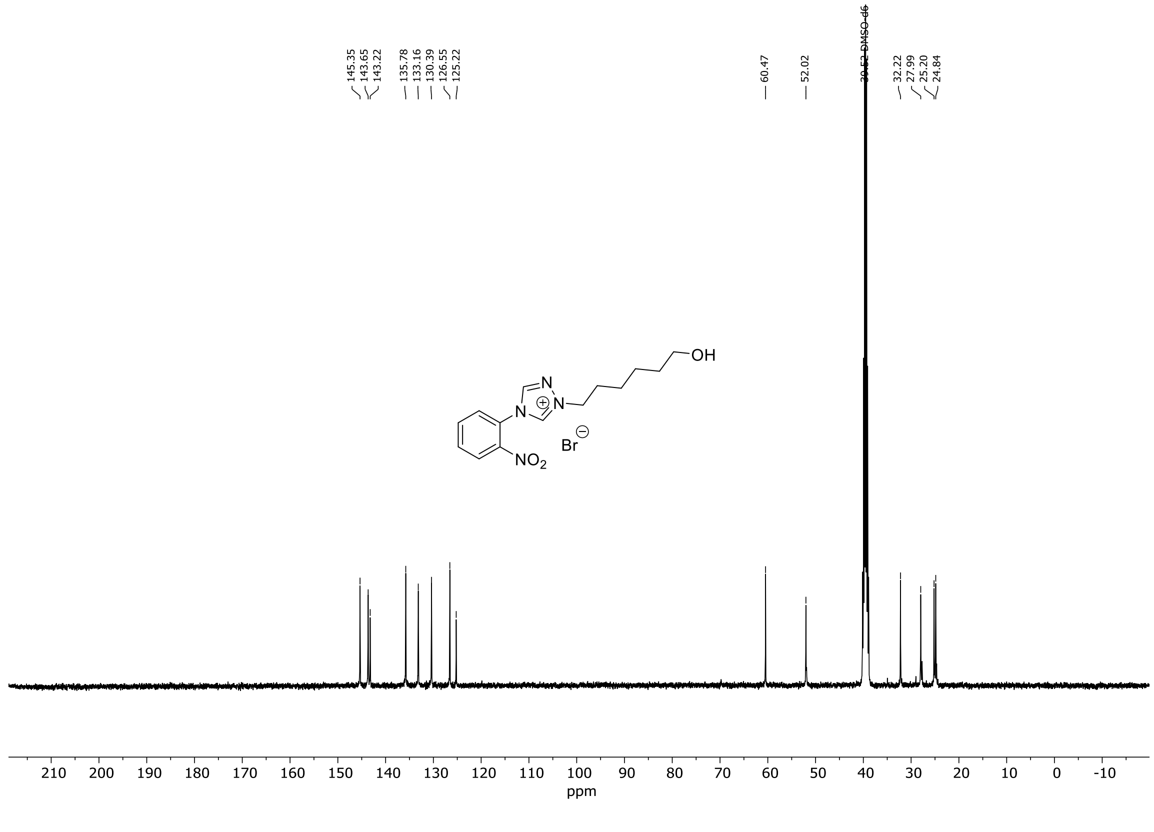

Figure S12: ^{13}C{^{1}H} NMR spectrum of 1-(6-hydroxyhexyl)-4-(2-nitrophenyl)-4*H*-1,2,4-triazol-1-ium bromide (**2e**) (100 MHz, DMSO-d_6, 298 K).

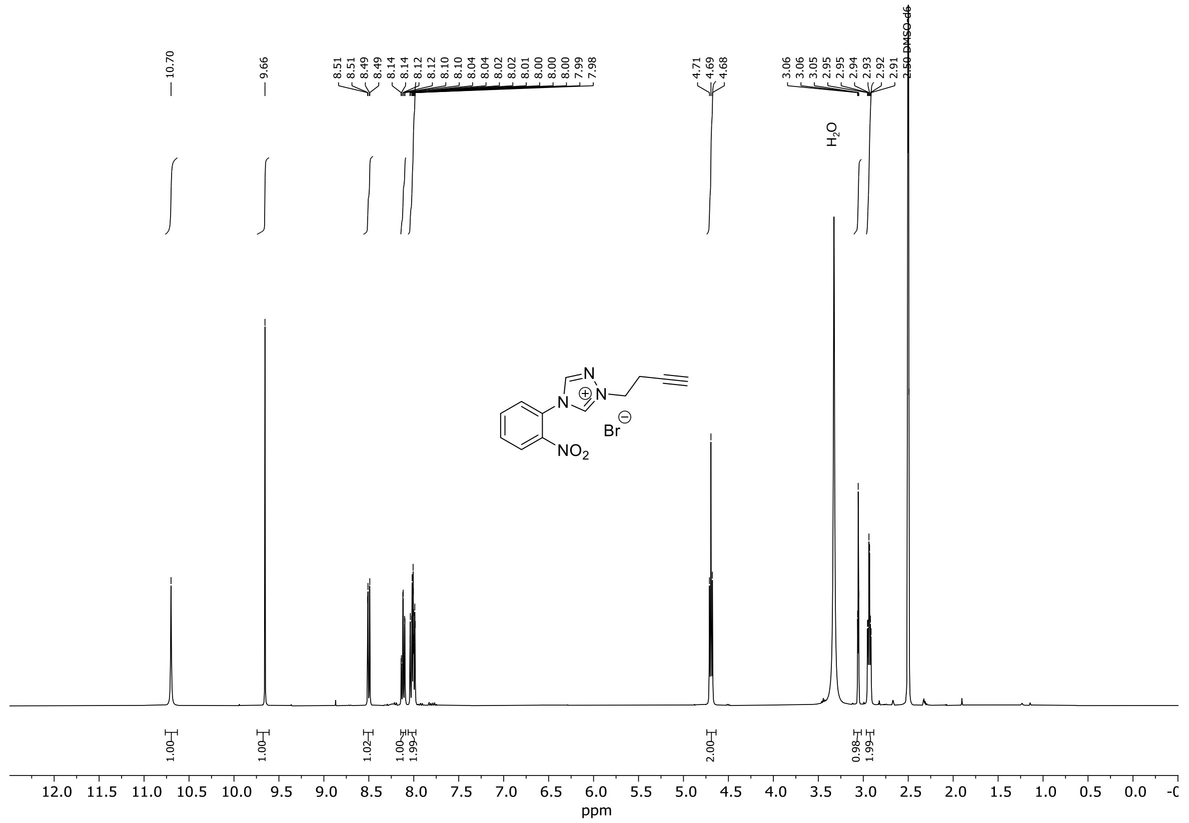

Figure S13: ^{1}H NMR spectrum of 1-(but-3-yn-1-yl)-4-(2-nitrophenyl)-4*H*-1,2,4-triazol-1-ium bromide (**2f**) (400 MHz, DMSO-d_6, 298 K).

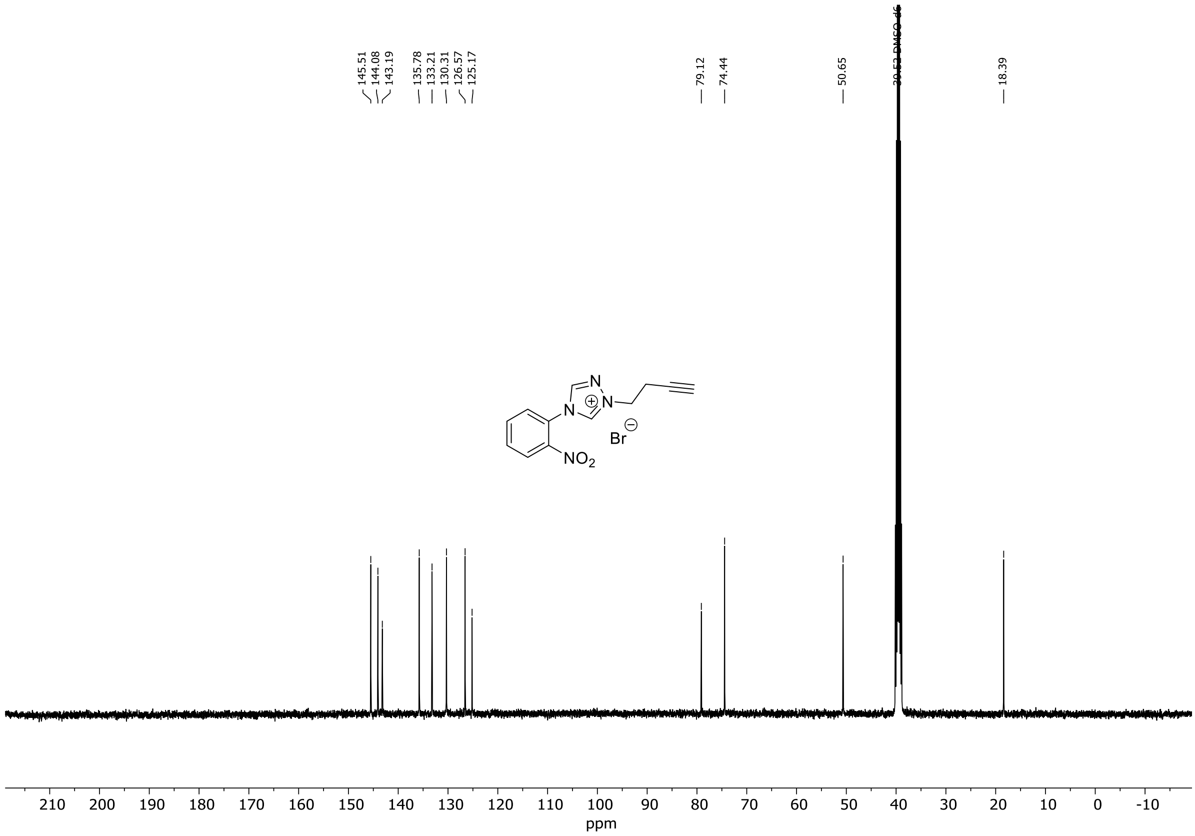

Figure S14: ^{13}C{^{1}H} NMR spectrum of 1-(but-3-yn-1-yl)-4-(2-nitrophenyl)-4*H*-1,2,4-triazol-1-ium bromide (**2f**) (100 MHz, DMSO-d_6, 298 K).

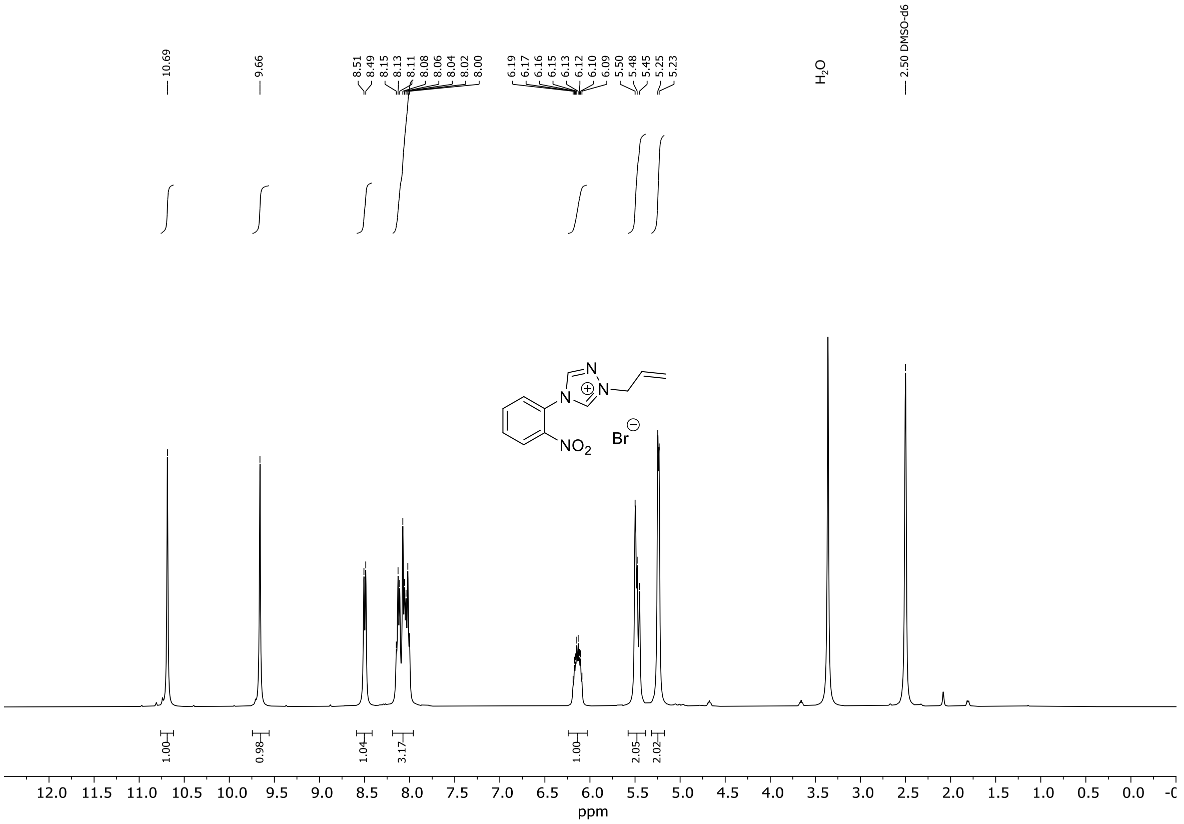

Figure S15: ^{1}H NMR spectrum of 1-allyl-4-(2-nitrophenyl)-4*H*-1,2,4-triazol-1-ium bromide (**2g**) (400 MHz, DMSO-d_6, 298 K).

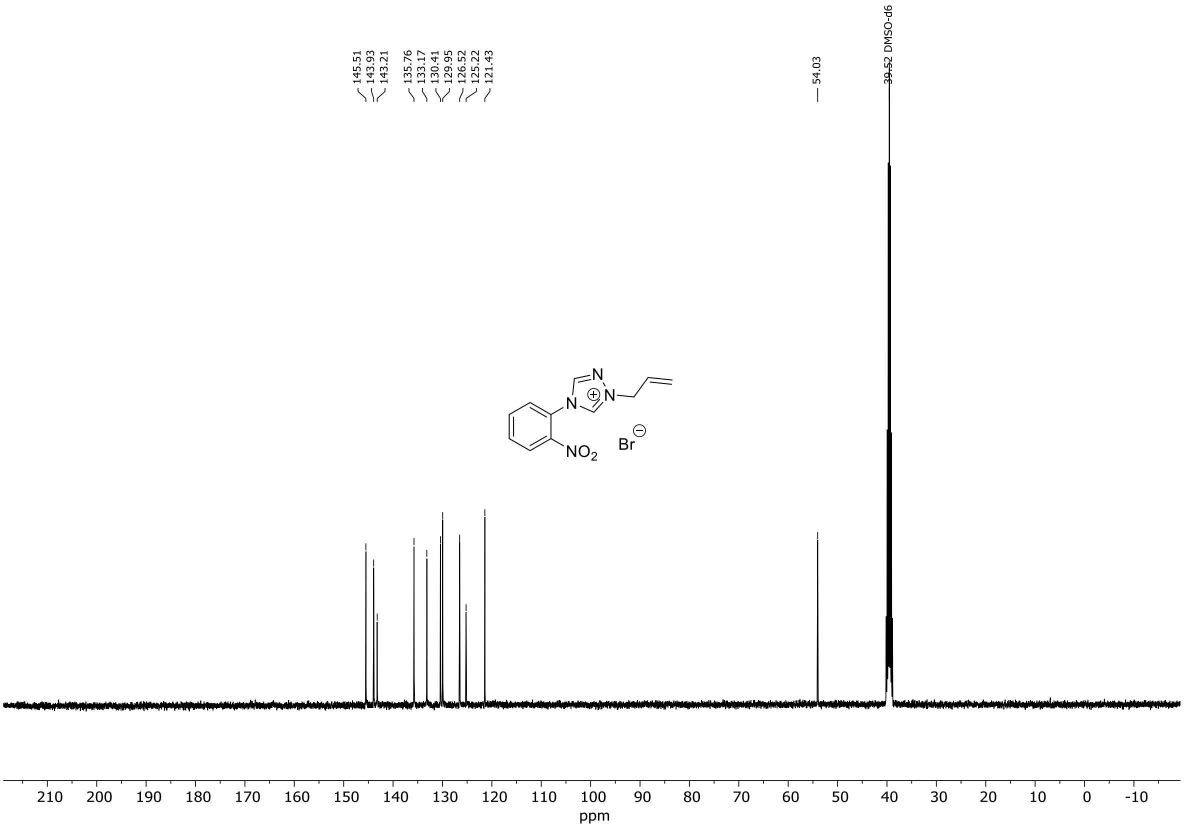

Figure S16: ^{13}C{^{1}H} NMR spectrum of 1-allyl-4-(2-nitrophenyl)-4*H*-1,2,4-triazol-1-ium bromide (**2g**) (100 MHz, DMSO-d_6, 298 K).

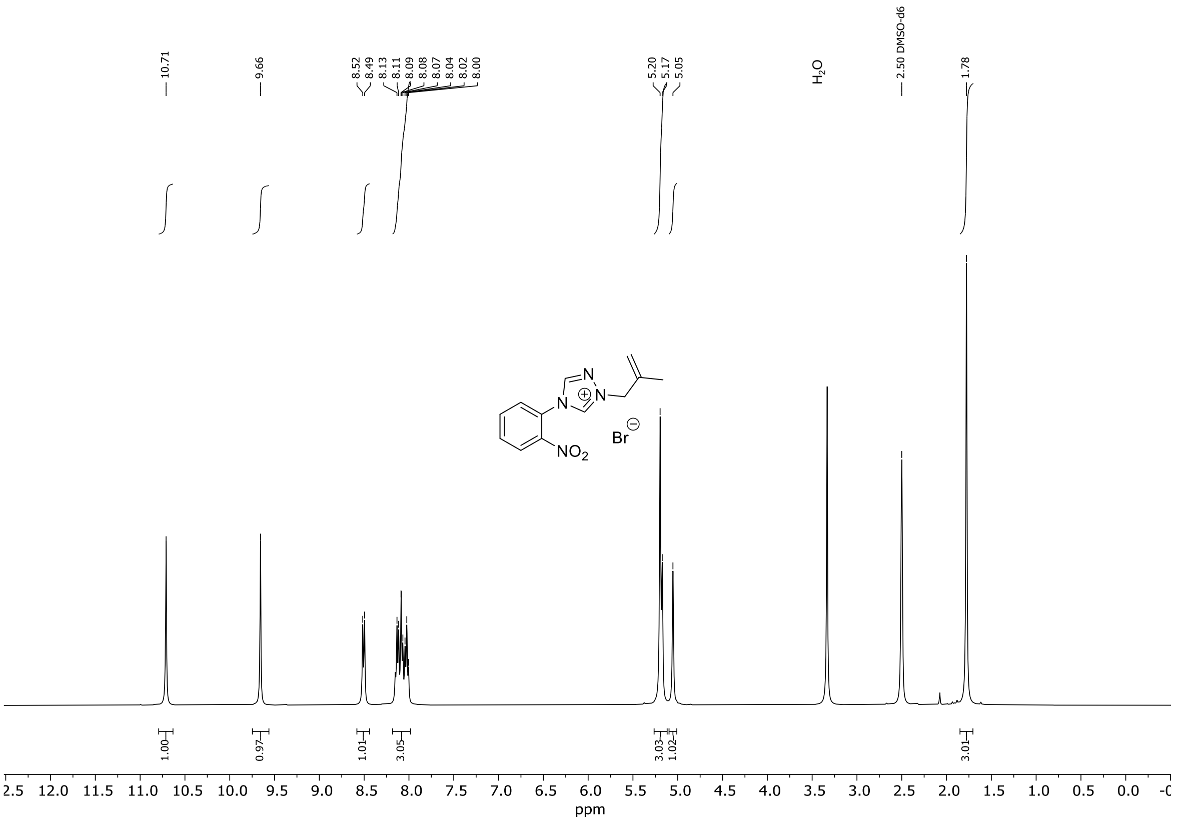

Figure S17: ^{1}H NMR spectrum of 1-(2-methylallyl)-4-(2-nitrophenyl)-4*H*-1,2,4-triazol-1-ium bromide (**2h**) (400 MHz, DMSO-d_6, 298 K).

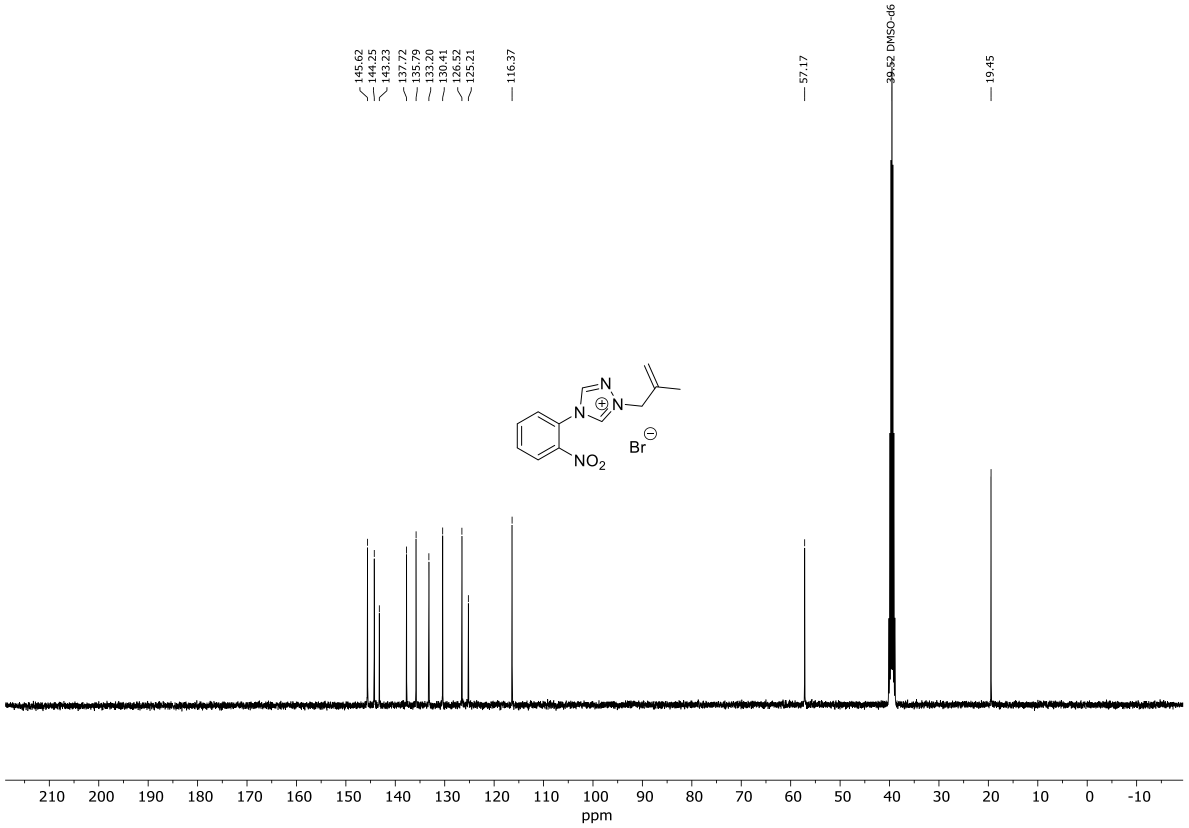

Figure S18: ^{13}C{^{1}H} NMR spectrum of 1-(2-methylallyl)-4-(2-nitrophenyl)-4*H*-1,2,4-triazol-1-ium bromide (**2h**) (100 MHz, DMSO-d_6, 298 K).

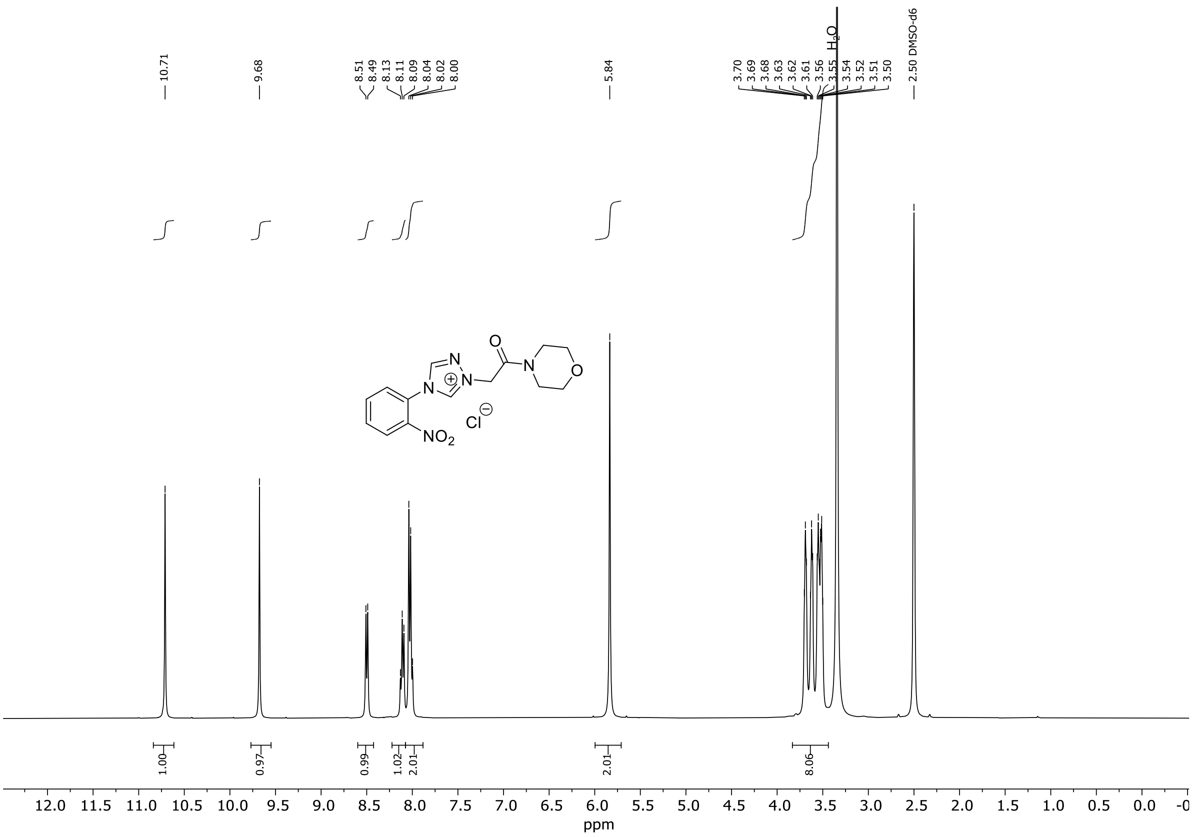

Figure S19: ^{1}H NMR spectrum of 1-(2-morpholino-2-oxoethyl)-4-(2-nitrophenyl)-4*H*-1,2,4-triazol-1-ium chloride (**2i**) (400 MHz, DMSO-d_6, 298 K).

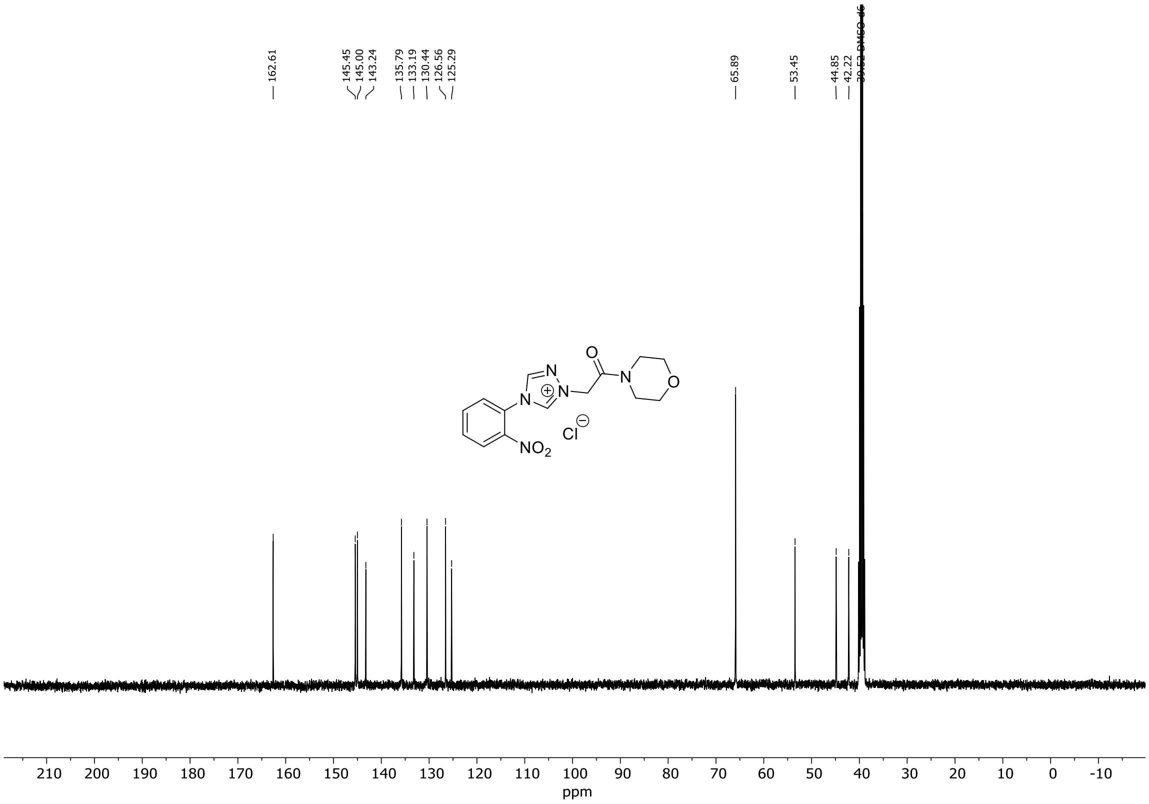

Figure S20: ^{13}C{^{1}H} NMR spectrum of 1-(2-morpholino-2-oxoethyl)-4-(2-nitrophenyl)-4*H*-1,2,4-triazol-1-ium chloride (**2i**) (100 MHz, DMSO-d_6, 298 K).

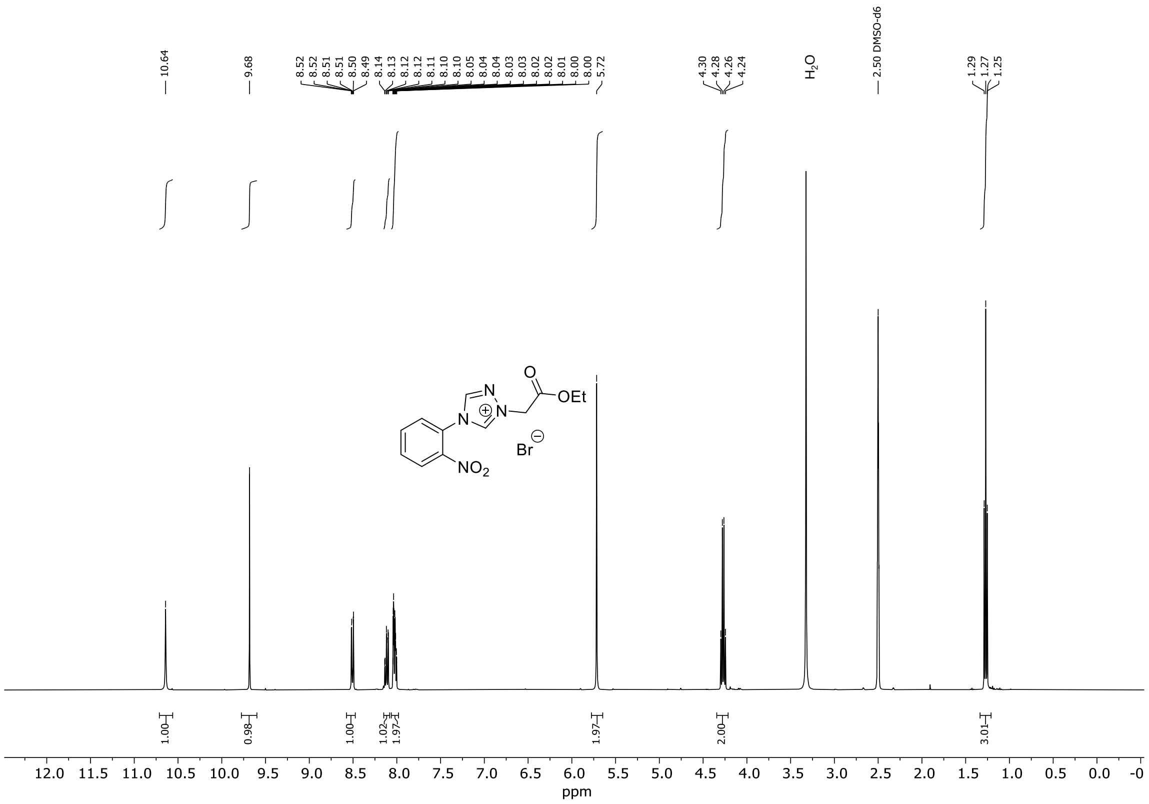

Figure S21: ^{1}H NMR spectrum of 1-(2-ethoxy-2-oxoethyl)-4-(2-nitrophenyl)-4*H*-1,2,4-triazol-1-ium bromide (**2j**) (400 MHz, DMSO-d_6, 298 K).

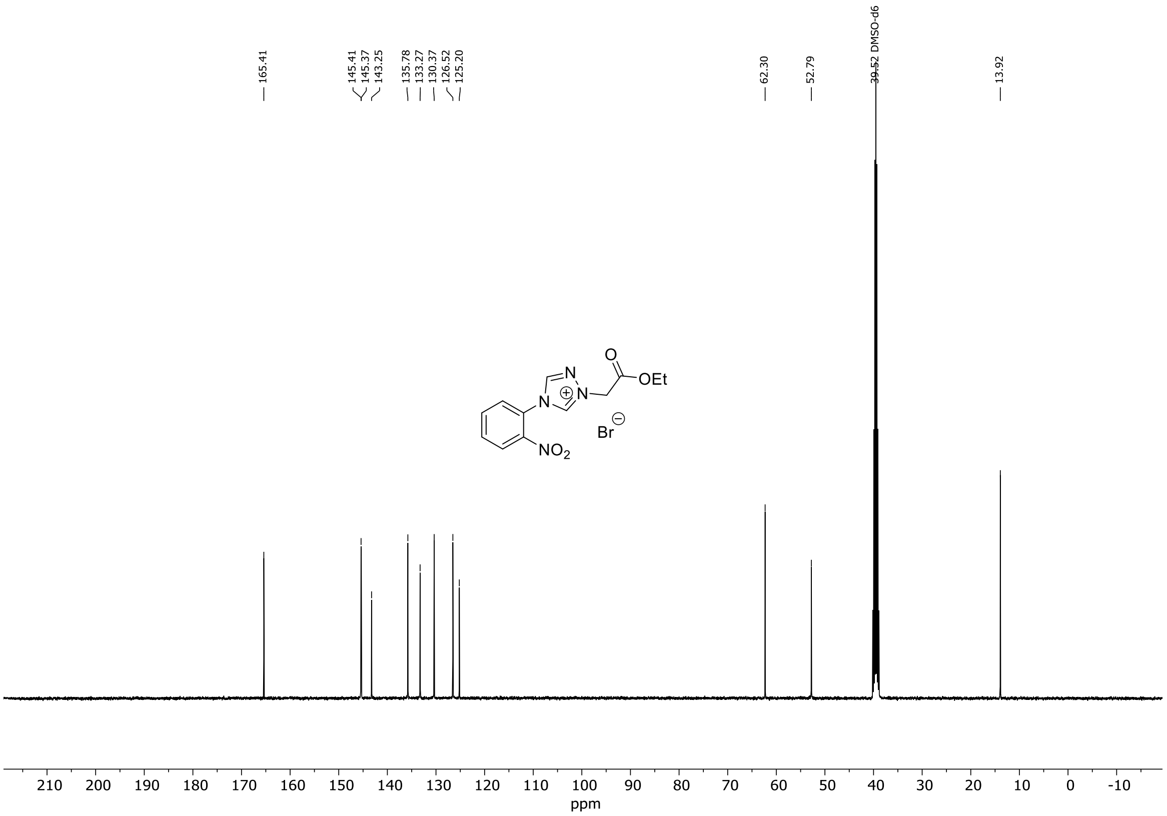

Figure S22: ^{13}C{^{1}H} NMR spectrum of 1-(2-ethoxy-2-oxoethyl)-4-(2-nitrophenyl)-4*H*-1,2,4-triazol-1-ium bromide (**2j**) (100 MHz, DMSO-d_6, 298 K).

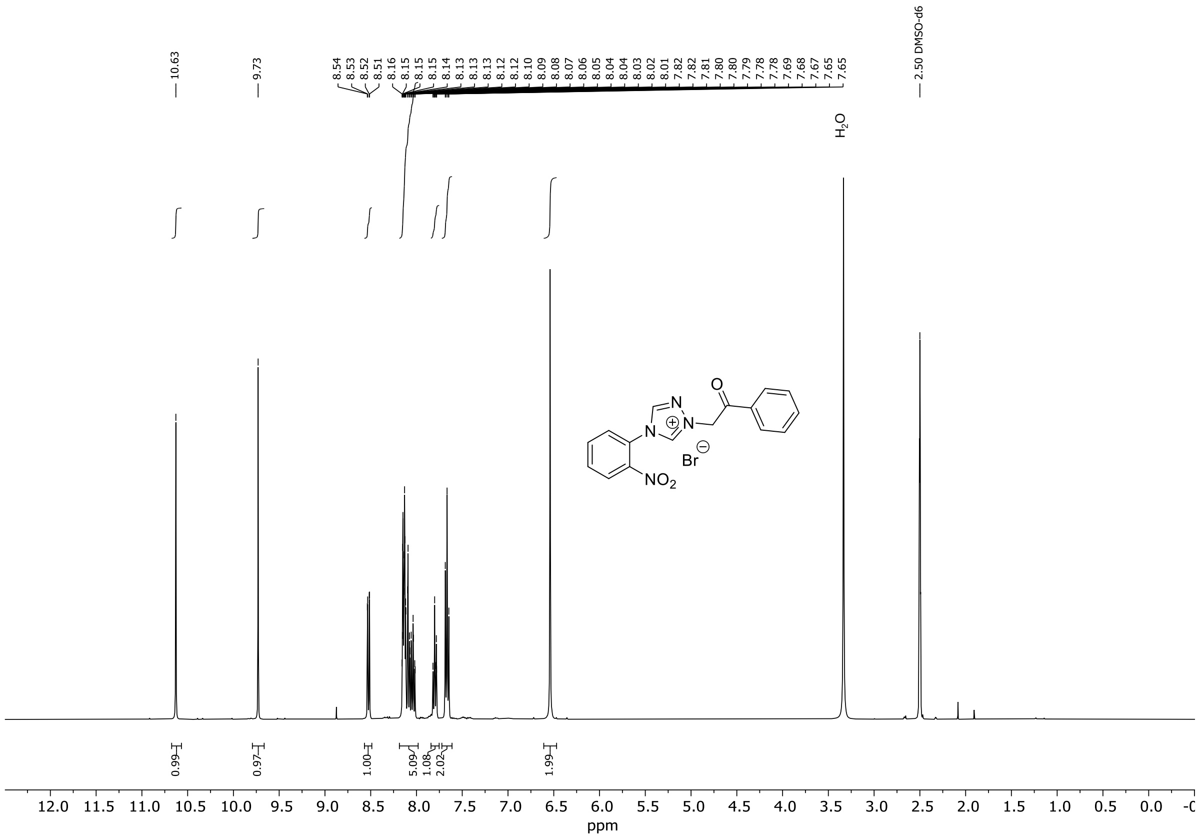

Figure S23: ^{1}H NMR spectrum of 4-(2-nitrophenyl)-1-(2-oxo-2-phenylethyl)-4*H*-1,2,4-triazol-1-ium bromide (**2k**) (400 MHz, DMSO-d_6, 298 K).

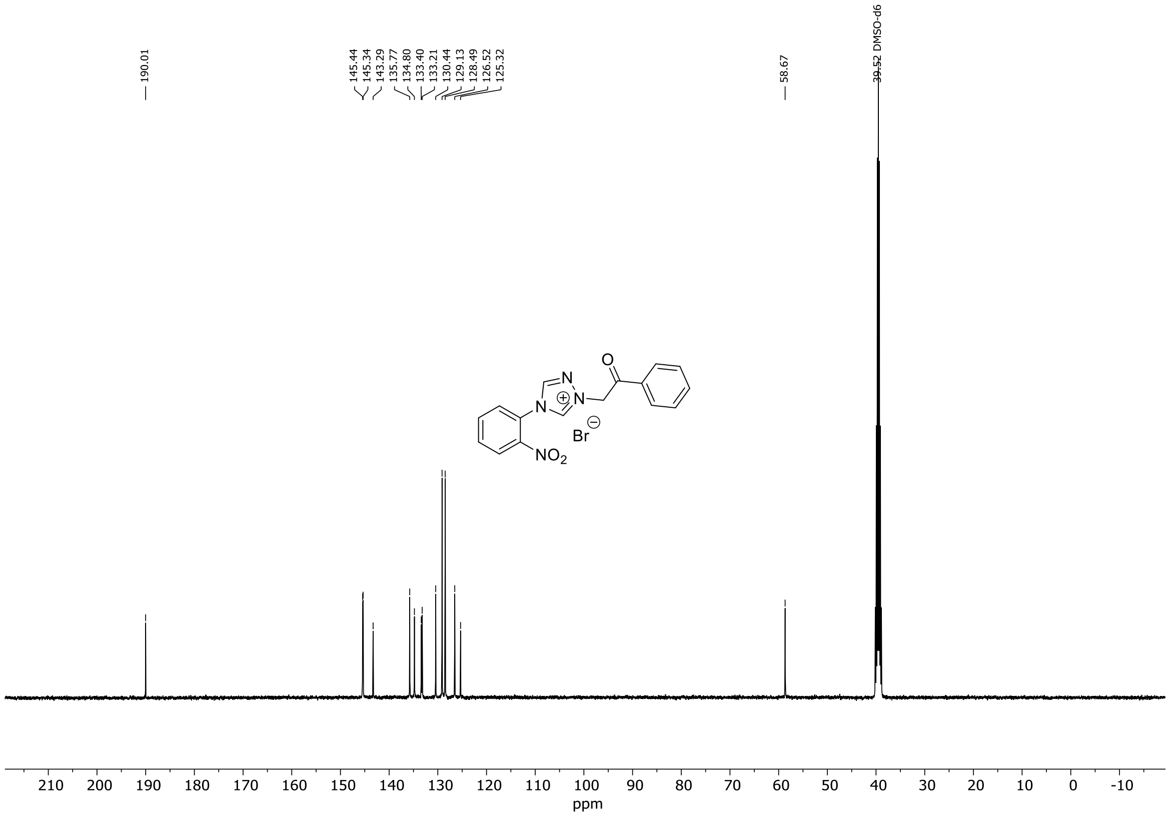

Figure S24: ^{13}C{^{1}H} NMR spectrum of 4-(2-nitrophenyl)-1-(2-oxo-2-phenylethyl)-4*H*-1,2,4-triazol-1-ium bromide (**2k**) (100 MHz, DMSO-d_6, 298 K).

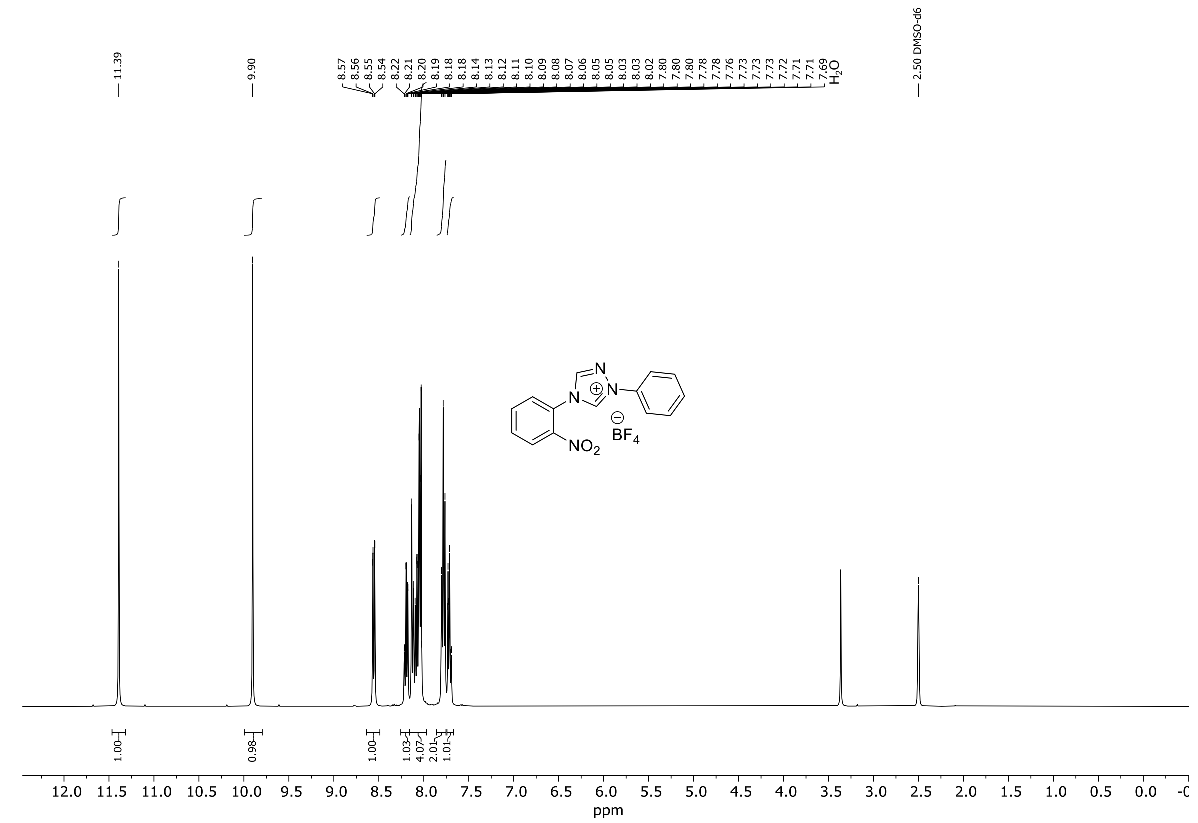

Figure S25: ^{1}H NMR spectrum of 4-(2-nitrophenyl)-1-phenyl-4*H*-1,2,4-triazol-1-ium tetrafluoroborate (**3a**) (400 MHz, DMSO-d_6, 298 K).

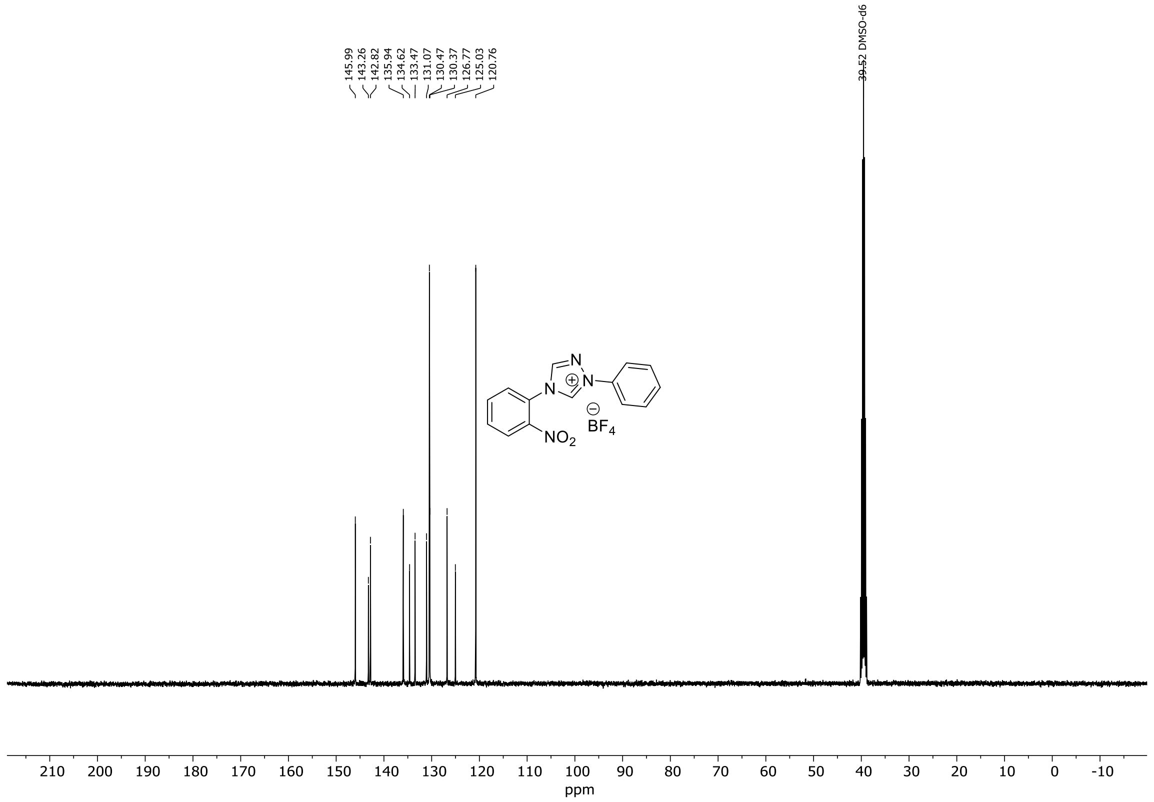

Figure S26: ^{13}C{^{1}H} NMR spectrum of 4-(2-nitrophenyl)-1-phenyl-4*H*-1,2,4-triazol-1-ium tetrafluoroborate (**3a**) (100 MHz, DMSO-d_6, 298 K).

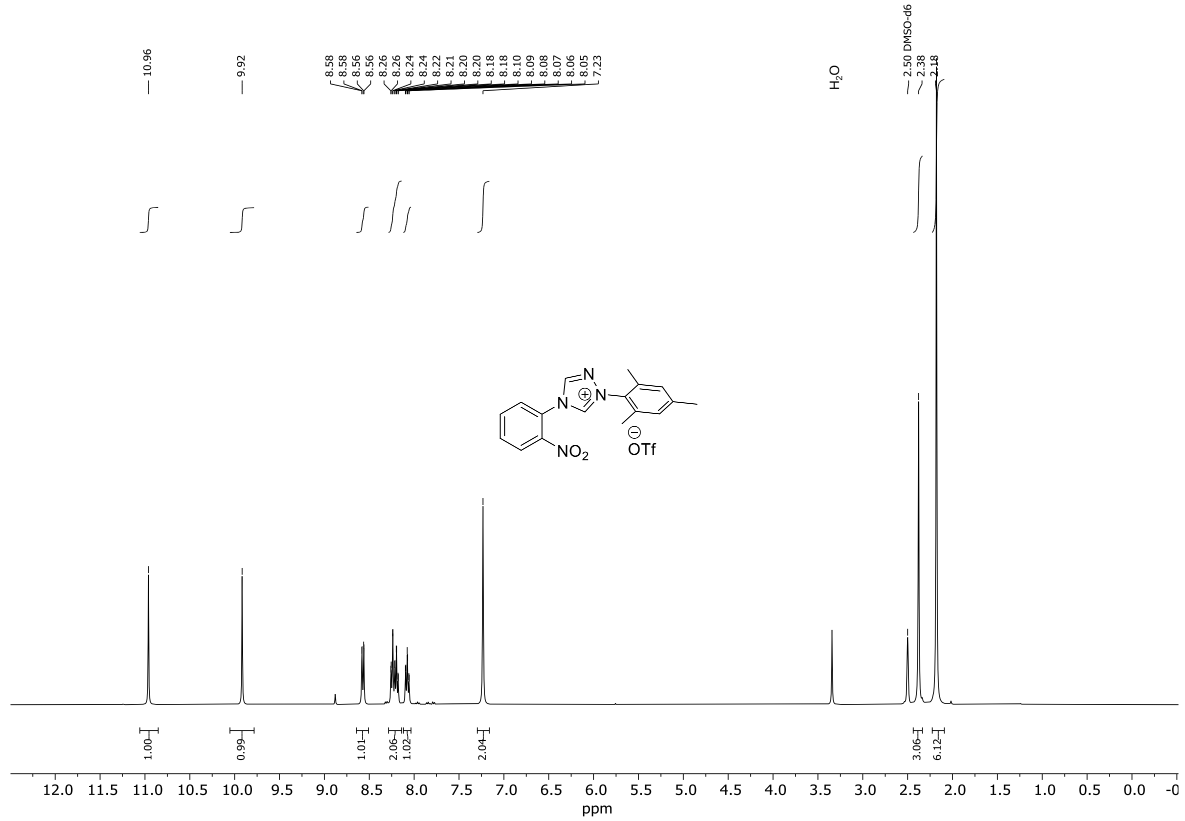

Figure S27: ^{1}H NMR spectrum of 1-mesityl-4-(2-nitrophenyl)-4*H*-1,2,4-triazol-1-ium trifluoromethanesulfonate (**3b**) (400 MHz, DMSO-d_6, 298 K).

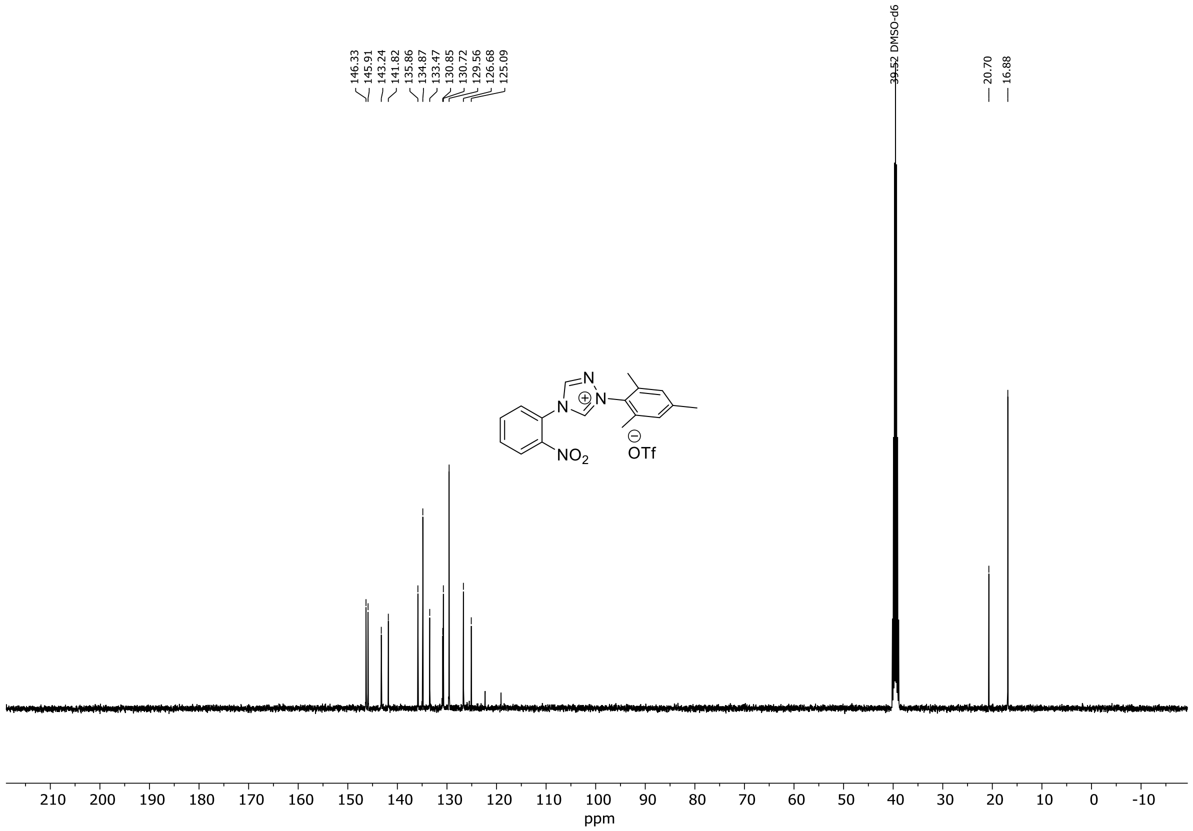

Figure S28: $^{13}C\{^{1}H\}$ NMR spectrum of 1-mesityl-4-(2-nitrophenyl)-4*H*-1,2,4-triazol-1-ium trifluoromethanesulfonate (**3b**) (100 MHz, DMSO-d_6, 298 K).

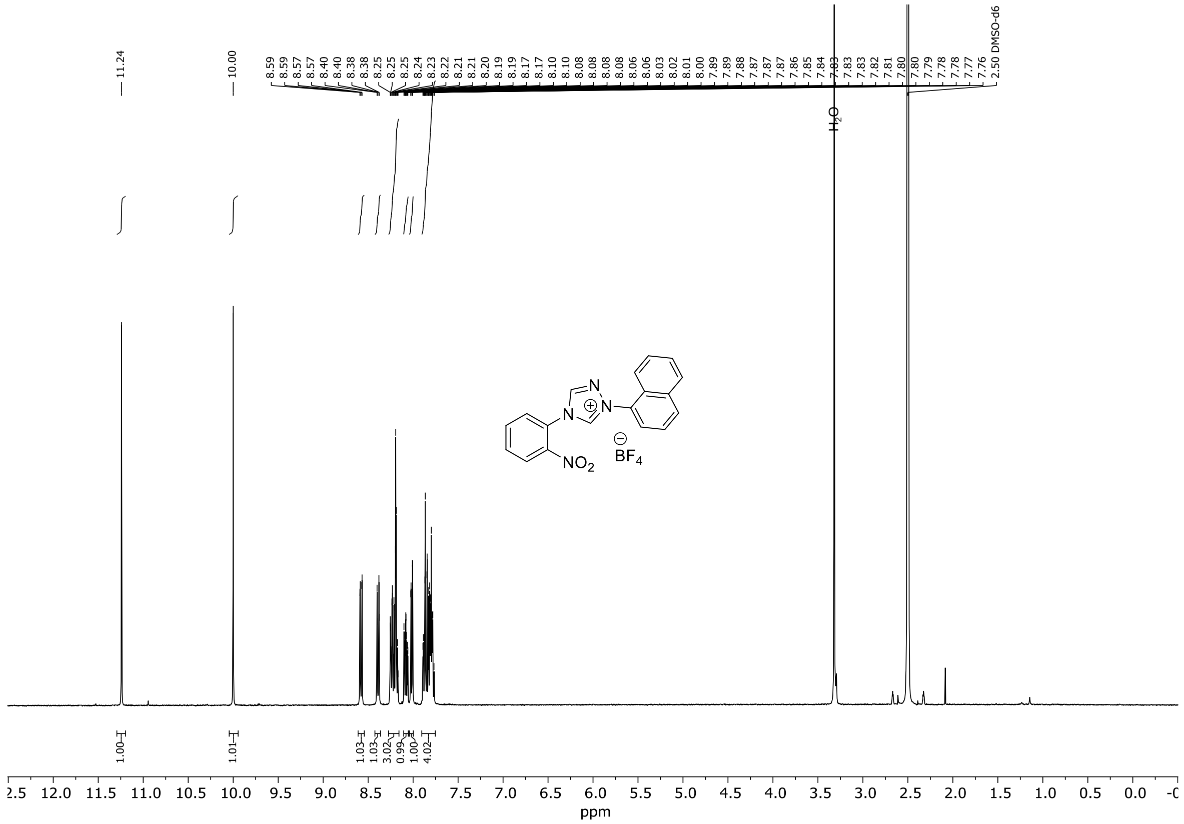

Figure S29: ^{1}H NMR spectrum of 1-(naphthalen-1-yl)-4-(2-nitrophenyl)-4*H*-1,2,4-triazol-1-ium tetrafluoroborate (**3c**) (400 MHz, DMSO-d_6, 298 K).

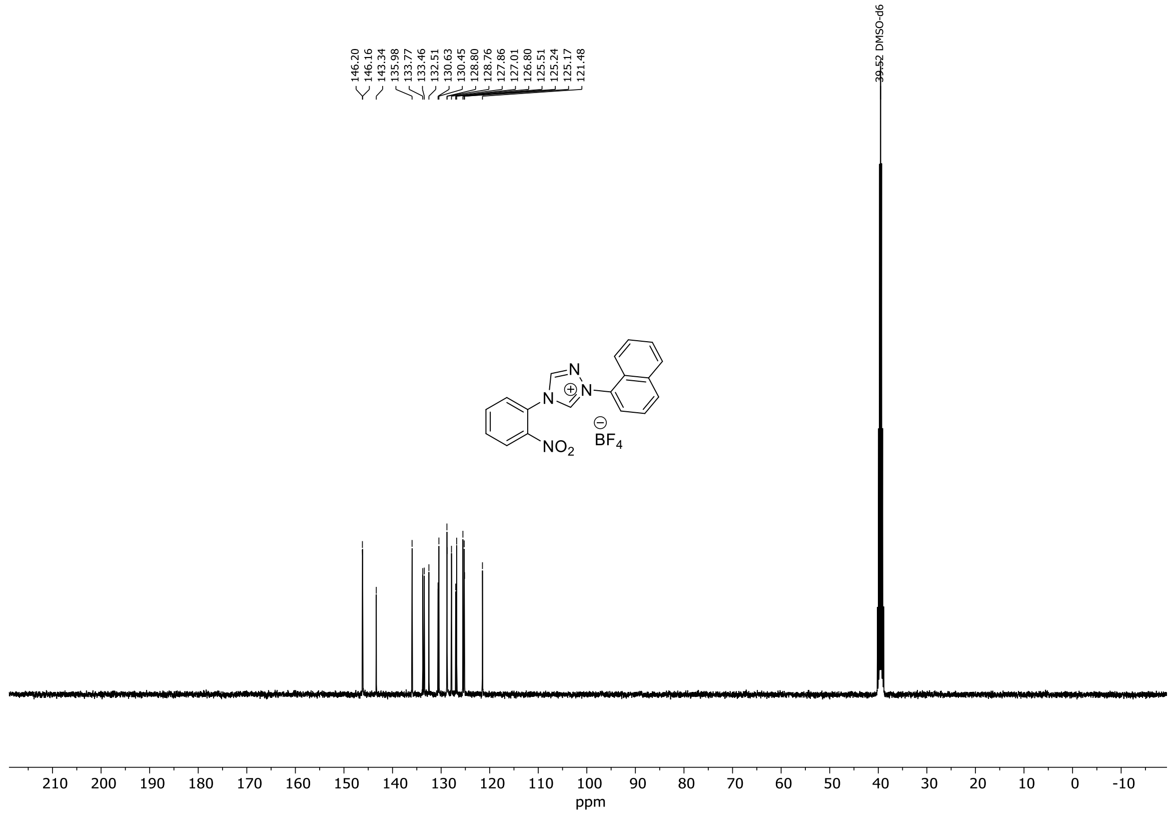

Figure S30: $^{13}C\{^{1}H\}$ NMR NMR spectrum of 1-(naphthalen-1-yl)-4-(2-nitrophenyl)-4*H*-1,2,4-triazol-1-ium tetrafluoroborate (**3c**) (100 MHz, DMSO-d_6, 298 K).

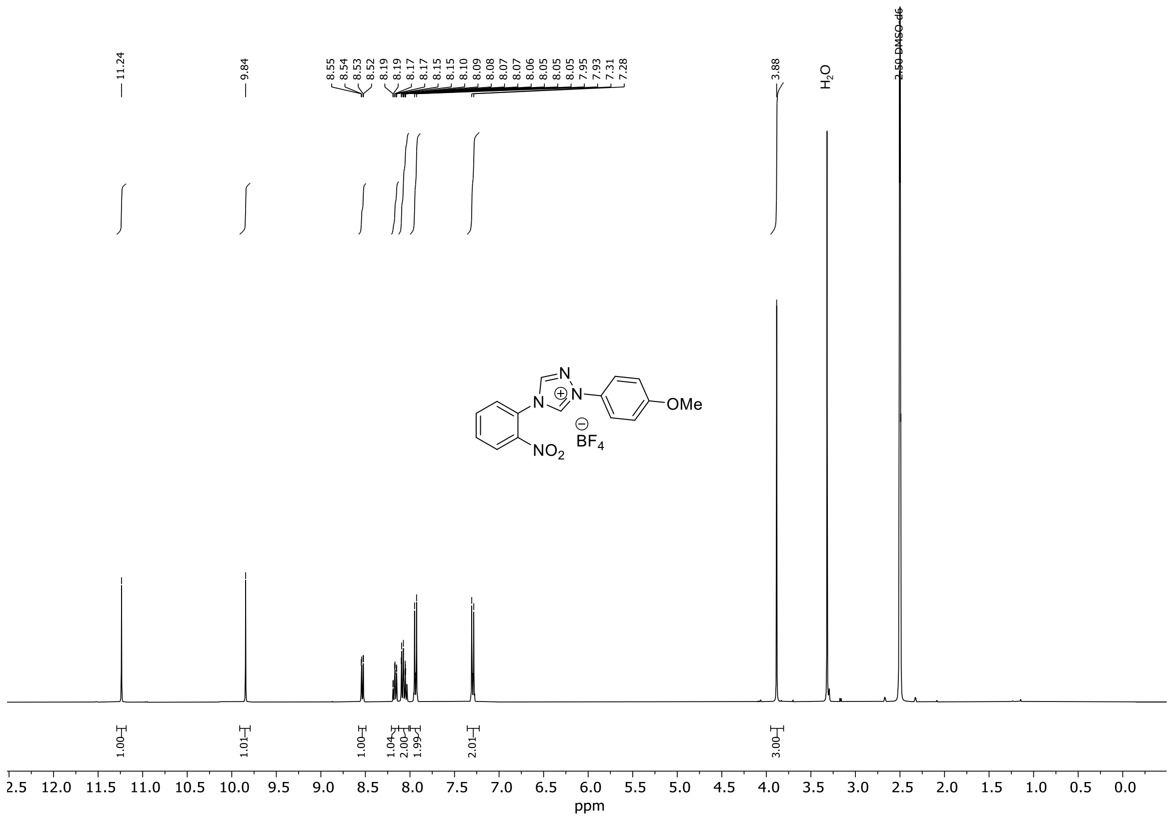

Figure S31: ^{1}H NMR spectrum of 1-(4-methoxyphenyl)-4-(2-nitrophenyl)-4*H*-1,2,4-triazol-1-ium tetrafluoroborate (**3d**) (400 MHz, DMSO-d_6, 298 K).

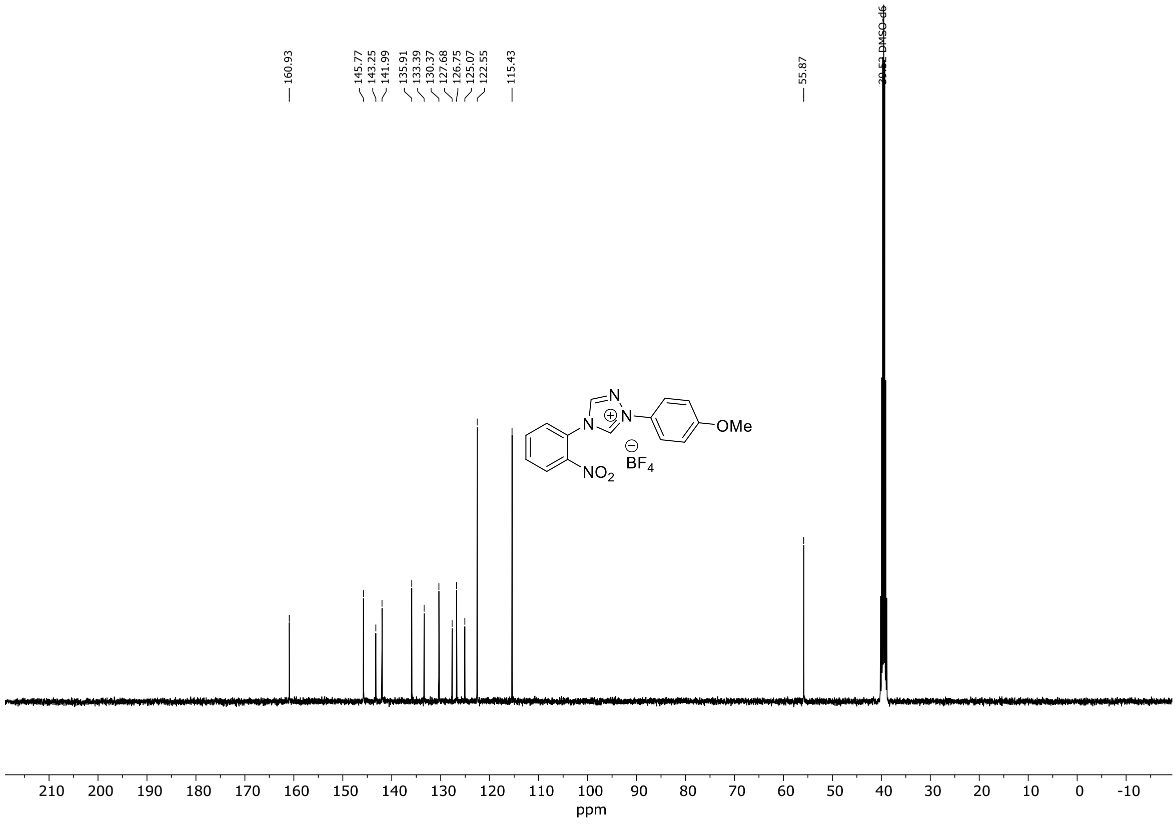

Figure S32: $^{13}C\{^{1}H\}$ NMR spectrum of 1-(4-methoxyphenyl)-4-(2-nitrophenyl)-4*H*-1,2,4-triazol-1-ium tetrafluoroborate (**3d**) (100 MHz, DMSO-d_6, 298 K).

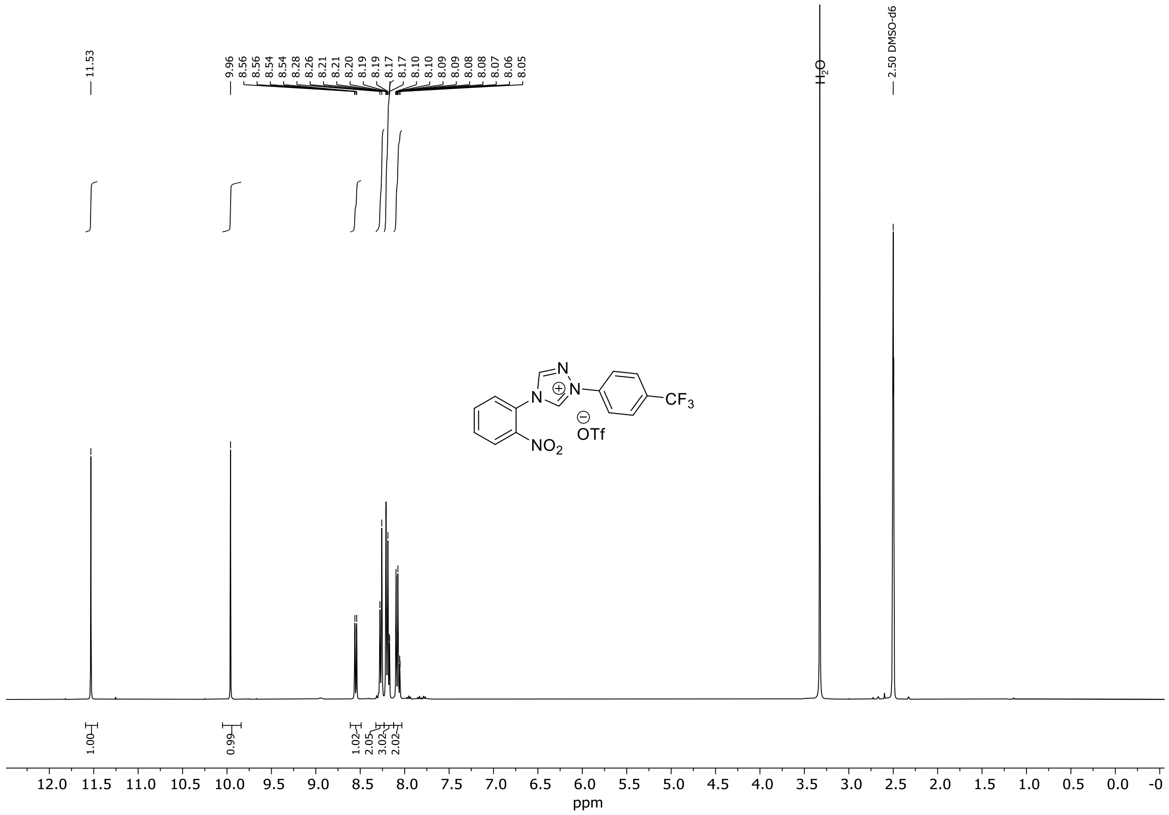

Figure S33: ^{1}H NMR spectrum of 4-(2-nitrophenyl)-1-(4-(trifluoromethyl)phenyl)-4*H*-1,2,4-triazol-1-ium trifluoromethanesulfonate (**3e**) (400 MHz, DMSO-d_6, 298 K).

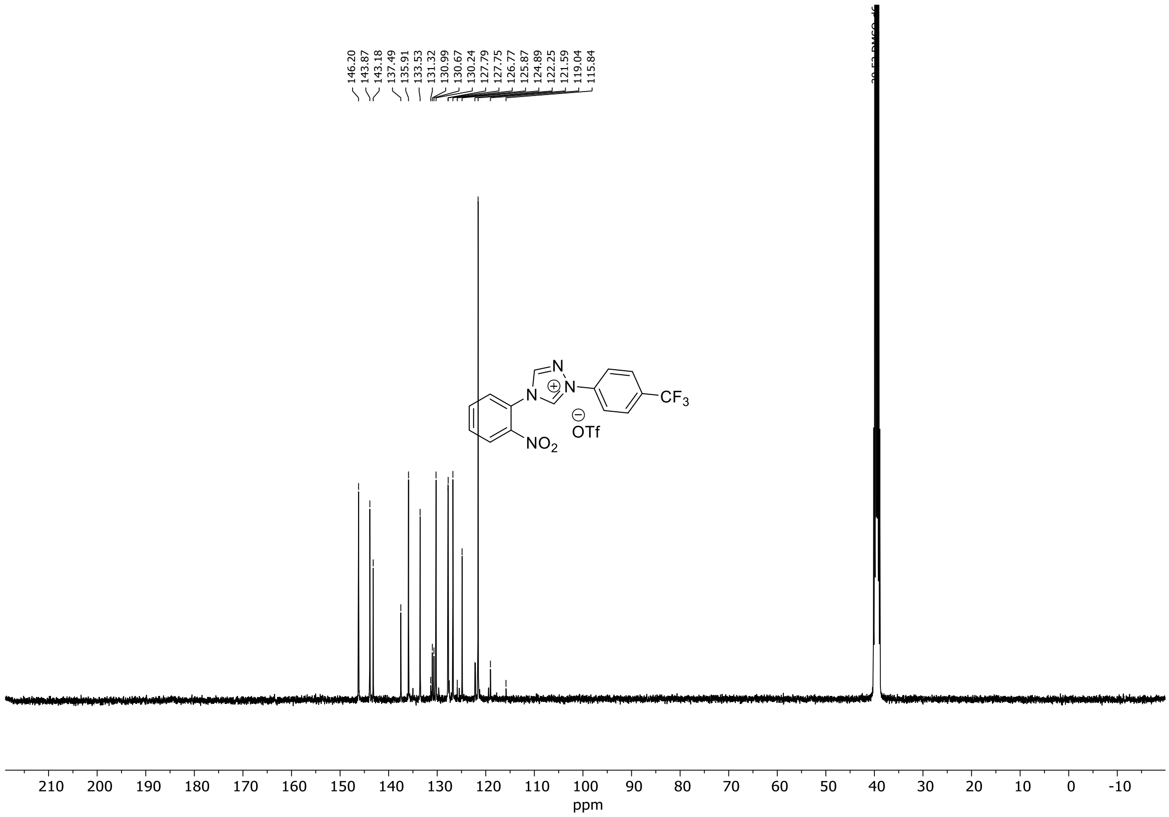

Figure S34: $^{13}C\{^{1}H\}$ NMR spectrum of 4-(2-nitrophenyl)-1-(4-(trifluoromethyl)phenyl)-4*H*-1,2,4-triazol-1-ium trifluoromethanesulfonate (**3e**) (100 MHz, DMSO-d_6, 298 K).

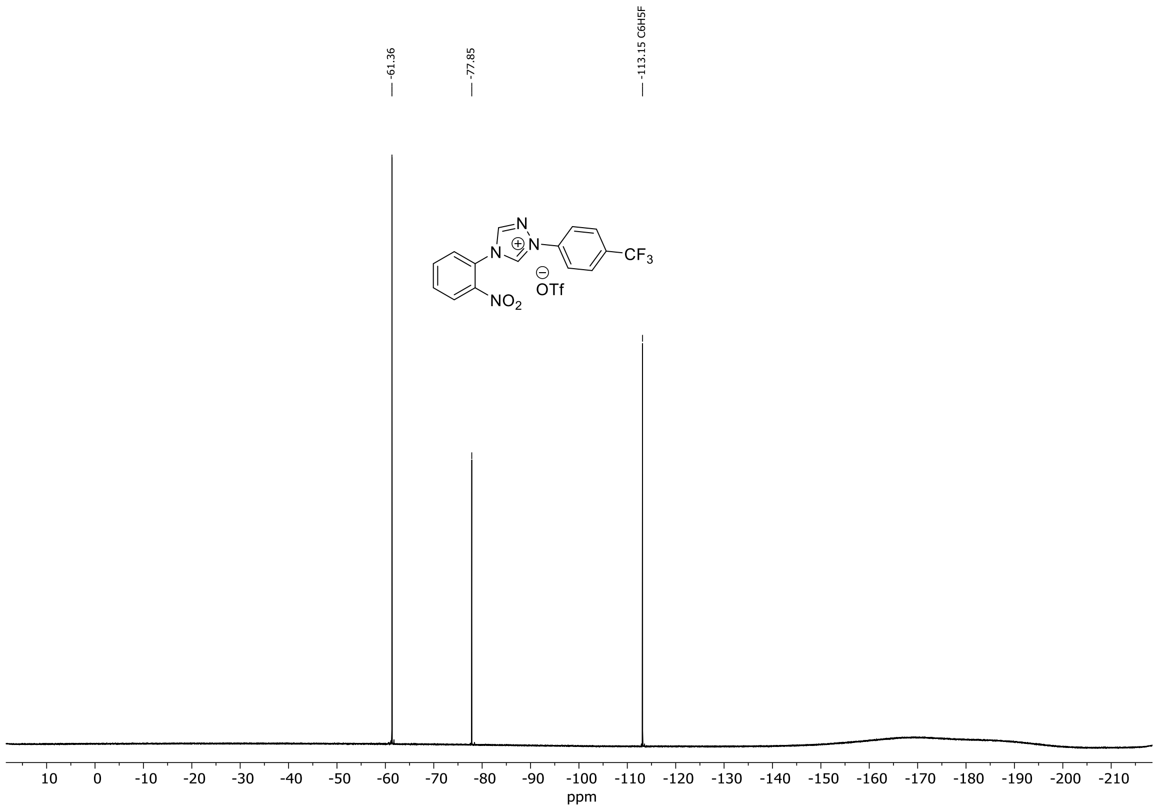

Figure S35: $^{19}F\{^{1}H\}$ NMR spectrum of 4-(2-nitrophenyl)-1-(4-(trifluoromethyl)phenyl)-4*H*-1,2,4-triazol-1-ium trifluoromethanesulfonate (**3e**) (376 MHz, DMSO-d_6, 298 K, referenced to fluorobenzene).

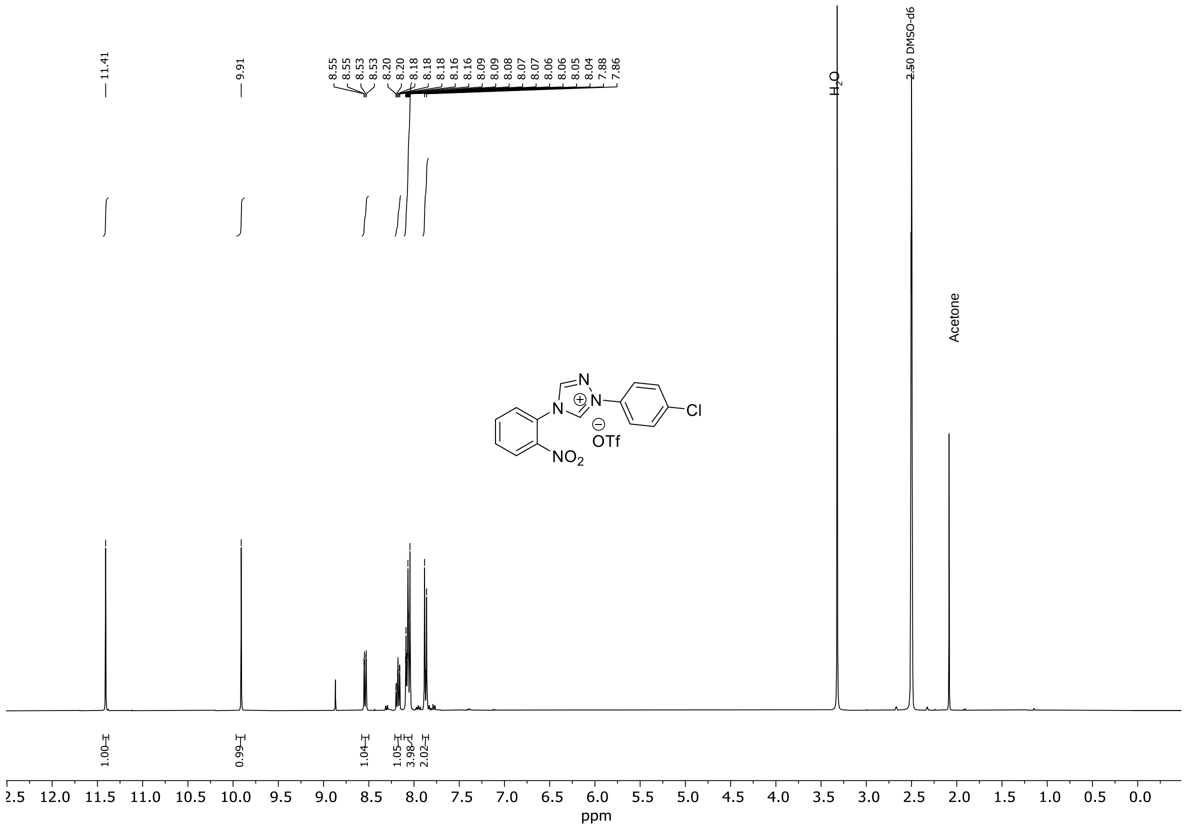

Figure S36: ^{1}H NMR spectrum of 1-(4-chlorophenyl)-4-(2-nitrophenyl)-4*H*-1,2,4-triazol-1-ium trifluoromethanesulfonate (**3f**) (400 MHz, DMSO-d_6, 298 K).

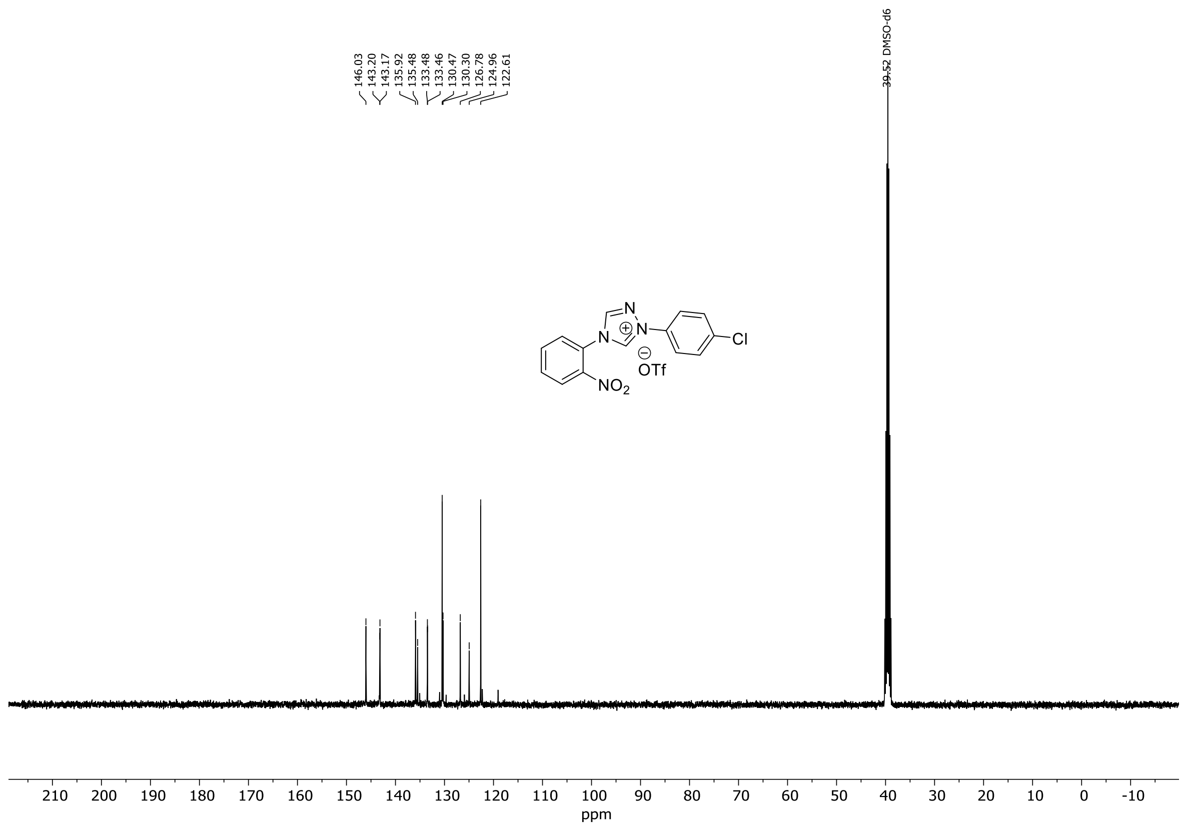

Figure S37: $^{13}C\{^1H\}$ NMR spectrum of 1-(4-chlorophenyl)-4-(2-nitrophenyl)-4*H*-1,2,4-triazol-1-ium trifluoromethanesulfonate (**f**

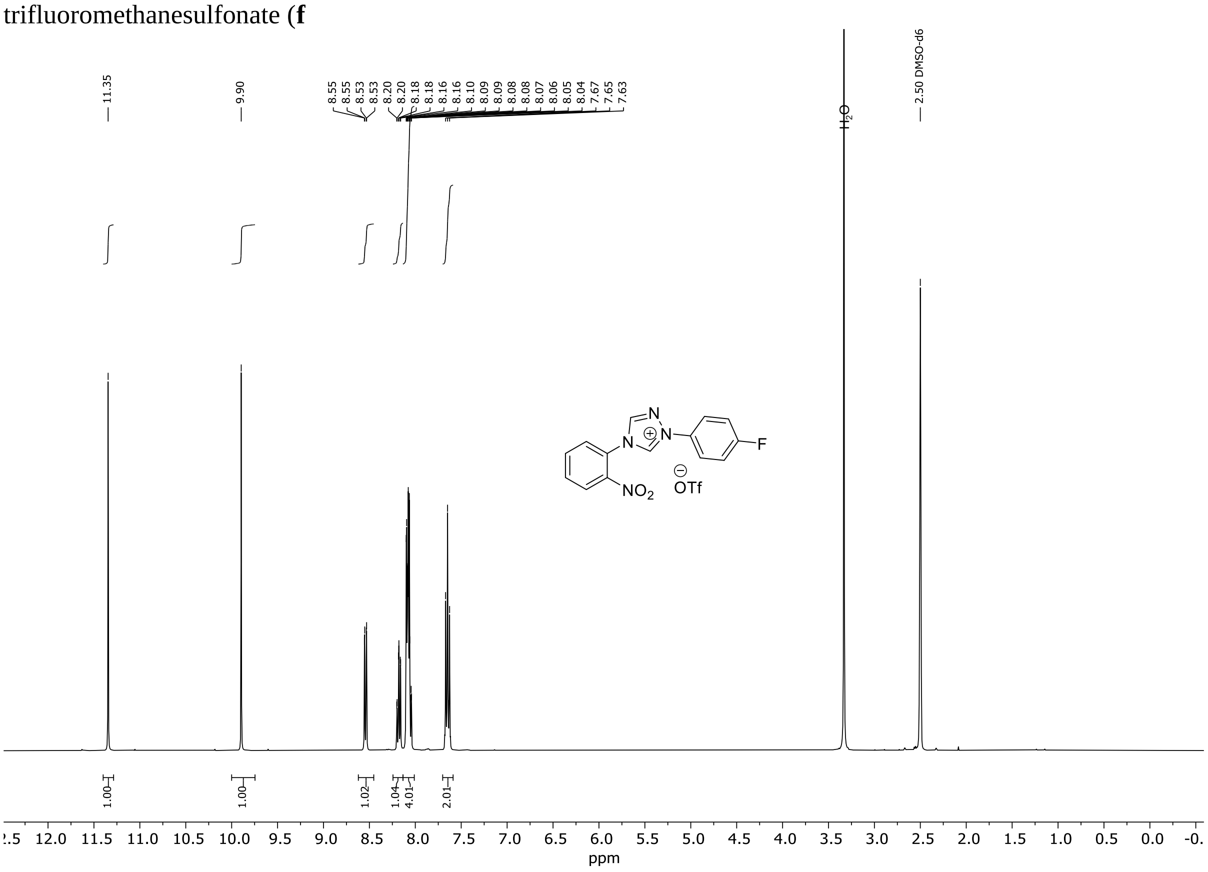

Figure S38: ^1H NMR spectrum of 1-(4-fluorophenyl)-4-(2-nitrophenyl)-4*H*-1,2,4-triazol-1-ium trifluoromethanesulfonate (**3g**) (400 MHz, DMSO-d_6, 298 K).

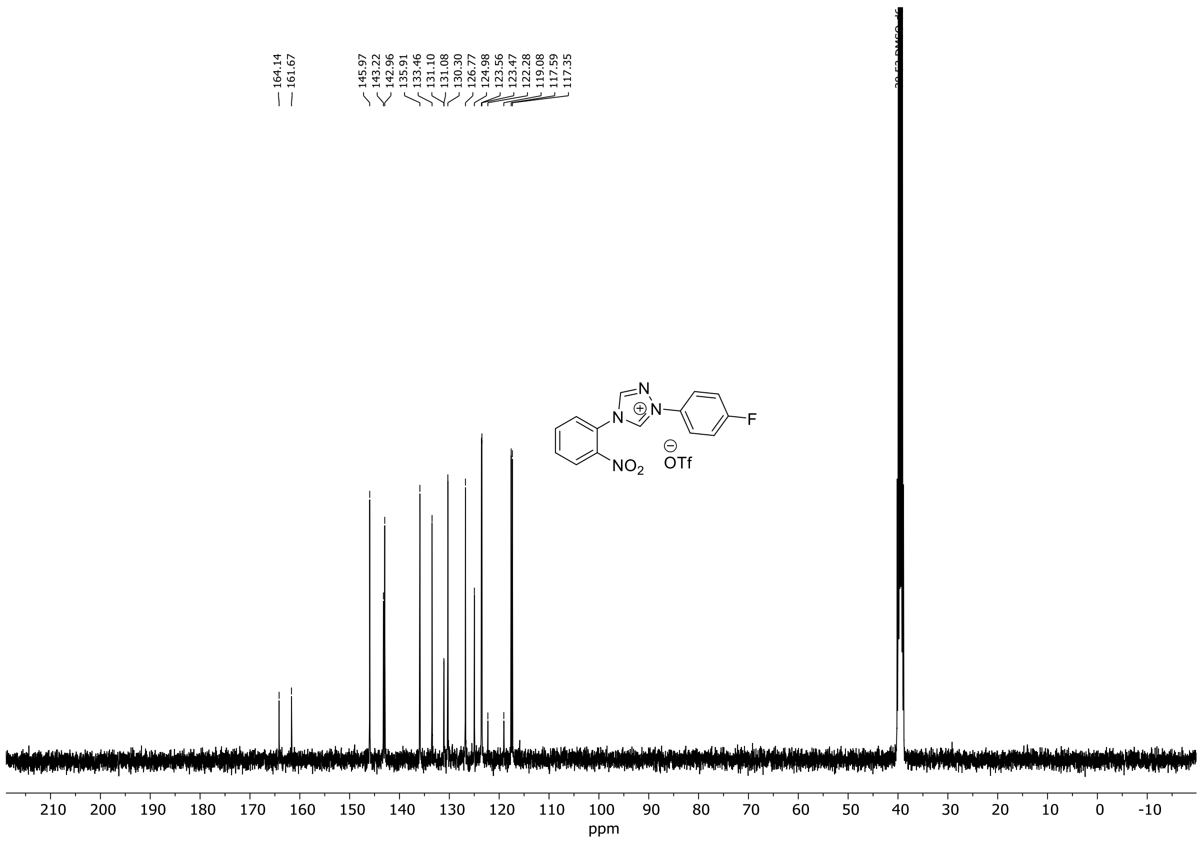

Figure S39: $^{13}C\{^{1}H\}$ NMR spectrum of 1-(4-fluorophenyl)-4-(2-nitrophenyl)-4*H*-1,2,4-triazol-1-ium trifluoromethanesulfonate (**3g**) (100 MHz, DMSO-d_6, 298 K).

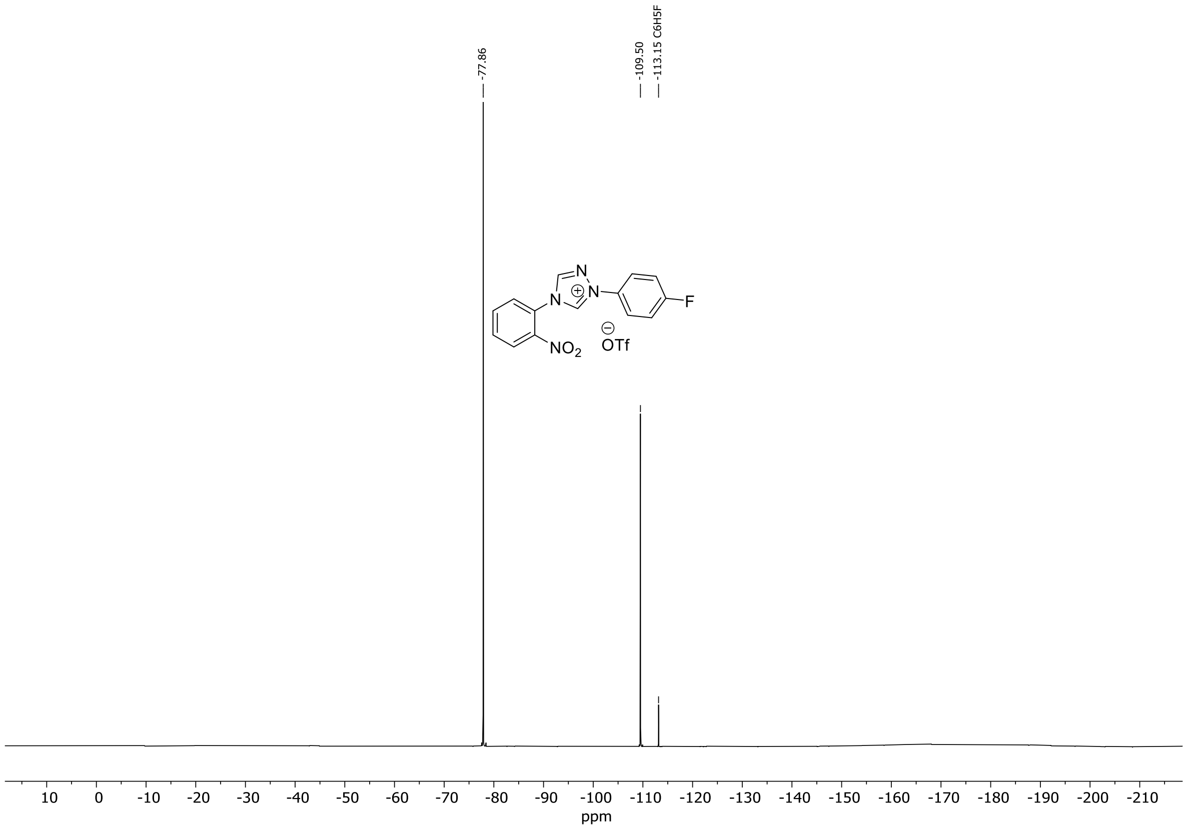

Figure S40: $^{19}F\{^{1}H\}$ NMR spectrum of 1-(4-fluorophenyl)-4-(2-nitrophenyl)-4*H*-1,2,4-triazol-1-ium trifluoromethanesulfonate (**3g**) (376 MHz, DMSO-d_6, 298 K, referenced to fluorobenzene).

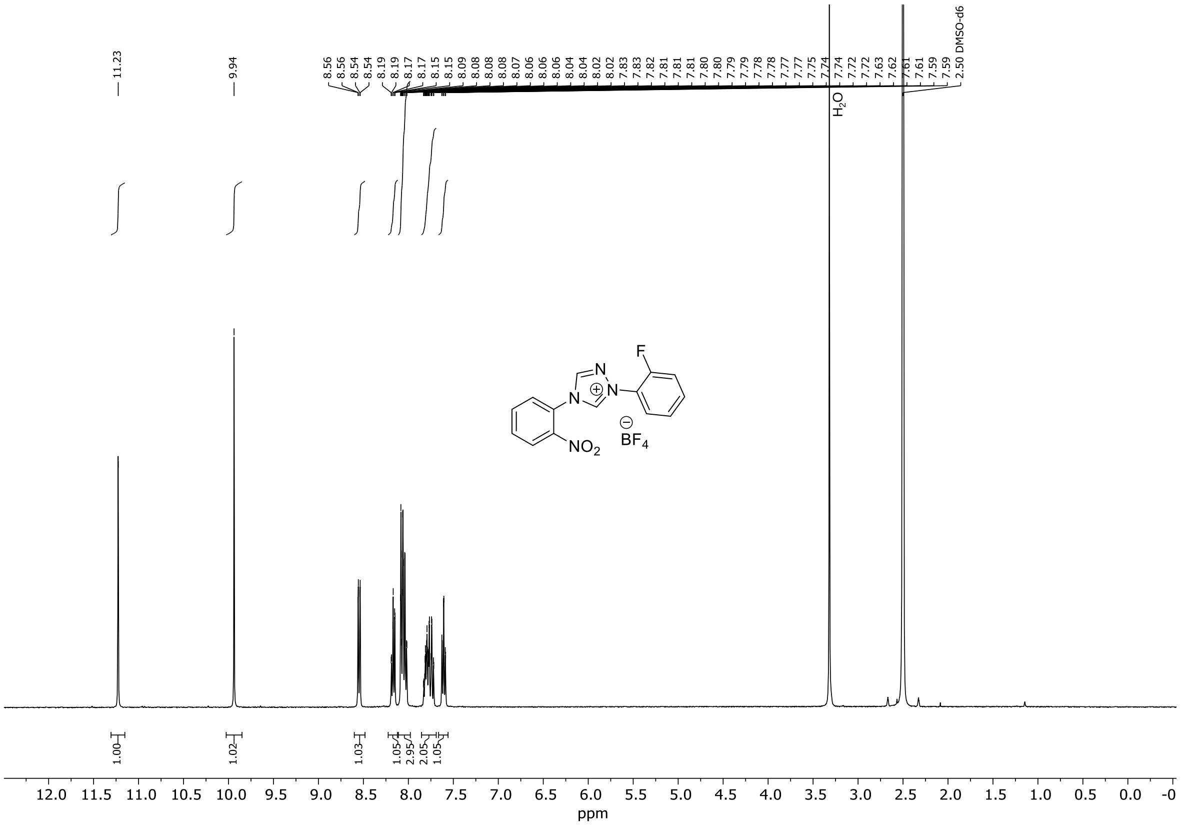

Figure S41: ^{1}H NMR spectrum of 1-(2-fluorophenyl)-4-(2-nitrophenyl)-4*H*-1,2,4-triazol-1-ium tetrafluoroborate (**3h**) (400 MHz, DMSO-d_6, 298 K).

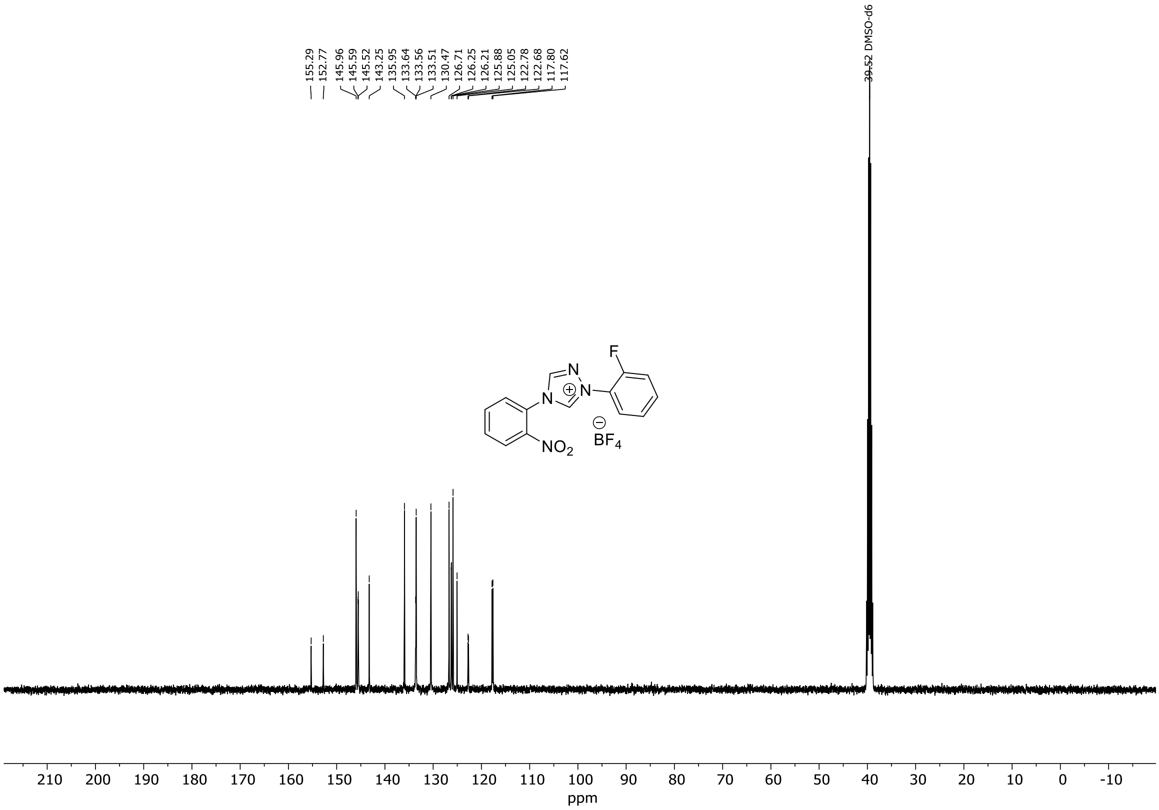

Figure S42: $^{13}C\{^{1}H\}$ NMR spectrum of 1-(2-fluorophenyl)-4-(2-nitrophenyl)-4*H*-1,2,4-triazol-1-ium tetrafluoroborate (**3h**) (100 MHz, DMSO-d_6, 298 K).

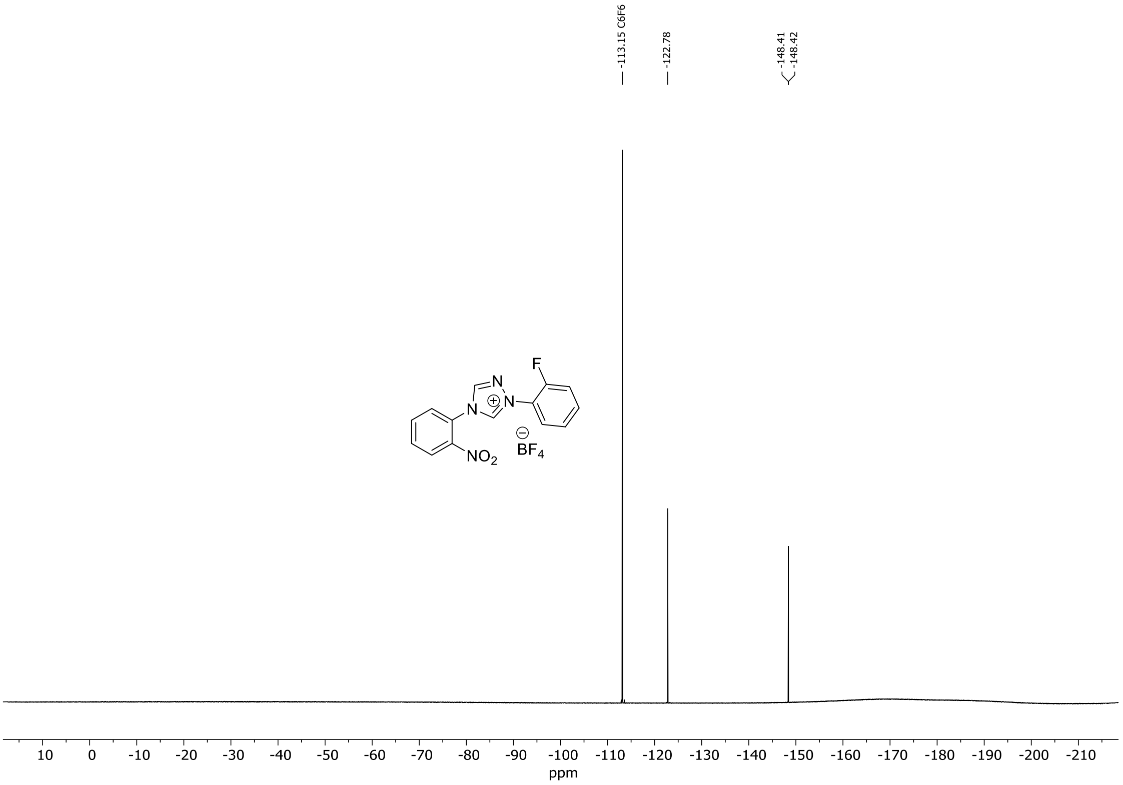

Figure S43: $^{19}F\{^{1}H\}$ NMR spectrum of 1-(2-fluorophenyl)-4-(2-nitrophenyl)-4*H*-1,2,4-triazol-1-ium tetrafluoroborate (**3h**) (376 MHz, DMSO-d_6, 298 K, referenced to fluorobenzene).

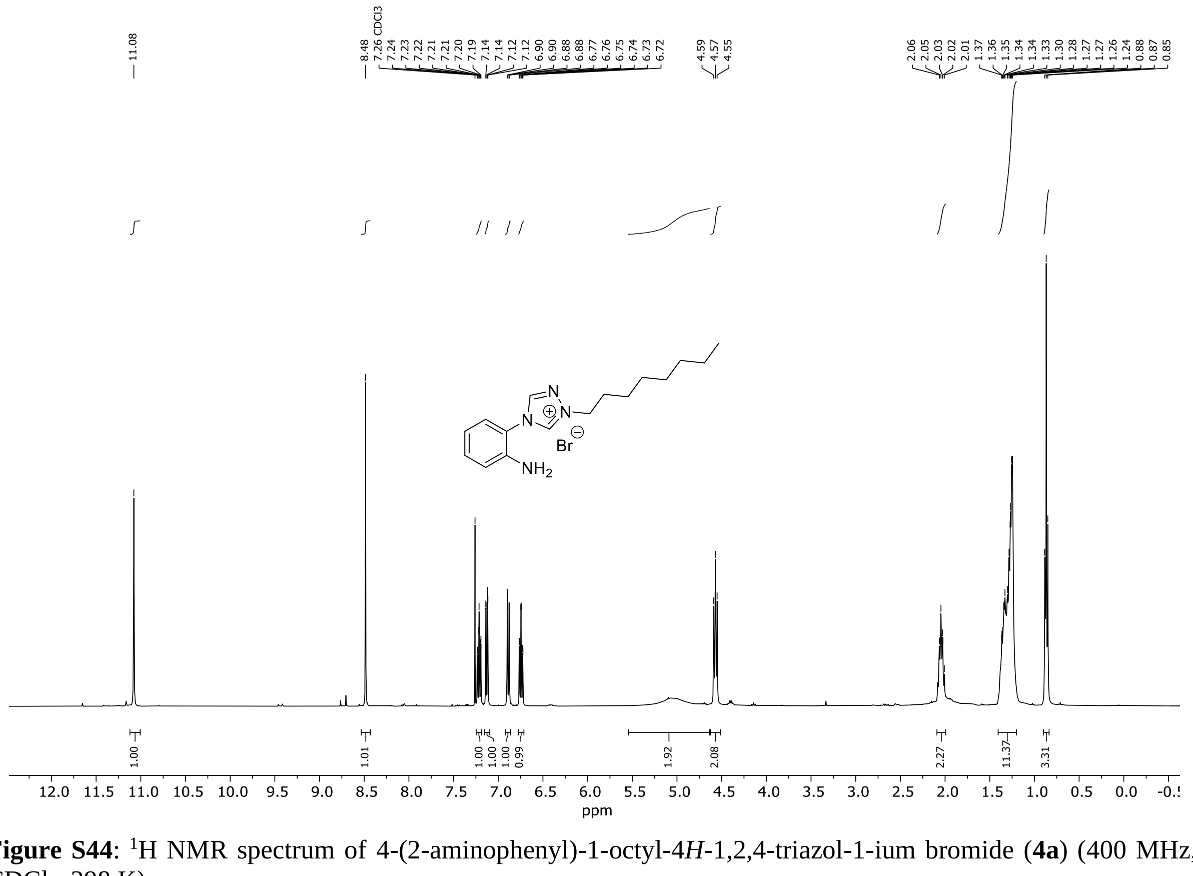

Figure S44: ^{1}H NMR spectrum of 4-(2-aminophenyl)-1-octyl-4*H*-1,2,4-triazol-1-ium bromide (**4a**) (400 MHz, $CDCl_3$, 298 K).

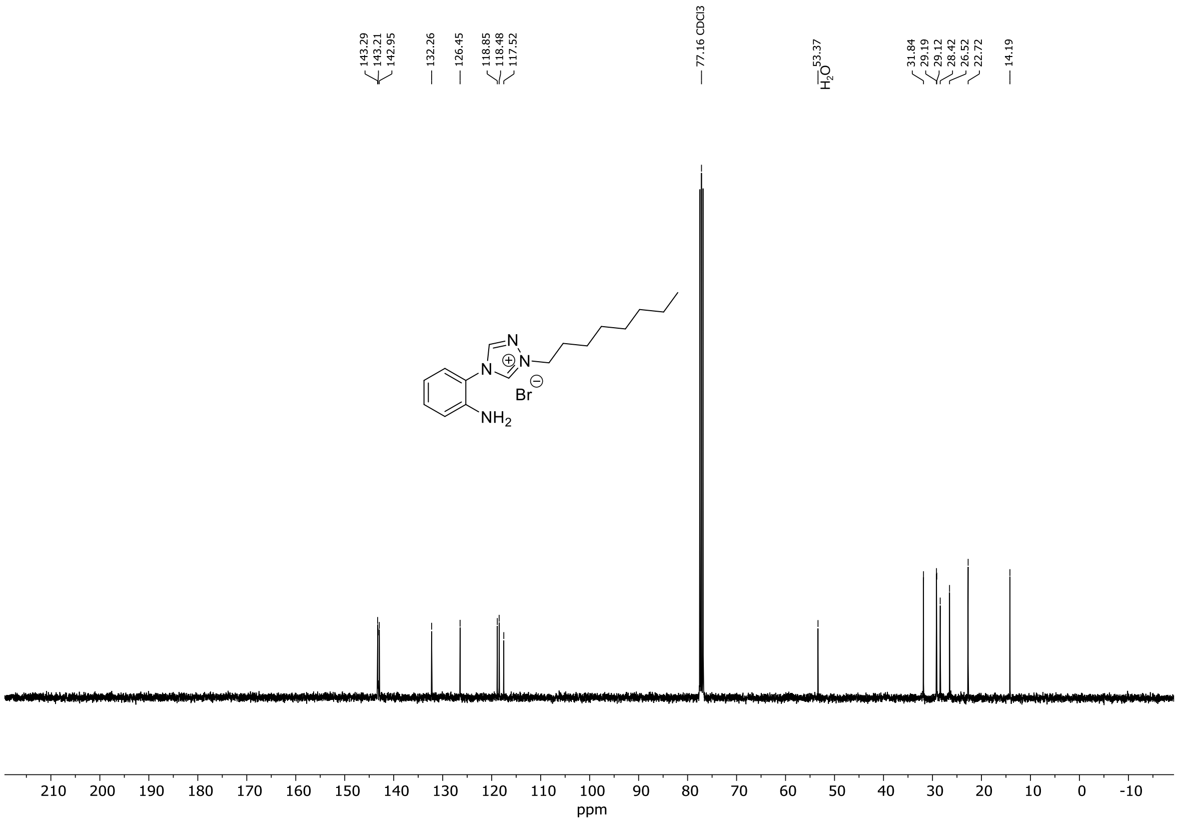

Figure S45: $^{13}C\{^1H\}$ NMR spectrum of 4-(2-aminophenyl)-1-octyl-4*H*-1,2,4-triazol-1-ium bromide (**4a**) (100 MHz, DMSO-d_6, 298 K).

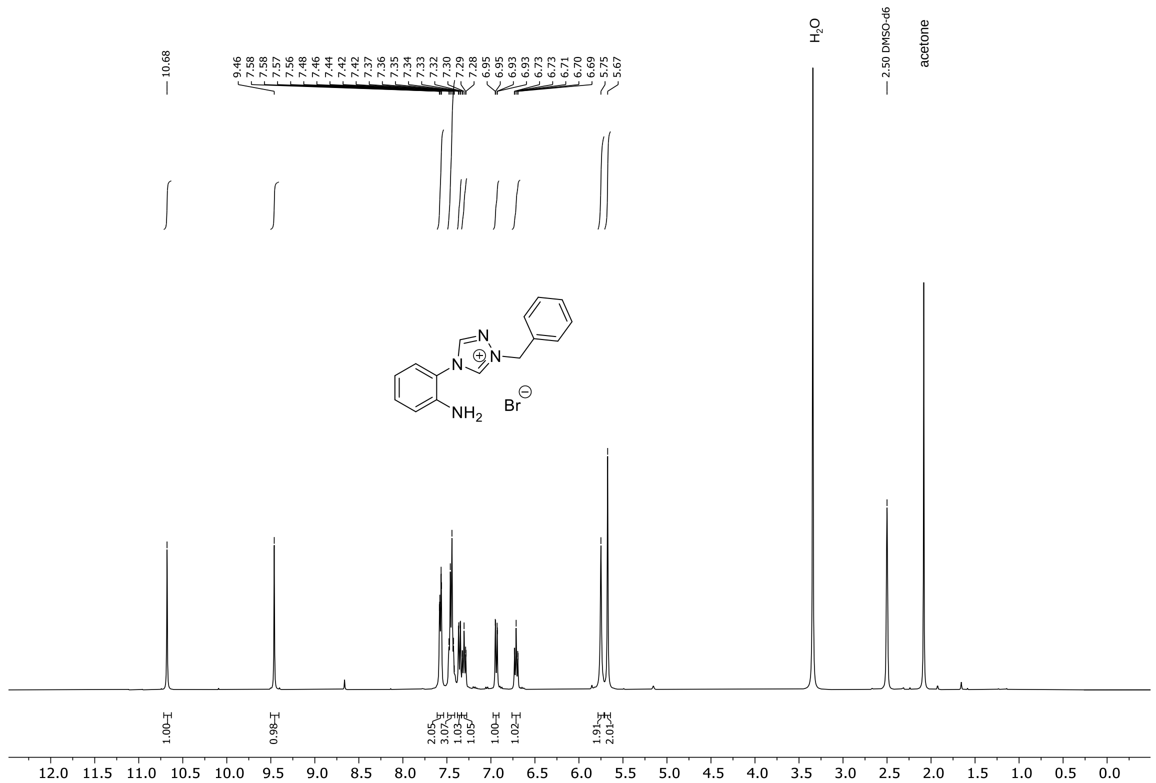

Figure S46: ^1H NMR spectrum of 4-(2-aminophenyl)-1-benzyl-4*H*-1,2,4-triazol-1-ium bromide (**4b**) (400 MHz, $CDCl_3$, 298 K).

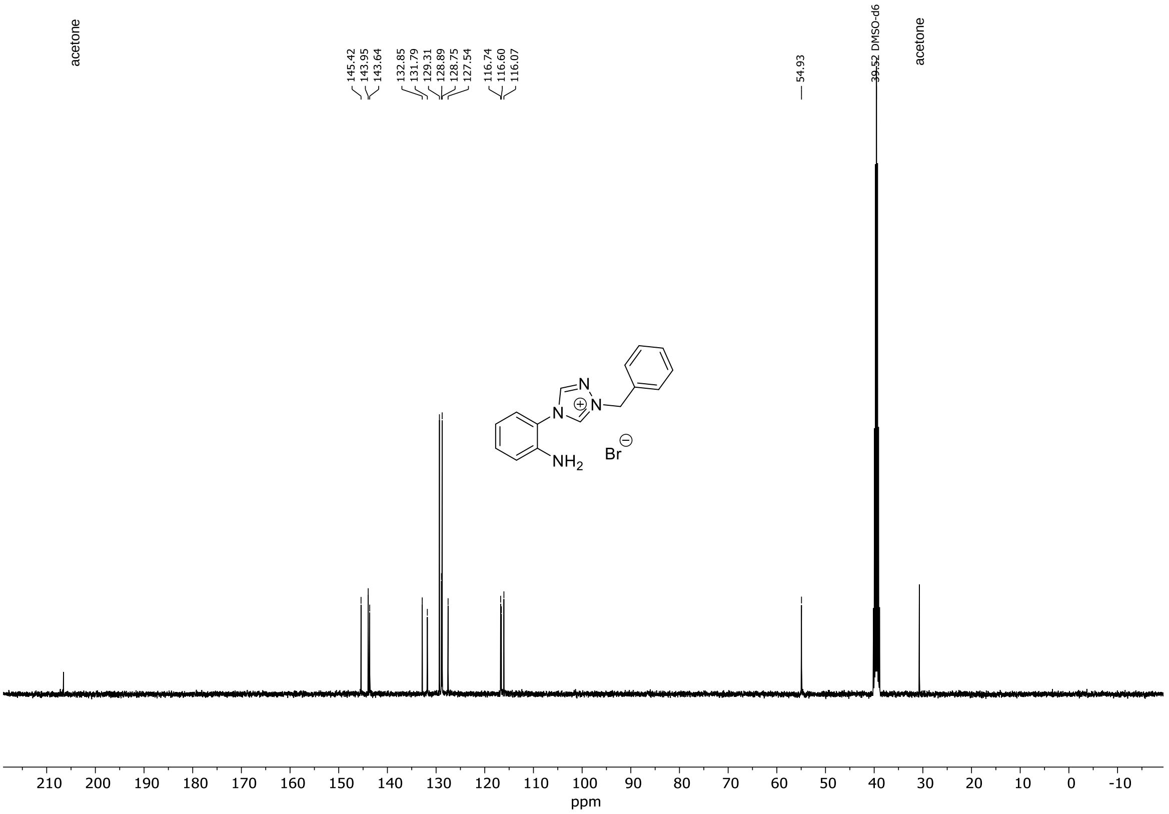

Figure S47: $^{13}C\{^{1}H\}$ NMR spectrum of 4-(2-aminophenyl)-1-benzyl-4*H*-1,2,4-triazol-1-ium bromide (**4b**) (100 MHz, DMSO-d_6, 298 K).

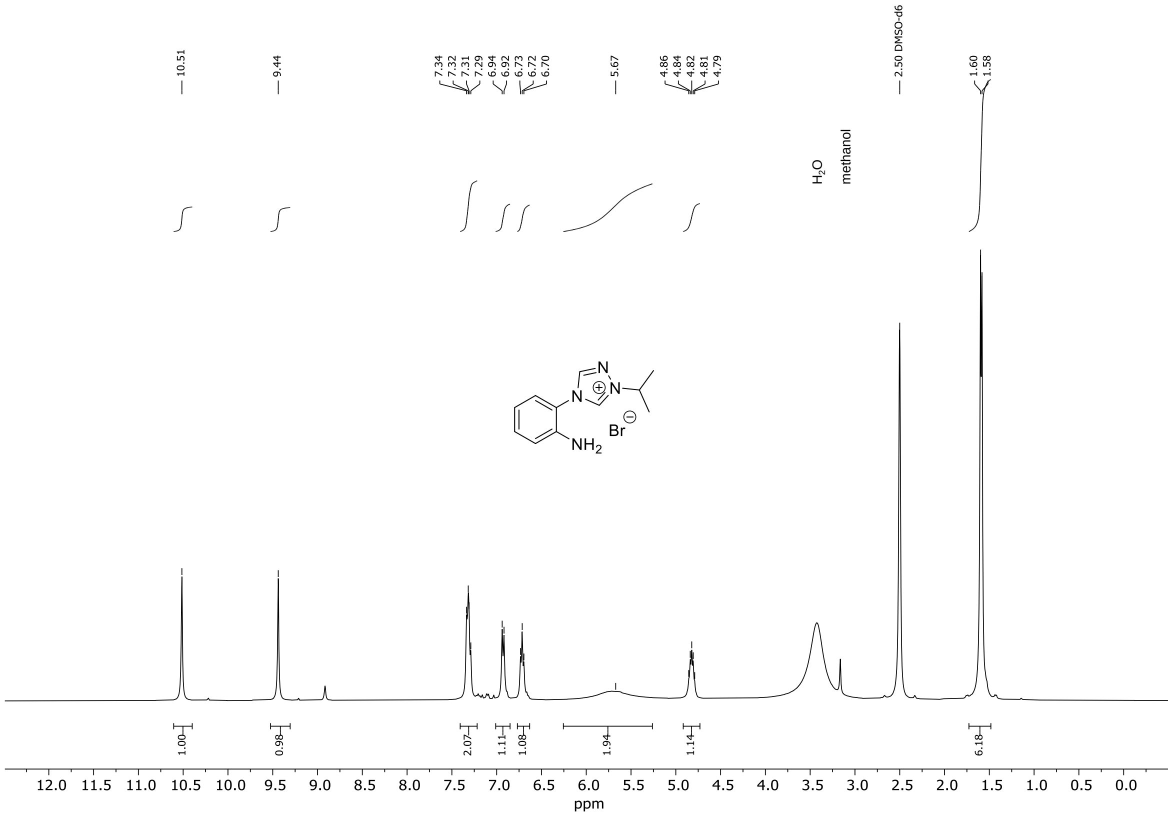

Figure S48: ^{1}H NMR spectrum of 4-(2-aminophenyl)-1-isopropyl-4*H*-1,2,4-triazol-1-ium bromide (**4c**) (400 MHz, DMSO-d_6, 298 K).

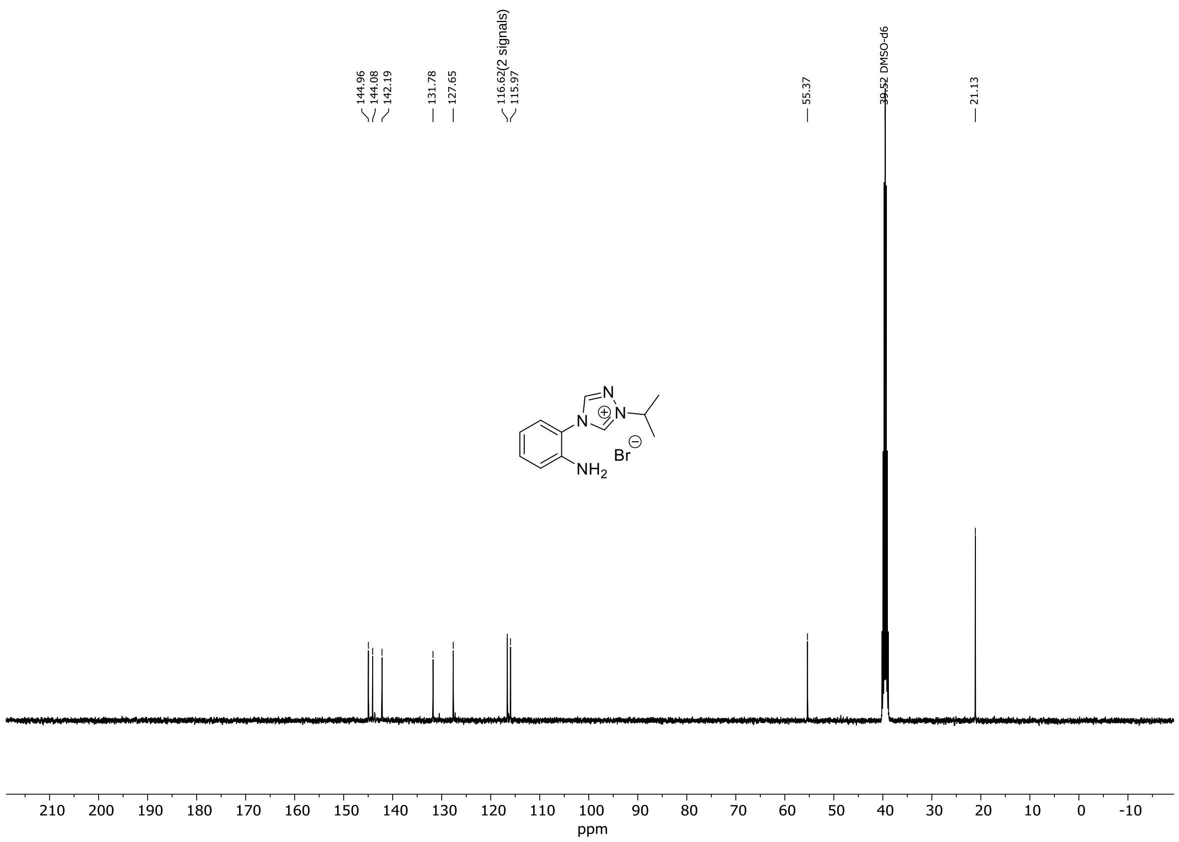

Figure S49: $^{13}C\{^1H\}$ NMR spectrum of 4-(2-aminophenyl)-1-isopropyl-4*H*-1,2,4-triazol-1-ium bromide (**4c**) (100 MHz, DMSO-d_6, 298 K).

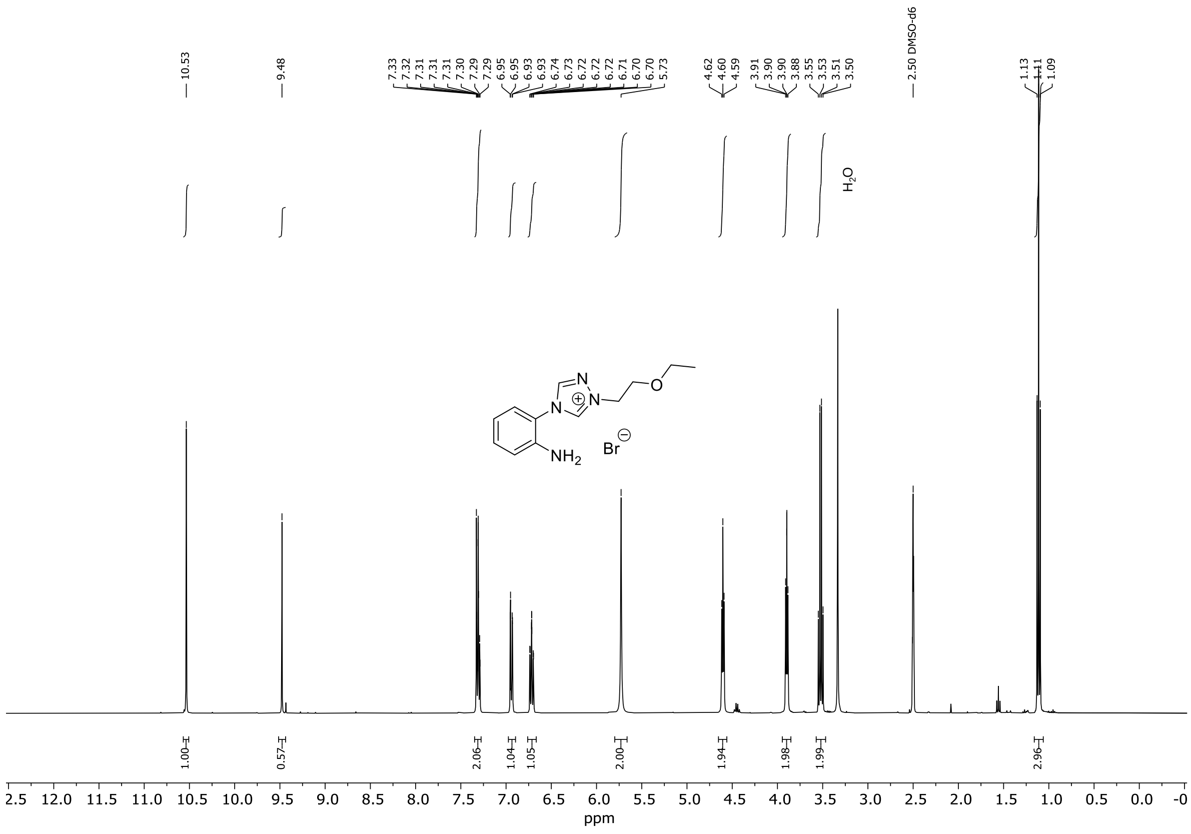

Figure S50: ^1H NMR spectrum of 4-(2-aminophenyl)-1-(2-ethoxyethyl)-4*H*-1,2,4-triazol-1-ium bromide (**4d**) (400 MHz, DMSO-d_6, 298 K).

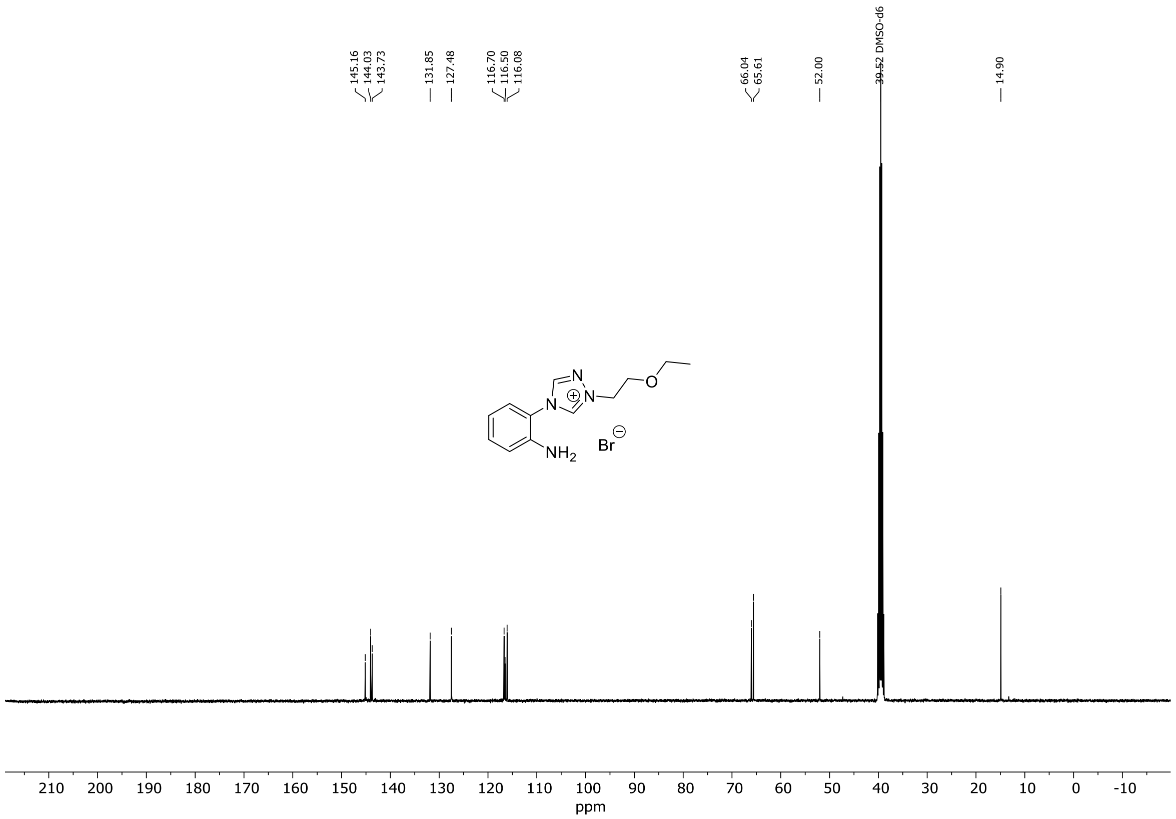

Figure S51: $^{13}C\{^1H\}$ NMR spectrum of 4-(2-aminophenyl)-1-(2-ethoxyethyl)-4*H*-1,2,4-triazol-1-ium bromide (**4d**) (100 MHz, DMSO-d_6, 298 K).

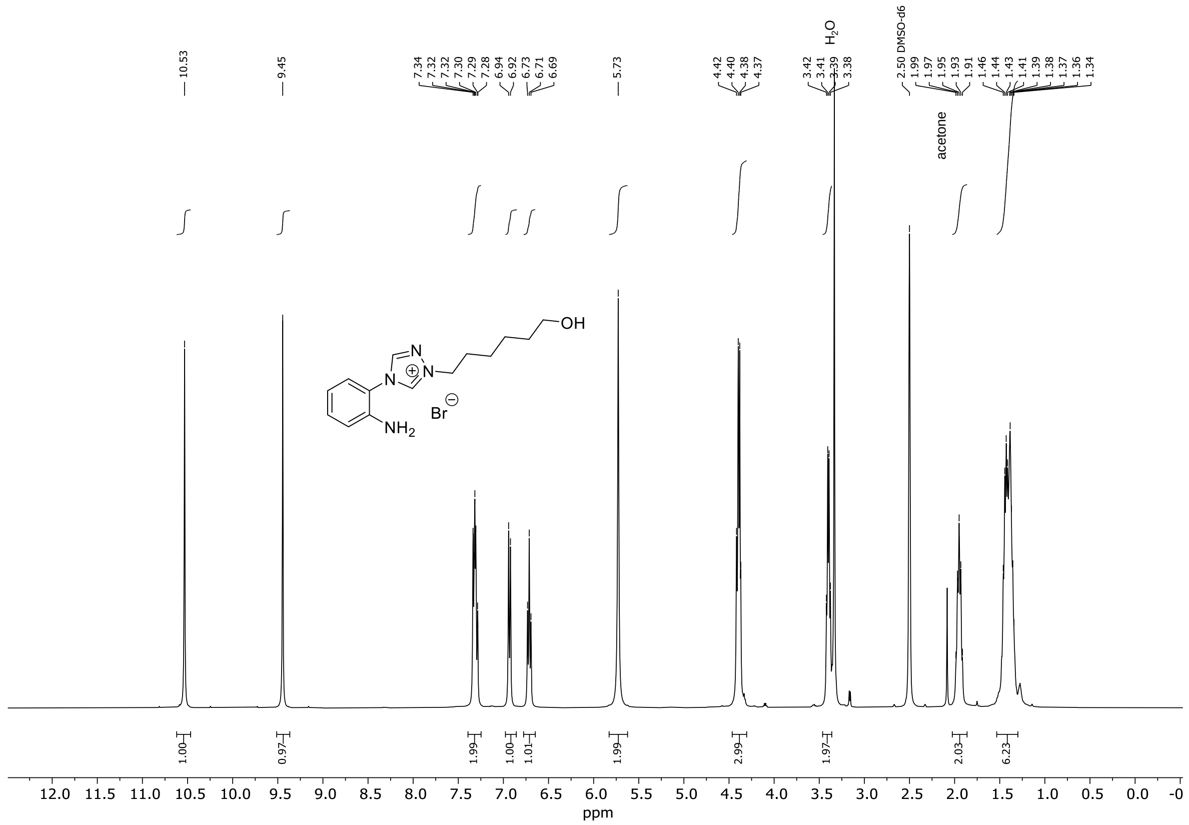

Figure S52: ^1H NMR spectrum of 4-(2-aminophenyl)-1-(6-hydroxyhexyl)-4*H*-1,2,4-triazol-1-ium bromide (**4e**) (400 MHz, DMSO-d_6, 298 K).

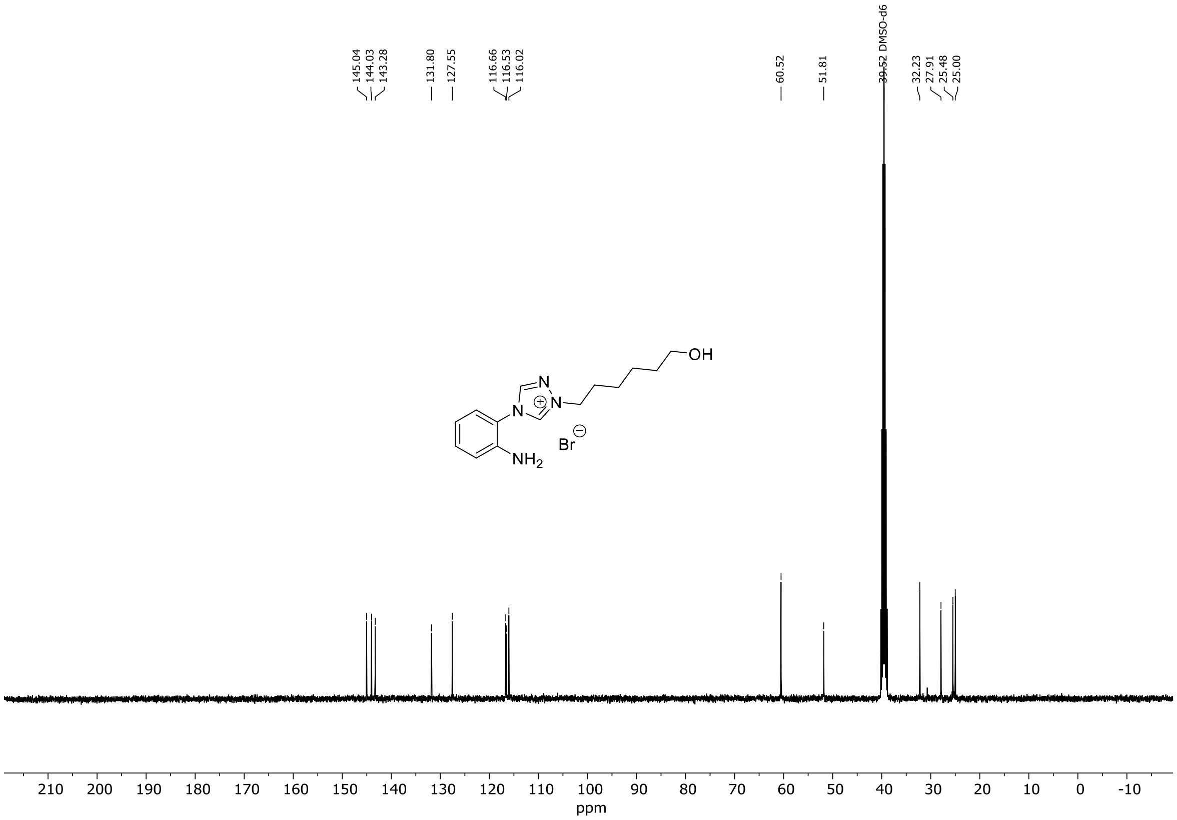

Figure S53: $^{13}C\{^{1}H\}$ NMR spectrum of 4-(2-aminophenyl)-1-(6-hydroxyhexyl)-4*H*-1,2,4-triazol-1-ium bromide (**4e**) (100 MHz, DMSO-d_6, 298 K).

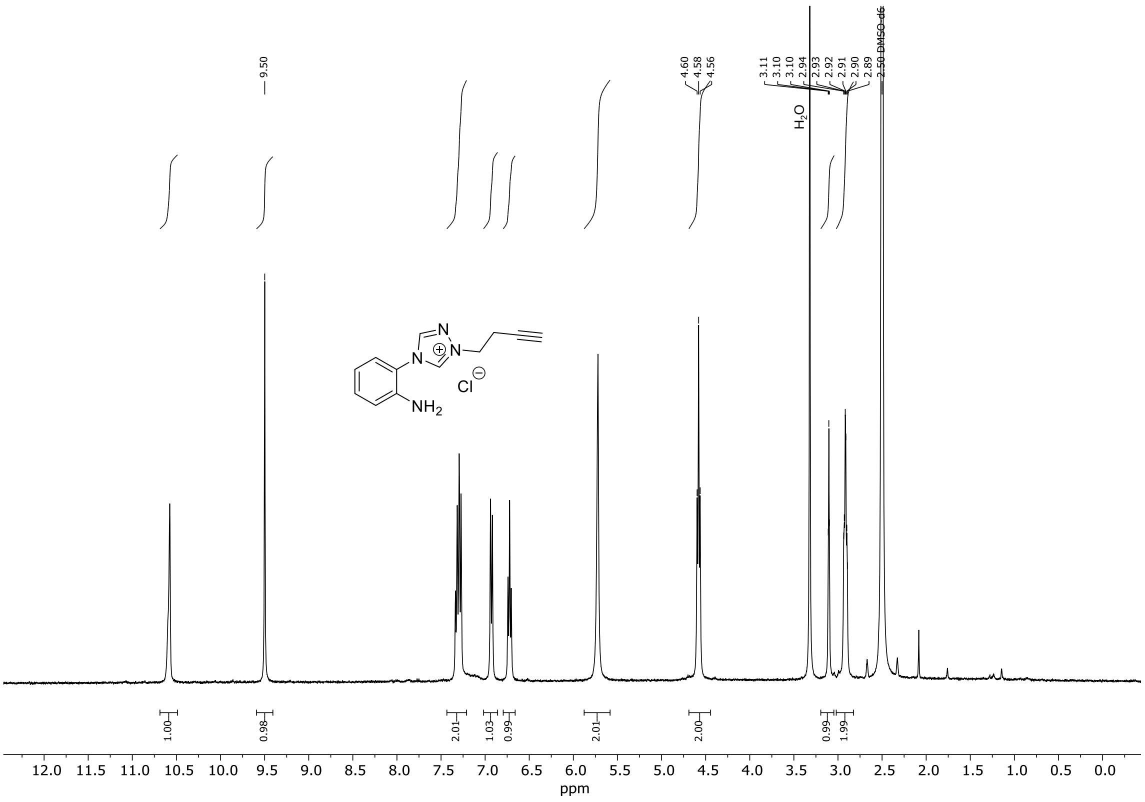

Figure S54: ^{1}H NMR spectrum of 4-(2-aminophenyl)-1-(but-3-yn-1-yl)-4*H*-1,2,4-triazol-1-ium chloride (**4f**) (400 MHz, DMSO-d_6, 298 K).

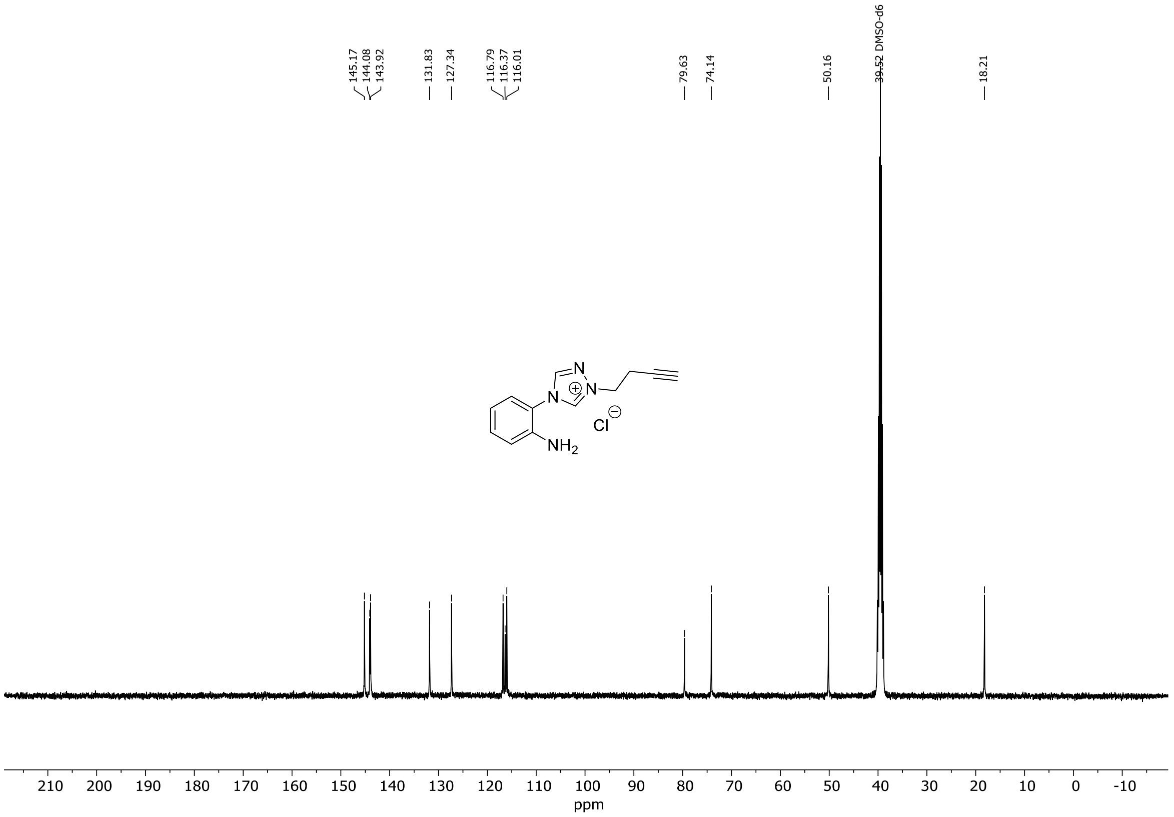

Figure S55: $^{13}C\{^{1}H\}$ NMR spectrum of 4-(2-aminophenyl)-1-(but-3-yn-1-yl)-4*H*-1,2,4-triazol-1-ium chloride (**4f**) (100 MHz, DMSO-d_6, 298 K).

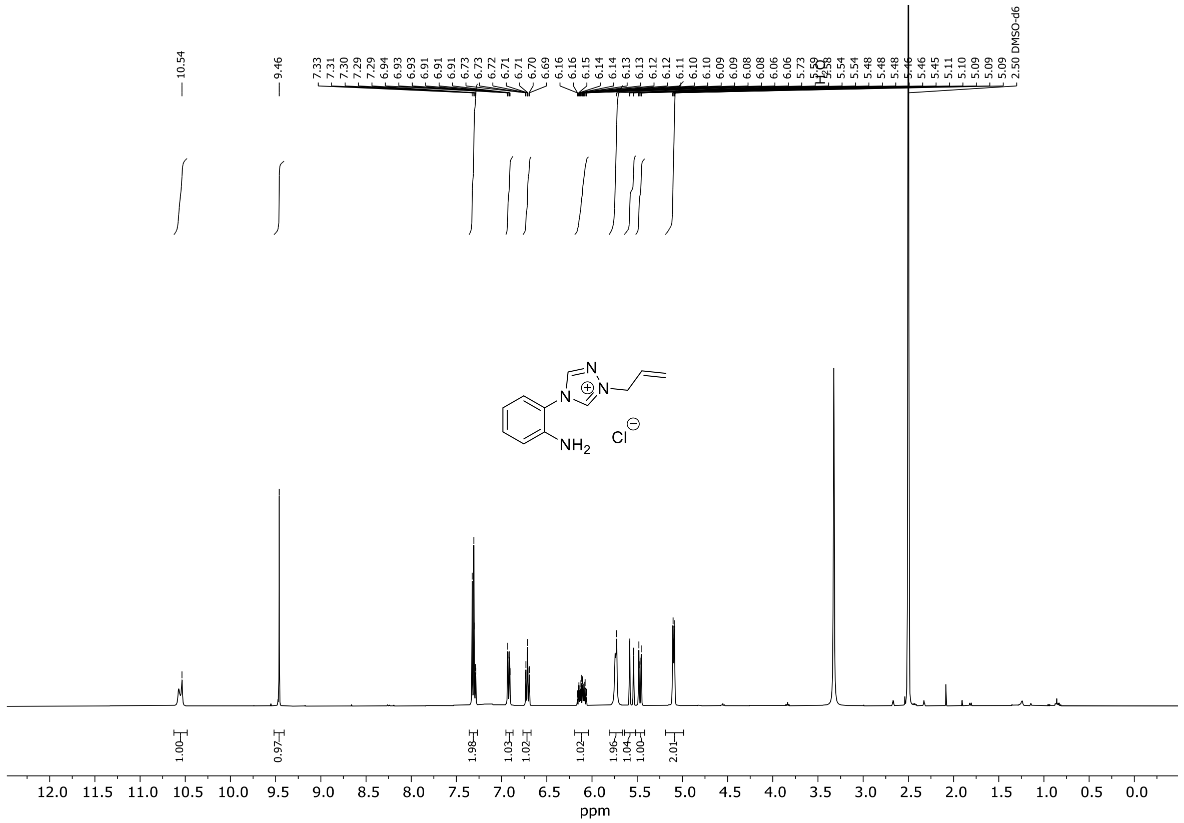

Figure S56: ^{1}H NMR spectrum of 1-allyl-4-(2-aminophenyl)-4*H*-1,2,4-triazol-1-ium chloride (**4g**) (400 MHz, DMSO-d_6, 298 K).

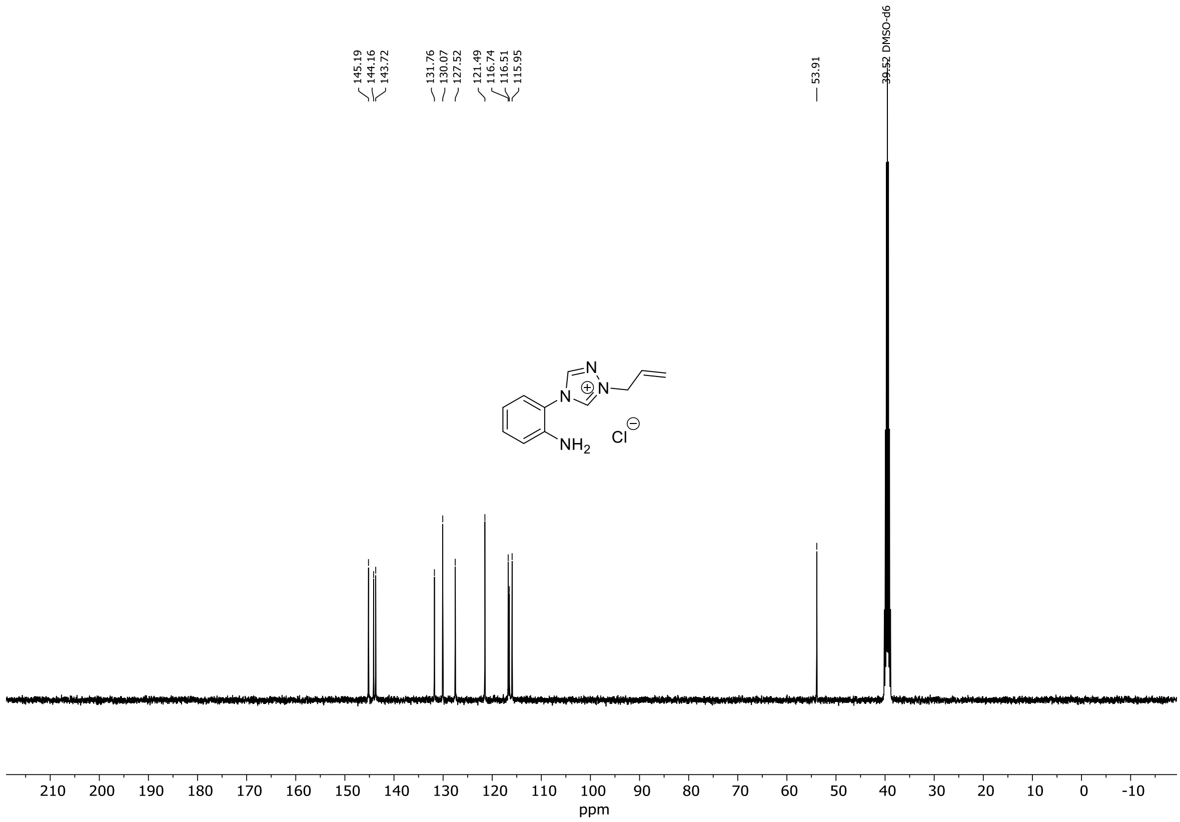

Figure S57: $^{13}C\{^{1}H\}$ NMR spectrum of 1-allyl-4-(2-aminophenyl)-4*H*-1,2,4-triazol-1-ium chloride (**4g**) (100 MHz, DMSO-d_6, 298 K).

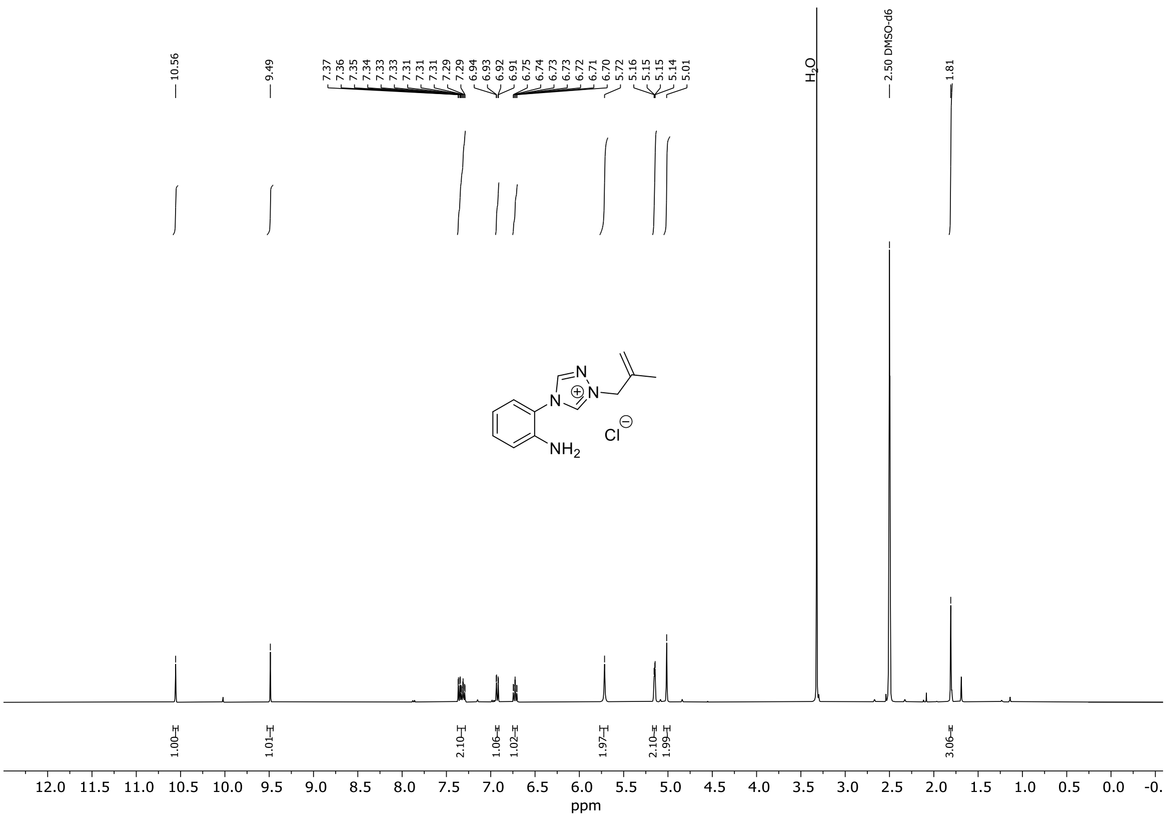

Figure S58: ^{1}H NMR spectrum of 4-(2-aminophenyl)-1-(2-methylallyl)-4*H*-1,2,4-triazol-1-ium chloride (**4h**) (400 MHz, DMSO-d_6, 298 K).

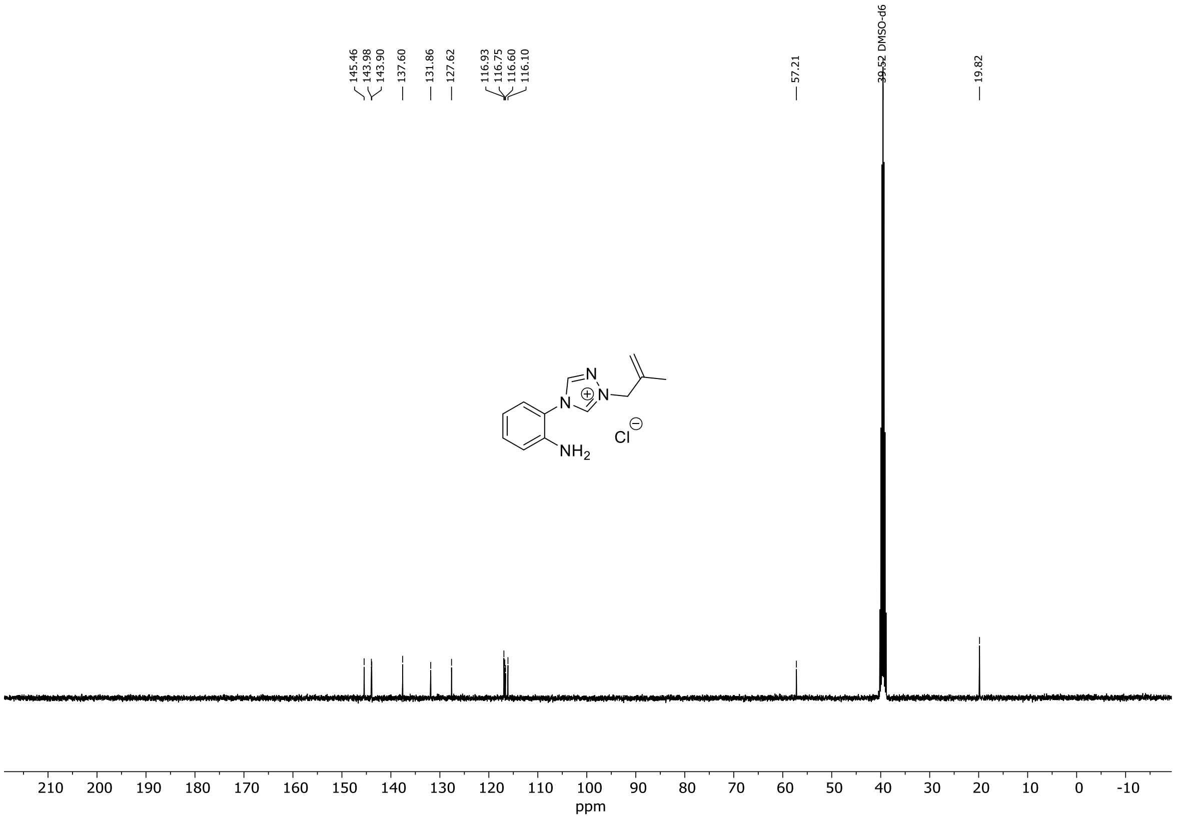

Figure S59: $^{13}C\{^{1}H\}$ NMR spectrum of 4-(2-aminophenyl)-1-(2-methylallyl)-4*H*-1,2,4-triazol-1-ium chloride (**4h**) (100 MHz, DMSO-d_6, 298 K).

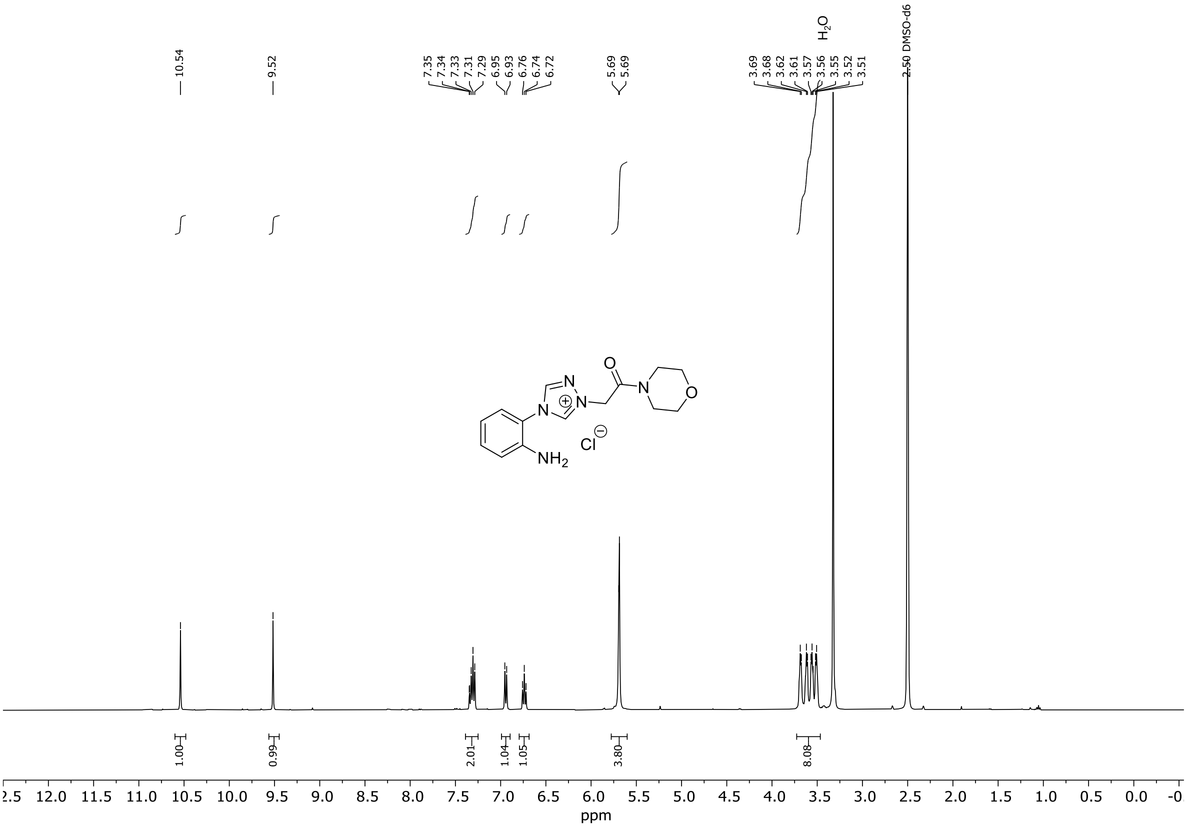

Figure S60: ^{1}H NMR spectrum of 4-(2-aminophenyl)-1-(2-morpholino-2-oxoethyl)-4*H*-1,2,4-triazol-1-ium chloride (**4i**) (400 MHz, DMSO-d_6, 298 K).

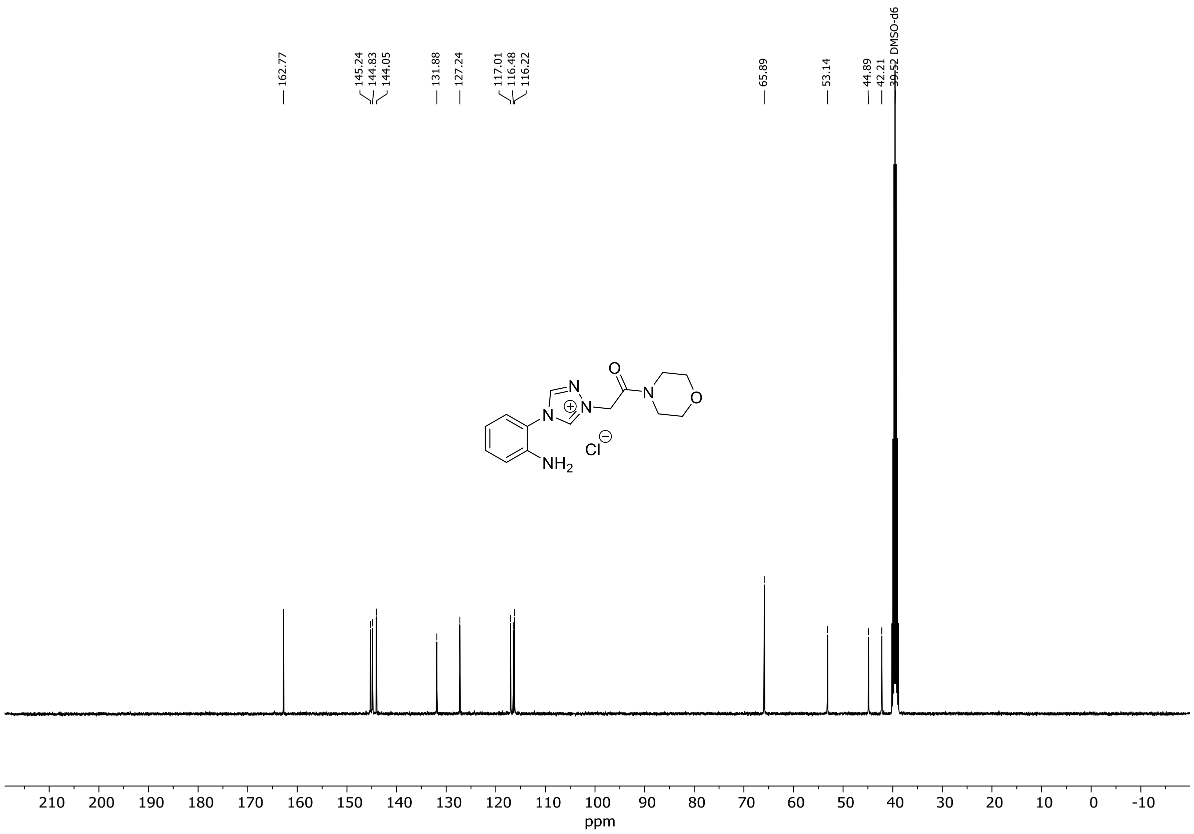

Figure S61: $^{13}C\{^{1}H\}$ NMR spectrum of 4-(2-aminophenyl)-1-(2-morpholino-2-oxoethyl)-4*H*-1,2,4-triazol-1-ium chloride (**4i**) (100 MHz, DMSO-d_6, 298 K).

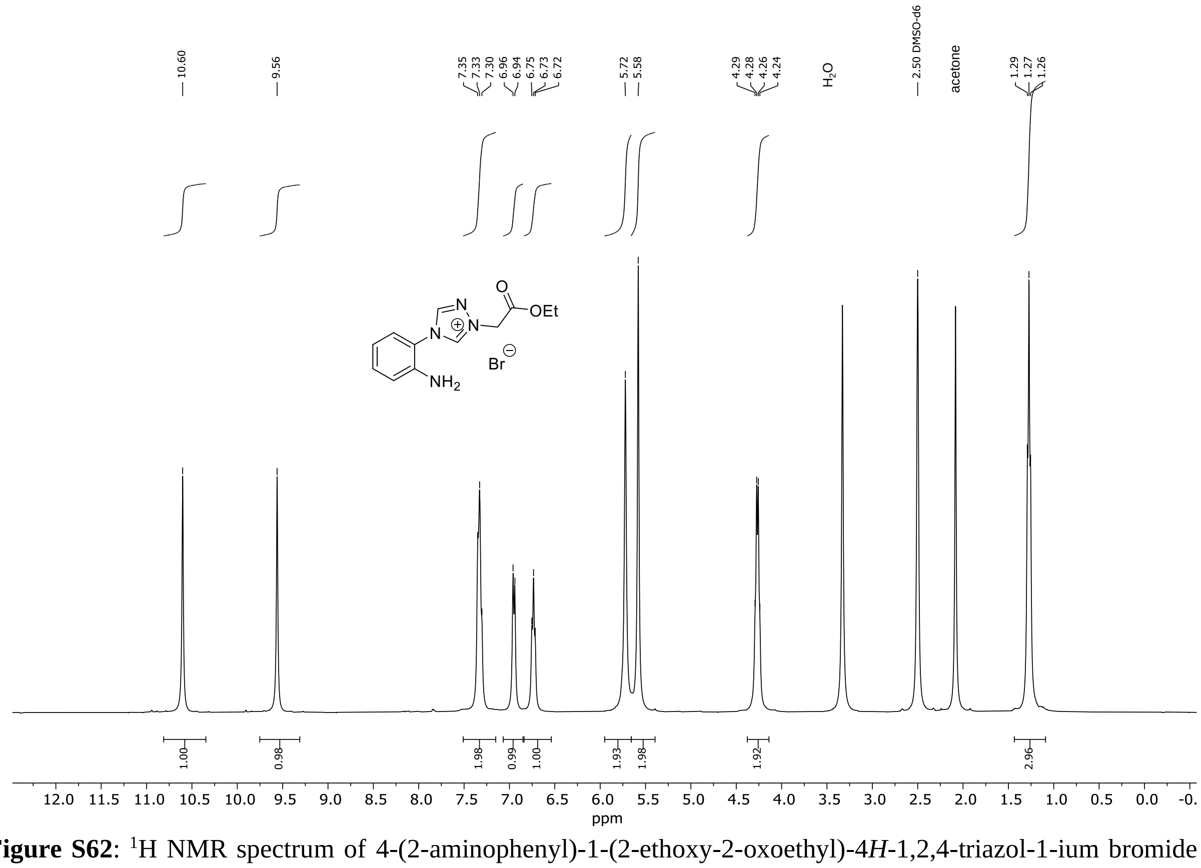

Figure S62: ^{1}H NMR spectrum of 4-(2-aminophenyl)-1-(2-ethoxy-2-oxoethyl)-4*H*-1,2,4-triazol-1-ium bromide (**4j**) (400 MHz, DMSO-d_6, 298 K).

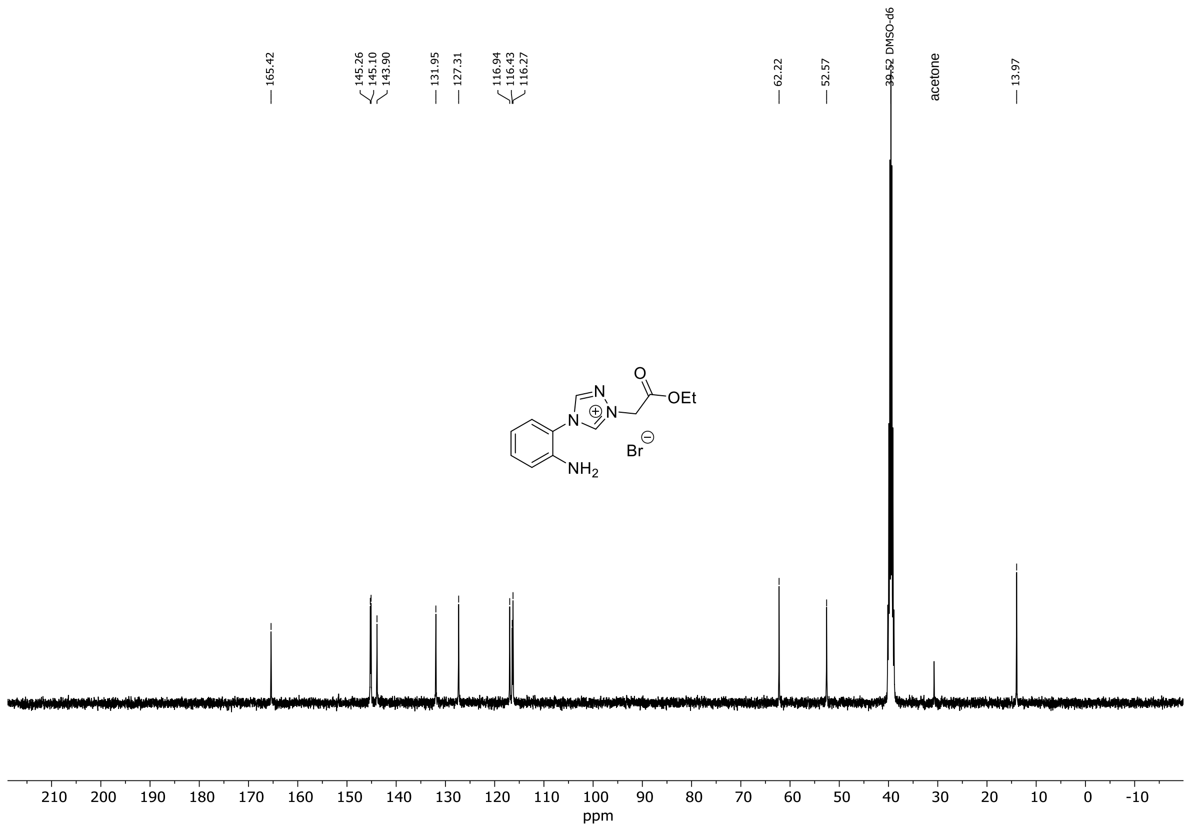

Figure S63: $^{13}C\{^1H\}$ NMR spectrum of 4-(2-aminophenyl)-1-(2-ethoxy-2-oxoethyl)-4*H*-1,2,4-triazol-1-ium bromide (**4j**) (100 MHz, DMSO-d_6, 298 K).

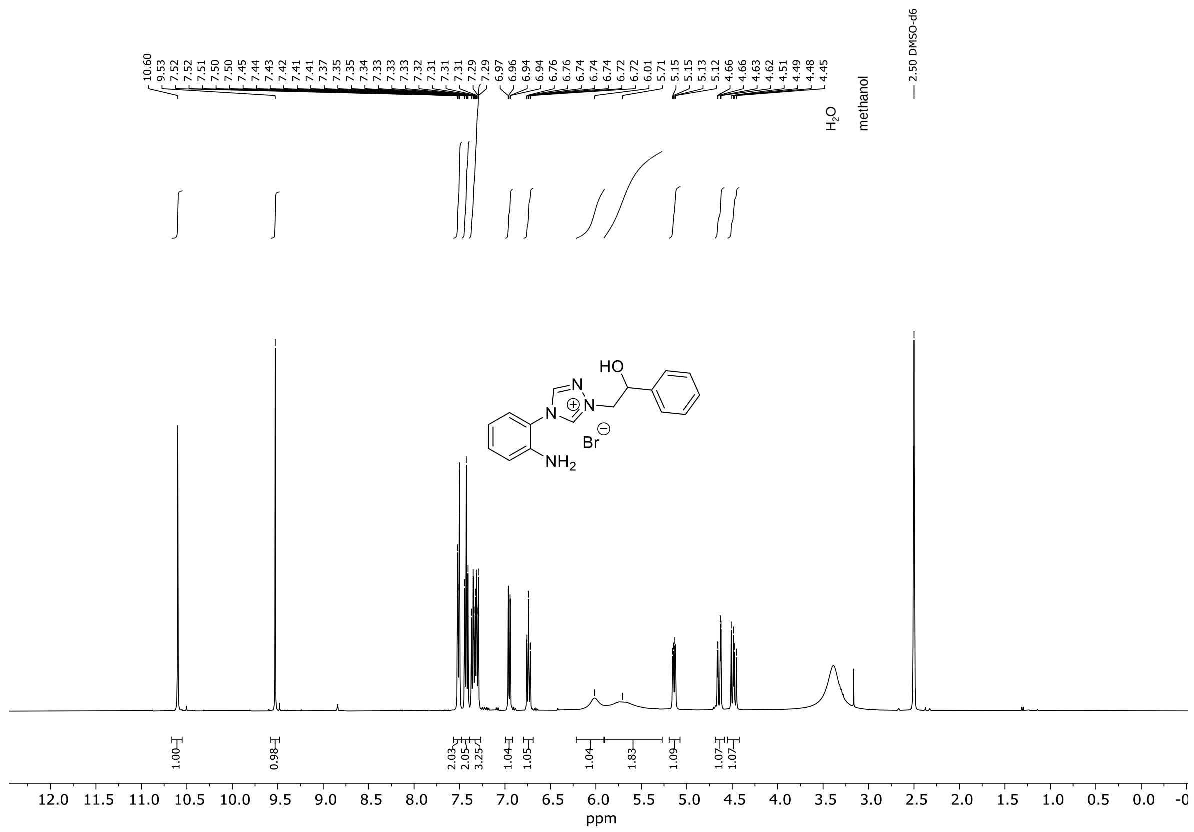

Figure S64: ^1H NMR spectrum of 4-(2-aminophenyl)-1-(2-hydroxy-2-phenylethyl)-4*H*-1,2,4-triazol-1-ium bromide (**4k'**) (400 MHz, DMSO-d_6, 298 K).

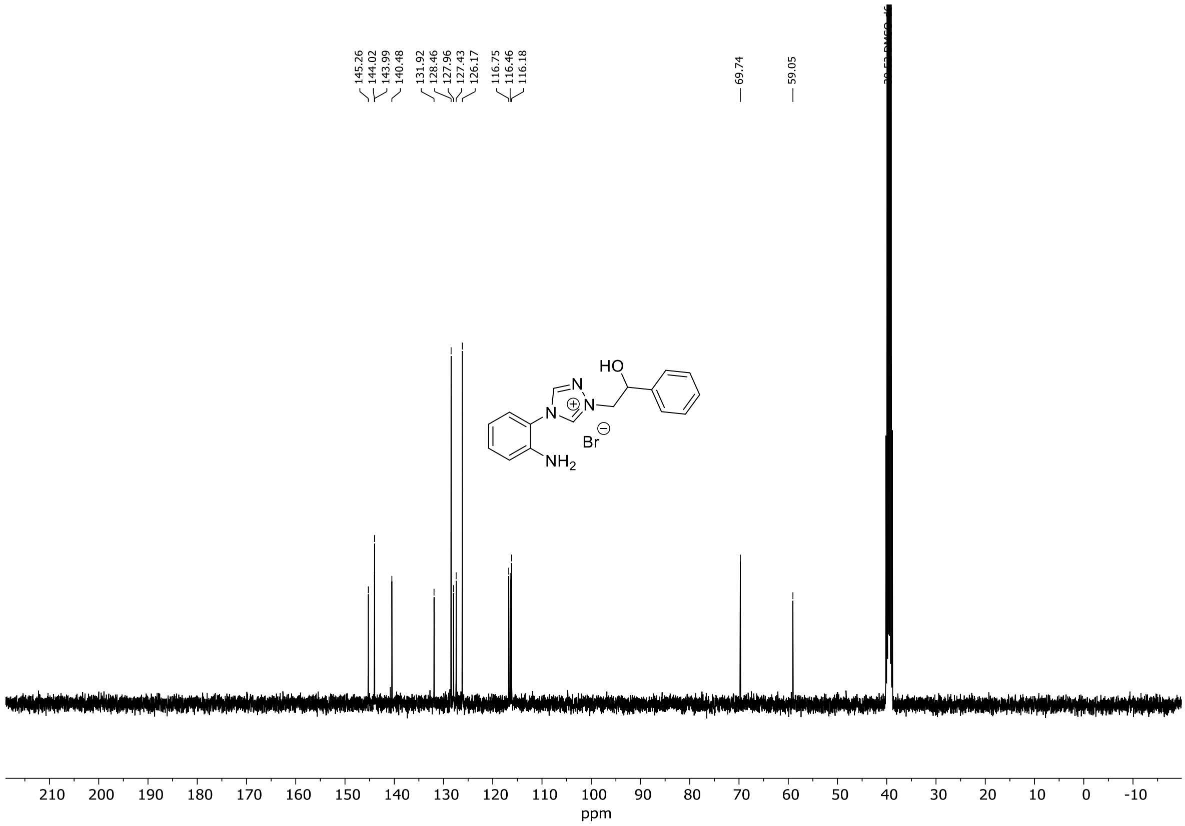

Figure S65: $^{13}C\{^1H\}$ NMR spectrum of 4-(2-aminophenyl)-1-(2-hydroxy-2-phenylethyl)-4*H*-1,2,4-triazol-1-ium bromide (**4k'**) (100 MHz, DMSO-d_6, 298 K).

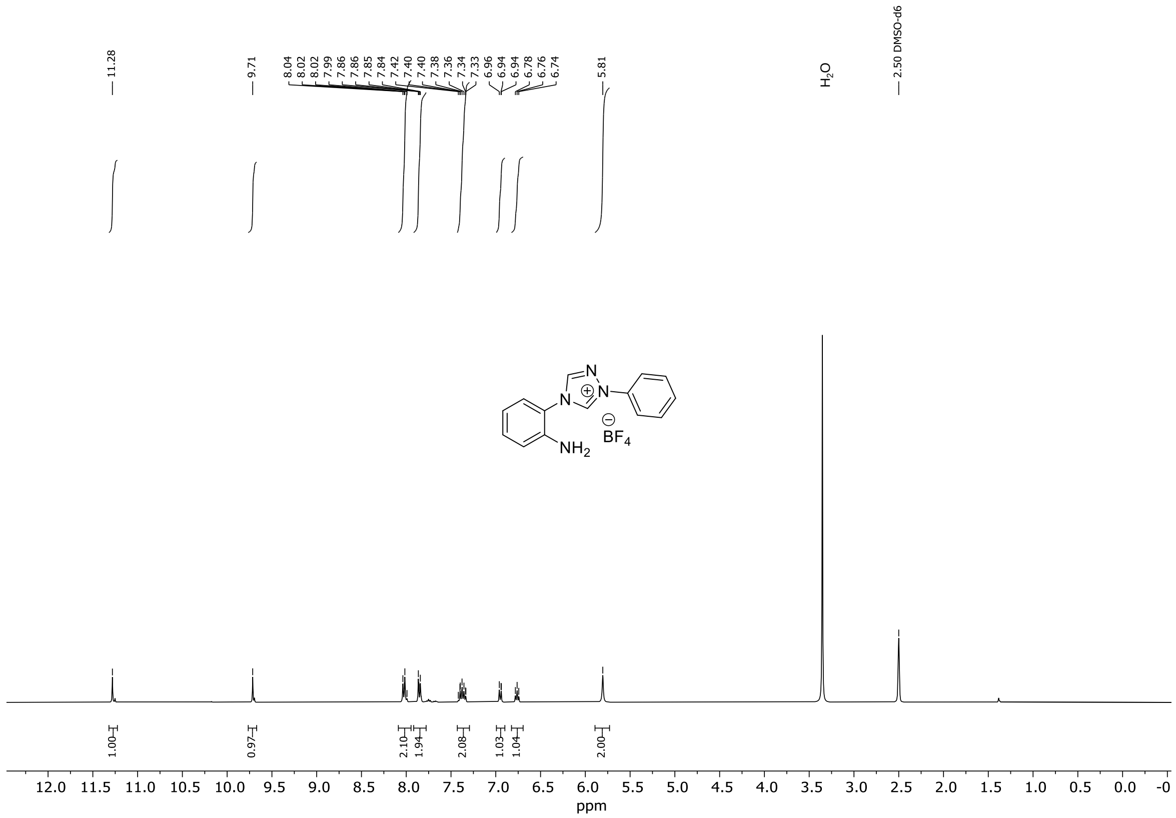

Figure S66: ^1H NMR spectrum of 4-(2-aminophenyl)-1-phenyl-4*H*-1,2,4-triazol-1-ium tetrafluoroborate (**5a**) (400 MHz, DMSO-d_6, 298 K).

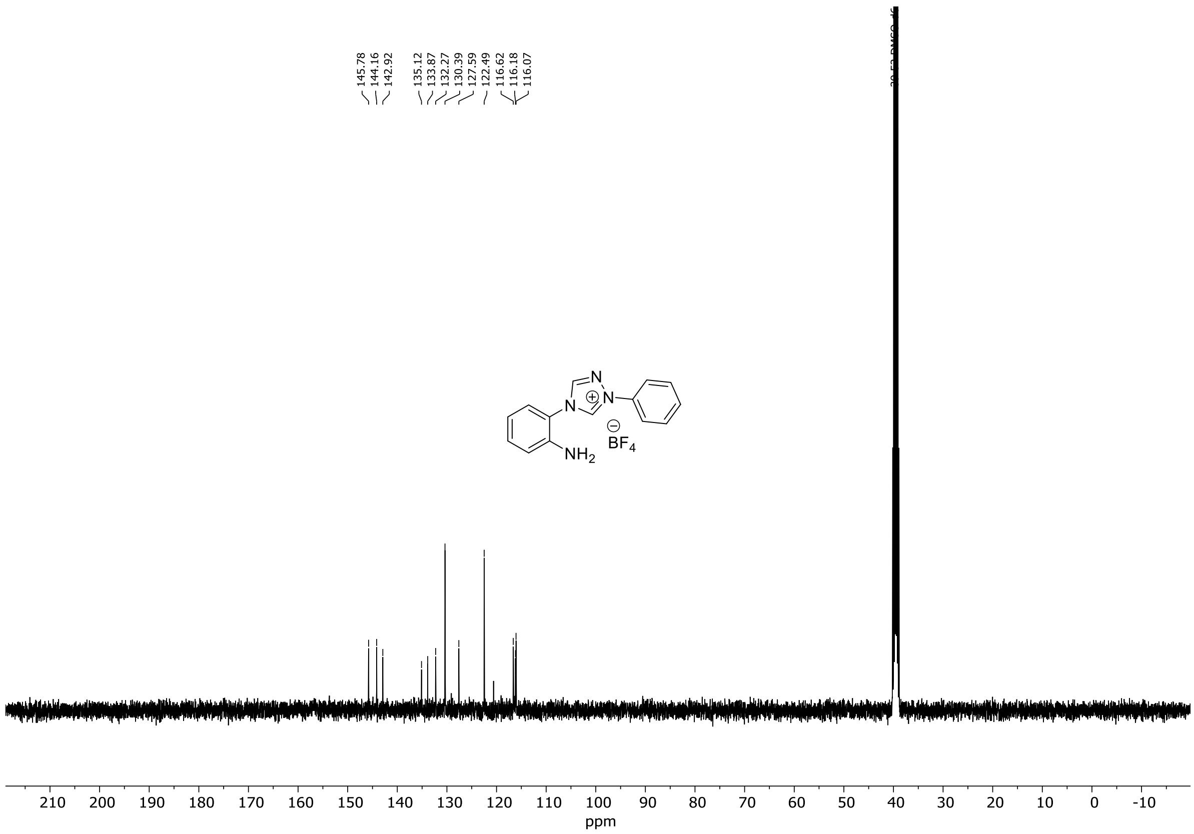

Figure S67: $^{13}C\{^{1}H\}$ NMR spectrum of 4-(2-aminophenyl)-1-phenyl-4*H*-1,2,4-triazol-1-ium tetrafluoroborate (**5a**) (100 MHz, DMSO-d_6, 298 K).

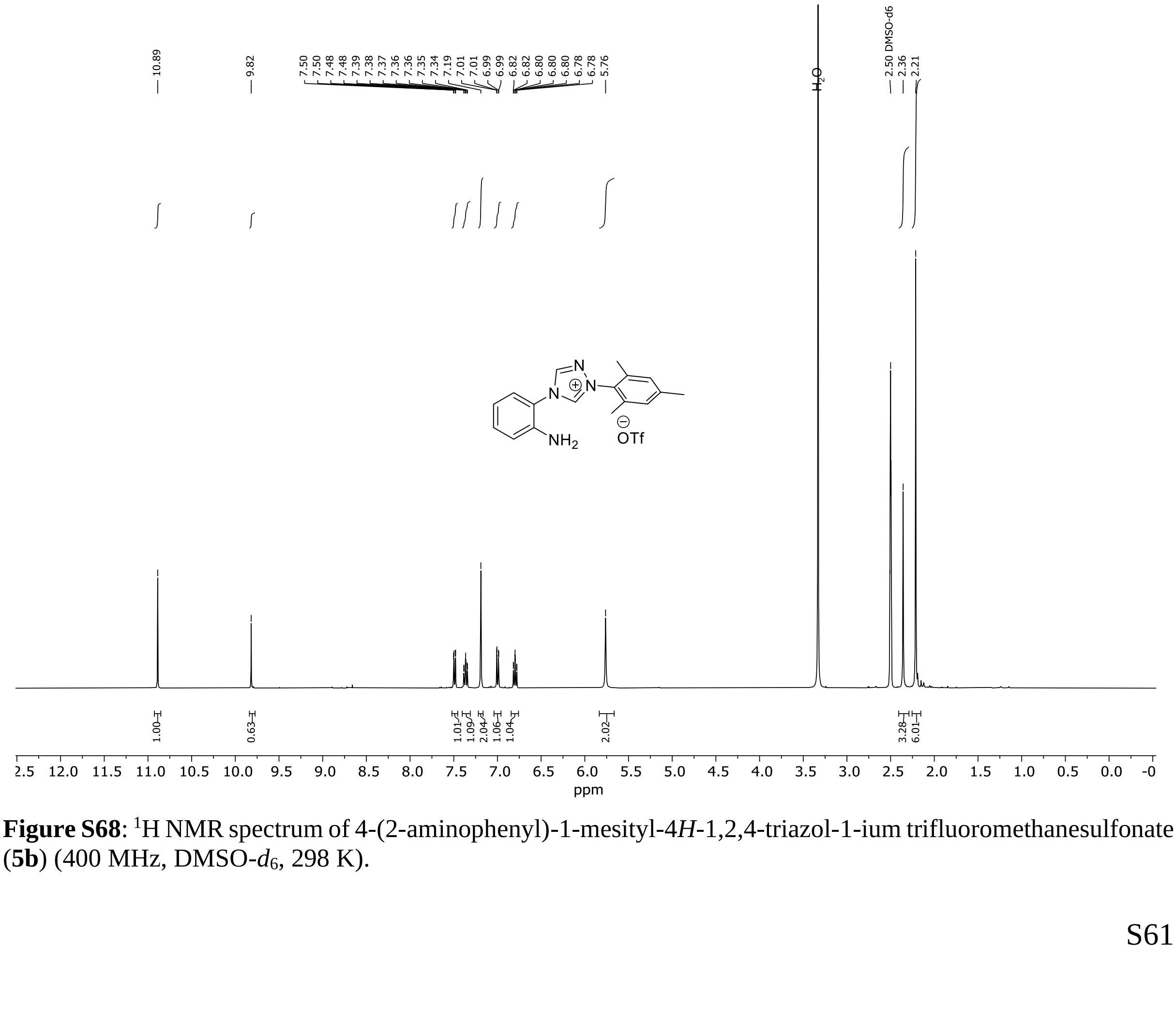

Figure S68: ^{1}H NMR spectrum of 4-(2-aminophenyl)-1-mesityl-4*H*-1,2,4-triazol-1-ium trifluoromethanesulfonate (**5b**) (400 MHz, DMSO-d_6, 298 K).

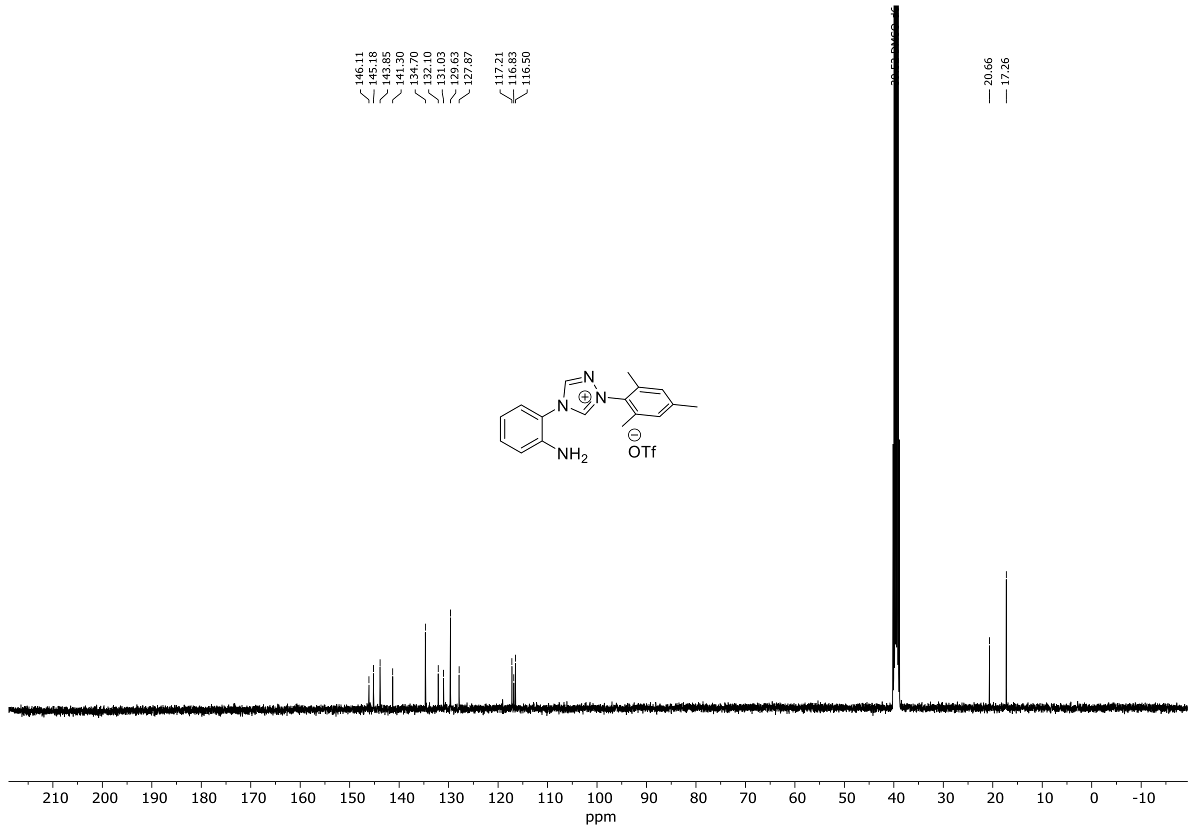

Figure S69: $^{13}C\{^{1}H\}$ NMR spectrum of 4-(2-aminophenyl)-1-mesityl-4*H*-1,2,4-triazol-1-ium trifluoromethanesulfonate (**5b**) (100 MHz, DMSO-d_6, 298 K).

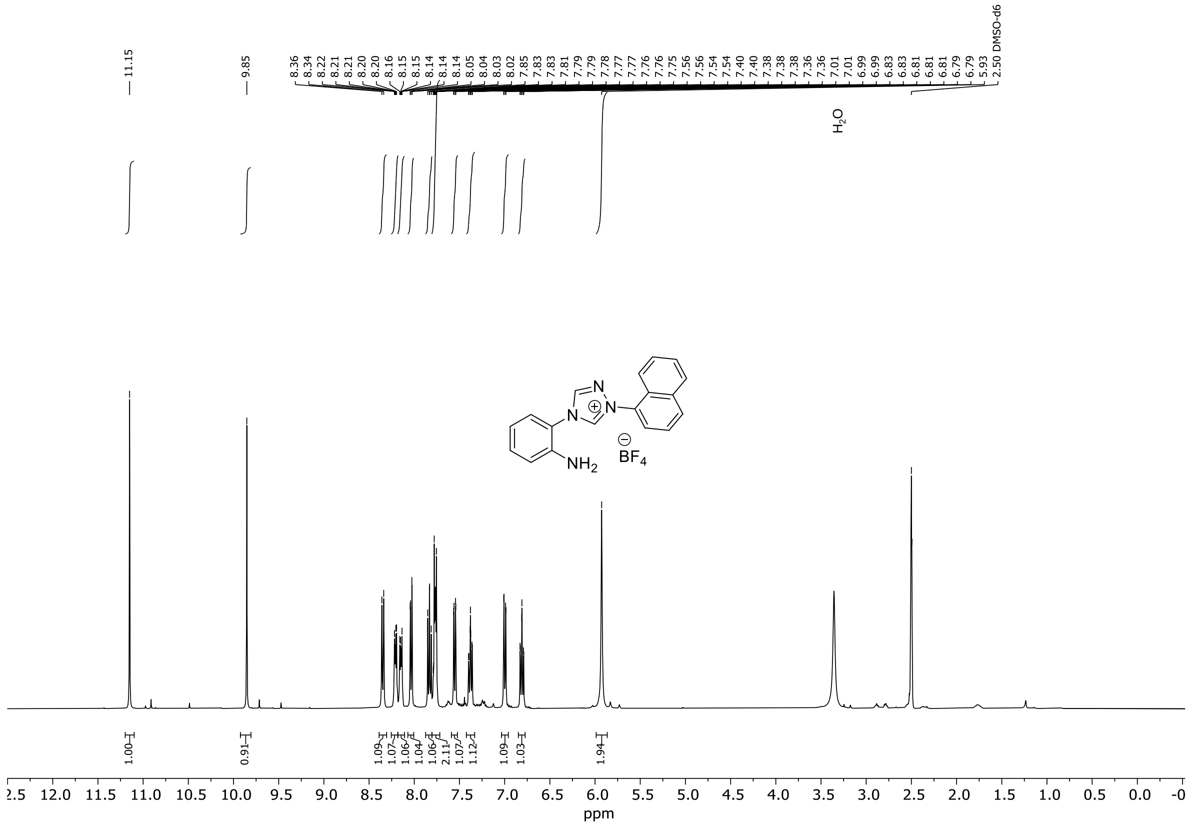

Figure S70: ^{1}H NMR spectrum of 4-(2-aminophenyl)-1-(naphthalen-1-yl)-4*H*-1,2,4-triazol-1-ium tetrafluoroborate (**5c**) (400 MHz, DMSO-d_6, 298 K).

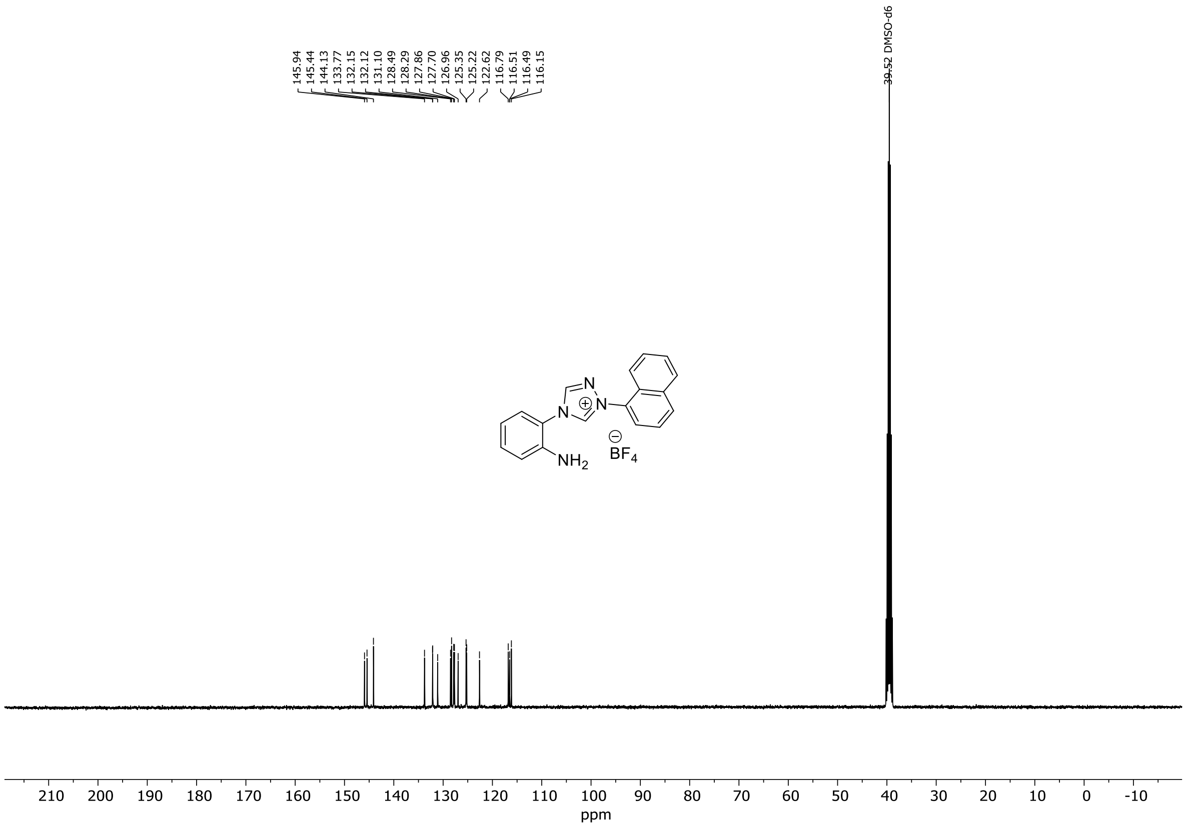

Figure S71: $^{13}C\{^{1}H\}$ NMR spectrum of 4-(2-aminophenyl)-1-(naphthalen-1-yl)-4*H*-1,2,4-triazol-1-ium tetrafluoroborate (**5c**) (100 MHz, DMSO-d_6, 298 K).

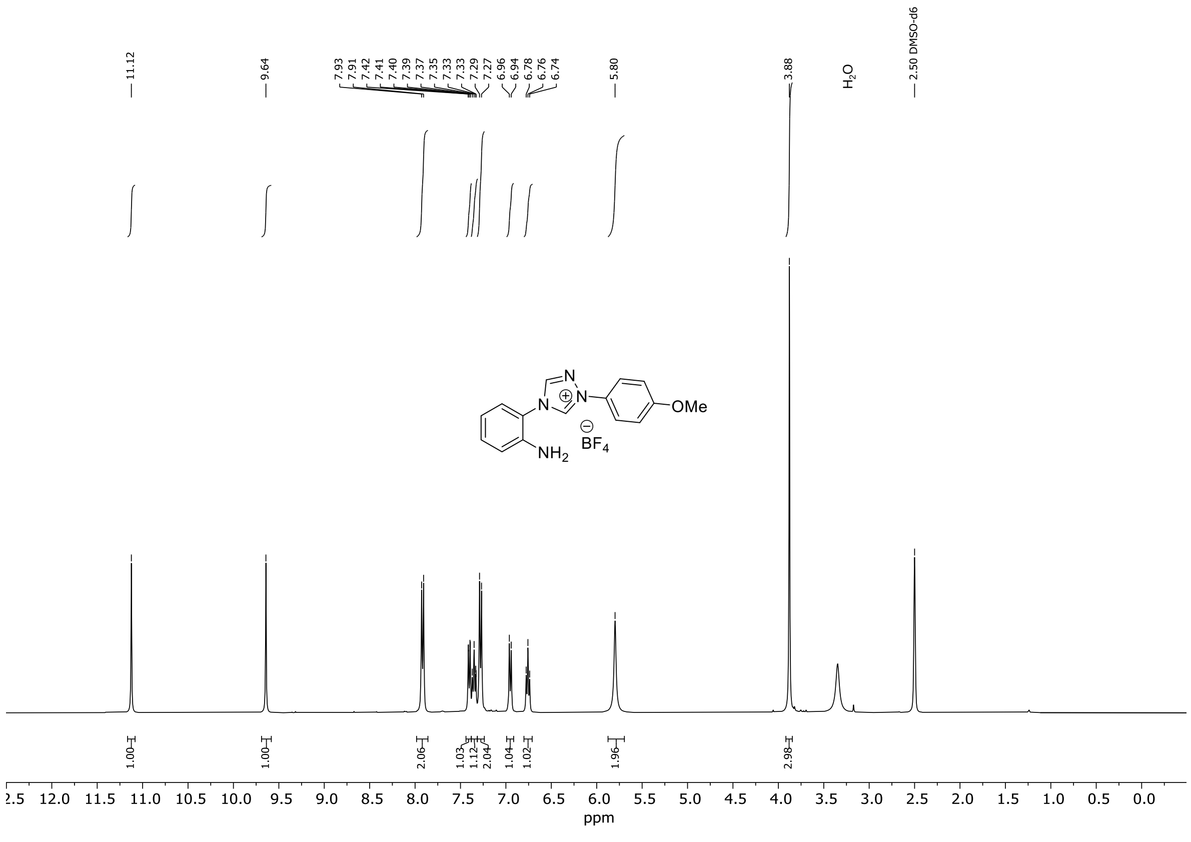

Figure S72: ^{1}H NMR spectrum of 4-(2-aminophenyl)-1-(4-methoxyphenyl)-4*H*-1,2,4-triazol-1-ium tetrafluoroborate (**5d**) (400 MHz, DMSO-d_6, 298 K).

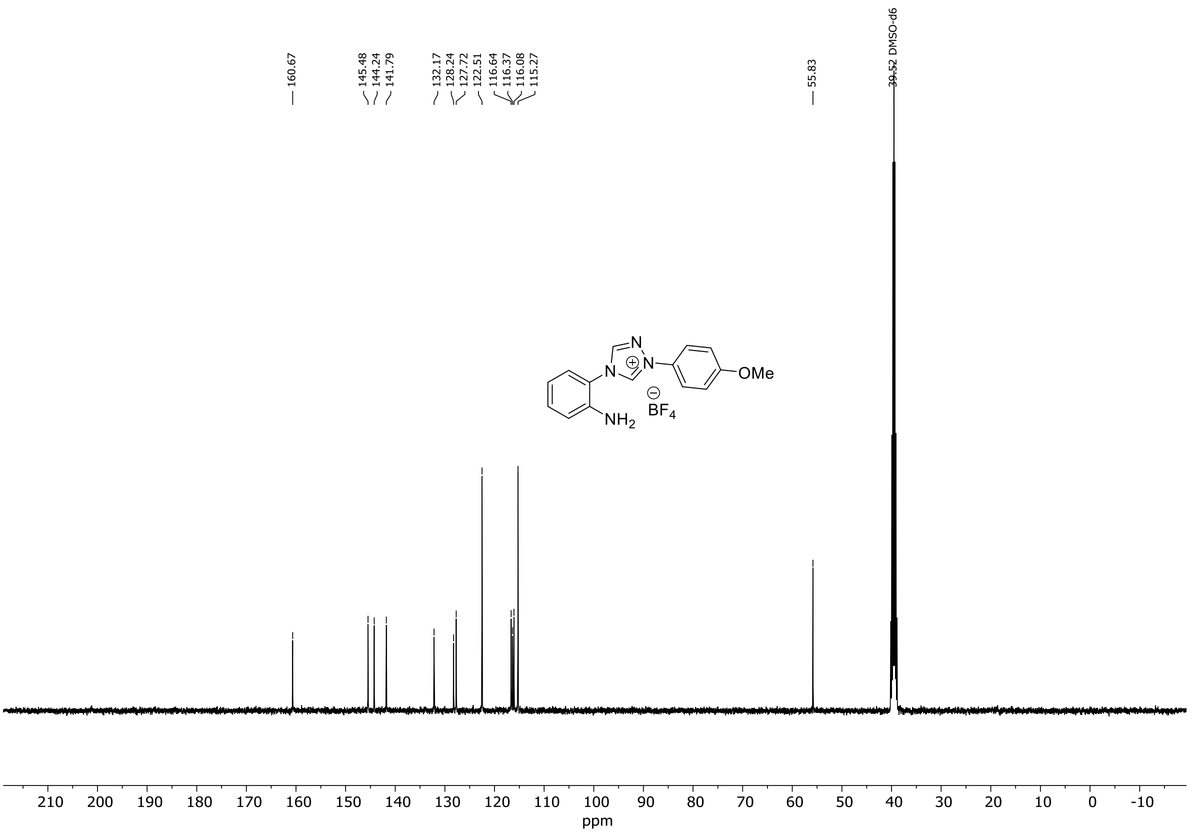

Figure S73: $^{13}C\{^1H\}$ NMR spectrum of 4-(2-aminophenyl)-1-(4-methoxyphenyl)-4*H*-1,2,4-triazol-1-ium tetrafluoroborate (**5d**) (100 MHz, DMSO-d_6, 298 K).

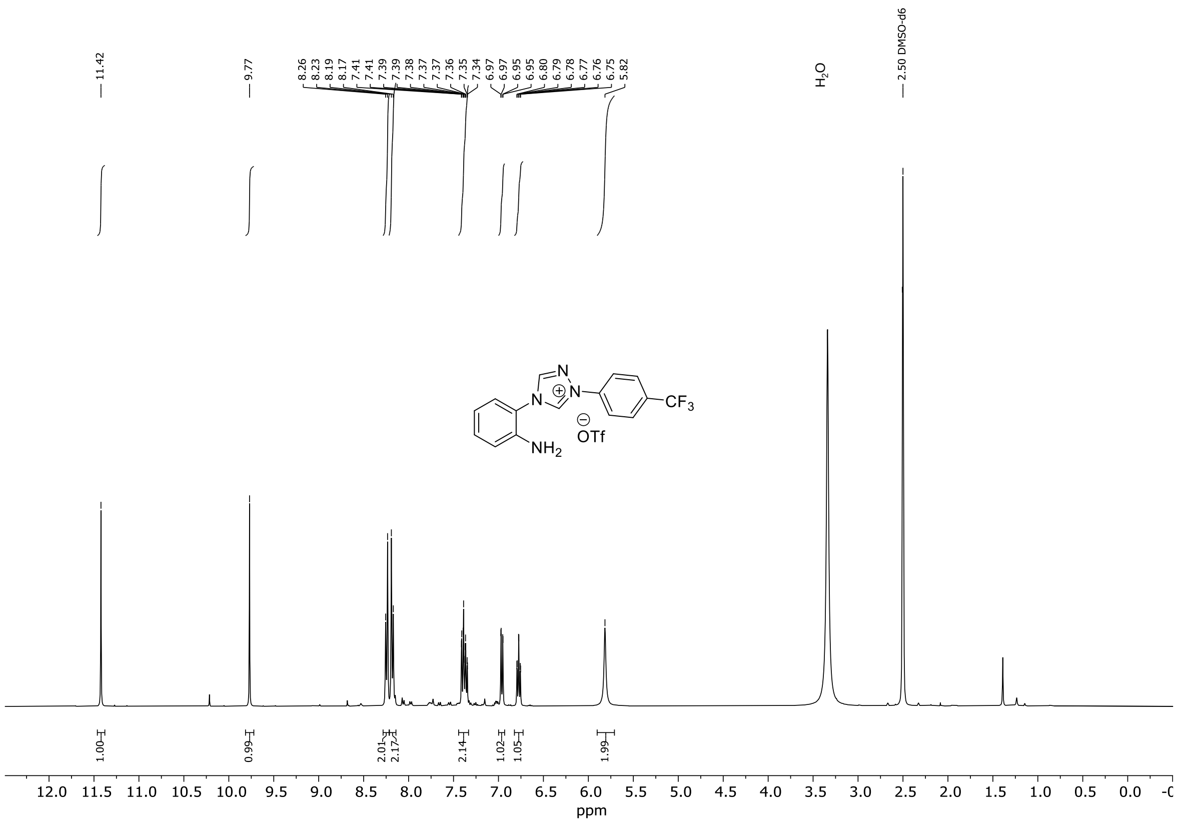

Figure S74: ^1H NMR spectrum of 4-(2-aminophenyl)-1-(4-(trifluoromethyl)phenyl)-4*H*-1,2,4-triazol-1-ium trifluoromethanesulfonate (**5e**) (400 MHz, DMSO-d_6, 298 K).

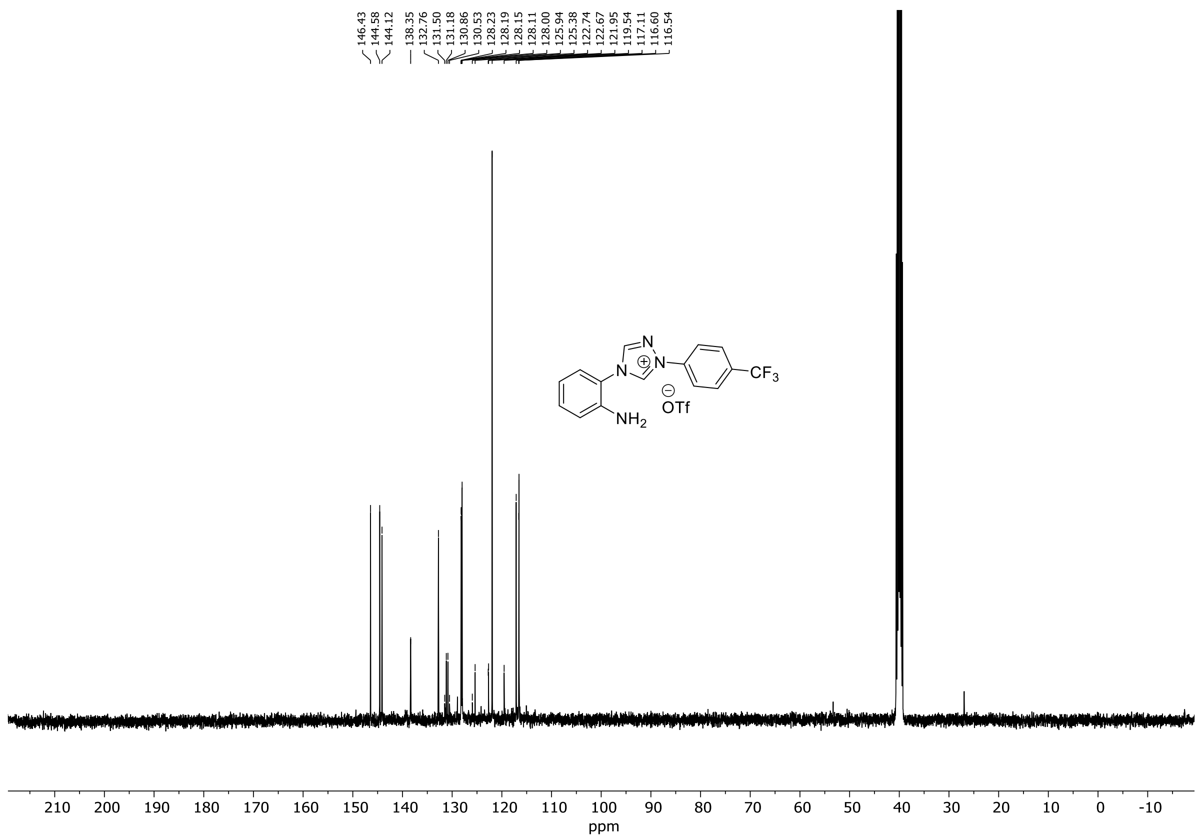

Figure S75: $^{13}C\{^1H\}$ NMR spectrum of 4-(2-aminophenyl)-1-(4-(trifluoromethyl)phenyl)-4*H*-1,2,4-triazol-1-ium trifluoromethanesulfonate (**5e**) (100 MHz, DMSO-d_6, 298 K).

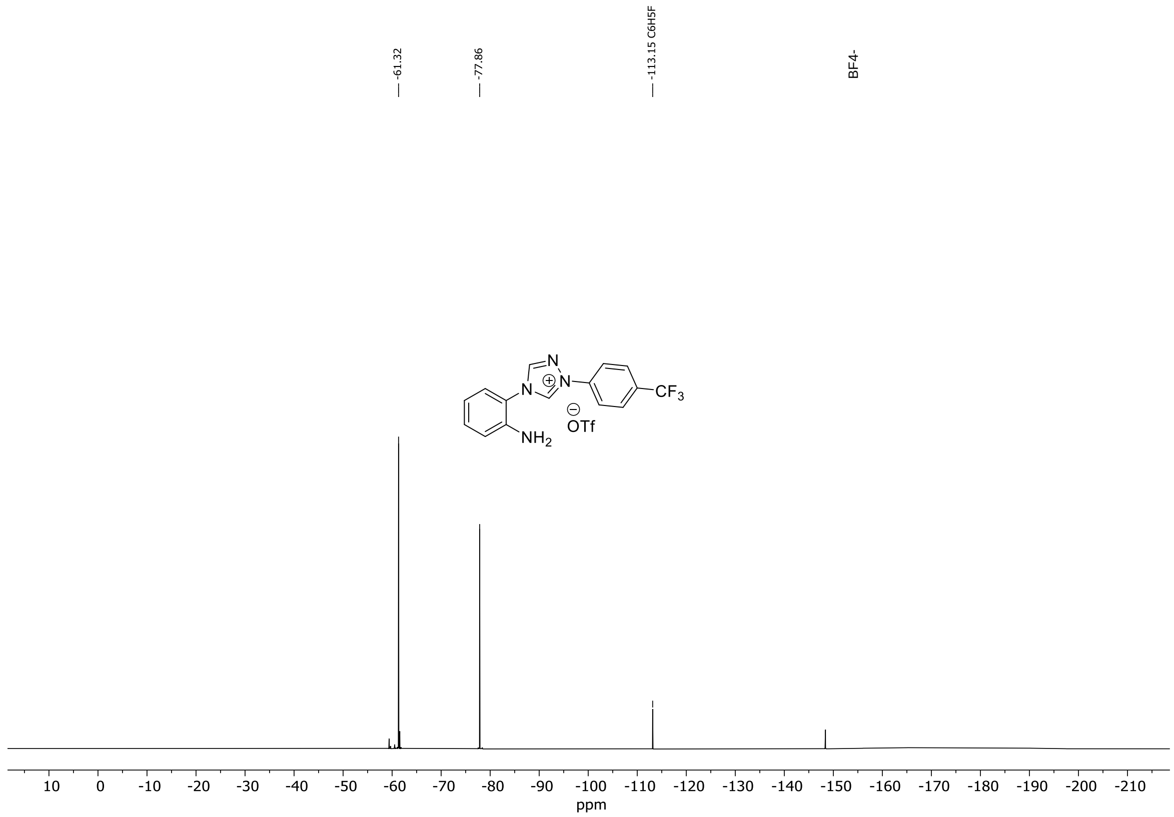

Figure S76: $^{19}F\{^1H\}$ NMR spectrum of 4-(2-aminophenyl)-1-(4-(trifluoromethyl)phenyl)-4*H*-1,2,4-triazol-1-ium trifluoromethanesulfonate (**5e**) (376 MHz, DMSO-d_6, 298 K, referenced to fluorobenzene).

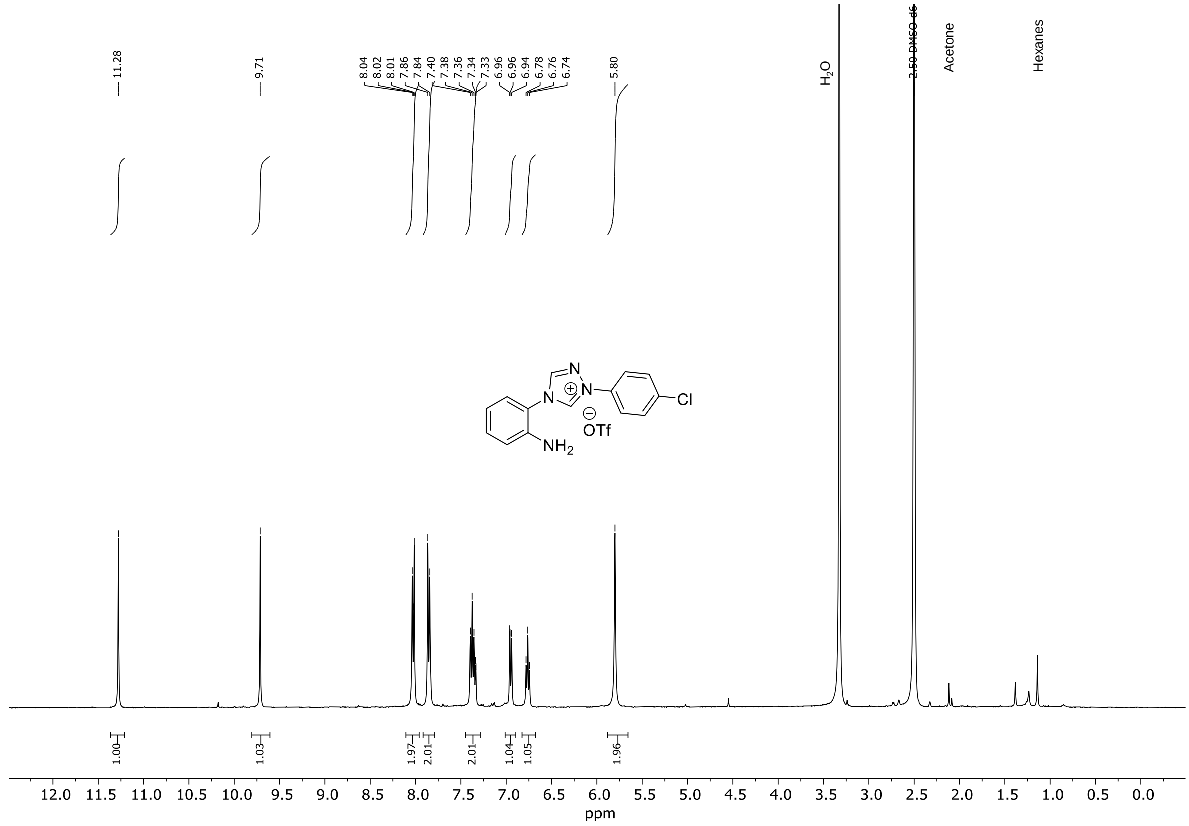

Figure S77: ^{1}H NMR spectrum of 4-(2-aminophenyl)-1-(4-chlorophenyl)-4*H*-1,2,4-triazol-1-ium trifluoromethanesulfonate (**5f**) (400 MHz, DMSO-d_6, 298 K).

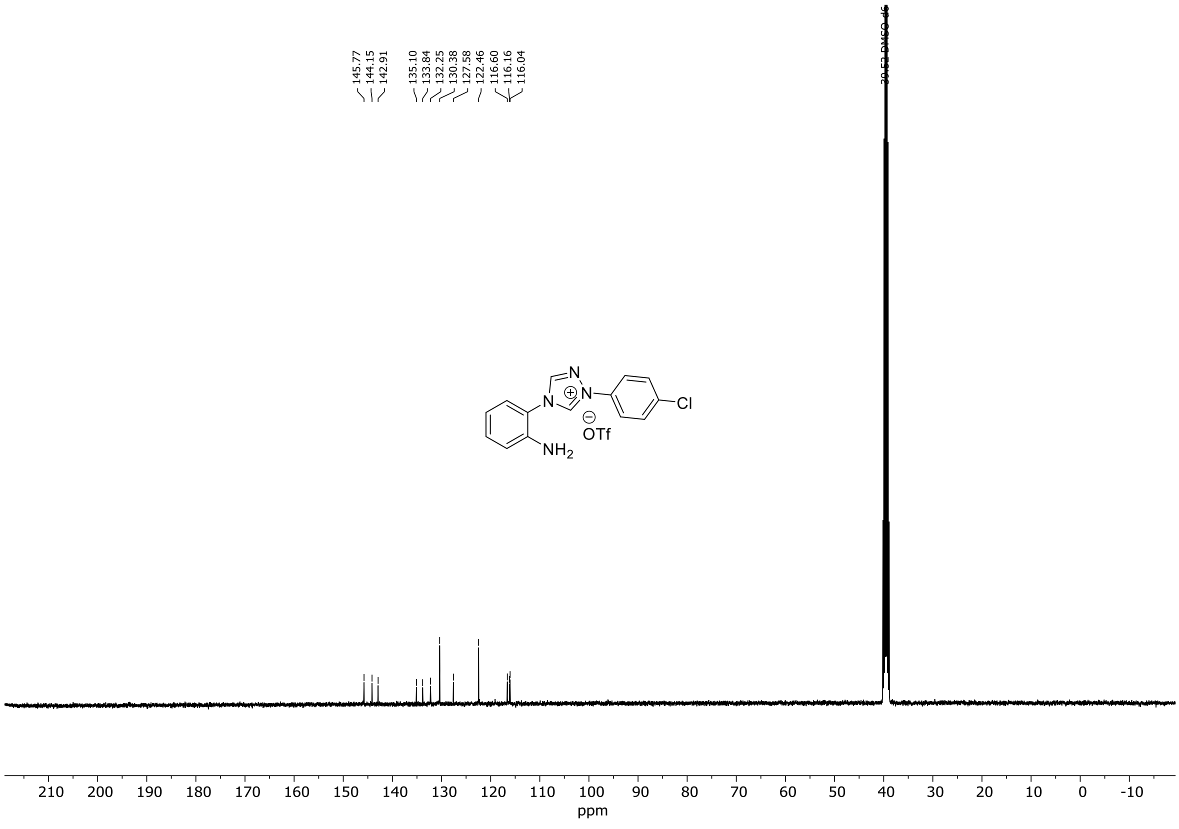

Figure S78: $^{13}C\{^{1}H\}$ NMR spectrum of 4-(2-aminophenyl)-1-(4-chlorophenyl)-4*H*-1,2,4-triazol-1-ium trifluoromethanesulfonate (**5f**) (100 MHz, DMSO-d_6, 298 K).

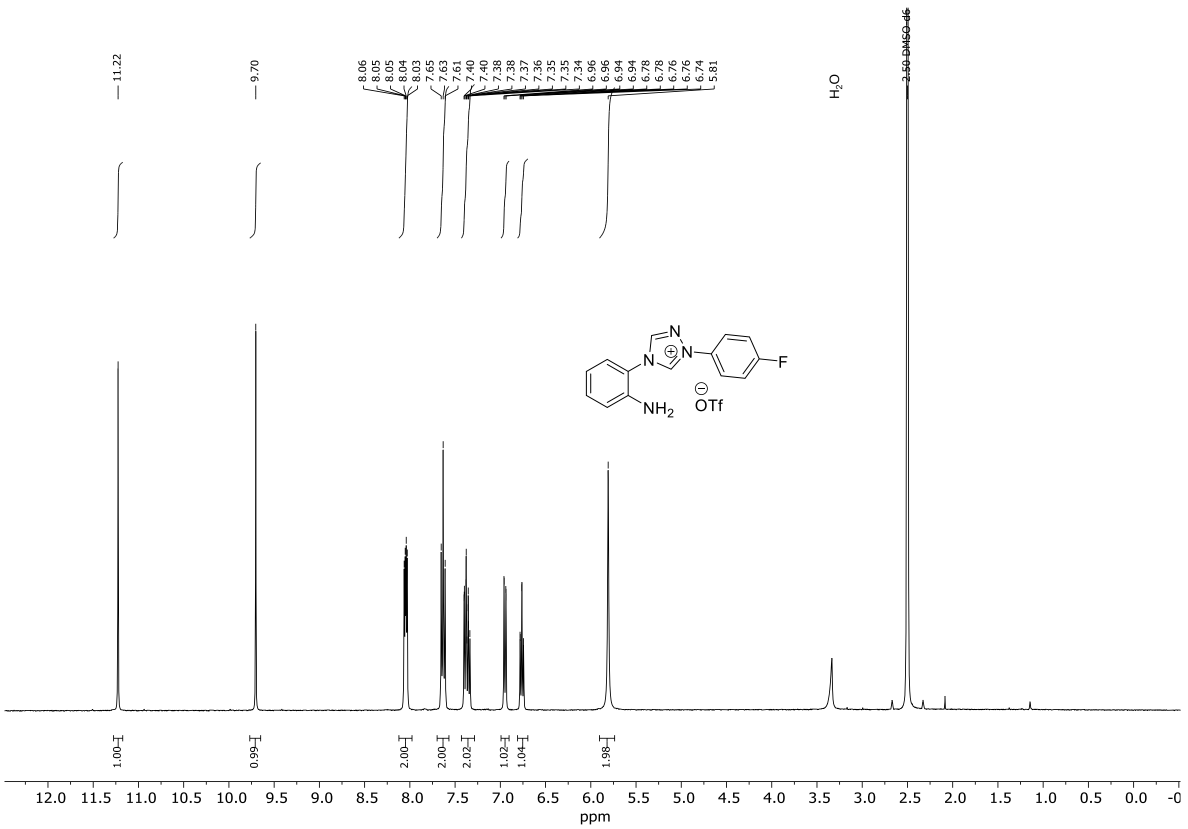

Figure S79: ^{1}H NMR spectrum of 4-(2-aminophenyl)-1-(4-fluorophenyl)-4*H*-1,2,4-triazol-1-ium trifluoromethanesulfonate (**5g**) (400 MHz, DMSO-d_6, 298 K).

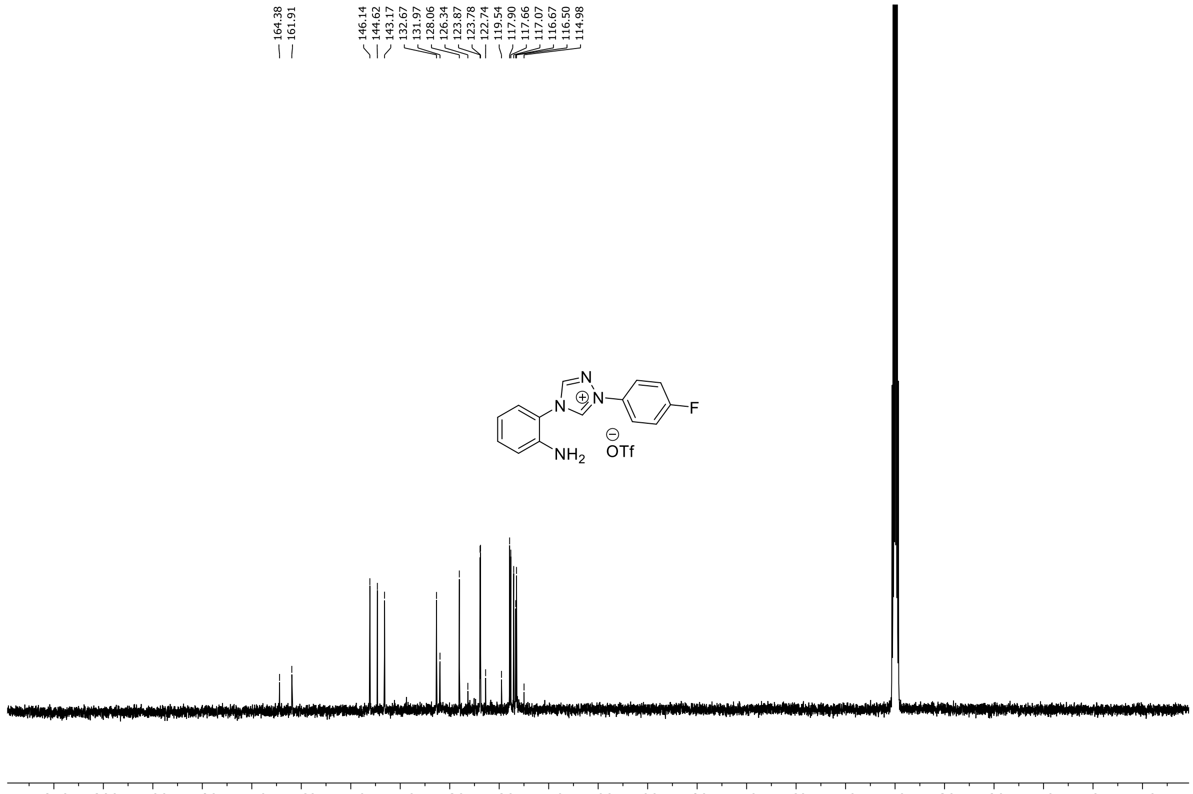

Figure S80: $^{13}C\{^1H\}$ NMR spectrum of 4-(2-aminophenyl)-1-(4-fluorophenyl)-4*H*-1,2,4-triazol-1-ium trifluoromethanesulfonate (**5g**) (100 MHz, DMSO-d_6, 298 K).

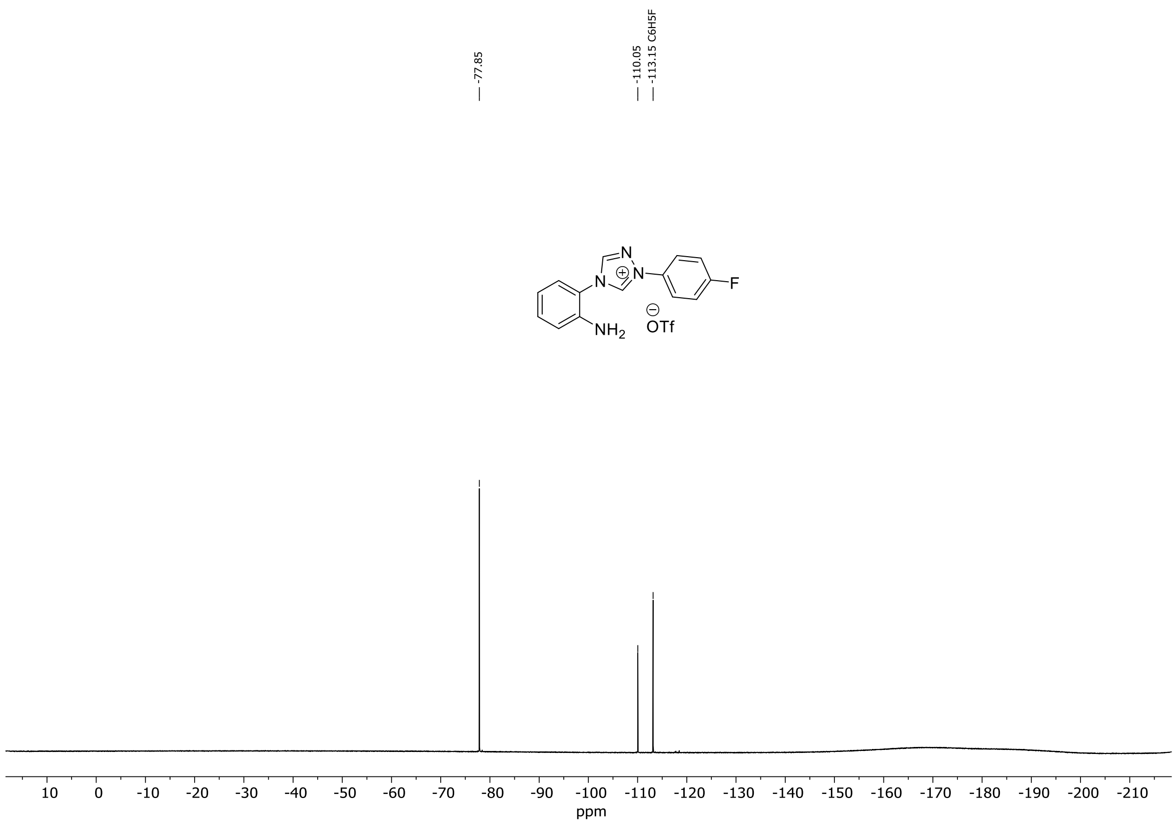

Figure S81: $^{19}F\{^1H\}$ NMR spectrum of 4-(2-aminophenyl)-1-(4-fluorophenyl)-4*H*-1,2,4-triazol-1-ium trifluoromethanesulfonate (**5g**) (376 MHz, DMSO-d_6, 298 K, referenced to fluorobenzene).

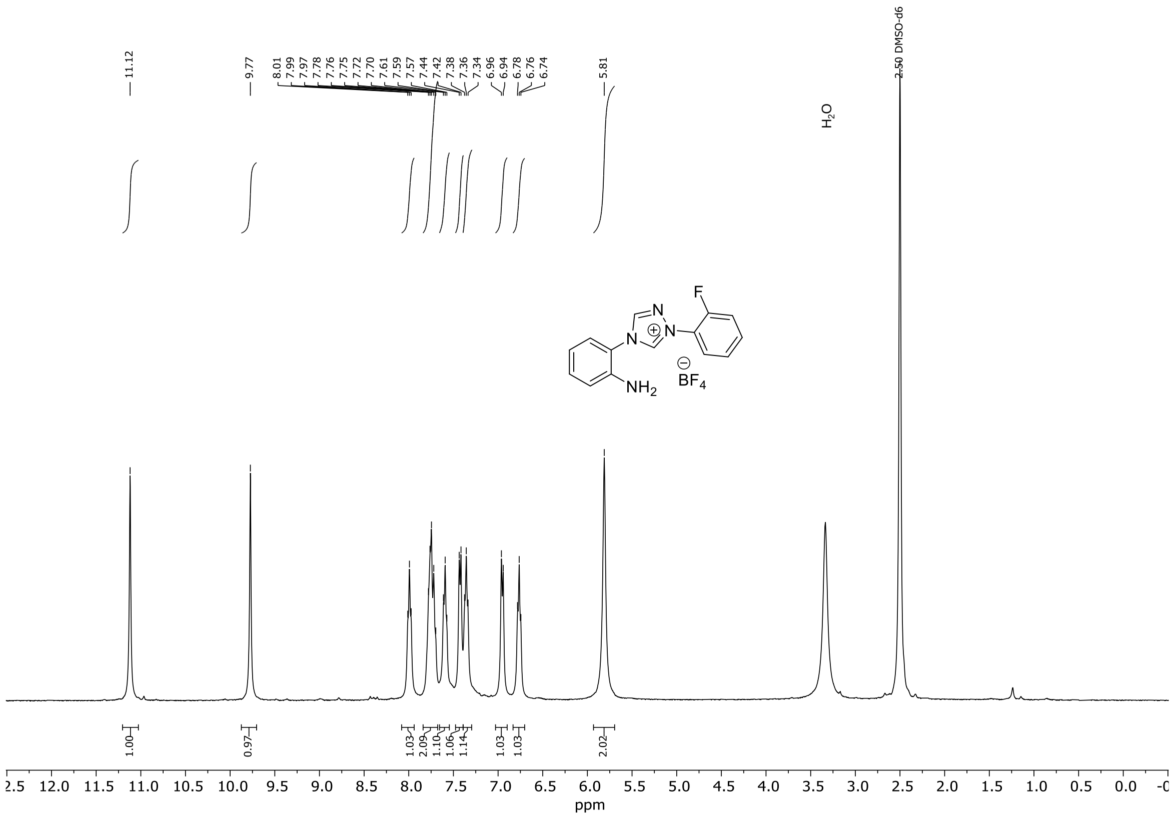

Figure S82: ^1H NMR spectrum of 4-(2-aminophenyl)-1-(2-fluorophenyl)-4*H*-1,2,4-triazol-1-ium tetrafluoroborate (**5h**) (400 MHz, DMSO-d_6, 298 K).

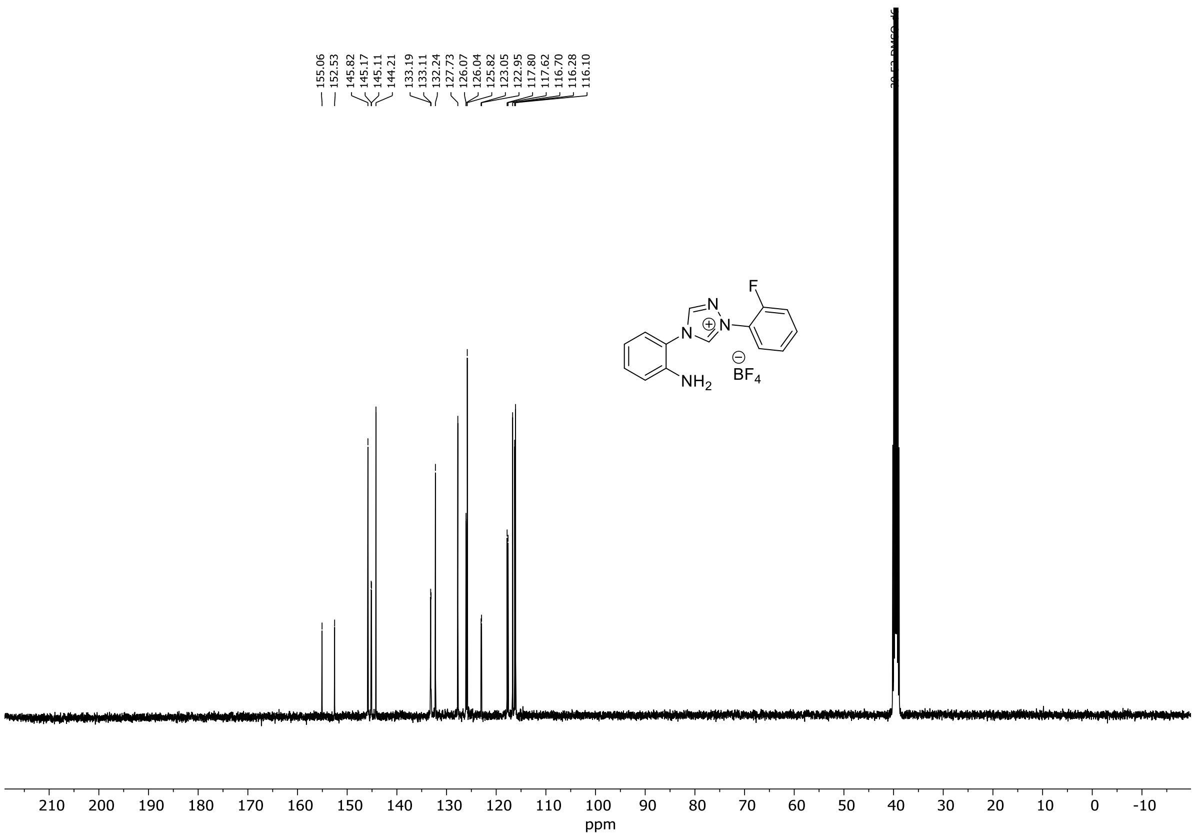

Figure S83: $^{13}C\{^{1}H\}$ NMR spectrum of 4-(2-aminophenyl)-1-(2-fluorophenyl)-4*H*-1,2,4-triazol-1-ium tetrafluoroborate (**5h**) (100 MHz, DMSO-d_6, 298 K).

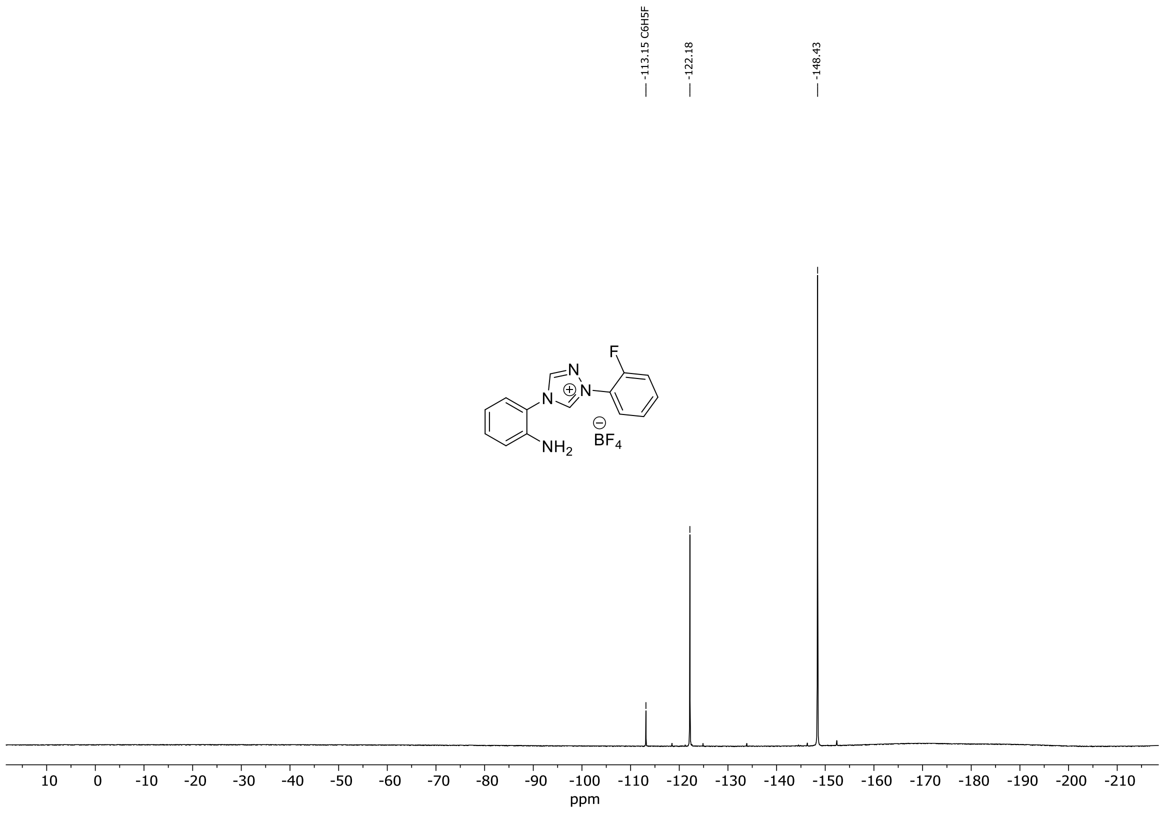

Figure S84: $^{19}F\{^{1}H\}$ NMR spectrum of 4-(2-aminophenyl)-1-(2-fluorophenyl)-4*H*-1,2,4-triazol-1-ium tetrafluoroborate (**5h**) (376 MHz, DMSO-d_6, 298 K, referenced to fluorobenzene).

CYCLIZATION PRODUCTS

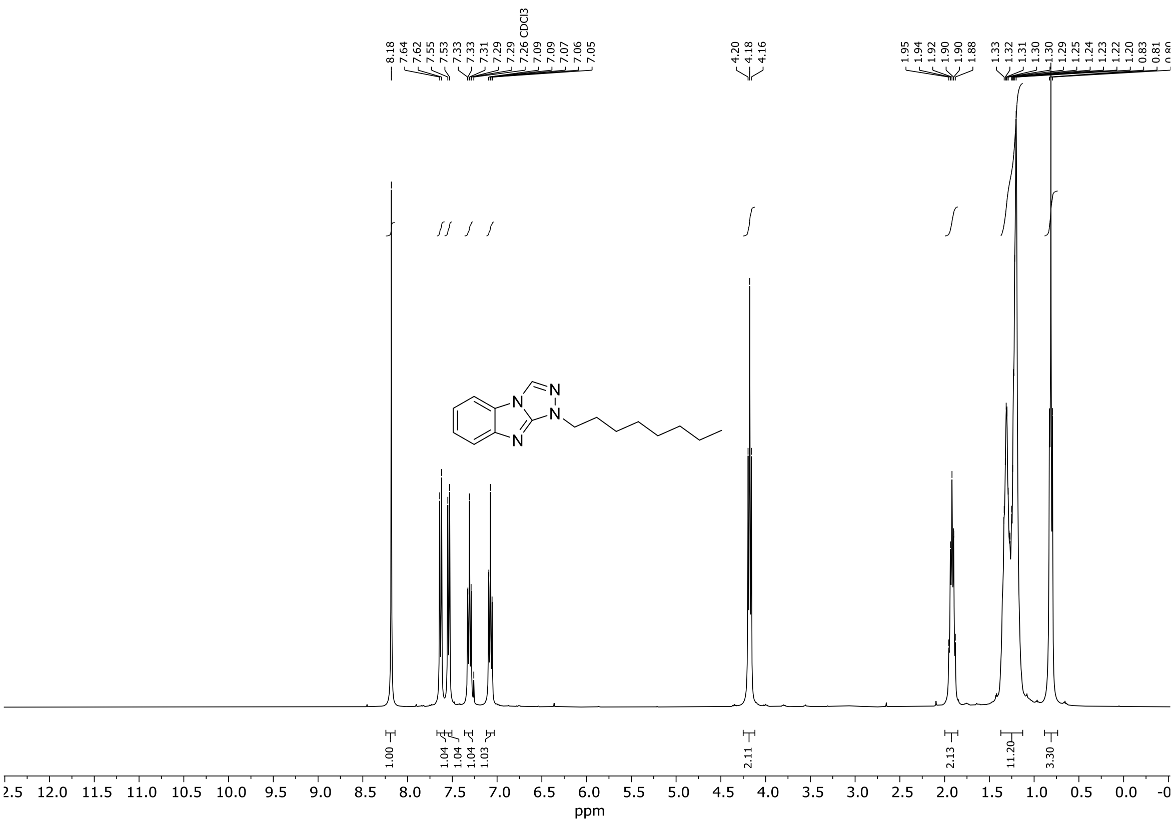

Figure S85: ^{1}H NMR spectrum of 1-octyl-1*H*-benzo[4,5]imidazo[2,1-*c*][1,2,4]triazole (**6a**) (400 MHz, $CDCl_3$, 298 K).

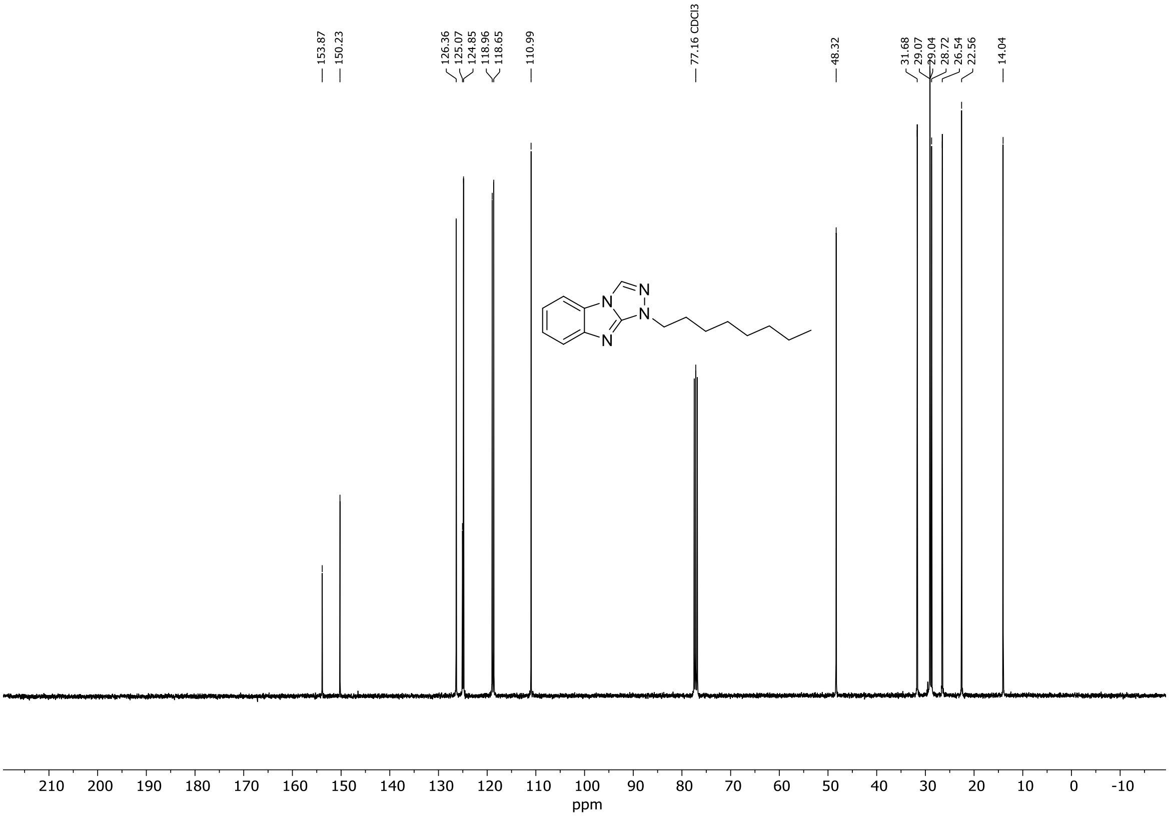

Figure S86: $^{13}C\{^{1}H\}$ NMR spectrum of 1-octyl-1*H*-benzo[4,5]imidazo[2,1-*c*][1,2,4]triazole (**6a**) (100 MHz, $CDCl_3$, 298 K).

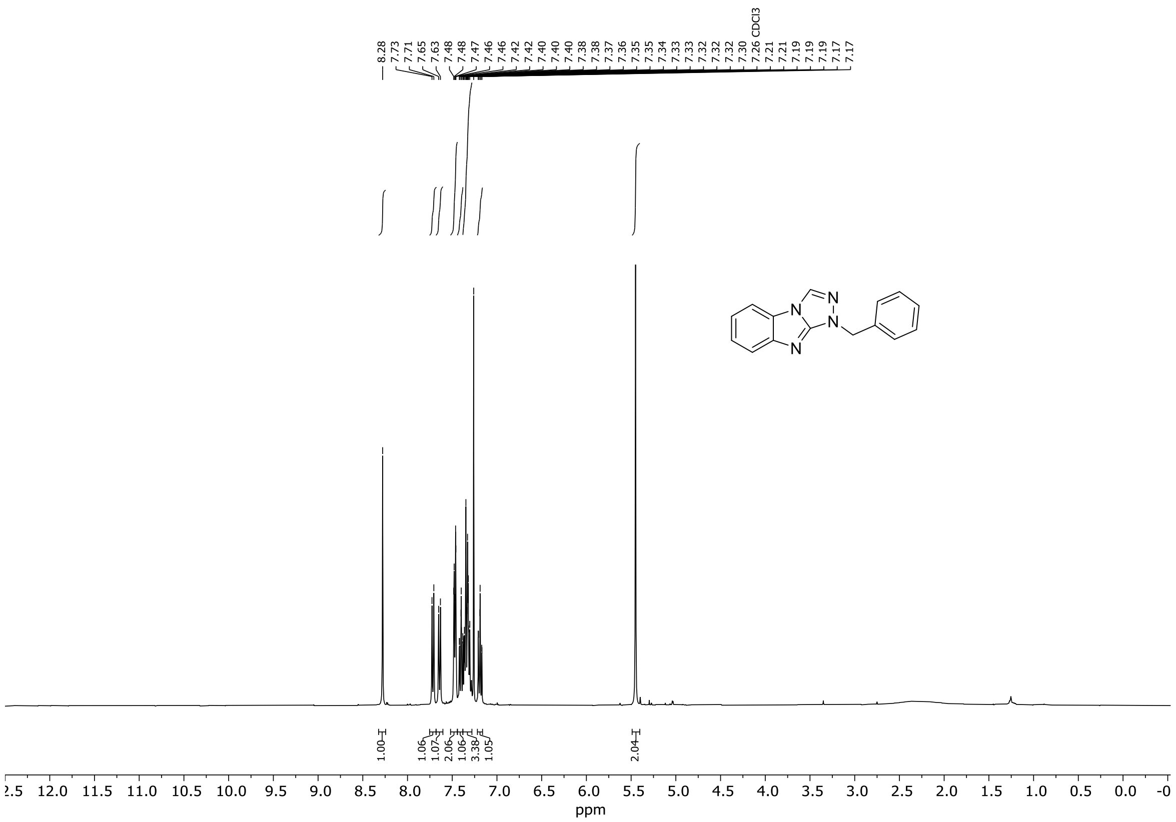

Figure S87: ^{1}H NMR spectrum of 1-benzyl-1*H*-benzo[4,5]imidazo[2,1-*c*][1,2,4]triazole (**6b**) (400 MHz, $CDCl_3$, 298 K).

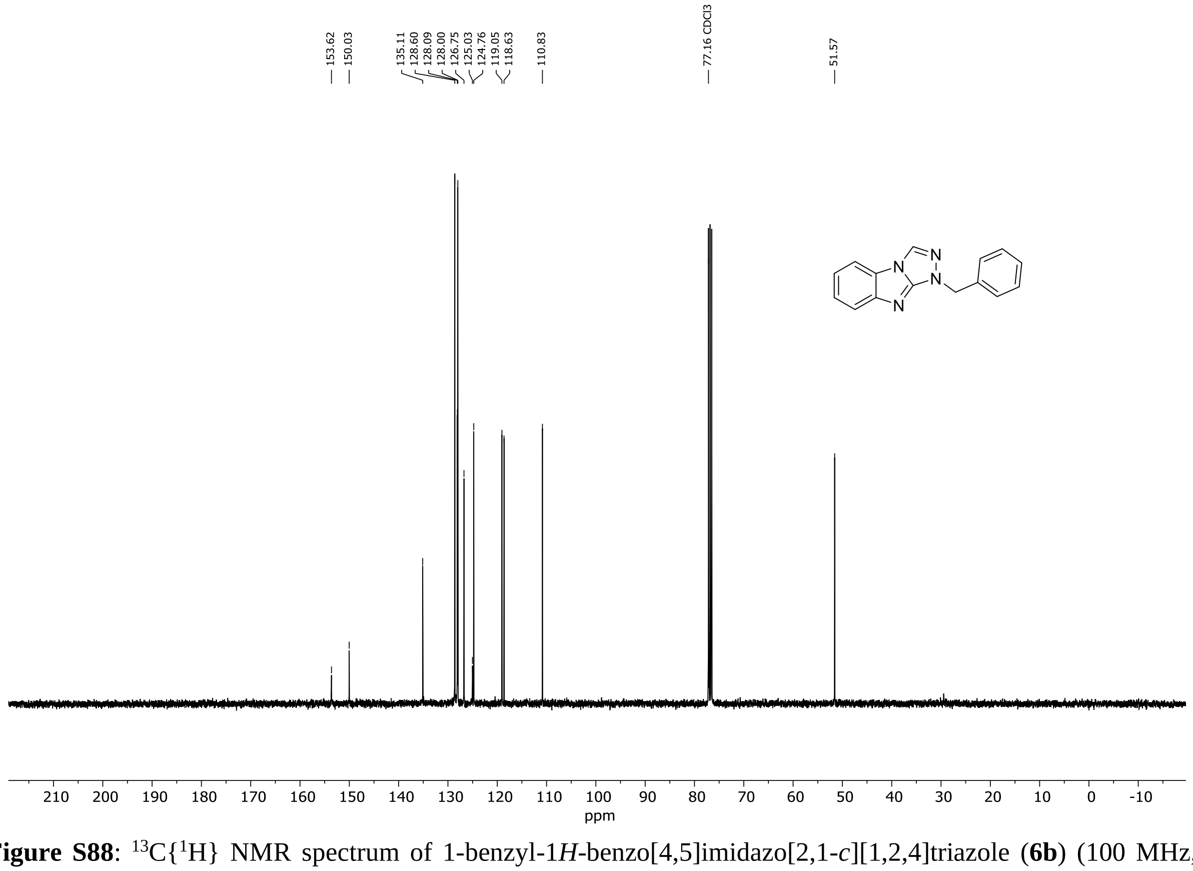

Figure S88: $^{13}C\{^{1}H\}$ NMR spectrum of 1-benzyl-1*H*-benzo[4,5]imidazo[2,1-*c*][1,2,4]triazole (**6b**) (100 MHz, $CDCl_3$, 298 K).

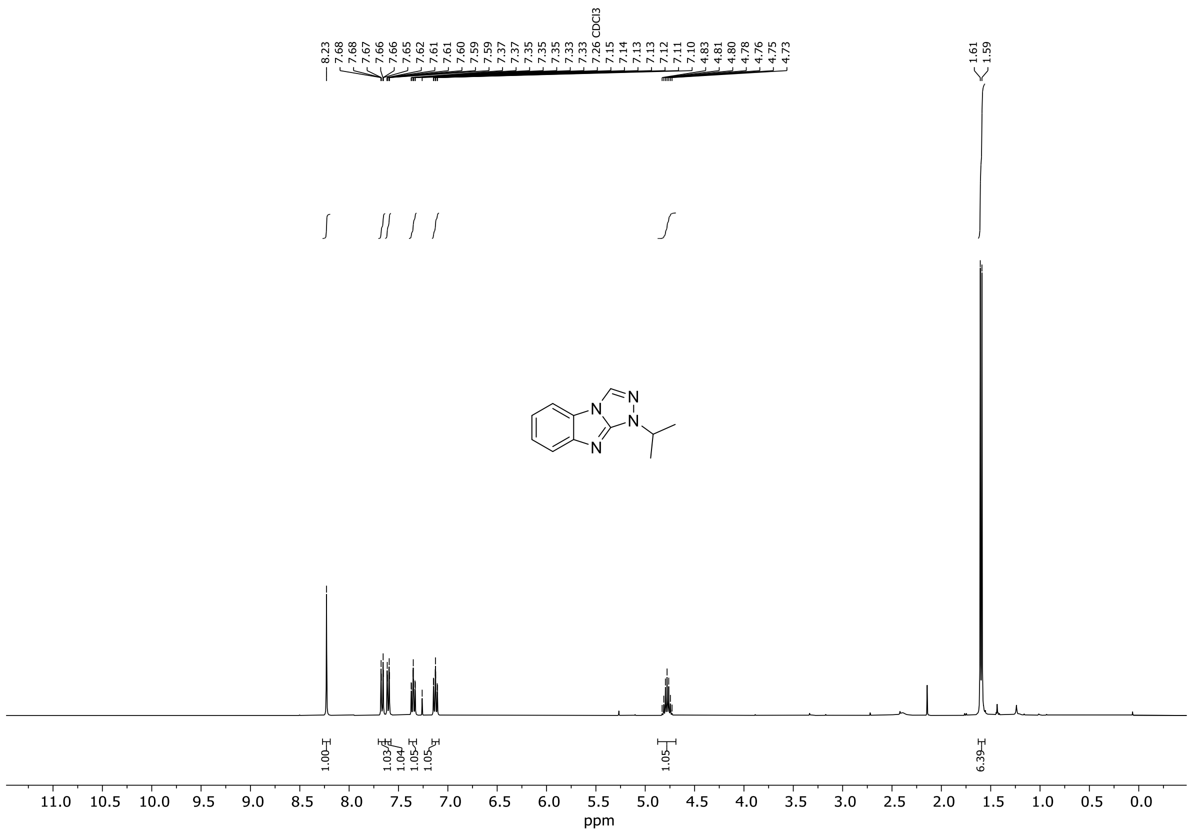

Figure S89: ^{1}H NMR spectrum of 1-isopropyl-1*H*-benzo[4,5]imidazo[2,1-*c*][1,2,4]triazole (**6c**) (400 MHz, $CDCl_3$, 298 K).

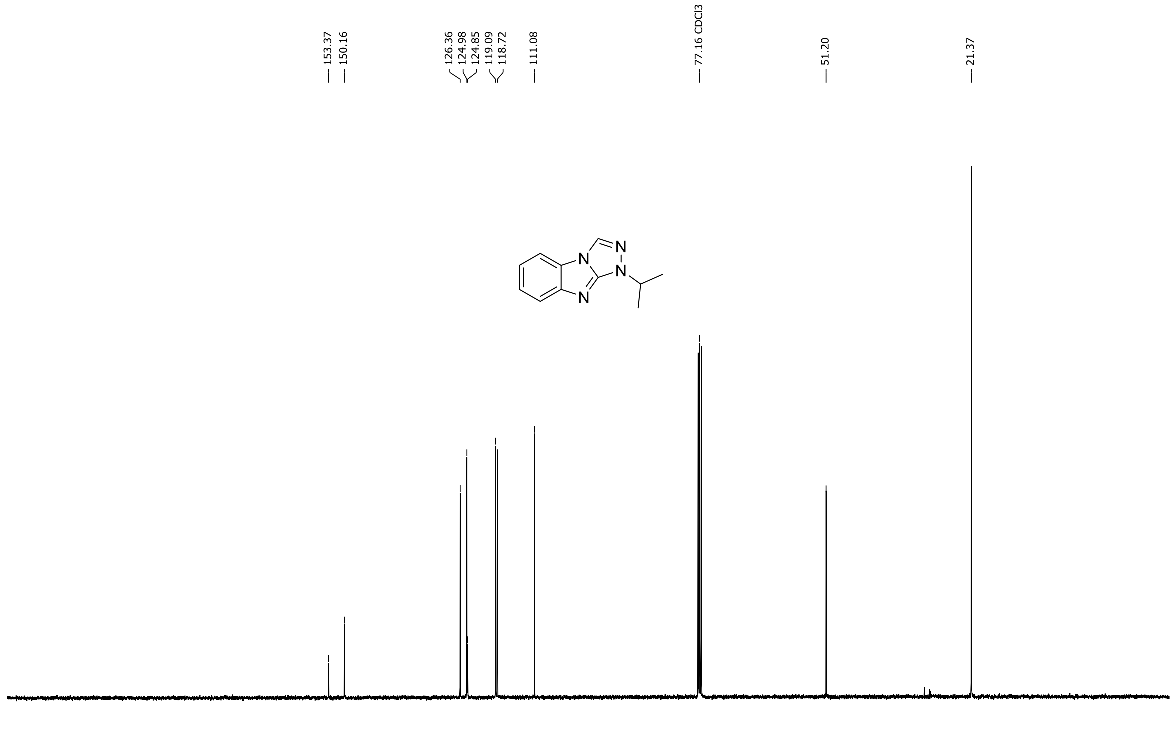

Figure S90: ^{13}C{^{1}H} NMR spectrum of 1-isopropyl-1*H*-benzo[4,5]imidazo[2,1-*c*][1,2,4]triazole (**6c**) (100 MHz, $CDCl_3$, 298 K).

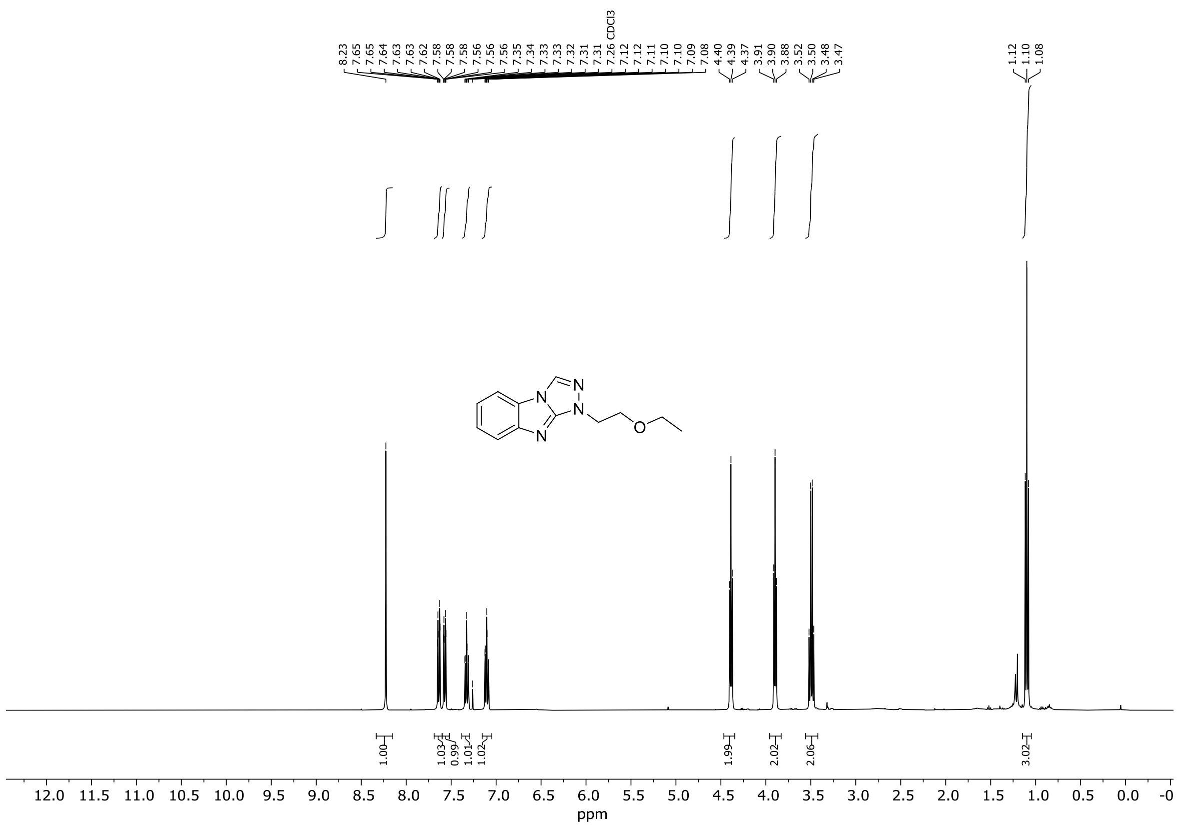

Figure S91: ^{1}H NMR spectrum of 1-(2-ethoxyethyl)-1*H*-benzo[4,5]imidazo[2,1-*c*][1,2,4]triazole (**6d**) (400 MHz, $CDCl_3$, 298 K).

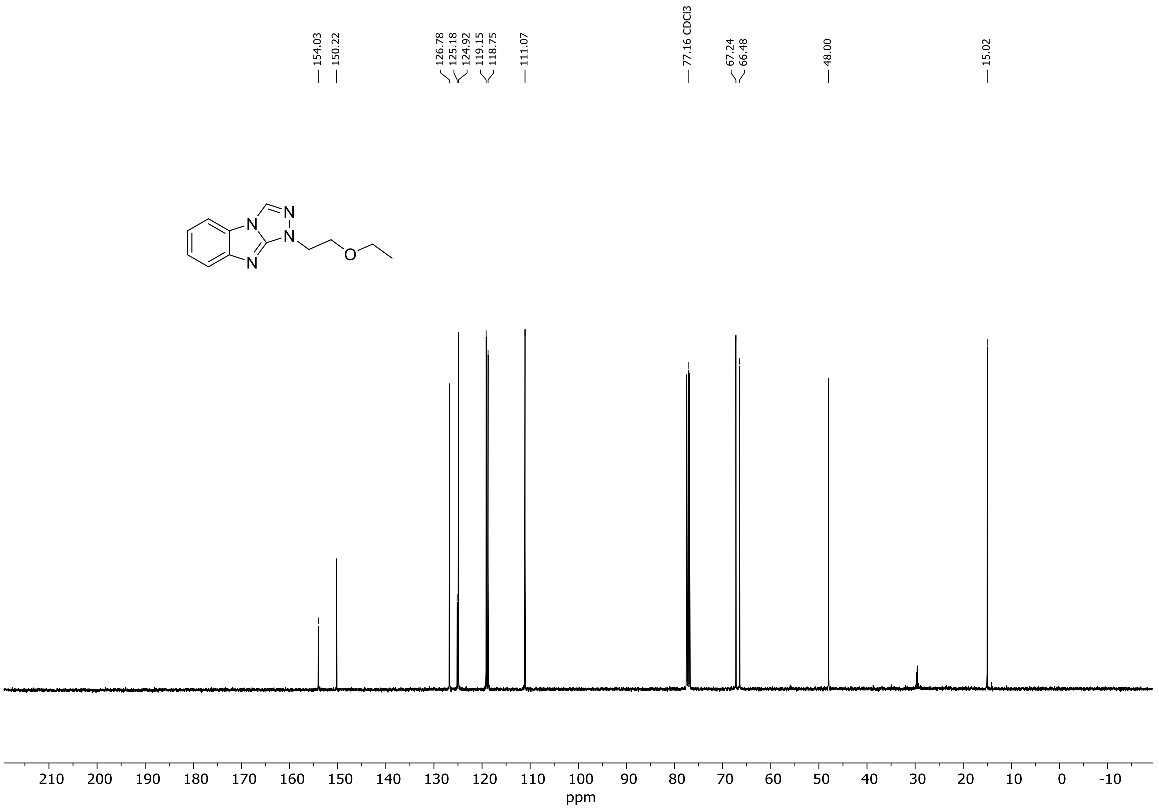

Figure S92: ^{13}C{^{1}H} NMR spectrum of 1-(2-ethoxyethyl)-1*H*-benzo[4,5]imidazo[2,1-*c*][1,2,4]triazole (**6d**) (100 MHz, $CDCl_3$, 298 K).

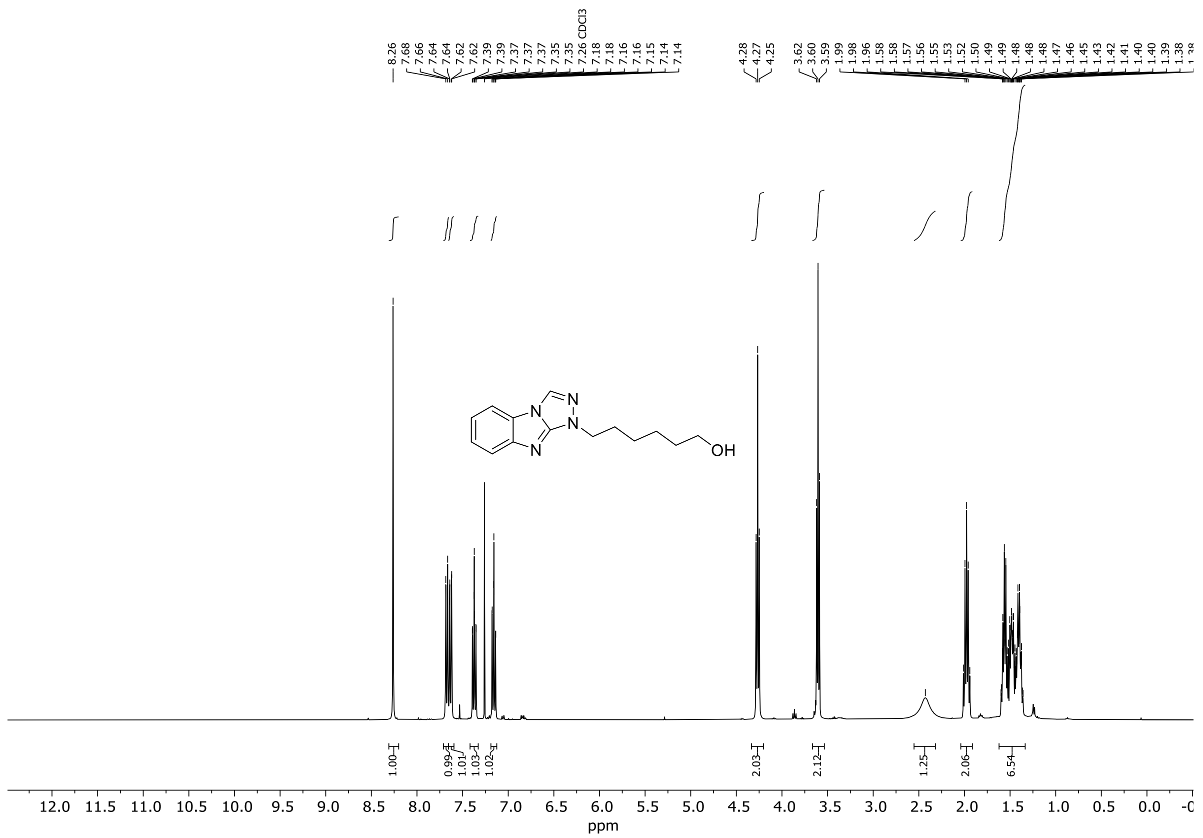

Figure S93: ^{1}H NMR spectrum of 6-(1*H*-benzo[4,5]imidazo[2,1-*c*][1,2,4]triazol-1-yl)hexan-1-ol (**6e**) (400 MHz, $CDCl_3$, 298 K).

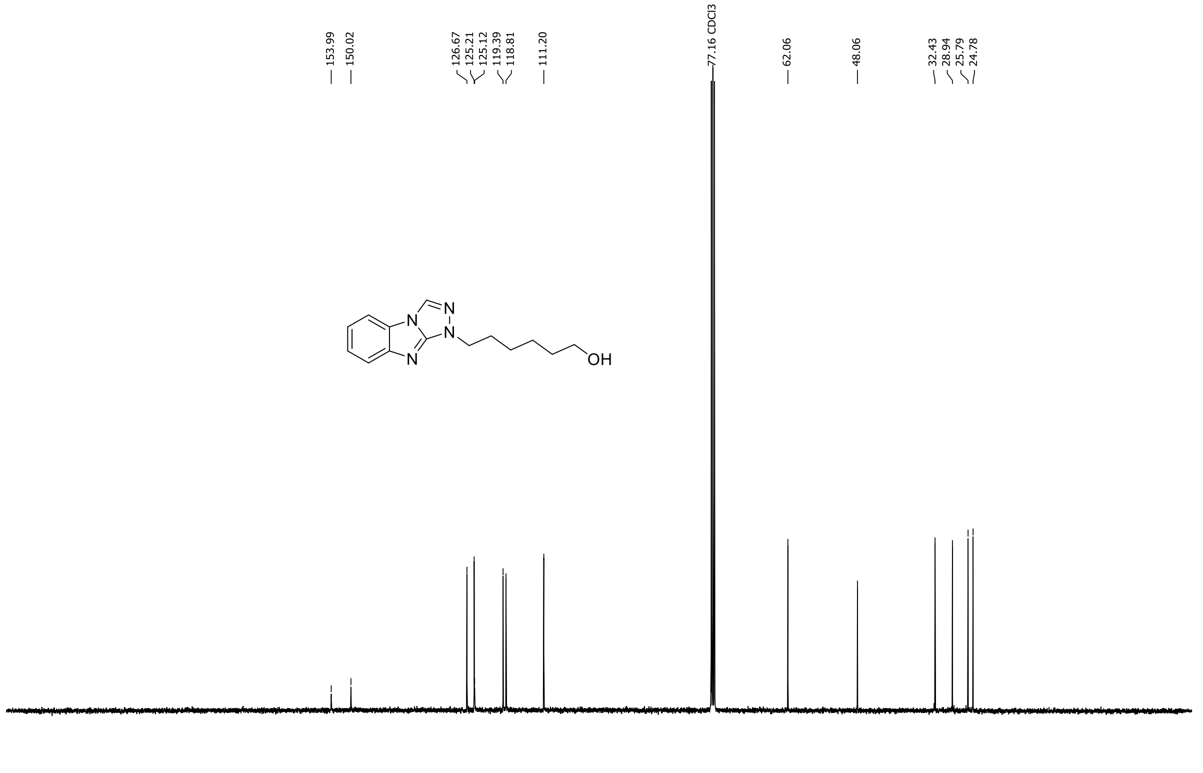

Figure S94: ^{13}C{^{1}H} NMR spectrum of 6-(1*H*-benzo[4,5]imidazo[2,1-*c*][1,2,4]triazol-1-yl)hexan-1-ol (**6e**) (100 MHz, $CDCl_3$, 298 K).

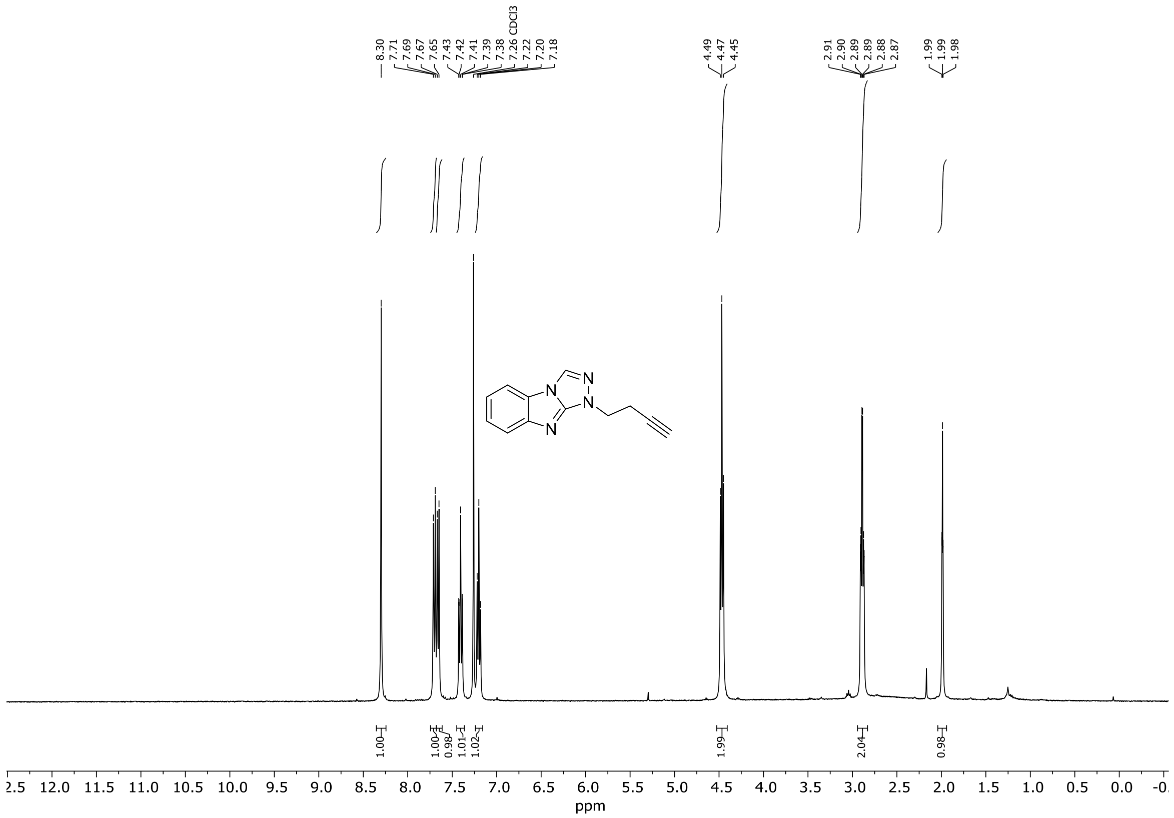

Figure S95: ^{1}H NMR spectrum of 1-(but-3-yn-1-yl)-1*H*-benzo[4,5]imidazo[2,1-*c*][1,2,4]triazole (**6f**) (400 MHz, $CDCl_3$, 298 K).

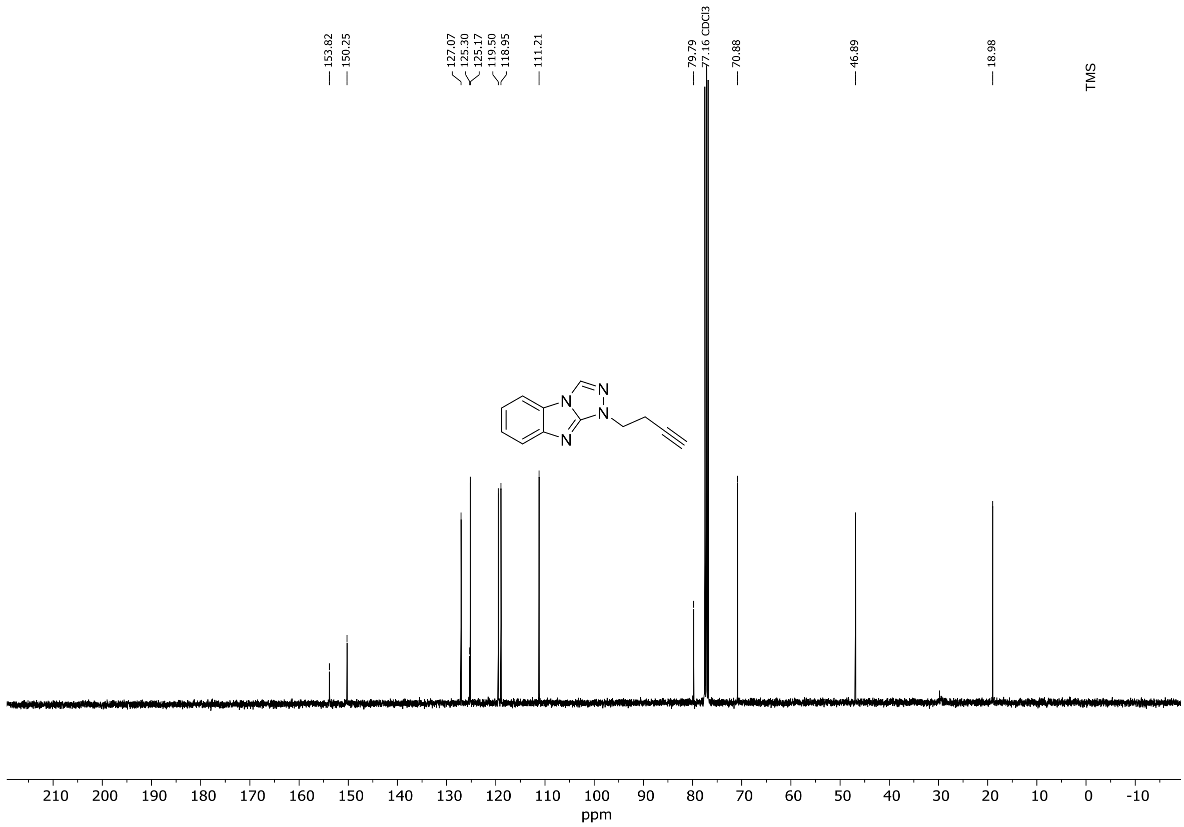

Figure S96: $^{13}C\{^{1}H\}$ NMR spectrum of 1-(but-3-yn-1-yl)-1*H*-benzo[4,5]imidazo[2,1-*c*][1,2,4]triazole (**6f**) (100 MHz, $CDCl_3$, 298 K).

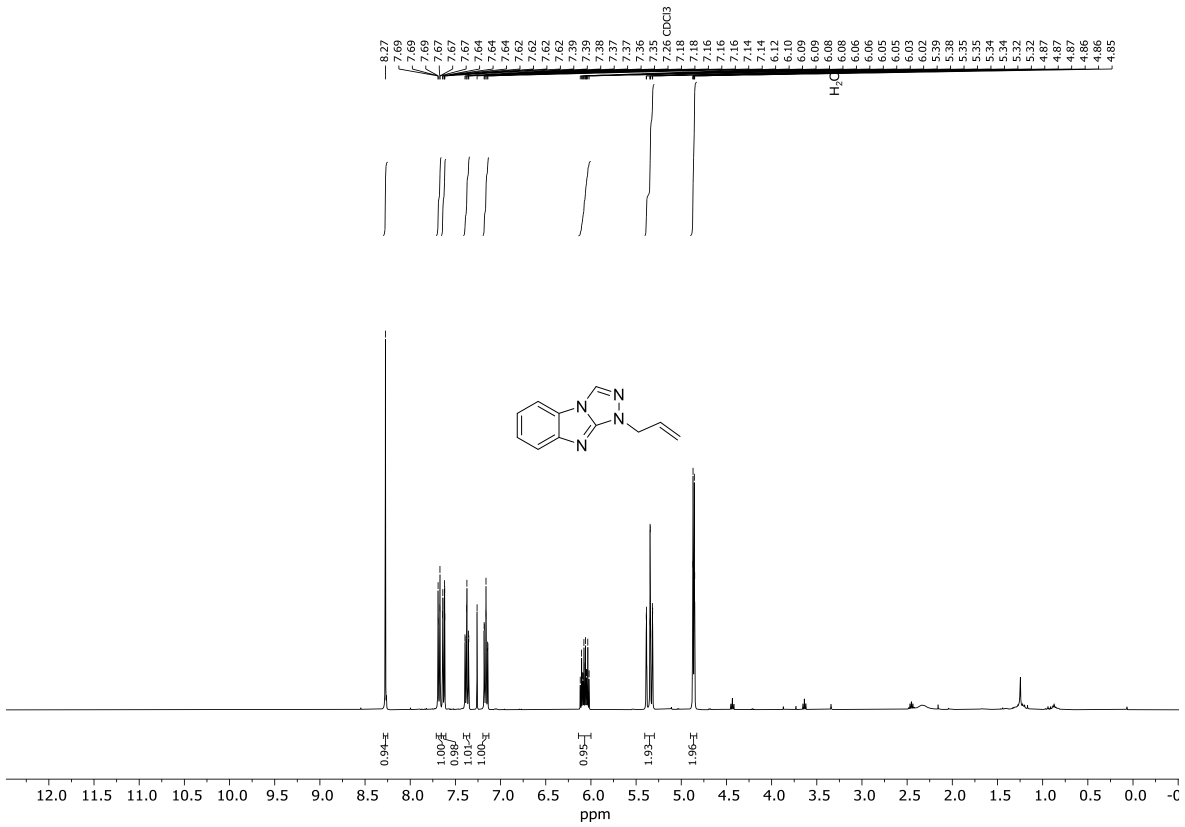

Figure S97: ^{1}H NMR spectrum of 1-allyl-1*H*-benzo[4,5]imidazo[2,1-*c*][1,2,4]triazole (**6g**) (400 MHz, $CDCl_3$, 298 K).

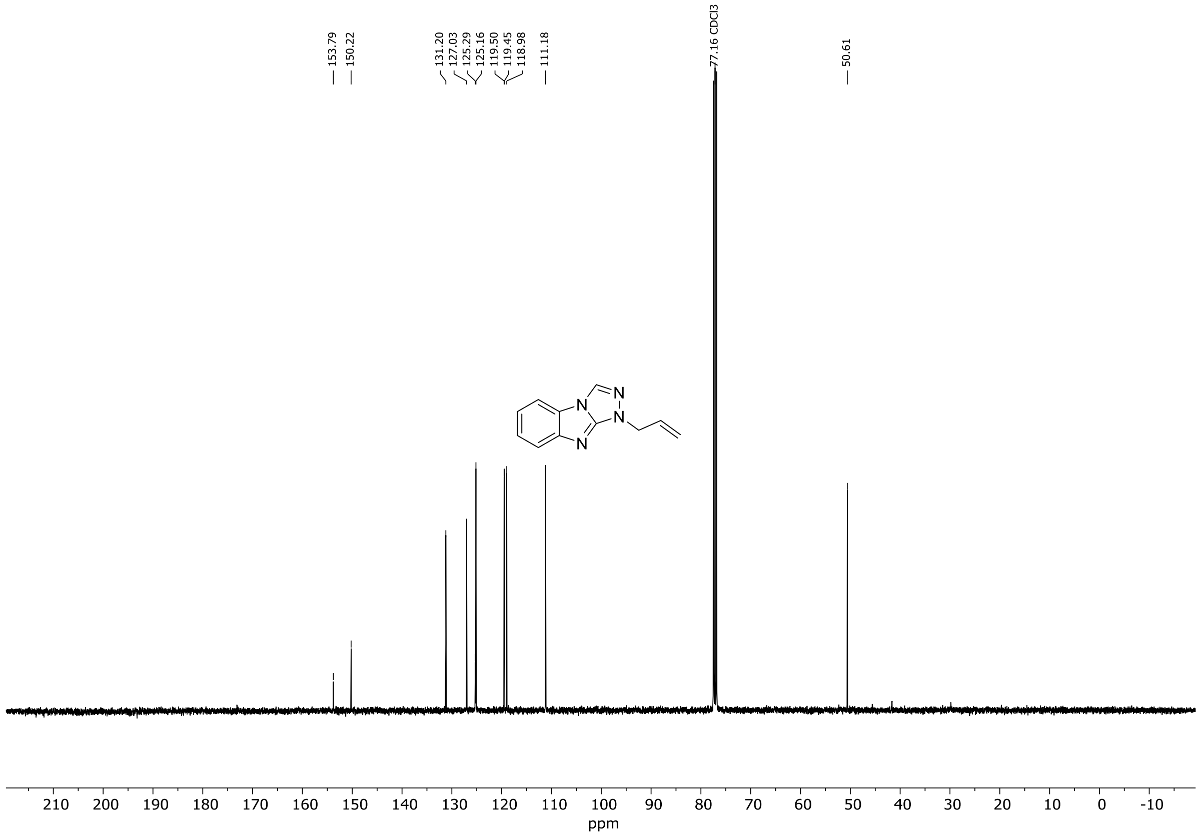

Figure S98: ^{13}C{^{1}H} NMR spectrum of 1-allyl-1*H*-benzo[4,5]imidazo[2,1-*c*][1,2,4]triazole (**6g**) (100 MHz, $CDCl_3$, 298 K).

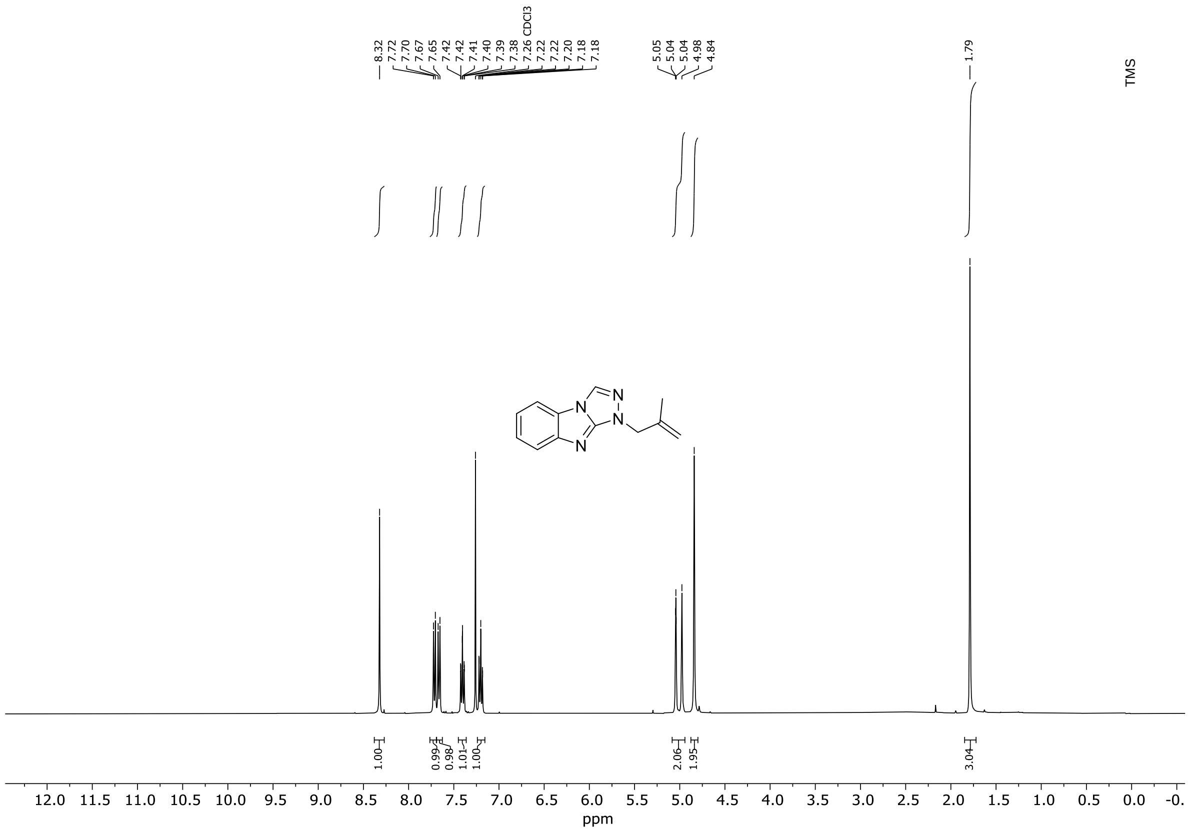

Figure S99 ^{1}H NMR spectrum of 1-(2-methylallyl)-1*H*-benzo[4,5]imidazo[2,1-*c*][1,2,4]triazole (**6h**) (400 MHz, $CDCl_3$, 298 K).

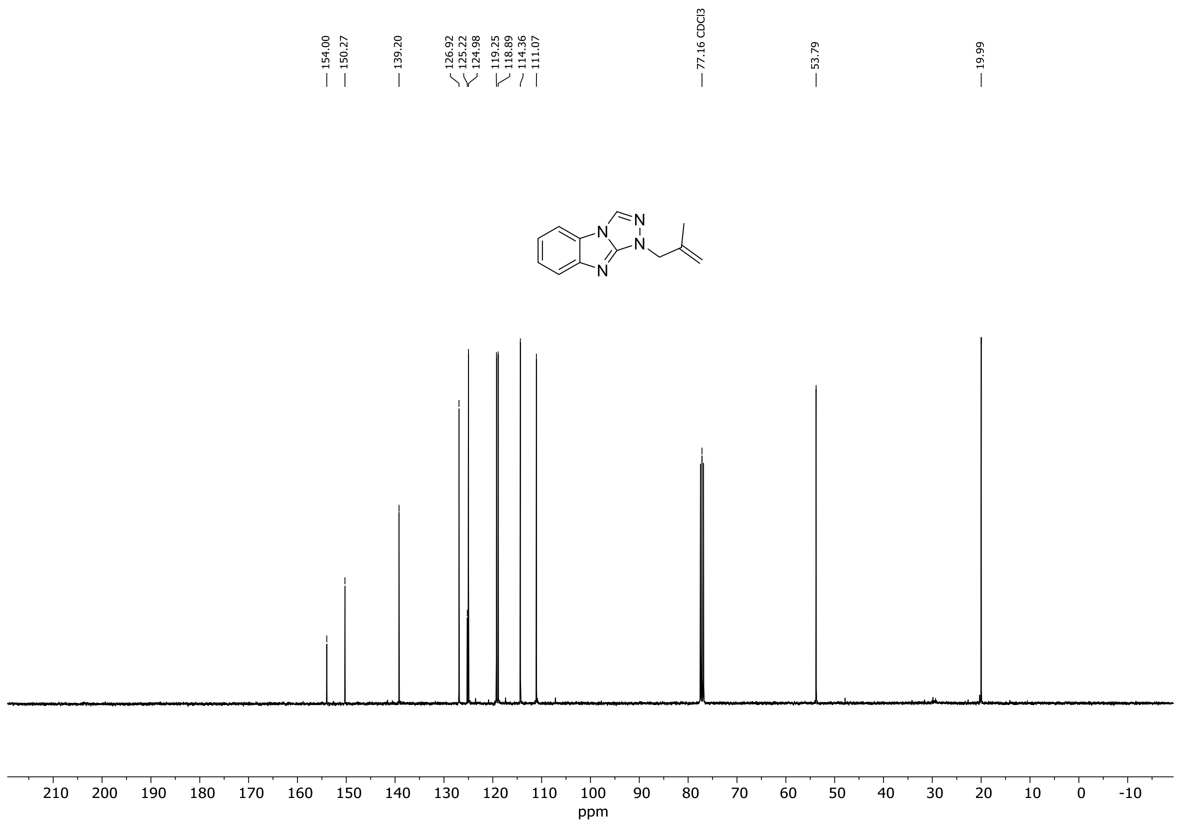

Figure S100: $^{13}C\{^1H\}$ NMR spectrum of 1-(2-methylallyl)-1*H*-benzo[4,5]imidazo[2,1-*c*][1,2,4]triazole (**6h**) (100 MHz, $CDCl_3$, 298 K).

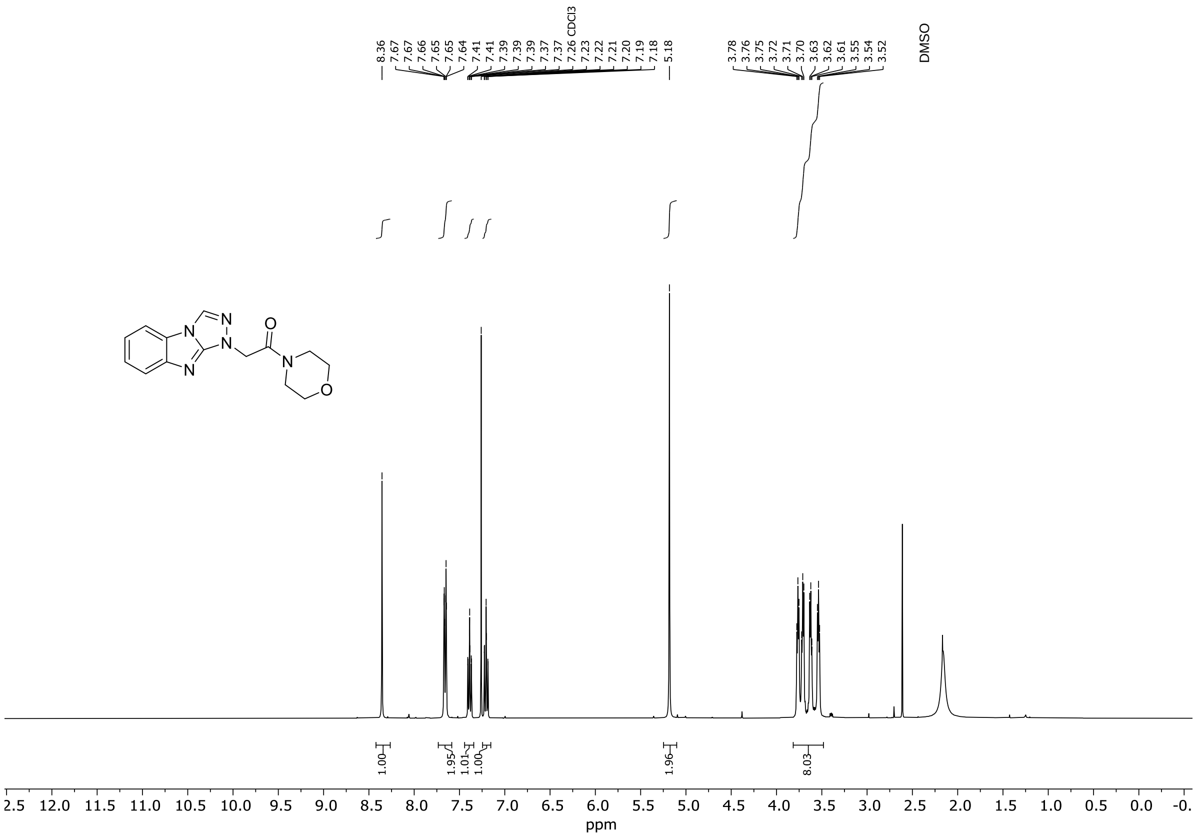

Figure S101: ^{1}H NMR spectrum of 2-(1*H*-benzo[4,5]imidazo[2,1-*c*][1,2,4]triazol-1-yl)-1-morpholinoethan-1-one (**6i**) (400 MHz, $CDCl_3$, 298 K).

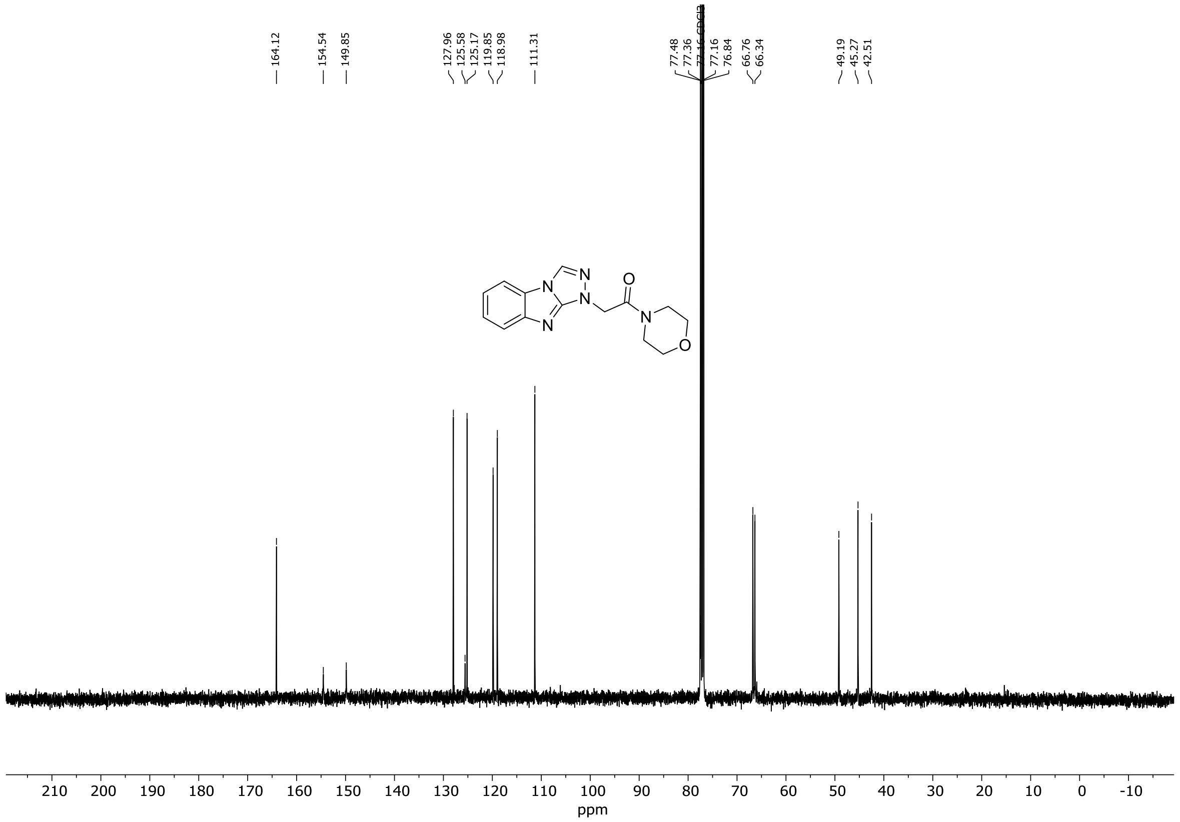

Figure S102: $^{13}C\{^{1}H\}$ NMR spectrum of 2-(1*H*-benzo[4,5]imidazo[2,1-*c*][1,2,4]triazol-1-yl)-1-morpholinoethan-1-one (**6i**) (100 MHz, $CDCl_3$, 298 K).

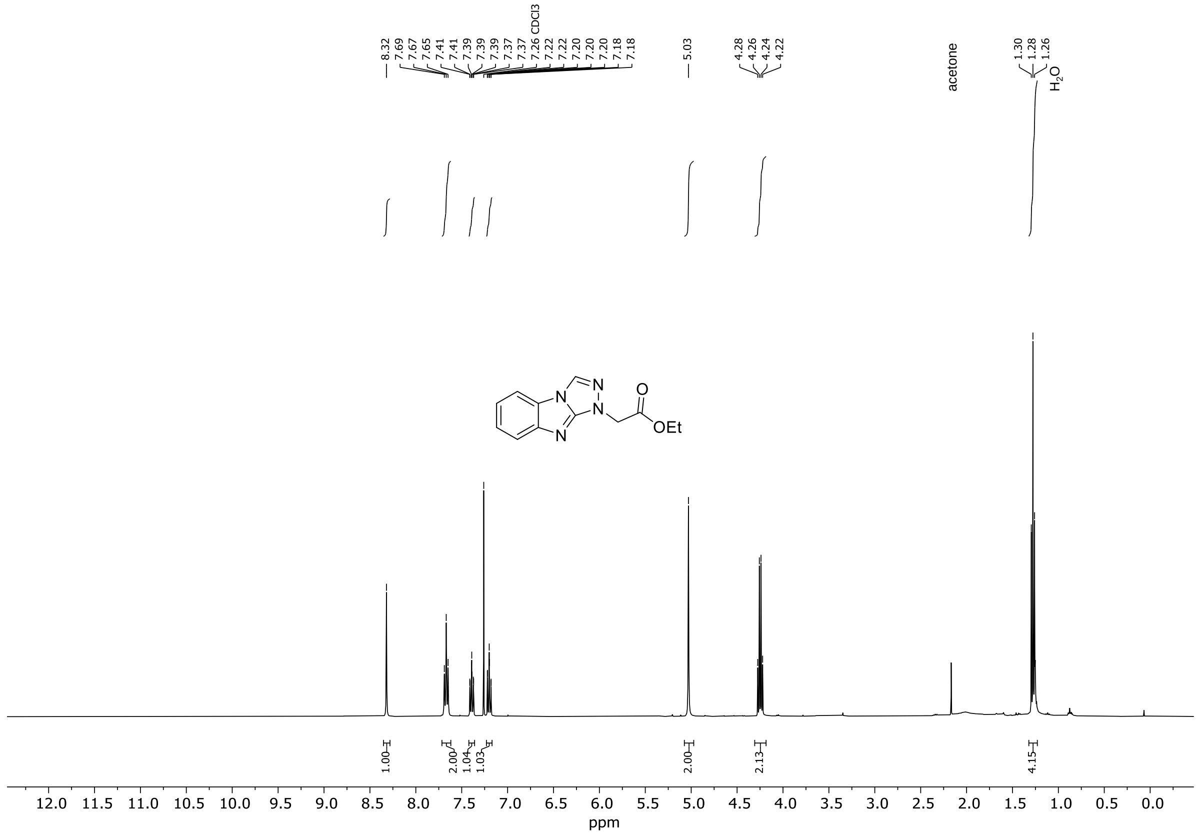

Figure S103: ^{1}H NMR spectrum of ethyl 2-(1*H*-benzo[4,5]imidazo[2,1-*c*][1,2,4]triazol-1-yl)acetate (**6j**) (400 MHz, $CDCl_3$, 298 K).

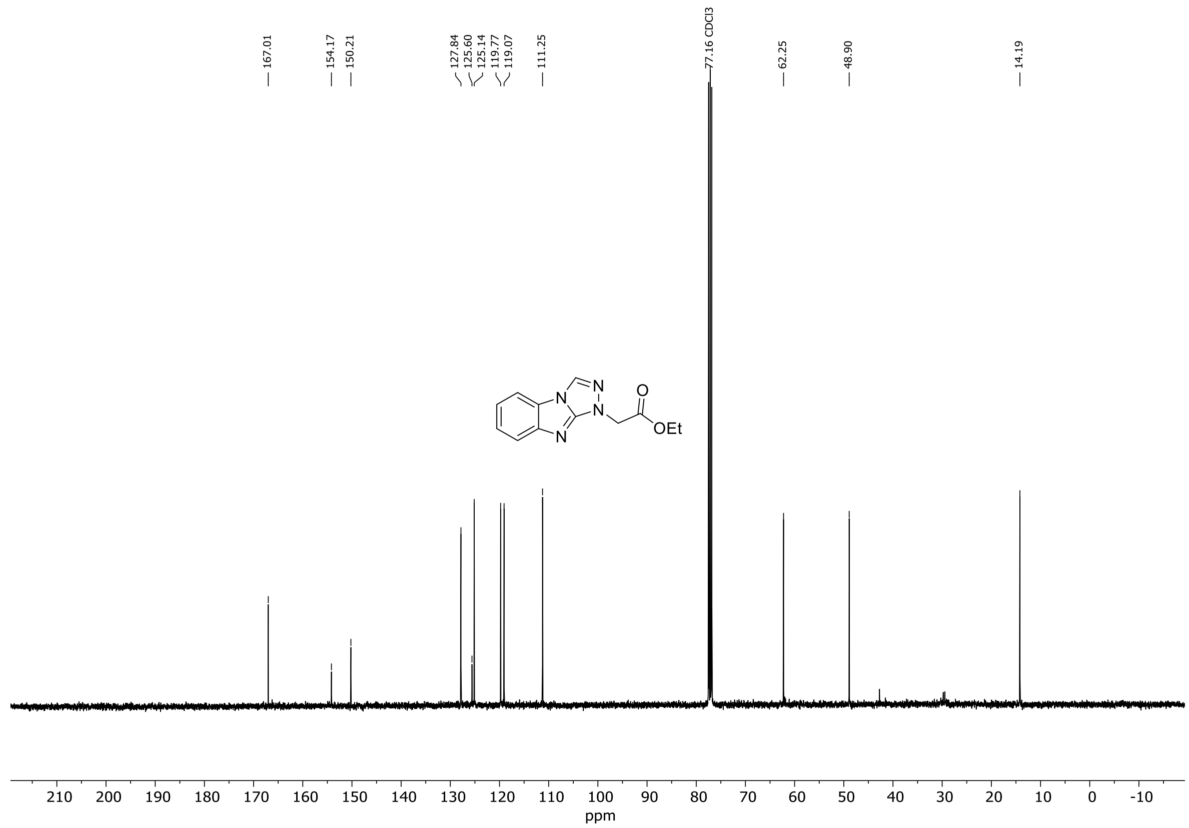

Figure S104: $^{13}C\{^{1}H\}$ NMR spectrum of ethyl 2-(1*H*-benzo[4,5]imidazo[2,1-*c*][1,2,4]triazol-1-yl)acetate (**6j**) (100 MHz, $CDCl_3$, 298 K).

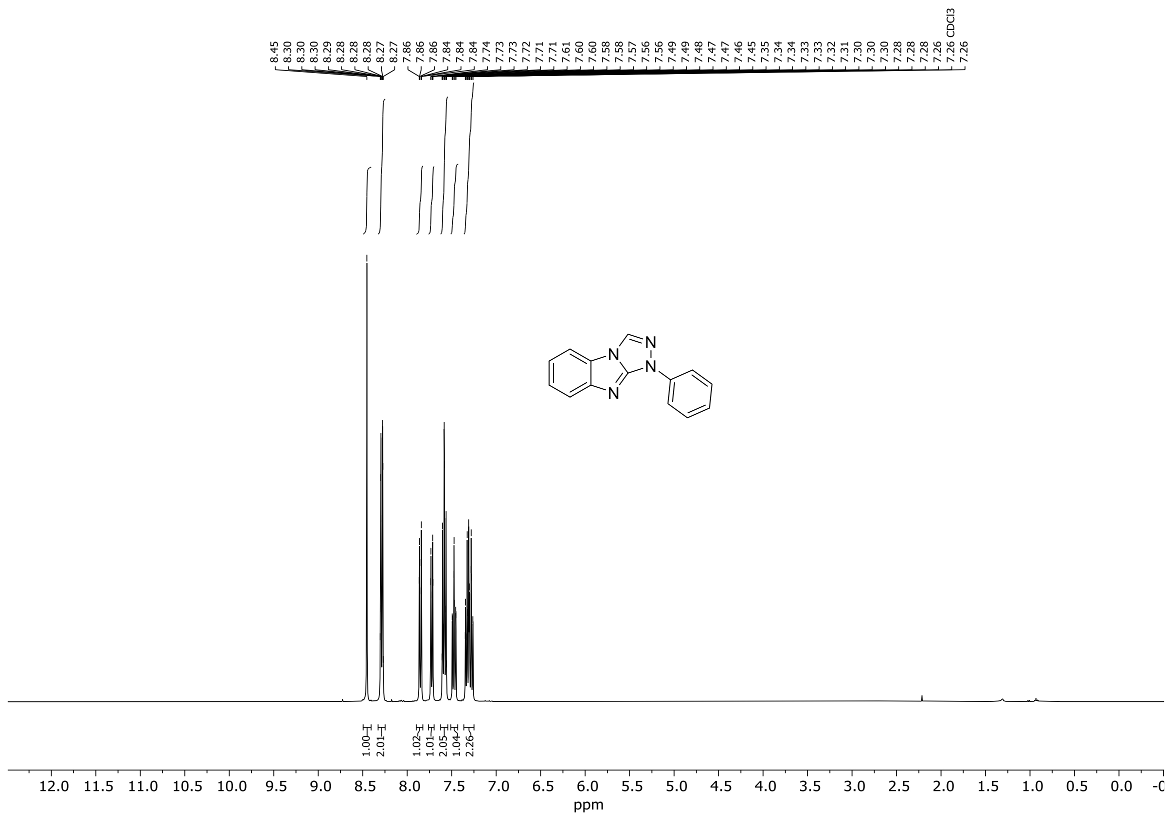

Figure S105: ^{1}H NMR spectrum of 1-phenyl-1*H*-benzo[4,5]imidazo[2,1-*c*][1,2,4]triazole (**7a**) (400 MHz, $CDCl_3$, 298 K).

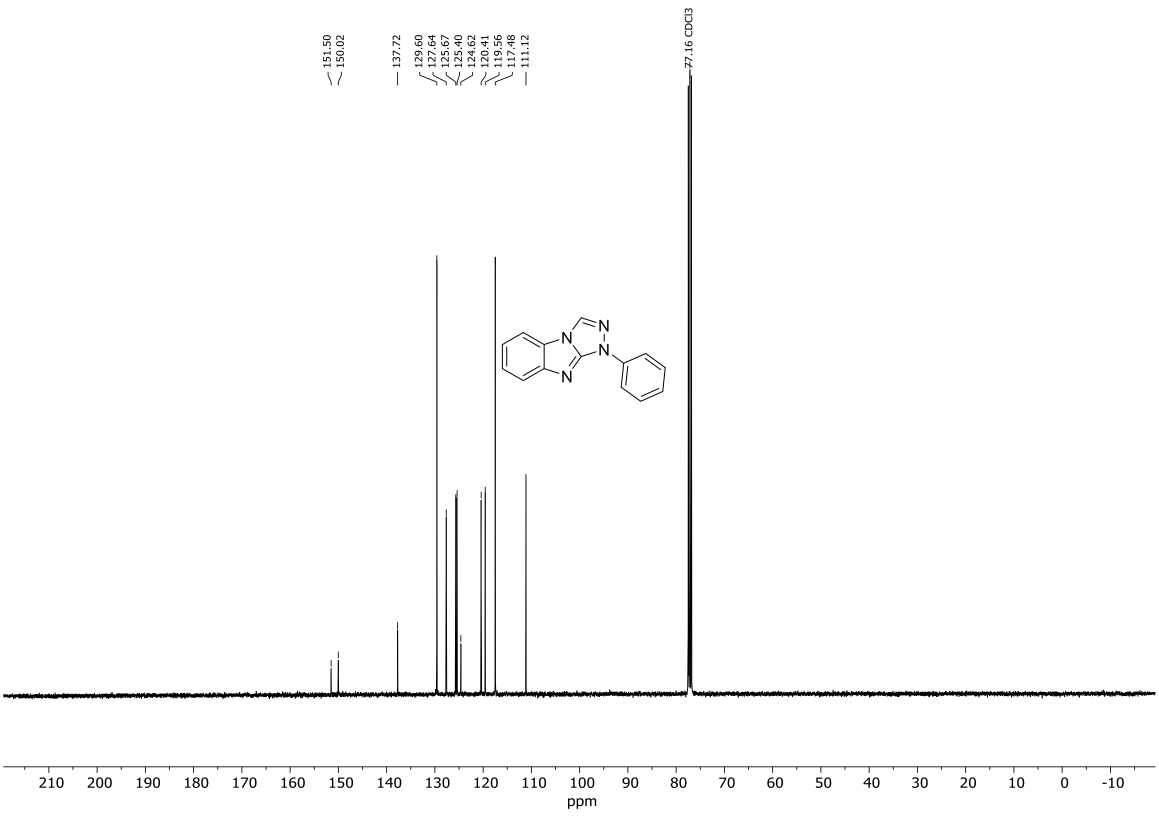

Figure S106: $^{13}C\{^{1}H\}$ NMR spectrum of 1-phenyl-1*H*-benzo[4,5]imidazo[2,1-*c*][1,2,4]triazole (**7a**) (100 MHz, $CDCl_3$, 298 K).

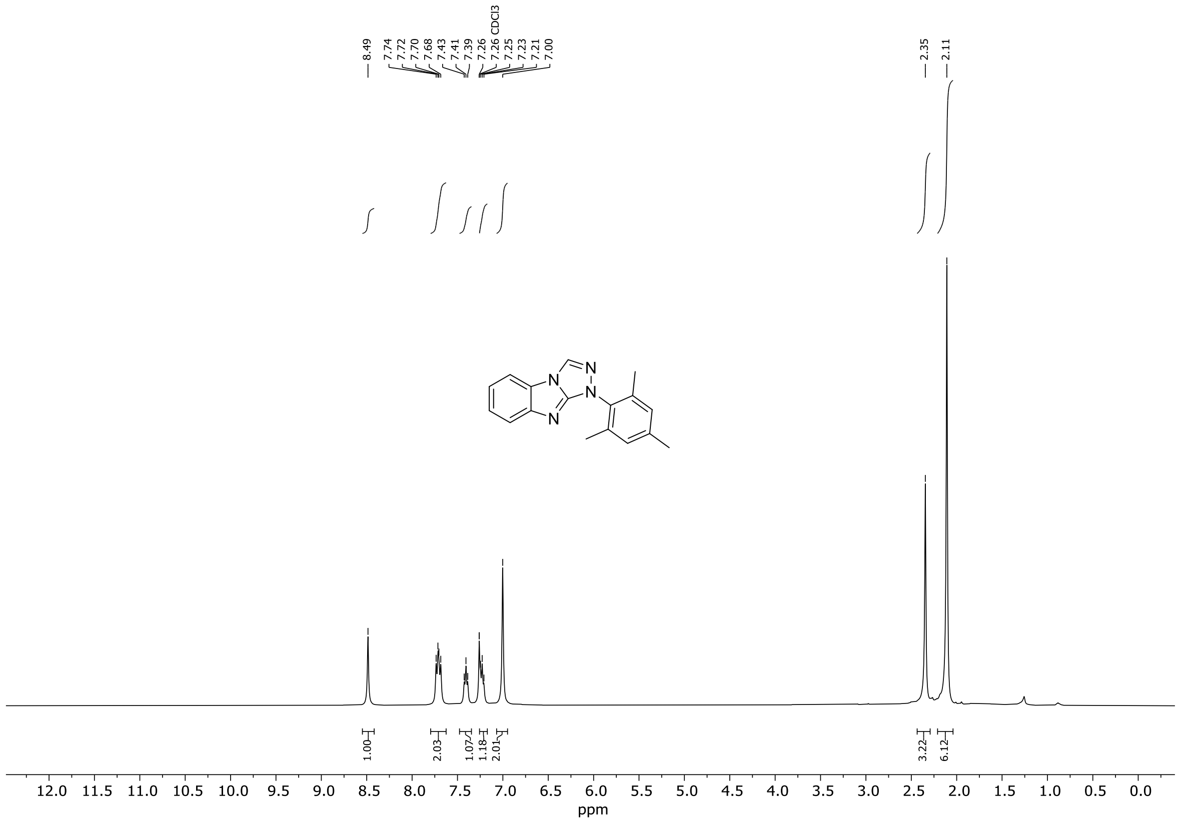

Figure S107: ^{1}H NMR spectrum of 1-mesityl-1*H*-benzo[4,5]imidazo[2,1-*c*][1,2,4]triazole (**7b**) (400 MHz, $CDCl_3$, 298 K).

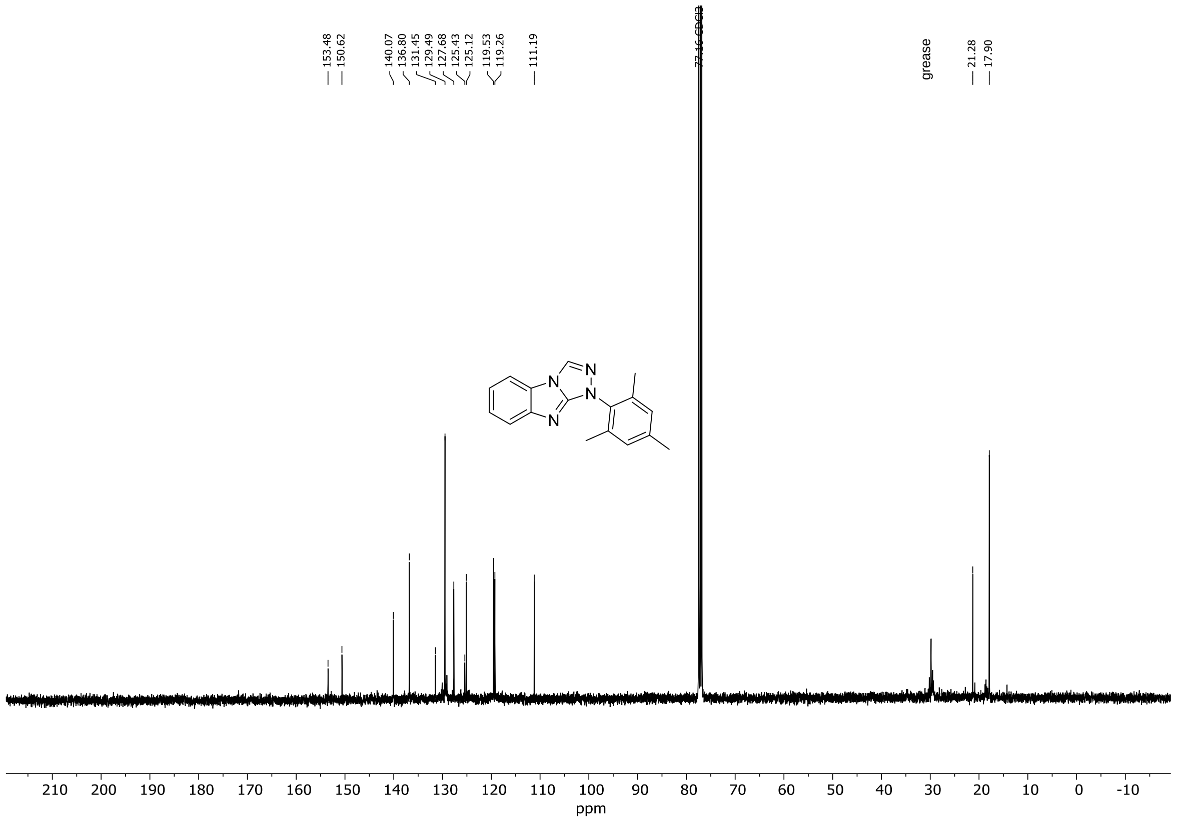

Figure S108: $^{13}C\{^{1}H\}$ NMR spectrum of 1-mesityl-1*H*-benzo[4,5]imidazo[2,1-*c*][1,2,4]triazole (**7b**) (100 MHz, $CDCl_3$, 298 K).

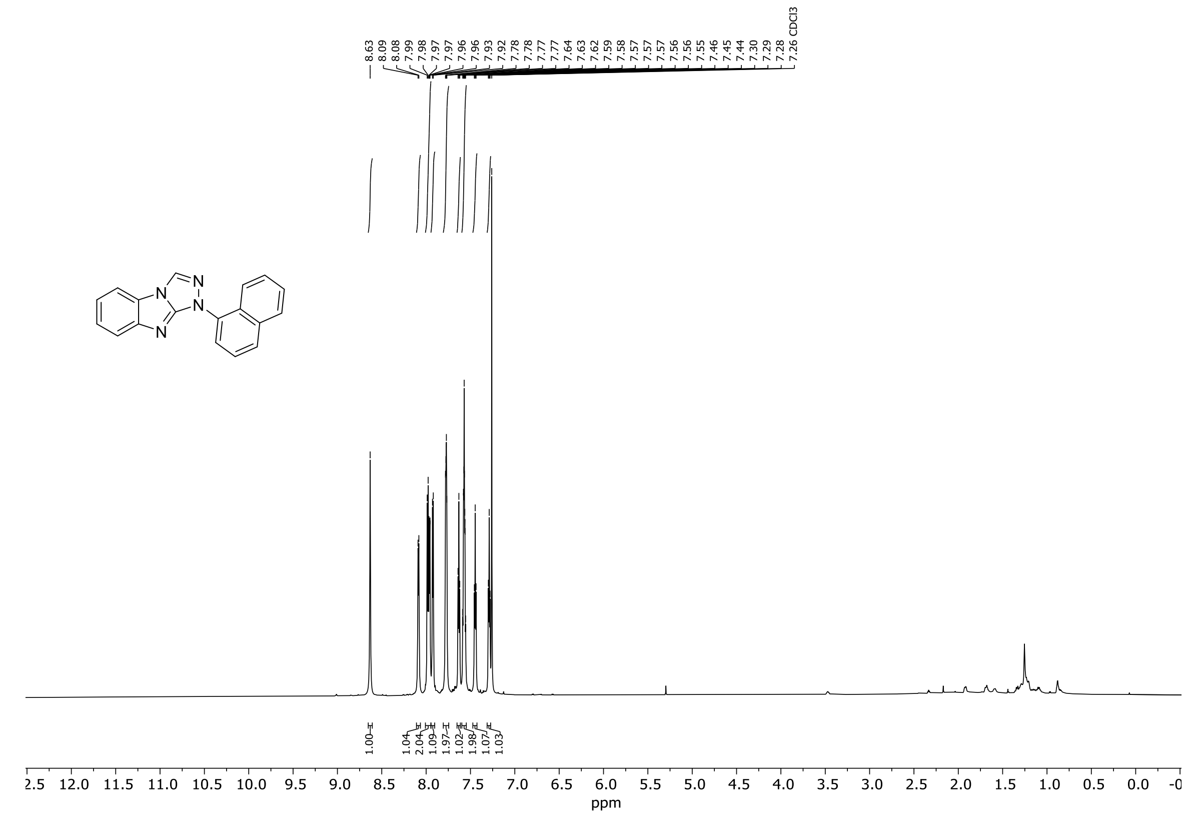

Figure S109: ^{1}H NMR spectrum of 1-(naphthalen-1-yl)-1*H*-benzo[4,5]imidazo[2,1-*c*][1,2,4]triazole (**7c**) (400 MHz, $CDCl_3$, 298 K).

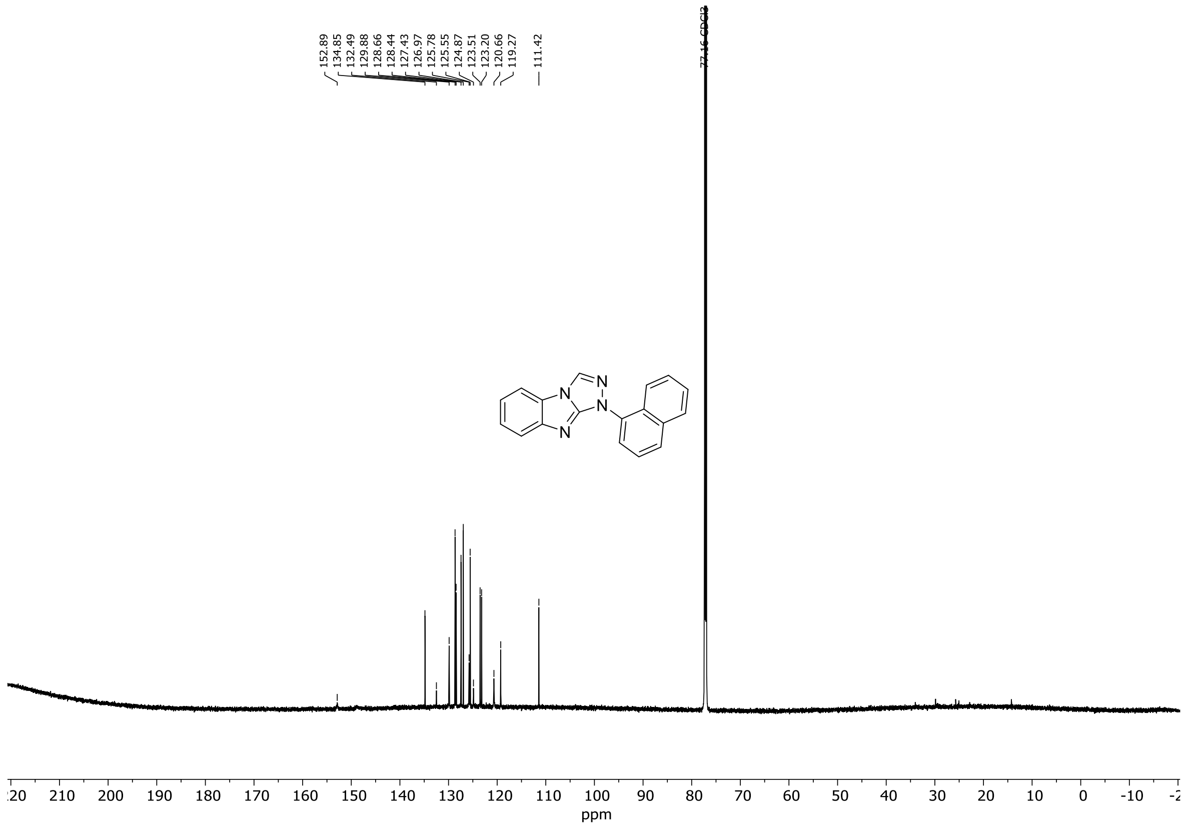

Figure S110: ^{13}C{^{1}H} NMR spectrum of 1-(naphthalen-1-yl)-1*H*-benzo[4,5]imidazo[2,1-*c*][1,2,4]triazole (**7c**) (100 MHz, $CDCl_3$, 298 K).

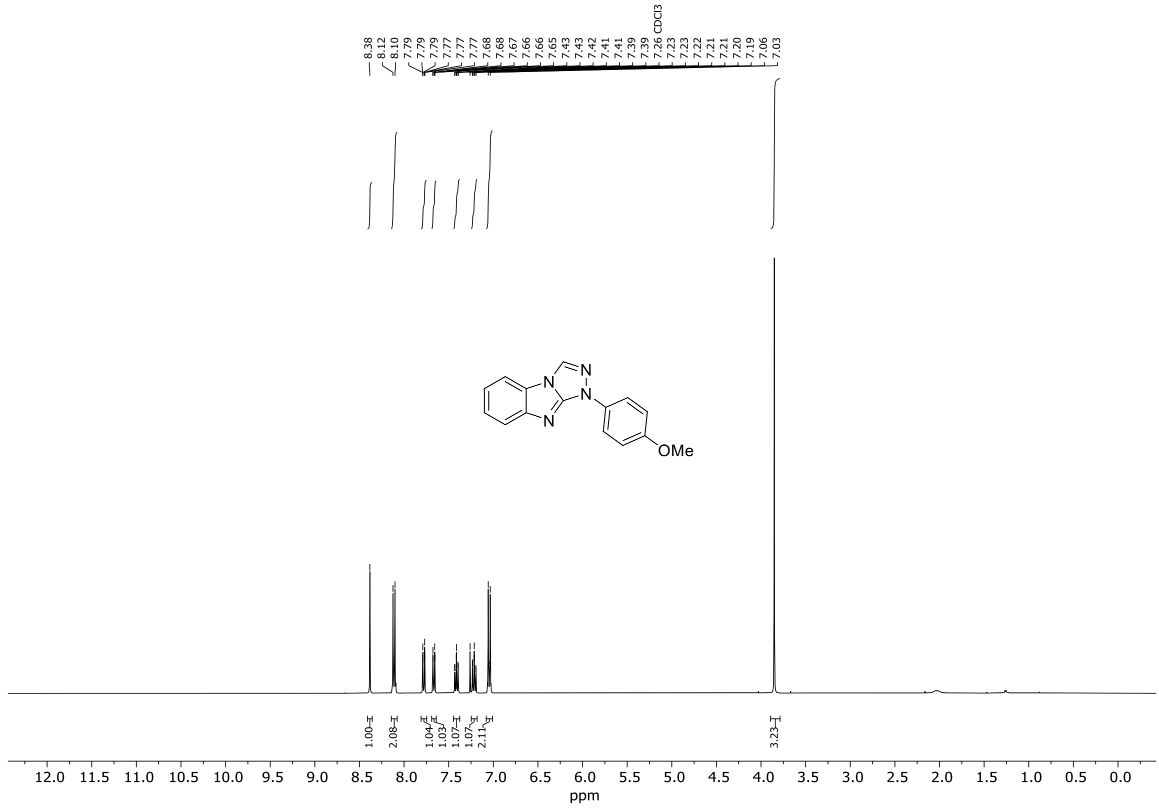

Figure S111: ^{1}H NMR spectrum of 1-(4-methoxyphenyl)-1*H*-benzo[4,5]imidazo[2,1-*c*][1,2,4]triazole (**7d**) (400 MHz, $CDCl_3$, 298 K).

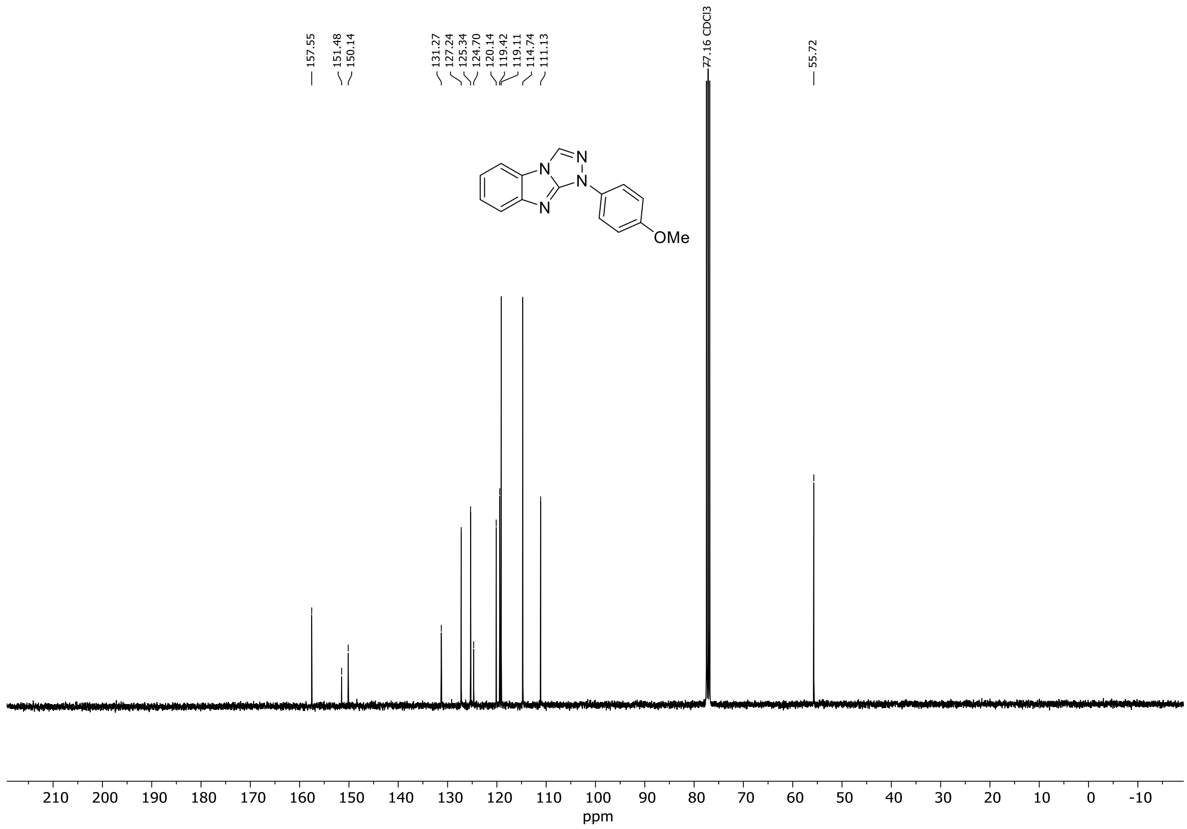

Figure S112: $^{13}C\{^{1}H\}$ NMR spectrum of 1-(4-methoxyphenyl)-1*H*-benzo[4,5]imidazo[2,1-*c*][1,2,4]triazole (**7d**) (100 MHz, $CDCl_3$, 298 K).

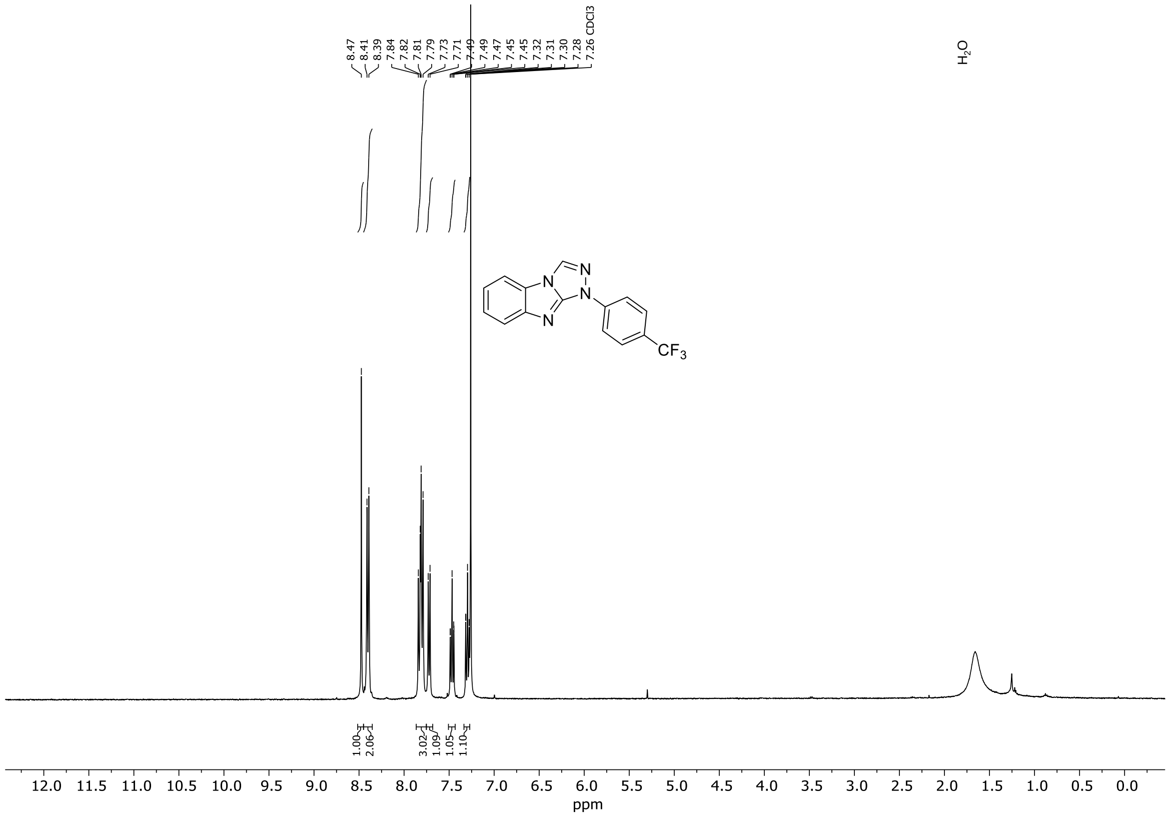

Figure S113: ^{1}H NMR spectrum of 1-(4-(trifluoromethyl)phenyl)-1*H*-benzo[4,5]imidazo[2,1-*c*][1,2,4]triazole (**7e**) (400 MHz, $CDCl_3$, 298 K).

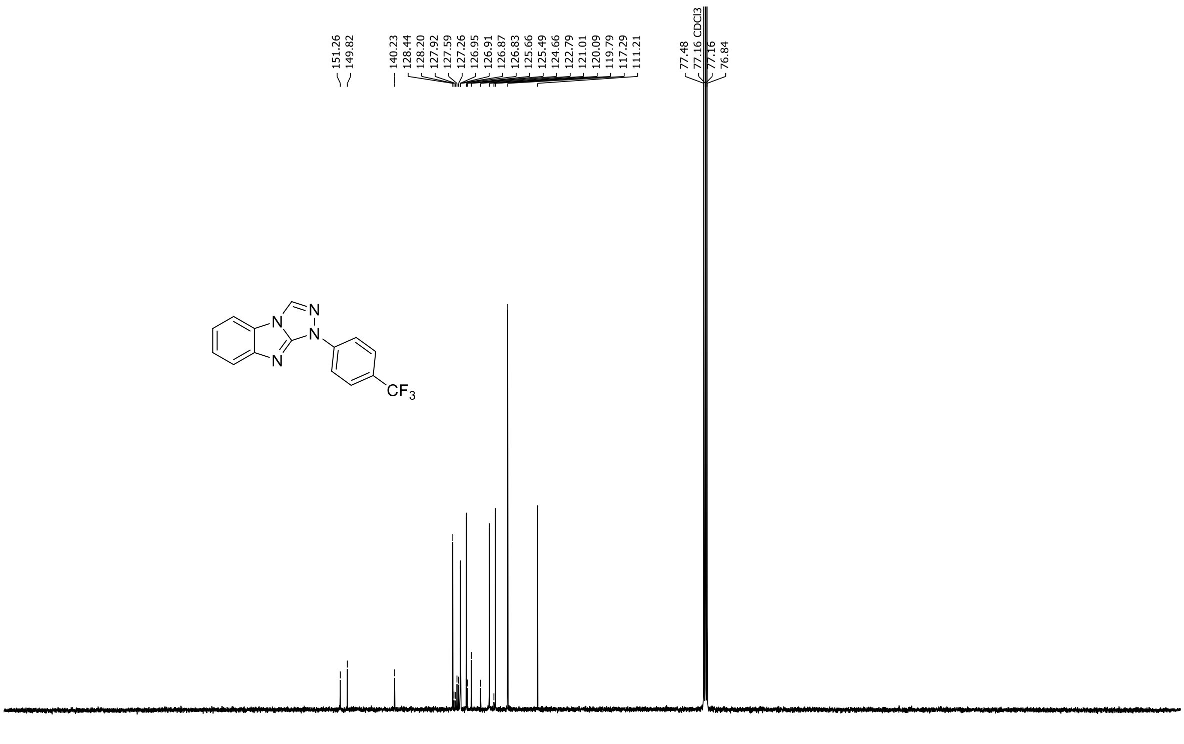

Figure S114: $^{13}C\{^{1}H\}$ NMR spectrum of 1-(4-(trifluoromethyl)phenyl)-1*H*-benzo[4,5]imidazo[2,1-*c*][1,2,4]triazole (**7e**) (100 MHz, $CDCl_3$, 298 K).

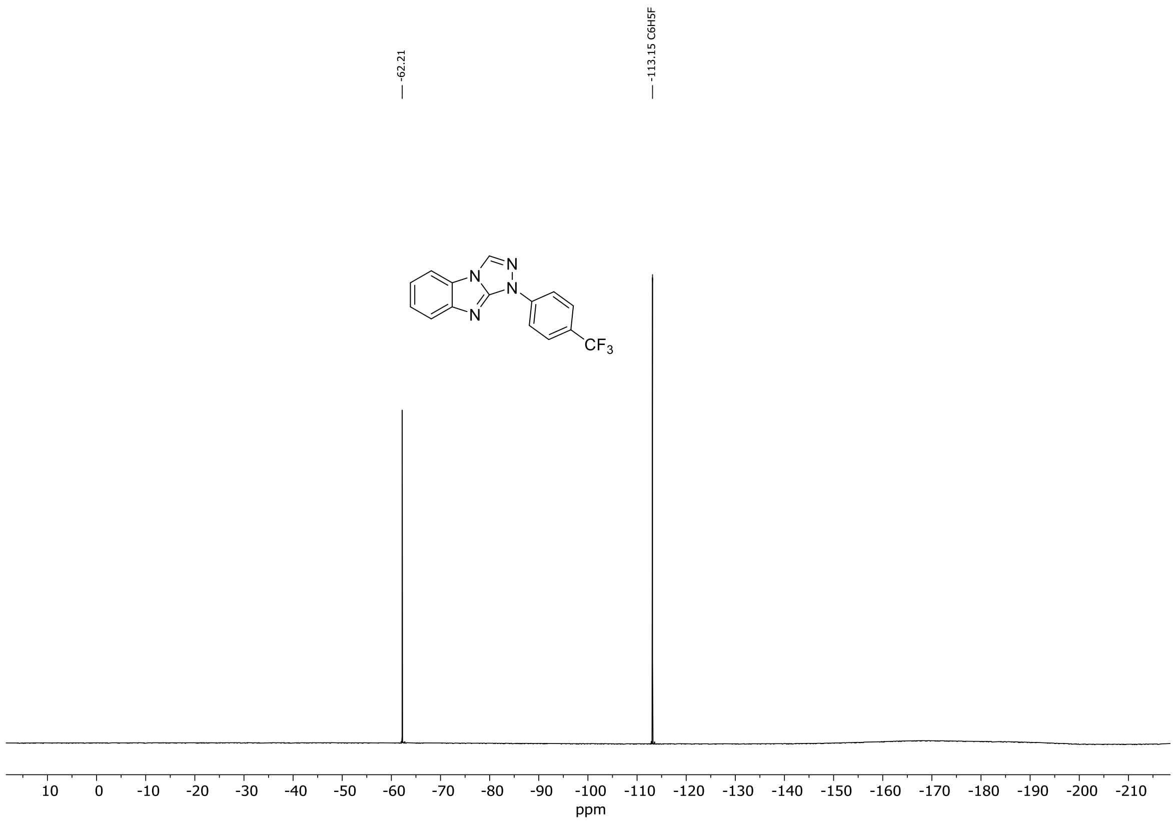

Figure S115: $^{19}F\{^{1}H\}$ NMR spectrum of 1-(4-(trifluoromethyl)phenyl)-1*H*-benzo[4,5]imidazo[2,1-*c*][1,2,4]triazole (**7e**) (376 MHz $CDCl_3$, 298 K, referenced to fluorobenzene).

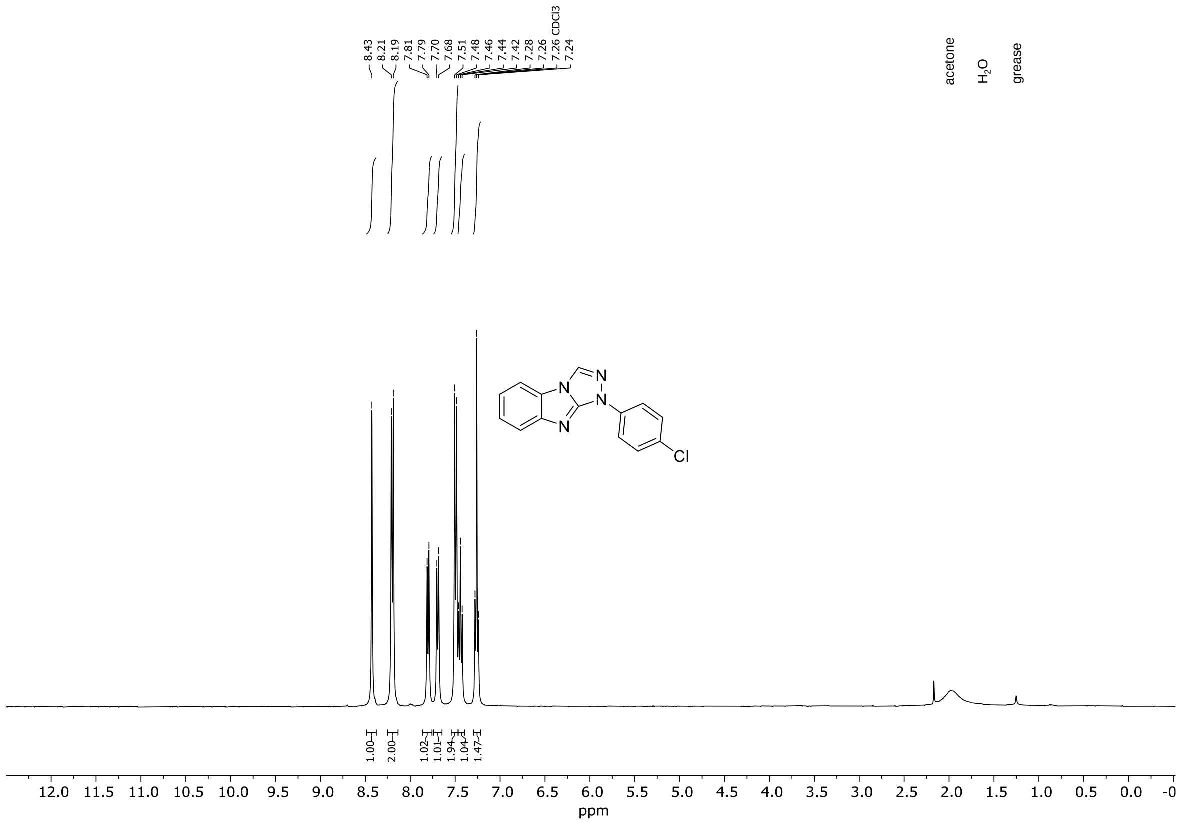

Figure S116: ^{1}H NMR spectrum of 1-(4-chlorophenyl)-1*H*-benzo[4,5]imidazo[2,1-*c*][1,2,4]triazole (**7f**) (400 MHz, $CDCl_3$, 298 K).

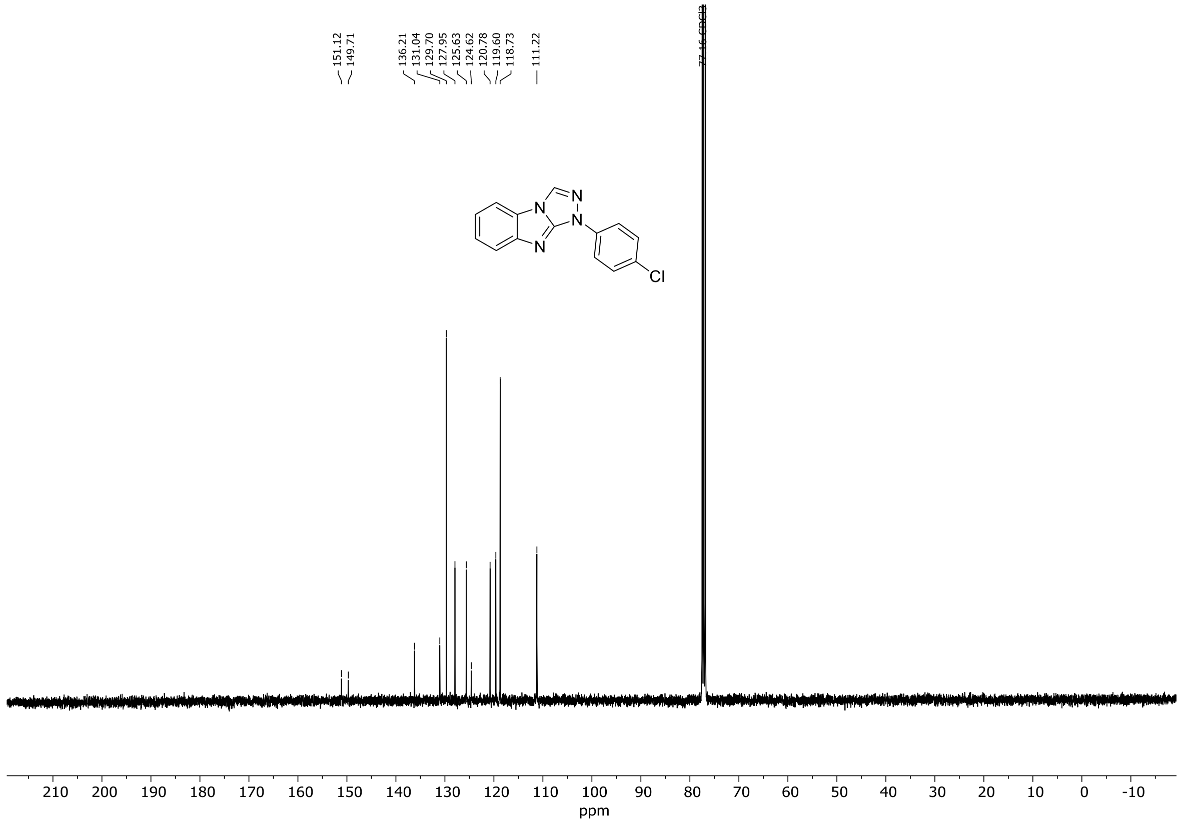

Figure S117: $^{13}C\{^1H\}$ NMR spectrum of 1-(4-chlorophenyl)-1*H*-benzo[4,5]imidazo[2,1-*c*][1,2,4]triazole (**7f**) (100 MHz, $CDCl_3$, 298 K).

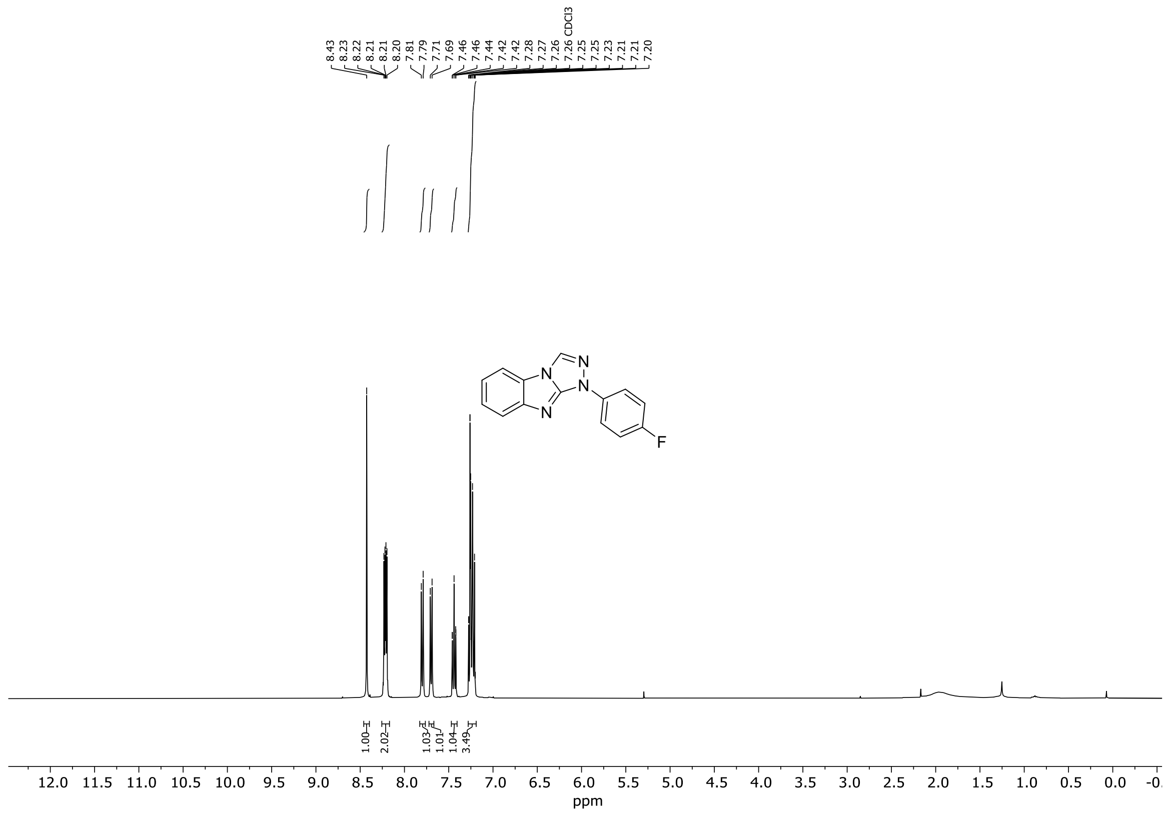

Figure S118: ^1H NMR spectrum of 1-(4-fluorophenyl)-1*H*-benzo[4,5]imidazo[2,1-*c*][1,2,4]triazole (**7g**) (400 MHz, $CDCl_3$, 298 K).

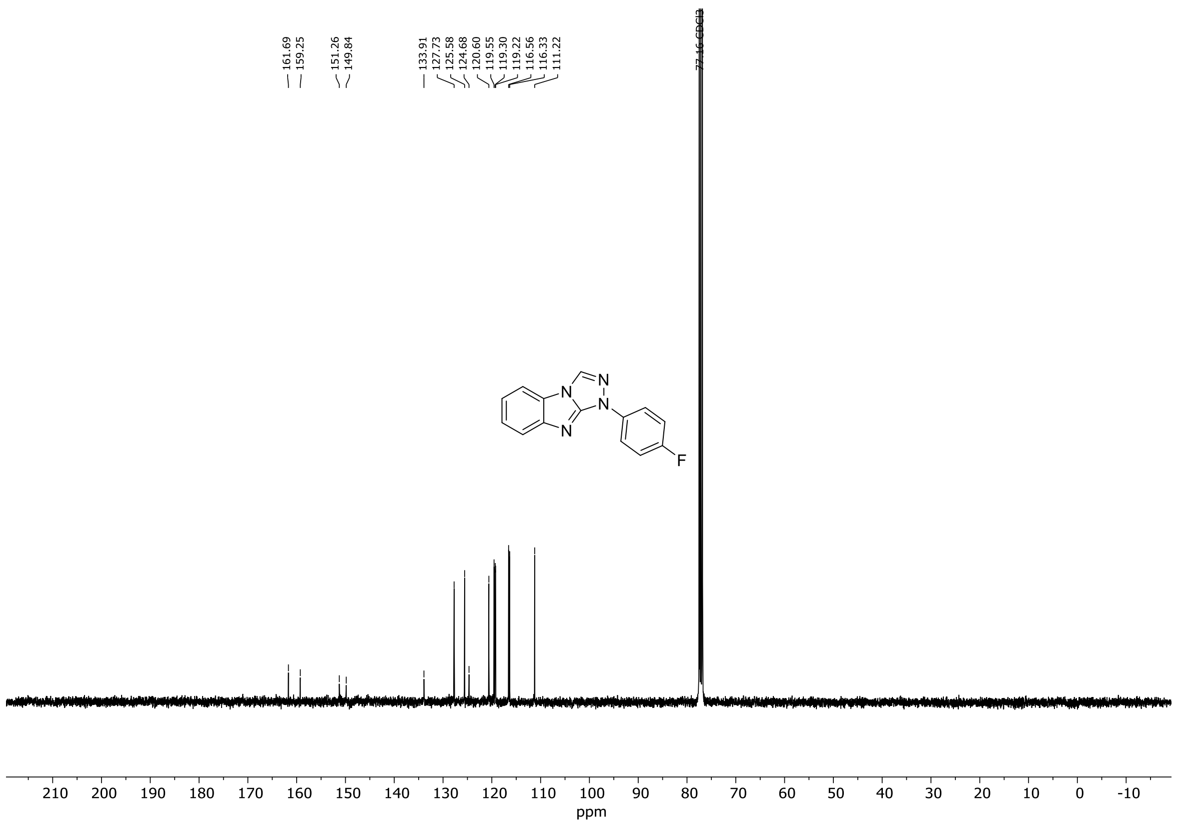

Figure S119: $^{13}C\{^1H\}$ NMR spectrum of 1-(4-fluorophenyl)-1*H*-benzo[4,5]imidazo[2,1-*c*][1,2,4]triazole (**7g**) (100 MHz, $CDCl_3$, 298 K).

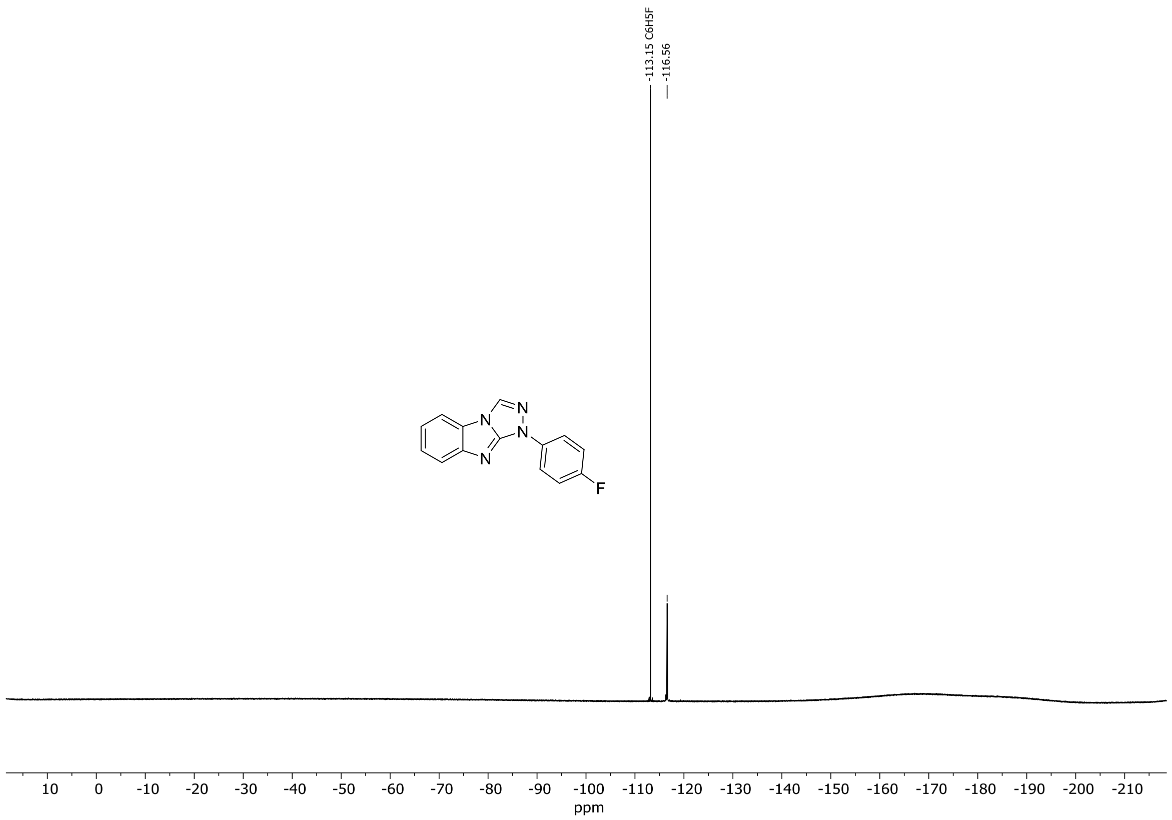

Figure S120: $^{19}F\{^1H\}$ NMR spectrum of 1-(4-fluorophenyl)-1*H*-benzo[4,5]imidazo[2,1-*c*][1,2,4]triazole (**7g**) (376 MHz, $CDCl_3$, 298 K, referenced to fluorobenzene).

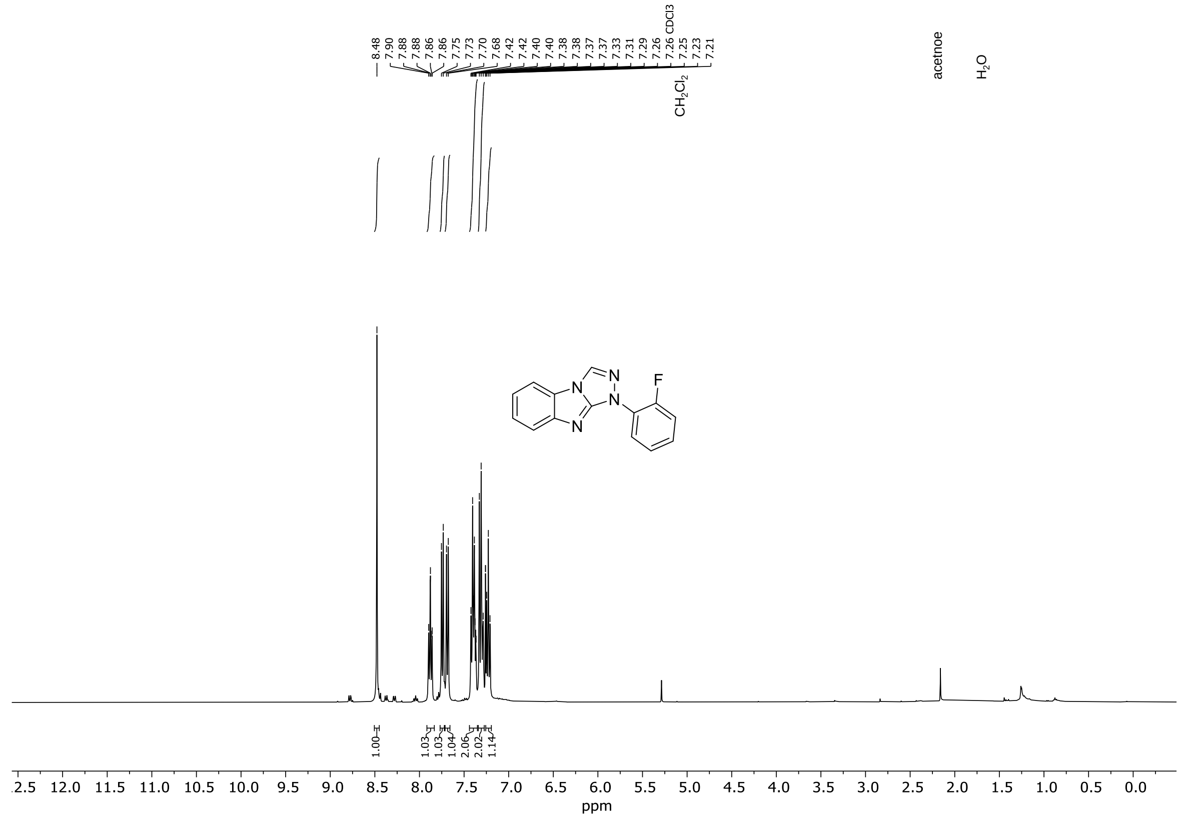

Figure S121: ^{1}H NMR spectrum of 1-(2-fluorophenyl)-1*H*-benzo[4,5]imidazo[2,1-*c*][1,2,4]triazole (**7h**) (400 MHz, $CDCl_3$, 298 K).

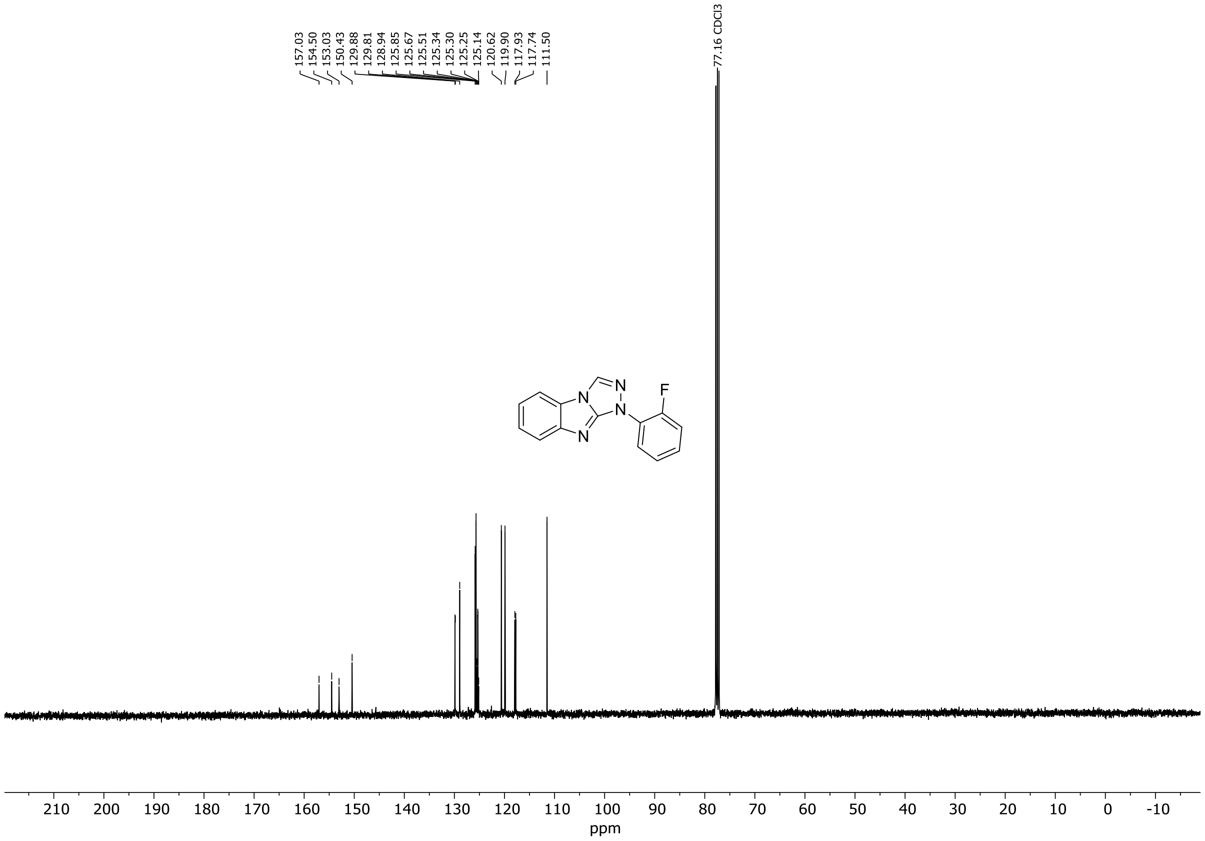

Figure S124: $^{13}C\{^{1}H\}$ NMR spectrum of 1-(2-fluorophenyl)-1*H*-benzo[4,5]imidazo[2,1-*c*][1,2,4]triazole (**7h**) (100 MHz, $CDCl_3$, 298 K).

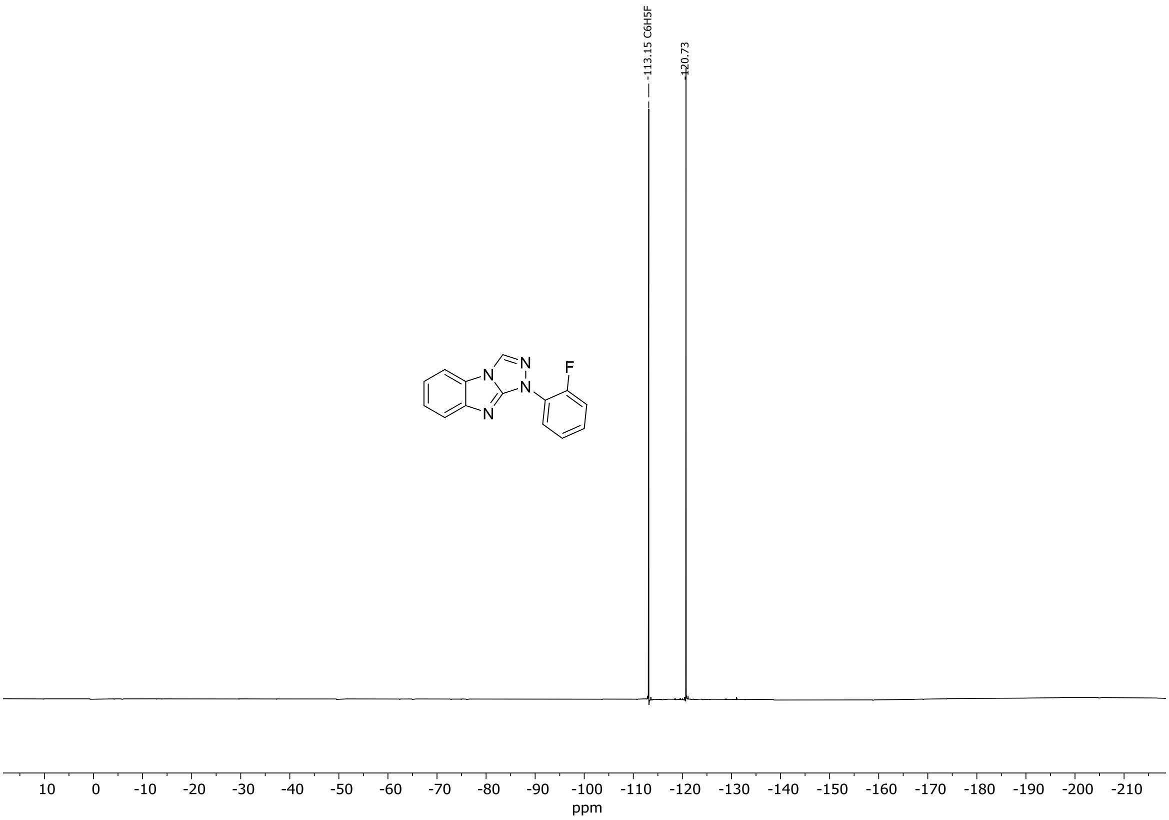

Figure S125: $^{19}F\{^1H\}$ NMR spectrum of 1-(2-fluorophenyl)-1*H*-benzo[4,5]imidazo[2,1-*c*][1,2,4]triazole (**7h**) (376 MHz, $CDCl_3$, 298 K, referenced to fluorobenzene).

4. LCMS data

a) Regular order of addition

Reaction performed in an LCMS vial on a 0.01 mmol scale. Order of addition: **4a** dissolved in DMSO, addition of I_2 (as DMSO solution), immediately followed by DBU (as DMSO solution).

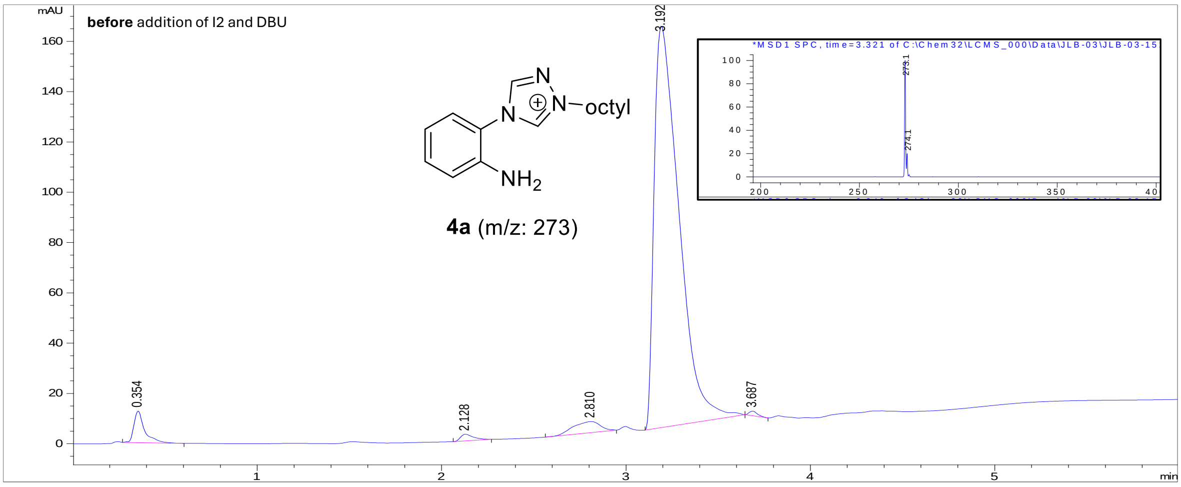

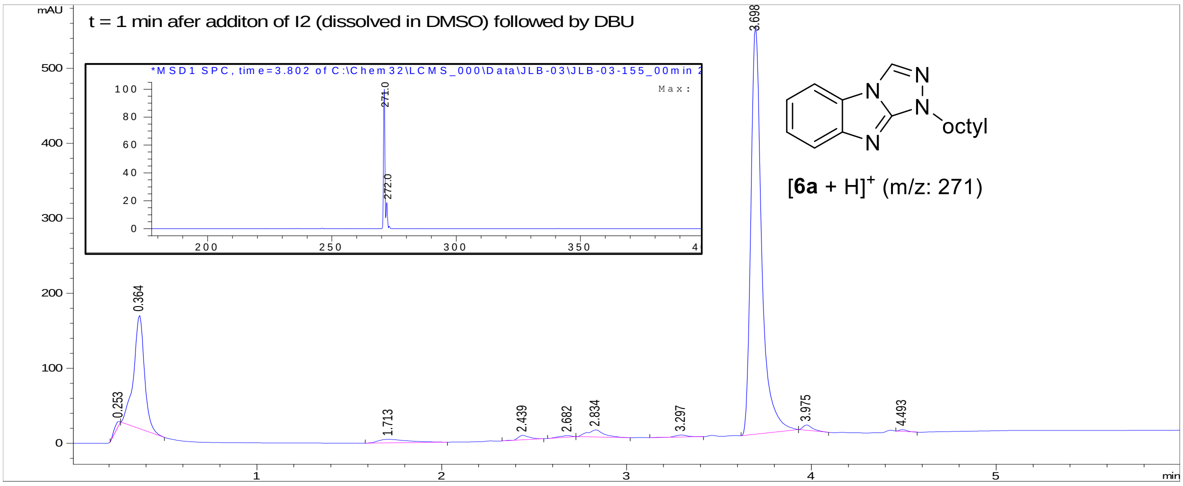

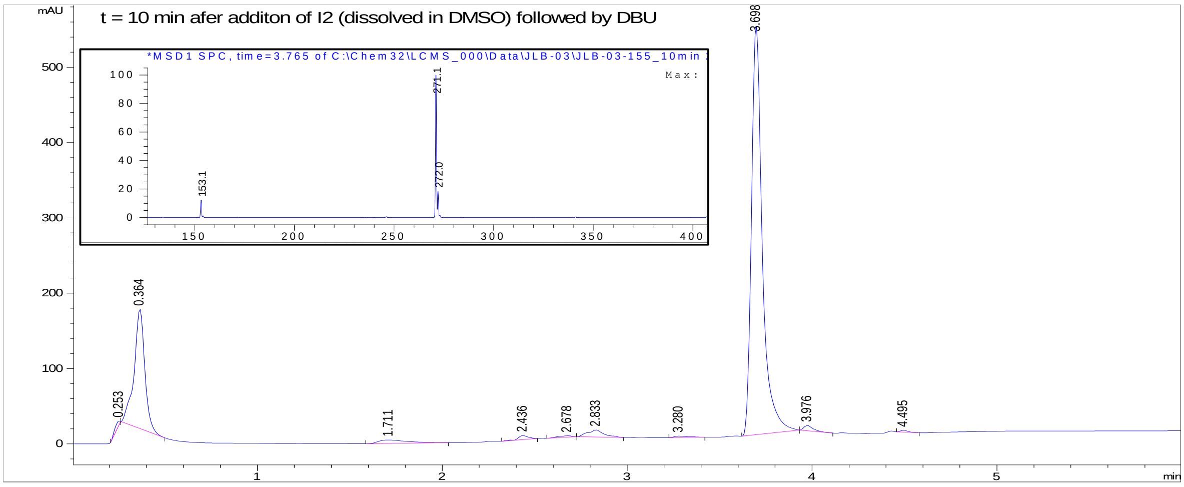

Figure S126: UV traces of LCMS measurements. Top: **4a** in DMSO. Middle: immediately after addition of I_2 followed by DBU. Bottom: after 10 minutes.

b) Inverse order of addition of DBU and iodine

Reaction performed in an LCMS vial on a 0.01 mmol scale. Order of addition: **4a** dissolved in DMSO, addition of DBU (as DMSO solution), followed by addition of I_2 (as DMSO solution).

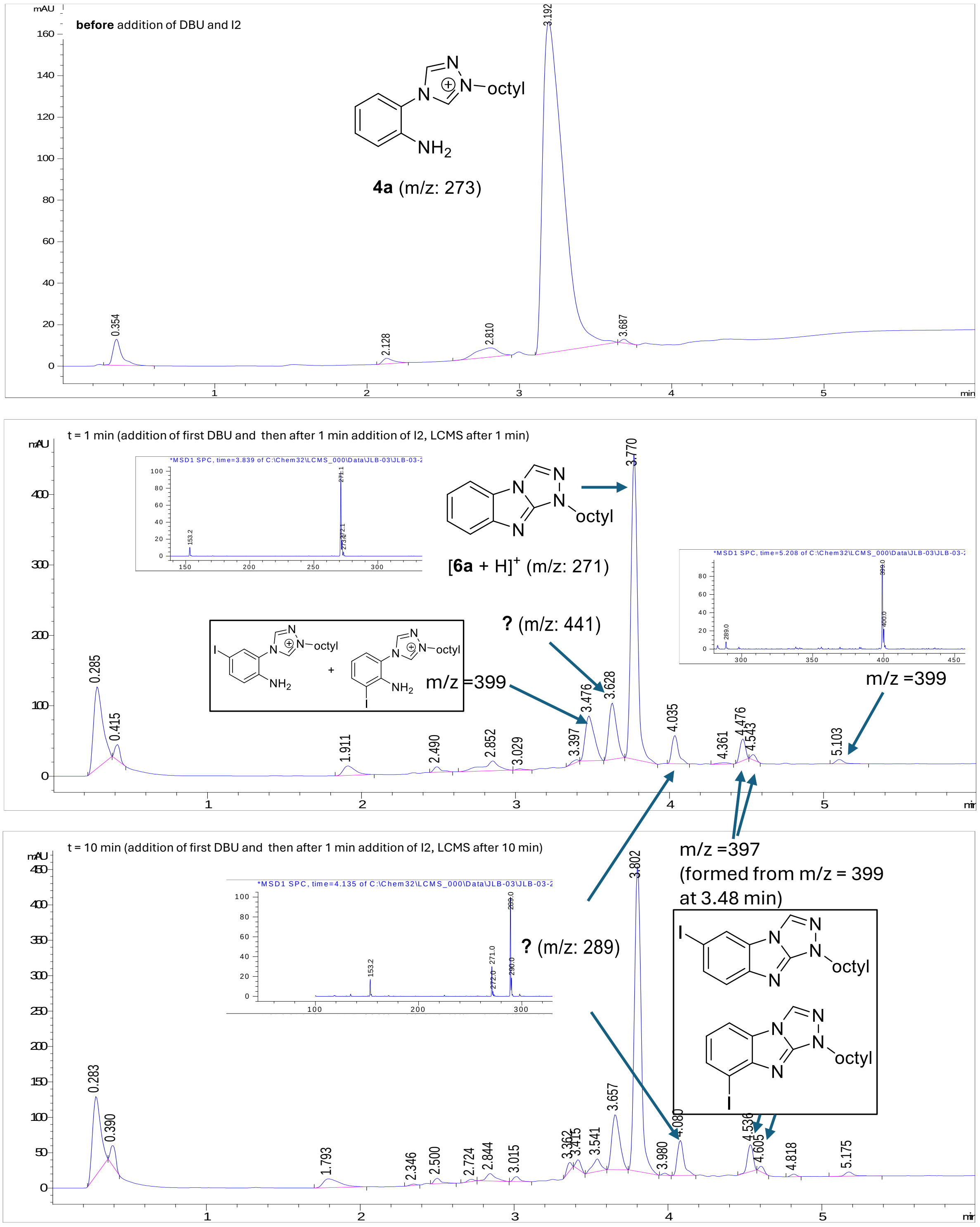

Figure S127: UV traces of LCMS measurements. Top: **4a** in DMSO. Middle: immediately after addition of DBU followed by I_2. Bottom: after 10 minutes of reaction time..
